# Supplementary material for: Impact of the COVID-19 pandemic on domiciliary care workers in Wales, UK: a data linkage cohort study using the SAIL Databank
Source: BMJ Open. 2023 Jun 1;13(6):e070637. doi: 10.1136/bmjopen-2022-070637 (PMC10255029; doi:10.1136/bmjopen-2022-070637)
Supplement: Supplementary data [file bmjopen-2022-070637supp001.pdf]

Supplementary material

Supplementary material 1: Data sources used in the OSCAR study

SAIL acronym	Name	Description	Meta-data link
ADDE	Annual District Death Extract	Monthly register of all deaths relating to Welsh residents, including those that died out of Wales from the Office for National Statistics (ONS).	https://web.www.healthdatagateway.org/dataset/15cf4241-abad-4dcc-95b0-8cd7c02be999
CENW	ONS 2011 Census Wales	A census in the UK is a count of all people and households. The census data held in SAIL was from the census held on 27 March 2011.	https://web.www.healthdatagateway.org/dataset/fc24482c-0f9b-445f-af7a-8598627c3b15
DSCW	Domiciliary Social Care Worker Dataset	Under the Care Standards Act 2000, SCW maintain a register of DCWs in Wales. The DSCW data was transferred securely from SCW to the SAIL Databank and made available as a core-restricted data source.	NA
EDDS	Emergency Department Dataset	Data captured for all NHS Wales Accident and Emergency department attendances. Data are collected and coded at each hospital	https://web.www.healthdatagateway.org/dataset/75c4dcb8-33bf-43f4-b2bb-db51b6621b2c
PATD	COVID-19 Test results	Daily update of all pillar 1 and pillar 2 PCR tests administered and associated results for Welsh residents and those receiving a test in Wales	https://web.www.healthdatagateway.org/dataset/f5f6d882-163d-4ef1-a53e-000fba409480
PEDW	Patient Episode Dataset for Wales	Weekly update of all inpatient and day case activity undertaken in NHS Wales plus data on Welsh residents treated in English Trusts	https://web.www.healthdatagateway.org/dataset/4c33a5d2-164c-41d7-9797-dc2b008cc852
WDSD	Welsh Demographic Service Dataset	Weekly update of all individuals registered with a Welsh general practice, inc. address and practice history	https://web.www.healthdatagateway.org/dataset/8a8a5e90-b0c6-4839-bcd2-c69e6e8dca6d
WLGP	Wales Longitudinal General Practice	Monthly update of all attendance and clinical information for SAIL submitting general practices (~80% national coverage)	https://web.www.healthdatagateway.org/dataset/33fc3ffd-aa4c-4a16-a32f-0c900aaea3d2

Cannings-John R, *et al. BMJ Open* 2023; 13:e070637. doi: 10.1136/bmjopen-2022-070637

Supplementary material 2: Coding lists for OSCAR outcomes

Primary outcome

Confirmed-COVID: Confirmed COVID-19 will be defined as the earliest of the following events:

- Positive polymerase chain reaction (PCR) test result (Source: PATD);
- Hospital admission (main or secondary diagnostic ICD-10 code of U07.1 position in the admitted patient care component of PEDW);
- Death registration including a diagnosis ICD-10 code U07.1 (as underlying or contributing cause) from COVID-19 (Source: ADDE);
- COVID-19 diagnosis Read code (Source: WLGP).

TYPE	CODE	DESCRIPTION
ICD-10	U071	COVID-19 virus identified
READ	A795.	Coronavirus infection
READ	A7951	Disease caused by 2019-nCoV (novel coronavirus)
READ	A7952	COVID-19 confirmed by laboratory test
READ	A7953	COVID-19 confirmed using clinical diagnostic criteria

Secondary outcomes

Suspected COVID-19

We will ascertain DCWs with a suspected COVID-19 diagnosis taking the earliest of:

- Negative PCR or serology/antigen test from the COVID-19 test data (Source: PATD);
- Diagnosis ICD-10 code (U07.2) main or secondary diagnostic code position (Source: PEDW);
- Suspected COVID-19 Read codes (Source: WLGP).

TYPE	CODE	DESCRIPTION
ICD-10	U072	COVID-19 virus not identified
READ	1JX..	Suspected coronavirus infection
READ	1JX1.	Suspected coronavirus infection
READ	65PW.	Coronavirus contact
READ	65PW1	Exposure to 2019-nCoV (novel coronavirus) infection

Mental Health

(A) Hospital admissions (ICD-10)

CODE	DESCRIPTION
F20	Schizophrenia
F200	Paranoid schizophrenia
F201	Hebephrenic schizophrenia
F202	Catatonic schizophrenia
F203	Undifferentiated schizophrenia
F204	Post-schizophrenic depression

Cannings-John R, *et al. BMJ Open* 2023; 13:e070637. doi: 10.1136/bmjopen-2022-070637

CODE	DESCRIPTION
F205	Residual schizophrenia
F206	Simple schizophrenia
F208	Other schizophrenia
F209	Schizophrenia, unspecified
F21X	Schizotypal disorder
F22	Persistent delusional disorders
F220	Delusional disorder
F228	Other persistent delusional disorders
F229	Persistent delusional disorder, unspecified
F23	Acute and transient psychotic disorders
F230	Acute polymorphic psychotic disorder without symptoms of schizophrenia
F231	Acute polymorphic psychotic disorder with symptoms of schizophrenia
F232	Acute schizophrenia-like psychotic disorder
F233	Other acute predominantly delusional psychotic disorders
F238	Other acute and transient psychotic disorders
F239	Acute and transient psychotic disorder, unspecified
F24X	Induced delusional disorder
F25	Schizoaffective disorders
F250	Schizoaffective disorder, manic type
F251	Schizoaffective disorder, depressive type
F252	Schizoaffective disorder, mixed type
F258	Other schizoaffective disorders
F259	Schizoaffective disorder, unspecified
F28X	Other nonorganic psychotic disorders
F29X	Unspecified nonorganic psychosis
F30	Manic episode
F300	Hypomania
F301	Mania without psychotic symptoms
F302	Mania with psychotic symptoms
F308	Other manic episodes
F309	Manic episode, unspecified
F31	Bipolar affective disorder
F310	Bipolar affective disorder, current episode hypomanic
F311	Bipolar affective disorder, current episode manic without psychotic symptoms
F312	Bipolar affective disorder, current episode manic with psychotic symptoms
F313	Bipolar affective disorder, current episode mild or moderate depression
F314	Bipolar affective disorder, current episode severe depression without psychotic symptoms
F315	Bipolar affective disorder, current episode severe depression with psychotic symptoms
F316	Bipolar affective disorder, current episode mixed
F317	Bipolar affective disorder, currently in remission
F318	Other bipolar affective disorders
F319	Bipolar affective disorder, unspecified
F32	Depressive episode
F320	Mild depressive episode

CODE	DESCRIPTION
F321	Moderate depressive episode
F322	Severe depressive episode without psychotic symptoms
F323	Severe depressive episode with psychotic symptoms
F328	Other depressive episodes
F329	Depressive episode, unspecified
F33	Recurrent depressive disorder
F330	Recurrent depressive disorder, current episode mild
F331	Recurrent depressive disorder, current episode moderate
F332	Recurrent depressive disorder, current episode severe without psychotic symptoms
F333	Recurrent depressive disorder, current episode severe with psychotic symptoms
F334	Recurrent depressive disorder, currently in remission
F338	Other recurrent depressive disorders
F339	Recurrent depressive disorder, unspecified

(B) GP contacts and prescriptions (Read)

CODE	DESCRIPTION
146D.	H/O: manic depressive disorder
1B12.	Nerves - nervousness
1B13.	Anxiousness
1B17.	Depressed
1B1U.	Symptoms of depression
1BH3.	Paranoid ideation
1BQ..	Loss of capacity for enjoyment
1BT..	Depressed mood
1BU..	Loss of hope for the future
2257	O/E - depressed
2258	O/E - anxious
2259	O/E - nervous
66580	Antidepressant drug treatment changed
66590	Antidepressant drug treatment started
665A0	Antidepressant drug treatment stopped
d1...	HYPNOTICS
d11..	CHLORAL HYDRATE
d111.	*CHLORAL 500mg/5mL mixture
d112.	CHLORAL PAEDIATRIC 200mg/5mL elixir
d113.	*NOCTEC 500mg capsules
d114.	CHLORAL HYDRATE 500mg capsules
d115.	*WELLDORM 143mg/5mL elixir
d116.	*WELLDORM tablets
d117.	CHLORAL HYDRATE 414mg tablets
d118.	CHLORAL HYDRATE 143mg/5mL elixir
d119.	CHLORAL HYDRATE 500mg/5mL syrup
d11A.	SOMNWELL 707mg tablets

CODE	DESCRIPTION
d12..	CLOMETHIAZOLE EDISYLATE [HYPNOTIC]
d121.	*HEMINEVRIN 192mg capsules
d122.	*HEMINEVRIN 250mg/5mL syrup
d123.	*HEMINEVRIN 8mg/mL infusion
d12v.	CLOMETHIAZOLE(base) 192mg capsules
d12w.	CLOMETHIAZOLE EDISILATE 250mg/5mL syrup
d12z.	CHLORMETHIAZOLE EDISYLATE 8mg/mL infusion
d13..	*DICHLORALPHENAZONE
d131.	*WELLDORM 650mg tablets
d132.	*WELLDORM 225mg/5mL elixir
d13y.	DICHLORALPHENAZONE 650mg tablets
d13z.	DICHLORALPHENAZONE 225mg/5mL elixir
d14..	*FLUNITRAZEPAM
d141.	*ROHYPNOL 1mg tablets
d14z.	*FLUNITRAZEPAM 1mg tablets
d15..	FLURAZEPAM
d151.	DALMANE 15mg capsules
d152.	DALMANE 30mg capsules
d153.	*PAXANE 15mg capsules
d154.	*PAXANE 30mg capsules
d15y.	FLURAZEPAM 15mg capsules
d15z.	FLURAZEPAM 30mg capsules
d16..	LOPRAZOLAM
d161.	LOPRAZOLAM 1mg tablets
d162.	*DORMONOCT 1mg tablets
d17..	LORMETAZEPAM
d171.	LORMETAZEPAM 500micrograms tablets
d172.	LORMETAZEPAM 1mg tablets
d173.	NOCTAMID 500micrograms tablets
d174.	*NOCTAMID 1mg tablets
d18..	NITRAZEPAM
d181.	*NITRAZEPAM 5mg capsules
d182.	NITRAZEPAM 5mg tablets
d183.	*NITRAZEPAM 10mg tablets
d184.	NITRAZEPAM 2.5mg/5mL mixture
d185.	*MOGADON 5mg capsules
d186.	MOGADON 5mg tablets
d187.	*NITRADOS 5mg tablets
d188.	*NOCTESED 5mg tablets
d189.	REMNOS 5mg tablets
d18a.	*REMNOS 10mg tablets
d18b.	*SOMNITE 5mg tablets
d18c.	*SOMNITE 2.5mg/5mL mixture
d18d.	*SUREM 5mg capsules
d18e.	*UNISOMNIA 5mg tablets

Cannings-John R, *et al. BMJ Open* 2023; 13:e070637. doi: 10.1136/bmjopen-2022-070637

CODE	DESCRIPTION
d18f.	NITRAZEPAM 5mg/5mL suspension
d19..	PROMETHAZINE HCL [HYPNOTIC] see section c8i..
d1a..	TEMAZEPAM [HYPNOTIC]
d1a1.	*TEMAZEPAM 10mg capsules
d1a2.	*TEMAZEPAM 15mg capsules
d1a3.	*TEMAZEPAM 20mg capsules
d1a4.	*TEMAZEPAM 30mg capsules
d1a5.	*TEMAZEPAM 10mg/5mL elixir
d1a6.	*NORMISON 10mg capsules
d1a7.	*NORMISON 20mg capsules
d1a8.	*TEMAZEPAM PLANPAK capsules
d1a9.	TEMAZEPAM 10mg tablets
d1aa.	TEMAZEPAM 20mg tablets
d1ab.	TEMAZEPAM GELTHIX 10mg capsules
d1ac.	TEMAZEPAM GELTHIX 20mg capsules
d1ad.	TEMAZEPAM GELTHIX 15mg capsules
d1ae.	TEMAZEPAM GELTHIX 30mg capsules
d1af.	TEMAZEPAM GEL FILLED 10mg capsules
d1ag.	TEMAZEPAM GEL FILLED 20mg capsules
d1ah.	TEMAZEPAM GEL FILLED 15mg capsules
d1ai.	TEMAZEPAM GEL FILLED 30mg capsules
d1aj.	TEMAZEPAM 10mg/sachet oral solution
d1ak.	TEMAZEPAM 20mg/sachet oral solution
d1al.	EUHYPNOS 10mg/sachet oral solution
d1am.	EUHYPNOS 20mg/sachet oral solution
d1an.	EUHYPNOS 10mg/5mL oral solution
d1ao.	TEMAZEPAM 10mg/5mL sugar free oral solution
d1b..	*TRIAZOLAM
d1b1.	TRIAZOLAM 125microgram tablets
d1b2.	TRIAZOLAM 250microgram tablets
d1b3.	*HALCION 125microgram tablets
d1b4.	*HALCION 250microgram tablets
d1c..	TRICLOFOS SODIUM
d1c1.	*TRICLOFOS 500mg/5mL liquid
d1d..	ZOPICLONE
d1d1.	ZOPICLONE 7.5mg tablets
d1d2.	ZIMOVANE 7.5mg tablets
d1d3.	ZOPICLONE 3.75mg tablets
d1d4.	ZIMOVANE LS 3.75mg tablets
d1d5.	*ZILEZE 7.5 tablets
d1d6.	*ZILEZE 3.75 tablets
d1e..	*GLUTETHIMIDE [no drugs here]
d1f..	ZOLPIDEM
d1f1.	STILNOCT 5mg tablets
d1f2.	ZOLPIDEM TARTRATE 5mg tablets

CODE	DESCRIPTION
d1f3.	ZOLPIDEM TARTRATE 10mg tablets
d1f4.	STILNOCT 10mg tablets
d1g..	ZALEPLON
d1g1.	SONATA 5mg capsules
d1g2.	SONATA 10mg capsules
d1gy.	ZALEPLON 5mg capsules
d1gz.	ZALEPLON 10mg capsules
d1h..	MELATONIN
d1h1.	CIRCADIN 2mg m/r tablets
d1hz.	MELATONIN 2mg m/r tablets
d21..	DIAZEPAM [ANXIOLYTIC]
d22..	ALPRAZOLAM
d23..	BROMAZEPAM
d24..	CHLORDIAZEPOXIDE
d25..	CHLORMEZANONE
d26..	CLOBAZAM
d27..	CLORAZEPATE DIPOTASSIUM
d28..	HYDROXYZINE HCL [ANXIOLYTIC]
d29..	*KETAZOLAM
d2a..	LORAZEPAM [ANXIOLYTIC]
d2b..	*MEDAZEPAM
d2c..	MEPROBAMATE
d2d..	OXAZEPAM
d2f..	BUSPIRONE HYDROCHLORIDE
d2g..	FLUMAZENIL
d7...	TRICYCLIC ANTIDEPRESSANTS
d71..	AMITRIPTYLINE HYDROCHLORIDE [ANTIDEPRESSANT]
d711.	AMITRIPTYLINE 10mg tablets
d712.	AMITRIPTYLINE 25mg tablets
d713.	AMITRIPTYLINE 50mg tablets
d714.	*DOMICAL 10mg tablets
d715.	*DOMICAL 25mg tablets
d716.	*DOMICAL 50mg tablets
d717.	ELAVIL 10mg tablets
d718.	ELAVIL 25mg tablets
d719.	*LENTIZOL 25mg m/r capsules
d71a.	*LENTIZOL 50mg m/r capsules
d71b.	*TRYPTIZOL 75mg m/r capsules
d71c.	*TRYPTIZOL 10mg tablets
d71d.	*TRYPTIZOL 25mg tablets
d71e.	*TRYPTIZOL 50mg tablets
d71f.	*TRYPTIZOL 10mg/5mL syrup
d71g.	TRYPTIZOL 100mg/10mL injection
d71h.	AMITRIPTYLINE 25mg/5mL sugar free solution
d71i.	AMITRIPTYLINE 50mg/5mL sugar free solution

CODE	DESCRIPTION
d71u.	AMITRIPTYLINE 25mg m/r capsules
d71v.	AMITRIPTYLINE 50mg m/r capsules
d71w.	AMITRIPTYLINE 75mg m/r capsules
d71y.	*AMITRIPTYLINE 10mg/5mL syrup
d71z.	AMITRIPTYLINE HYDROCHLORIDE 100mg/10mL injection
d72..	*BUTRIPTYLINE
d721.	*EVADYNE 25mg tablets
d722.	*EVADYNE 50mg tablets
d72y.	*BUTRIPTYLINE 25mg tablets
d72z.	*BUTRIPTYLINE 50mg tablets
d73..	CLOMIPRAMINE HYDROCHLORIDE
d731.	*ANAFRANIL 10mg capsules
d732.	*ANAFRANIL 25mg capsules
d733.	*ANAFRANIL 50mg capsules
d734.	*ANAFRANIL 25mg/5mL syrup
d735.	*ANAFRANIL 25mg/2mL injection
d736.	*ANAFRANIL SR 75mg m/r tablets
d737.	*TRANQUAX 10mg capsules
d738.	*TRANQUAX 25mg capsules
d739.	TRANQUAX 50mg capsules
d73r.	*CLOMIPRAMINE HCL 50mg tablets
d73s.	*CLOMIPRAMINE HCL 10mg tablets
d73t.	*CLOMIPRAMINE HCL 25mg tablets
d73u.	CLOMIPRAMINE HCL 10mg capsules
d73v.	CLOMIPRAMINE HCL 25mg capsules
d73w.	CLOMIPRAMINE HCL 50mg capsules
d73x.	CLOMIPRAMINE HYDROCHLORIDE 25mg/5mL syrup
d73y.	CLOMIPRAMINE HCL 25mg/2mL injection
d73z.	CLOMIPRAMINE HCL 75mg m/r tabs
d74..	DESIPRAMINE HYDROCHLORIDE
d741.	*PERTOFRAN 25mg tablets
d74z.	DESIPRAMINE HYDROCHLORIDE 25mg tablets
d75..	DOSULEPIN HYDROCHLORIDE
d751.	PROTHIADEN 25mg capsules
d752.	PROTHIADEN 75mg tablets
d753.	*PREPADINE 25mg capsules
d754.	*PREPADINE 75mg tablets
d755.	*DOTHAPAX 25mg capsules
d756.	*DOTHAPAX 75mg tablets
d759.	*THADEN 25mg capsules
d75A.	*THADEN 75mg tablets
d75y.	DOSULEPIN HYDROCHLORIDE 25mg capsules
d75z.	DOSULEPIN HYDROCHLORIDE 75mg tablets
d76..	DOXEPIN
d761.	*SINEQUAN 10mg capsules x56CP

CODE	DESCRIPTION
d762.	*SINEQUAN 25mg capsules x28CP
d763.	*SINEQUAN 50mg capsules x28CP
d764.	*SINEQUAN 75mg capsules x28CP
d765.	*SINEPIN 25mg capsules
d766.	*SINEPIN 50mg capsules
d76w.	*DOXEPIN 10mg capsules
d76x.	DOXEPIN 25mg capsules
d76y.	DOXEPIN 50mg capsules
d76z.	*DOXEPIN 75mg capsules
d77..	IMIPRAMINE HYDROCHLORIDE [ANTIDEPRESSANT]
d771.	IMIPRAMINE 10mg tabs
d772.	IMIPRAMINE 25mg tablets
d773.	*PRAMINIL 10mg tablets
d774.	*PRAMINIL 25mg tablets
d775.	*TOFRANIL 10mg tablets
d776.	*TOFRANIL 25mg tablets
d777.	*TOFRANIL 25mg/5mL syrup
d77w.	IMIPRAMINE HYDROCHLORIDE 25mg/5mL oral solution
d77x.	IMIPRAMINE HYDROCHLORIDE 10mg tablets
d77y.	IMIPRAMINE HYDROCHLORIDE 25mg tablets
d77z.	IMIPRAMINE HYDROCHLORIDE 25mg/5mL syrup
d78..	IPRINDOLE
d781.	*PRONDOL 15mg tablets
d782.	*PRONDOL 30mg tablets
d78y.	*IPRINDOLE 15mg tablets
d78z.	*IPRINDOLE 30mg tablets
d79..	LOFEPRAMINE
d791.	*GAMANIL 70mg tablets x56CP
d792.	LOMONT 70mg/5mL sugar free suspension
d793.	*FEPRAPAX 70mg tablets
d794.	*GAMANIL 70mg tablets
d79y.	LOFEPRAMINE 70mg/5mL sugar free suspension
d79z.	LOFEPRAMINE 70mg tablets
d7a..	MAPROTILINE HYDROCHLORIDE
d7a1.	*LUDIOMIL 10mg tablets
d7a2.	*LUDIOMIL 25mg tablets
d7a3.	*LUDIOMIL 50mg tablets
d7a4.	*LUDIOMIL 75mg tablets x28CP
d7aw.	MAPROTILINE HYDROCHLORIDE 10mg tablets
d7ax.	MAPROTILINE HYDROCHLORIDE 25mg tablets
d7ay.	MAPROTILINE HYDROCHLORIDE 50mg tablets
d7az.	MAPROTILINE HYDROCHLORIDE 75mg tablets
d7b..	MIANSERIN HYDROCHLORIDE
d7b1.	MIANSERIN 10mg tablets
d7b2.	MIANSERIN 20mg tablets

CODE	DESCRIPTION
d7b3.	MIANSERIN 30mg tablets
d7b4.	*BOLVIDON 10mg tablets
d7b5.	*BOLVIDON 20mg tablets
d7b6.	*BOLVIDON 30mg tablets
d7b7.	*NORVAL 10mg tablets
d7b8.	*NORVAL 20mg tablets
d7b9.	*NORVAL 30mg tablets
d7c..	NORTRIPTYLINE
d7c1.	*ALLEGRON 10mg tablets
d7c2.	*ALLEGRON 25mg tablets
d7c3.	*AVENTYL 10mg capsules
d7c4.	*AVENTYL 25mg capsules
d7c5.	*AVENTYL 10mg/5mL liquid
d7c6.	NORTRIPTYLINE 10mg tablets
d7c7.	*NORTRIPTYLINE 10mg/5mL liquid
d7c8.	NORTRIPTYLINE 25mg tablets
d7c9.	*NORTRIPTYLINE 10mg capsules
d7cy.	*NORTRIPTYLINE 25mg capsules
d7d..	PROTRIPTYLINE HYDROCHLORIDE
d7d1.	*CONCORDIN 5mg tablets
d7d2.	*CONCORDIN 10mg tablets
d7d3.	PROTRIPTYLINE HYDROCHLORIDE 5mg tablets
d7d4.	PROTRIPTYLINE HYDROCHLORIDE 10mg tablets
d7e..	TRAZODONE HYDROCHLORIDE
d7e1.	MOLIPAXIN 50mg capsules x84CP
d7e2.	MOLIPAXIN 100mg capsules x56CP
d7e3.	*MOLIPAXIN 50mg/5mL liquid
d7e4.	MOLIPAXIN 150mg tablets x28CP
d7e5.	TRAZODONE HYDROCHLORIDE 150mg tablets
d7e6.	MOLIPAXIN CR 150mg m/r tablets x28
d7e7.	TRAZODONE HYDROCHLORIDE 150mg m/r tablets
d7ew.	TRAZODONE HYDROCHLORIDE 100mg capsules
d7ex.	TRAZODONE HYDROCHLORIDE 50mg capsules
d7ez.	TRAZODONE HYDROCHLORIDE 50mg/5mL liquid
d7f..	TRIMIPRAMINE
d7f1.	*SURMONTIL 50mg capsules x28CP
d7f2.	*SURMONTIL 10mg tablets
d7f3.	*SURMONTIL 25mg tablets
d7fx.	TRIMIPRAMINE 50mg capsules
d7fy.	TRIMIPRAMINE 10mg tablets
d7fz.	TRIMIPRAMINE 25mg tablets
d7g..	VILOXAZINE HYDROCHLORIDE
d7g1.	*VIVALAN 50mg tablets
d7gz.	*VILOXAZINE 50mg tablets
d7h..	AMOXAPINE

CODE	DESCRIPTION
d7h1.	*AMOXAPINE 25mg tablets
d7h2.	*AMOXAPINE 50mg tablets
d7h3.	*AMOXAPINE 100mg tablets
d7h4.	*AMOXAPINE 150mg tablets
d7h5.	*ASENDIS 25mg tablets
d7h6.	*ASENDIS 50mg tablets
d7h7.	*ASENDIS 100mg tablets
d7h8.	*ASENDIS 150mg tablets
d9...	COMPOUND ANTIDEPRESSANT DRUGS
d91..	COMPOUND ANTIDEPRESSANTS A-Z
d911.	*LIMBITROL 5 capsules
d912.	*LIMBITROL 10 capsules
d913.	*MOTIPRESS tablets x28CP
d914.	*MOTIVAL tablets
d915.	*PARSTELIN tablets
d916.	TRIPTAFEN tablets
d917.	*TRIPTAFEN-M tablets
da...	OTHER ANTIDEPRESSANT DRUGS
da1..	FLUPENTIXOL [ANTIDEPRESSANT]
da11.	FLUANXOL 500micrograms tablets
da12.	FLUANXOL 1mg tablets
da1y.	FLUPENTIXOL 500micrograms tablets
da1z.	FLUPENTIXOL 1mg tablets
da2..	TRYPTOPHAN
da21.	*OPTIMAX 500mg tablets
da22.	*OPTIMAX 1g/6g powder
da23.	*OPTIMAX WV 500mg tablets
da24.	*PACITRON 500mg tablets
da2y.	*TRYPTOPHAN 500mg tablets
da2z.	*TRYPTOPHAN 1g/6g powder
da3..	FLUVOXAMINE MALEATE
da31.	FAVERIN 50mg tablets
da32.	FLUVOXAMINE MALEATE 50mg tablets
da33.	FAVERIN 100mg tablets
da34.	FLUVOXAMINE MALEATE 100mg tablets
da4..	FLUOXETINE HYDROCHLORIDE
da41.	FLUOXETINE 20mg capsules
da42.	*PROZAC 20mg capsules x30
da43.	FLUOXETINE 20mg/5mL oral liquid
da44.	PROZAC 20mg/5mL oral liquid
da45.	PROZAC 20mg capsules
da46.	FLUOXETINE 60mg capsules
da47.	*PROZAC 60mg capsules
da48.	*FELICIUM 20mg capsules
da49.	OXACTIN 20mg capsules

CODE	DESCRIPTION
da4A.	RANFLUTIN 20mg capsules
da4B.	PROZIT 20mg/5mL oral solution
da4C.	PROZEP 20mg/5mL oral solution
da5..	SERTRALINE HYDROCHLORIDE
da51.	SERTRALINE 50mg tablets
da52.	SERTRALINE 100mg tablets
da53.	LUSTRAL 50mg tablets
da54.	LUSTRAL 100mg tablets
da6..	PAROXETINE HYDROCHLORIDE
da61.	PAROXETINE 20mg tablets
da62.	SEROXAT 20mg tablets x30
da63.	PAROXETINE 30mg tablets
da64.	SEROXAT 30mg tablets x30
da65.	PAROXETINE 10mg/5mL sugar free liquid
da66.	SEROXAT 10mg/5mL sugar free liquid
da67.	PAROXETINE 10mg tablets
da68.	SEROXAT 10mg tablets
da7..	VENLAFAXINE
da71.	VENLAFAXINE 37.5mg tablets
da72.	VENLAFAXINE 75mg tablets
da73.	*EFEXOR 37.5mg tablets
da74.	*EFEXOR 75mg tablets
da75.	*VENLAFAXINE 50mg tablets
da76.	*EFEXOR 50mg tablets
da77.	VENLAFAXINE 75mg m/r capsules
da78.	EFEXOR XL 75mg m/r capsules
da79.	VENLAFAXINE 150mg m/r capsules
da7a.	VENAXX XL 150mg m/r capsules
da7A.	EFEXOR XL 150mg m/r capsules
da7b.	VAXALIN XL 75mg m/r capsules
da7B.	RODOMEL XL 75mg m/r capsules
da7c.	VAXALIN XL 150mg m/r capsules
da7C.	RODOMEL XL 150mg m/r capsules
da7d.	ALVENTA XL 75mg m/r capsules
da7D.	WINFEX XL 75mg m/r capsules
da7e.	ALVENTA XL 150mg m/r capsules
da7E.	WINFEX XL 150mg m/r capsules
da7f.	RANFAXINE XL 150mg m/r capsules
da7F.	TRIXAT XL 75mg m/r capsules
da7g.	RANFAXINE XL 75mg m/r capsules
da7G.	TRIXAT XL 150mg m/r capsules
da7h.	BONILUX XL 75mg m/r capsules
da7H.	VIEPAX XL 75mg m/r tablets
da7i.	BONILUX XL 150mg m/r capsules
da7I.	VENLAFAXINE 75mg m/r tablets

Cannings-John R, *et al. BMJ Open* 2023; 13:e070637. doi: 10.1136/bmjopen-2022-070637

CODE	DESCRIPTION
da7j.	TONPULAR XL 75mg m/r capsules
da7J.	VIEPAX XL 150mg m/r tablets
da7k.	TONPULAR XL 150mg m/r capsules
da7K.	VENLAFAXINE 150mg m/r tablets
da7l.	FORAVEN XL 75mg m/r capsules
da7L.	*TARDCAPS XL 75mg m/r capsules
da7m.	FORAVEN XL 150mg m/r capsules
da7M.	*TARDCAPS XL 150mg m/r capsule
da7n.	DEPEFEX XL 75mg m/r capsules
da7N.	VIEPAX 37.5mg tablets
da7o.	DEPEFEX XL 150mg m/r capsules
da7O.	VIEPAX 75mg tablets
da7p.	VENLALIC XL 37.5mg m/r tablets
da7P.	VENSIR XL 75mg m/r capsules
da7q.	VENLAFAXINE 37.5mg m/r tablets
da7Q.	VENSIR XL 150mg m/r capsules
da7R.	TIFAXIN XL 75mg m/r capsules
da7S.	TIFAXIN XL 150mg m/r capsules
da7T.	VEXARIN XL 75mg m/r capsules
da7U.	VEXARIN XL 150mg m/r capsules
da7V.	VENLALIC XL 75mg m/r tablets
da7W.	VENLALIC XL 150mg m/r tablets
da7X.	VENLALIC XL 225mg m/r tablets
da7Y.	VENLAFAXINE 225mg m/r tablets
da7Z.	VENAXX XL 75mg m/r capsules
da8..	NEFAZODONE
da81.	NEFAZODONE HYDROCHLORIDE 100mg tablets
da82.	NEFAZODONE HYDROCHLORIDE 200mg tablets
da83.	*DUTONIN 100mg tablets
da84.	*DUTONIN 200mg tablets
da85.	NEFAZODONE HCL 50mg+100mg+200mg initiation tablets pack
da86.	DUTONIN 50mg+100mg+200mg treatment initiation tablets pack
da9..	CITALOPRAM
da91.	CITALOPRAM 20mg tablets
da92.	CIPRAMIL 20mg tablets
da93.	CITALOPRAM 10mg tablets
da94.	*CIPRAMIL 10mg tablets
da95.	CITALOPRAM 40mg tablets
da96.	*CIPRAMIL 40mg tablets
da97.	CIPRAMIL 40mg/mL oral drops 15mL
da98.	*PAXORAN 10mg tablets
da99.	*PAXORAN 20mg tablets
da9A.	*PAXORAN 40mg tablets
da9z.	CITALOPRAM 40mg/mL oral drops
daA..	REBOXETINE

Cannings-John R, *et al. BMJ Open* 2023; 13:e070637. doi: 10.1136/bmjopen-2022-070637

CODE	DESCRIPTION
daA1.	REBOXETINE 4mg tablets
daA2.	EDRONAX 4mg tablets
daB..	MIRTAZAPINE
daB1.	MIRTAZAPINE 30mg tablets
daB2.	*ZISPIN 30mg tablets
daB3.	MIRTAZAPINE 30mg oro-dispersible tablets
daB4.	ZISPIN SOLTAB 30mg oro-dispersible tablets
daB5.	MIRTAZAPINE 15mg oro-dispersible tablets
daB6.	ZISPIN SOLTAB 15mg oro-dispersible tablets
daB7.	MIRTAZAPINE 45mg oro-dispersible tablets
daB8.	ZISPIN SOLTAB 45mg oro-dispersible tablets
daBy.	MIRTAZAPINE 45mg tablets
daBz.	MIRTAZAPINE 15mg tablets
daC..	ESCITALOPRAM
daC1.	ESCITALOPRAM 10mg tablets
daC2.	CIPRALEX 10mg tablets
daC3.	ESCITALOPRAM 20mg tablets
daC4.	CIPRALEX 20mg tablets
daC5.	ESCITALOPRAM 5mg tablets
daC6.	CIPRALEX 5mg tablets
daC7.	ESCITALOPRAM 10mg/mL oral drops
daC8.	*CIPRALEX 10mg/mL oral drops
daC9.	CIPRALEX 20mg/mL oral drops
daCA.	ESCITALOPRAM 20mg/mL oral drops
daD..	AGOMELATINE
daD1.	VALDOXAN 25mg tablets
daD2.	AGOMELATINE 25mg tablets
E1...	Non-organic psychoses
E10..	Schizophrenic disorders
E100.	Simple schizophrenia
E1000	Unspecified schizophrenia
E1001	Subchronic schizophrenia
E1002	Chronic schizophrenic
E1003	Acute exacerbation of subchronic schizophrenia
E1004	Acute exacerbation of chronic schizophrenia
E1005	Schizophrenia in remission
E100z	Simple schizophrenia NOS
E101.	Hebephrenic schizophrenia
E1010	Unspecified hebephrenic schizophrenia
E1011	Subchronic hebephrenic schizophrenia
E1012	Chronic hebephrenic schizophrenia
E1013	Acute exacerbation of subchronic hebephrenic schizophrenia
E1014	Acute exacerbation of chronic hebephrenic schizophrenia
E1015	Hebephrenic schizophrenia in remission
E101z	Hebephrenic schizophrenia NOS

Cannings-John R, *et al. BMJ Open* 2023; 13:e070637. doi: 10.1136/bmjopen-2022-070637

CODE	DESCRIPTION
E102.	Catatonic schizophrenia
E1020	Unspecified catatonic schizophrenia
E1021	Subchronic catatonic schizophrenia
E1022	Chronic catatonic schizophrenia
E1023	Acute exacerbation of subchronic catatonic schizophrenia
E1024	Acute exacerbation of chronic catatonic schizophrenia
E1025	Catatonic schizophrenia in remission
E102z	Catatonic schizophrenia NOS
E103.	Paranoid schizophrenia
E1030	Unspecified paranoid schizophrenia
E1031	Subchronic paranoid schizophrenia
E1032	Chronic paranoid schizophrenia
E1033	Acute exacerbation of subchronic paranoid schizophrenia
E1034	Acute exacerbation of chronic paranoid schizophrenia
E1035	Paranoid schizophrenia in remission
E103z	Paranoid schizophrenia NOS
E104.	Acute schizophrenic episode
E105.	Latent schizophrenia
E1050	Unspecified latent schizophrenia
E1051	Subchronic latent schizophrenia
E1052	Chronic latent schizophrenia
E1053	Acute exacerbation of subchronic latent schizophrenia
E1054	Acute exacerbation of chronic latent schizophrenia
E1055	Latent schizophrenia in remission
E105z	Latent schizophrenia NOS
E106.	Residual schizophrenia
E107.	Schizo-affective schizophrenia
E1070	Unspecified schizo-affective schizophrenia
E1071	Subchronic schizo-affective schizophrenia
E1072	Chronic schizo-affective schizophrenia
E1073	Acute exacerbation subchronic schizo-affective schizophrenia
E1074	Acute exacerbation of chronic schizo-affective schizophrenia
E1075	Schizo-affective schizophrenia in remission
E107z	Schizo-affective schizophrenia NOS
E10y.	Other schizophrenia
E10y0	Atypical schizophrenia
E10y1	Coenesthopathic schizophrenia
E10yz	Other schizophrenia NOS
E10z.	Schizophrenia NOS
E11..	Affective psychoses
E110.	Manic disorder, single episode
E1100	Single manic episode, unspecified
E1101	Single manic episode, mild
E1102	Single manic episode, moderate
E1103	Single manic episode, severe without mention of psychosis

Cannings-John R, *et al. BMJ Open* 2023; 13:e070637. doi: 10.1136/bmjopen-2022-070637

CODE	DESCRIPTION
E1104	Single manic episode, severe, with psychosis
E1105	Single manic episode in partial or unspecified remission
E1106	Single manic episode in full remission
E110z	Manic disorder, single episode NOS
E111.	Recurrent manic episodes
E1110	Recurrent manic episodes, unspecified
E1111	Recurrent manic episodes, mild
E1112	Recurrent manic episodes, moderate
E1113	Recurrent manic episodes, severe without mention psychosis
E1114	Recurrent manic episodes, severe, with psychosis
E1115	Recurrent manic episodes, partial or unspecified remission
E1116	Recurrent manic episodes, in full remission
E111z	Recurrent manic episode NOS
E112.	Single major depressive episode
E1120	Single major depressive episode, unspecified
E1121	Single major depressive episode, mild
E1122	Single major depressive episode, moderate
E1123	Single major depressive episode, severe, without psychosis
E1124	Single major depressive episode, severe, with psychosis
E1125	Single major depressive episode, partial or unspec remission
E1126	Single major depressive episode, in full remission
E112z	Single major depressive episode NOS
E113.	Recurrent major depressive episode
E1130	Recurrent major depressive episodes, unspecified
E1131	Recurrent major depressive episodes, mild
E1132	Recurrent major depressive episodes, moderate
E1133	Recurrent major depressive episodes, severe, no psychosis
E1134	Recurrent major depressive episodes, severe, with psychosis
E1135	Recurrent major depressive episodes,partial/unspec remission
E1136	Recurrent major depressive episodes, in full remission
E1137	Recurrent depression
E113z	Recurrent major depressive episode NOS
E114.	Bipolar affective disorder, currently manic
E1140	Bipolar affective disorder, currently manic, unspecified
E1141	Bipolar affective disorder, currently manic, mild
E1142	Bipolar affective disorder, currently manic, moderate
E1143	Bipolar affect disord, currently manic, severe, no psychosis
E1144	Bipolar affect disord, currently manic,severe with psychosis
E1145	Bipolar affect disord,currently manic, part/unspec remission
E1146	Bipolar affective disorder, currently manic, full remission
E114z	Bipolar affective disorder, currently manic, NOS
E115.	Bipolar affective disorder, currently depressed
E1150	Bipolar affective disorder, currently depressed, unspecified
E1151	Bipolar affective disorder, currently depressed, mild
E1152	Bipolar affective disorder, currently depressed, moderate

Cannings-John R, *et al. BMJ Open* 2023; 13:e070637. doi: 10.1136/bmjopen-2022-070637

CODE	DESCRIPTION
E1153	Bipolar affect disord, now depressed, severe, no psychosis
E1154	Bipolar affect disord, now depressed, severe with psychosis
E1155	Bipolar affect disord, now depressed, part/unspec remission
E1156	Bipolar affective disorder, now depressed, in full remission
E115z	Bipolar affective disorder, currently depressed, NOS
E116.	Mixed bipolar affective disorder
E1160	Mixed bipolar affective disorder, unspecified
E1161	Mixed bipolar affective disorder, mild
E1162	Mixed bipolar affective disorder, moderate
E1163	Mixed bipolar affective disorder, severe, without psychosis
E1164	Mixed bipolar affective disorder, severe, with psychosis
E1165	Mixed bipolar affective disorder, partial/unspec remission
E1166	Mixed bipolar affective disorder, in full remission
E116z	Mixed bipolar affective disorder, NOS
E117.	Unspecified bipolar affective disorder
E1170	Unspecified bipolar affective disorder, unspecified
E1171	Unspecified bipolar affective disorder, mild
E1172	Unspecified bipolar affective disorder, moderate
E1173	Unspecified bipolar affective disorder, severe, no psychosis
E1174	Unspecified bipolar affective disorder,severe with psychosis
E1175	Unspecified bipolar affect disord, partial/unspec remission
E1176	Unspecified bipolar affective disorder, in full remission
E117z	Unspecified bipolar affective disorder, NOS
E118.	Seasonal affective disorder
E11y.	Other and unspecified manic-depressive psychoses
E11y0	Unspecified manic-depressive psychoses
E11y1	Atypical manic disorder
E11y2	Atypical depressive disorder
E11y3	Other mixed manic-depressive psychoses
E11yz	Other and unspecified manic-depressive psychoses NOS
E11z.	Other and unspecified affective psychoses
E11z0	Unspecified affective psychoses NOS
E11zz	Other affective psychosis NOS
E12..	Paranoid states
E120.	Simple paranoid state
E121.	Chronic paranoid psychosis
E122.	Paraphrenia
E123.	Shared paranoid disorder
E12y.	Other paranoid states
E12y0	Paranoia querulans
E12yz	Other paranoid states NOS
E12z.	Paranoid psychosis NOS
E13..	Other nonorganic psychoses
E130.	Reactive depressive psychosis
E131.	Acute hysterical psychosis

Cannings-John R, *et al. BMJ Open* 2023; 13:e070637. doi: 10.1136/bmjopen-2022-070637

CODE	DESCRIPTION
E132.	Reactive confusion
E133.	Acute paranoid reaction
E134.	Psychogenic paranoid psychosis
E135.	Agitated depression
E13y.	Other reactive psychoses
E13y0	Psychogenic stupor
E13y1	Brief reactive psychosis
E13yz	Other reactive psychoses NOS
E13z.	Nonorganic psychosis NOS
E1y..	Other specified non-organic psychoses
E1z..	Non-organic psychosis NOS
E200.	Anxiety states
E2000	Anxiety state unspecified
E2001	Panic disorder
E2002	Generalised anxiety disorder
E2003	Anxiety with depression
E2004	Chronic anxiety
E2005	Recurrent anxiety
E200z	Anxiety state NOS
E204.	Neurotic depression reactive type
E212.	Schizoid personality disorder
E2120	Unspecified schizoid personality disorder
E2121	Introverted personality
E2122	Schizotypal personality
E212z	Schizoid personality disorder NOS
E258.	Nondependent antidepressant type drug abuse
E2580	Nondependent antidepressant type drug abuse, unspecified
E2581	Nondependent antidepressant type drug abuse, continuous
E2582	Nondependent antidepressant type drug abuse, episodic
E2583	Nondependent antidepressant type drug abuse in remission
E258z	Nondependent antidepressant type drug abuse NOS
E291.	Prolonged depressive reaction
E2B..	Depressive disorder NEC
E2B0.	Postviral depression
E2B1.	Chronic depression
Eu...	[X]Mental and behavioural disorders
Eu2..	[X]Schizophrenia, schizotypal and delusional disorders
Eu20.	[X]Schizophrenia
Eu200	[X]Paranoid schizophrenia
Eu201	[X]Hebephrenic schizophrenia
Eu202	[X]Catatonic schizophrenia
Eu203	[X]Undifferentiated schizophrenia
Eu204	[X]Post-schizophrenic depression
Eu205	[X]Residual schizophrenia
Eu206	[X]Simple schizophrenia

CODE	DESCRIPTION
Eu20y	[X]Other schizophrenia
Eu20z	[X]Schizophrenia, unspecified
Eu21.	[X]Schizotypal disorder
Eu22.	[X]Persistent delusional disorders
Eu220	[X]Delusional disorder
Eu221	[X]Delusional misidentification syndrome
Eu222	[X]Cotard syndrome
Eu223	[X]Paranoid state in remission
Eu22y	[X]Other persistent delusional disorders
Eu22z	[X]Persistent delusional disorder, unspecified
Eu23.	[X]Acute and transient psychotic disorders
Eu230	[X]Acute polymorphic psychot disord without symp of schizoph
Eu231	[X]Acute polymorphic psychot disord with symp of schizophren
Eu232	[X]Acute schizophrenia-like psychotic disorder
Eu233	[X]Other acute predominantly delusional psychotic disorders
Eu23y	[X]Other acute and transient psychotic disorders
Eu23z	[X]Acute and transient psychotic disorder, unspecified
Eu24.	[X]Induced delusional disorder
Eu25.	[X]Schizoaffective disorders
Eu250	[X]Schizoaffective disorder, manic type
Eu251	[X]Schizoaffective disorder, depressive type
Eu252	[X]Schizoaffective disorder, mixed type
Eu25y	[X]Other schizoaffective disorders
Eu25z	[X]Schizoaffective disorder, unspecified
Eu26.	[X]Nonorganic psychosis in remission
Eu2y.	[X]Other nonorganic psychotic disorders
Eu2z.	[X]Unspecified nonorganic psychosis
Eu3..	[X]Mood - affective disorders
Eu30.	[X]Manic episode
Eu300	[X]Hypomania
Eu301	[X]Mania without psychotic symptoms
Eu302	[X]Mania with psychotic symptoms
Eu30y	[X]Other manic episodes
Eu30z	[X]Manic episode, unspecified
Eu31.	[X]Bipolar affective disorder
Eu310	[X]Bipolar affective disorder, current episode hypomanic
Eu311	[X]Bipolar affect disorder cur epi manic wout psychotic symp
Eu312	[X]Bipolar affect disorder cur epi manic with psychotic symp
Eu313	[X]Bipolar affect disorder cur epi mild or moderate depressn
Eu314	[X]Bipol aff disord, curr epis sev depress, no psychot symp
Eu315	[X]Bipolar affect dis cur epi severe depres with psyc symp
Eu316	[X]Bipolar affective disorder, current episode mixed
Eu317	[X]Bipolar affective disorder, currently in remission
Eu318	[X]Bipolar affective disorder type I
Eu319	[X]Bipolar affective disorder type II

CODE	DESCRIPTION
Eu31y	[X]Other bipolar affective disorders
Eu31z	[X]Bipolar affective disorder, unspecified
Eu32.	[X]Depressive episode
Eu320	[X]Mild depressive episode
Eu321	[X]Moderate depressive episode
Eu322	[X]Severe depressive episode without psychotic symptoms
Eu323	[X]Severe depressive episode with psychotic symptoms
Eu324	[X]Mild depression
Eu328	[X]Major depression, severe with psychotic symptoms
Eu329	[X]Single major depr ep, severe with psych, psych in remiss
Eu32A	[X]Recurr major depr ep, severe with psych, psych in remiss
Eu32y	[X]Other depressive episodes
Eu32z	[X]Depressive episode, unspecified
Eu33.	[X]Recurrent depressive disorder
Eu330	[X]Recurrent depressive disorder, current episode mild
Eu331	[X]Recurrent depressive disorder, current episode moderate
Eu332	[X]Recurr depress disorder cur epi severe without psyc sympt
Eu333	[X]Recurrent depress disorder cur epi severe with psyc symp
Eu334	[X]Recurrent depressive disorder, currently in remission
Eu33y	[X]Other recurrent depressive disorders
Eu33z	[X]Recurrent depressive disorder, unspecified
Eu34.	[X]Persistent mood affective disorders
Eu341	[X]Dysthymia
Eu34y	[X]Other persistent mood affective disorders
Eu34z	[X]Persistent mood affective disorder, unspecified
Eu3y.	[X]Other mood affective disorders
Eu3y0	[X]Other single mood affective disorders
Eu3y1	[X]Other recurrent mood affective disorders
Eu3yy	[X]Other specified mood affective disorders
Eu3z.	[X]Unspecified mood affective disorder
Eu41.	[X]Other anxiety disorders
Eu410	[X]Panic disorder [episodic paroxysmal anxiety]
Eu411	[X]Generalized anxiety disorder
Eu412	[X]Mixed anxiety and depressive disorder
Eu413	[X]Other mixed anxiety disorders
Eu41y	[X]Other specified anxiety disorders
Eu41z	[X]Anxiety disorder, unspecified
Eu601	[X]Schizoid personality disorder
R2y2.	[D]Nervousness
SL90.	Antidepressant poisoning
SL90z	Anti-depressant poisoning NOS
SyuFK	[X]Poisoning by other and unspecified antidepressants
T840.	Accidental poisoning by antidepressants
T840z	Accidental poisoning by antidepressants NOS
TJ90.	Adverse reaction to antidepressants

CODE	DESCRIPTION
TJ90z	Adverse reaction to antidepressants NOS
U6090	[X]Tricyc/tetracyc antidepres caus advers eff therapeut use
U6091	[X]Monoamine oxid inhib antidep caus advers eff therap use
U6092	[X]Other unspec antidepres caus advers effect in therap use
ZV110	[V]Personal history of schizophrenia
ZV111	[V]Personal history of affective disorder

Fit notes (Read)

CODE	DESCRIPTION
13JJ1	Benefit agncy reprt unfit for work but fit note no lngr need
9D1..	MED3 - doctor's statement
9D11.	MED3 issued to patient
9D12.	MED3 duplicate issued
9D14.	MED3 issued - back to work
9D15.	eMED3 (2010) new statement issued, not fit for work
9D19.	MED3 (2010) issued by hand, not fit for work
9D1C.	MED3 (2010) issued to patient
9D1Z.	MED3 - NOS
9D5..	Private sickness certificate
9DC..	SC1 - self certificate admin.
9DC1.	SC1 self-certificate advised
9DC2.	SC1 self certificate given
9DCZ.	SC1 self-certification NOS
9DL..	SC2 - self-certificate administration
9DL0.	SC2 self-certificate advised
9DL1.	SC2 self certificate given
9K8..	Sickness certificate
ZV680	[V]Issue of medical certificate

Hospital admissions for Non-COVID-19 respiratory infections (ICD-10)

CODE	DESCRIPTION
A37	Whooping cough
A370	Whooping cough due to Bordetella pertussis
A371	Whooping cough due to Bordetella parapertussis
A378	Whooping cough due to other Bordetella species
A379	Whooping cough, unspecified
B012	Varicella pneumonia
B052	Measles complicated by pneumonia
B250	Cytomegaloviral pneumonitis
J10	Influenza due to identified seasonal influenza virus
J100	Influenza with pneumonia, seasonal influenza virus identified
J101	Influenza with other respiratory manifestations, seasonal influenza virus identified

CODE	DESCRIPTION
J108	Influenza with other manifestations, seasonal influenza virus identified
J11	Influenza, virus not identified
J110	Influenza with pneumonia, virus not identified
J111	Influenza with other respiratory manifestations, virus not identified
J118	Influenza with other manifestations, virus not identified
J12	Viral pneumonia, not elsewhere classified
J120	Adenoviral pneumonia
J121	Respiratory syncytial virus pneumonia
J122	Parainfluenza virus pneumonia
J123	Human metapneumovirus pneumonia
J128	Other viral pneumonia
J129	Viral pneumonia, unspecified
J15	Bacterial pneumonia, not elsewhere classified
J150	Pneumonia due to Klebsiella pneumoniae
J151	Pneumonia due to Pseudomonas
J152	Pneumonia due to staphylococcus
J153	Pneumonia due to streptococcus, group B
J154	Pneumonia due to other streptococci
J155	Pneumonia due to Escherichia coli
J156	Pneumonia due to other Gram-negative bacteria
J157	Pneumonia due to Mycoplasma pneumoniae
J158	Other bacterial pneumonia
J159	Bacterial pneumonia, unspecified
J16	Pneumonia due to other infectious organisms, not elsewhere classified
J168	Pneumonia due to other specified infectious organisms
J17	Pneumonia in diseases classified elsewhere
J170	Pneumonia in bacterial diseases classified elsewhere
J171	Pneumonia in viral diseases classified elsewhere
J172	Pneumonia in mycoses
J173	Pneumonia in parasitic diseases
J178	Pneumonia in other diseases classified elsewhere
J18	Pneumonia, organism unspecified
J180	Bronchopneumonia, unspecified
J181	Lobar pneumonia, unspecified
J182	Hypostatic pneumonia, unspecified
J188	Other pneumonia, organism unspecified
J189	Pneumonia, unspecified
J20	Acute bronchitis
J200	Acute bronchitis due to Mycoplasma pneumoniae
J201	Acute bronchitis due to Haemophilus influenzae
J202	Acute bronchitis due to streptococcus
J203	Acute bronchitis due to coxsackievirus
J204	Acute bronchitis due to parainfluenza virus
J205	Acute bronchitis due to respiratory syncytial virus
J206	Acute bronchitis due to rhinovirus

Cannings-John R, *et al. BMJ Open* 2023; 13:e070637. doi: 10.1136/bmjopen-2022-070637

CODE	DESCRIPTION
J207	Acute bronchitis due to echovirus
J208	Acute bronchitis due to other specified organisms
J209	Acute bronchitis, unspecified
J21	Acute bronchiolitis
J210	Acute bronchiolitis due to respiratory syncytial virus
J211	Acute bronchiolitis due to human metapneumovirus
J218	Acute bronchiolitis due to other specified organisms
J219	Acute bronchiolitis, unspecified
J41	Simple and mucopurulent chronic bronchitis
J410	Simple chronic bronchitis
J411	Mucopurulent chronic bronchitis
J418	Mixed simple and mucopurulent chronic bronchitis
J43	Emphysema
J430	MacLeod syndrome
J431	Panlobular emphysema
J432	Centrilobular emphysema
J438	Other emphysema
J439	Emphysema, unspecified
J440	Chronic obstructive pulmonary disease with acute lower respiratory infection
J441	Chronic obstructive pulmonary disease with acute exacerbation, unspecified
J448	Other specified chronic obstructive pulmonary disease
J449	Chronic obstructive pulmonary disease, unspecified
J45	Asthma
J450	Predominantly allergic asthma
J451	Nonallergic asthma
J458	Mixed asthma
J459	Asthma, unspecified
J851	Abscess of lung with pneumonia
J86	Pyothorax
J860	Pyothorax with fistula
J869	Pyothorax without fistula
J982	Interstitial emphysema
J983	Compensatory emphysema
U04	Severe acute respiratory syndrome [SARS]

Supplementary material 3: The RECORD statement – checklist of items, extended from the STROBE statement, that should be reported in observational studies using routinely collected health data.

	Item No.	STROBE items	Section/Location in manuscript where items are reported	RECORD items	Section/Location in manuscript where items are reported
**Title and abstract**					
	1	(a) Indicate the study's design with a commonly used term in the title or the abstract<br><br>(b) Provide in the abstract an informative and balanced summary of what was done and what was found	Title<br><br>Title	RECORD 1.1: The type of data used should be specified in the title or abstract. When possible, the name of the databases used should be included.<br><br>RECORD 1.2: If applicable, the geographic region and timeframe within which the study took place should be reported in the title or abstract.<br><br>RECORD 1.3: If linkage between databases was conducted for the study, this should be clearly stated in the title or abstract.	Title<br><br>Title<br><br>NA
**Introduction**					
Background rationale	2	Explain the scientific background and rationale for the investigation being reported	Introduction		
Objectives	3	State specific objectives, including any prespecified hypotheses	Introduction		
**Methods**					
Study Design	4	Present key elements of study design early in the paper	Study design and data sources		
Setting	5	Describe the setting, locations, and relevant dates, including periods of recruitment, exposure, follow-up, and data collection	Materials and methods		
Participants	6	*(a) Cohort study* - Give the eligibility criteria, and the sources and methods of selection of participants. Describe methods of follow-up<br>*Case-control study* - Give the eligibility criteria, and the sources and methods of case	Population	RECORD 6.1: The methods of study population selection (such as codes or algorithms used to identify subjects) should be listed in detail. If this is not possible, an explanation should be provided.	NA

Cannings-John R, *et al. BMJ Open* 2023; 13:e070637. doi: 10.1136/bmjopen-2022-070637

	Item No.	STROBE items	Section/Location in manuscript where items are reported	RECORD items	Section/Location in manuscript where items are reported
		ascertainment and control selection. Give the rationale for the choice of cases and controls<br>*Cross-sectional study* - Give the eligibility criteria, and the sources and methods of selection of participants<br><br>*(b) Cohort study* - For matched studies, give matching criteria and number of exposed and unexposed<br>*Case-control study* - For matched studies, give matching criteria and the number of controls per case		RECORD 6.2: Any validation studies of the codes or algorithms used to select the population should be referenced. If validation was conducted for this study and not published elsewhere, detailed methods and results should be provided.<br><br>RECORD 6.3: If the study involved linkage of databases, consider use of a flow diagram or other graphical display to demonstrate the data linkage process, including the number of individuals with linked data at each stage.	NA<br><br>NA
Variables	7	Clearly define all outcomes, exposures, predictors, potential confounders, and effect modifiers. Give diagnostic criteria, if applicable.	Primary and secondary outcomes	RECORD 7.1: A complete list of codes and algorithms used to classify exposures, outcomes, confounders, and effect modifiers should be provided. If these cannot be reported, an explanation should be provided.	Supplementary material
Data sources/ measurement	8	For each variable of interest, give sources of data and details of methods of assessment (measurement).<br>Describe comparability of assessment methods if there is more than one group	Primary and secondary outcomes and supplementary material 1		
Bias	9	Describe any efforts to address potential sources of bias	Discussion – strengths and weaknesses		
Study size	10	Explain how the study size was arrived at	Protocol paper		
Quantitative variables	11	Explain how quantitative variables were handled in the analyses. If applicable, describe which groupings were chosen, and why	Analysis		
Statistical methods	12	(a) Describe all statistical methods, including those used to control for confounding	Analysis		

Cannings-John R, *et al. BMJ Open* 2023; 13:e070637. doi: 10.1136/bmjopen-2022-070637

	Item No.	STROBE items	Section/Location in manuscript where items are reported	RECORD items	Section/Location in manuscript where items are reported
		(b) Describe any methods used to examine subgroups and interactions<br>(c) Explain how missing data were addressed<br>(d) *Cohort study* - If applicable, explain how loss to follow-up was addressed<br>*Case-control study* - If applicable, explain how matching of cases and controls was addressed<br>*Cross-sectional study* - If applicable, describe analytical methods taking account of sampling strategy<br>(e) Describe any sensitivity analyses			
Data access and cleaning methods		..		RECORD 12.1: Authors should describe the extent to which the investigators had access to the database population used to create the study population.<br><br>RECORD 12.2: Authors should provide information on the data cleaning methods used in the study.	Analysis<br><br>Analysis
Linkage		..		RECORD 12.3: State whether the study included person-level, institutional-level, or other data linkage across two or more databases. The methods of linkage and methods of linkage quality evaluation should be provided.	Study design and data sources
**Results**					
Participants	13	(a) Report the numbers of individuals at each stage of the study (*e.g.*, numbers potentially eligible, examined for eligibility, confirmed eligible, included in the study, completing follow-up, and analysed)<br>(b) Give reasons for non-participation at each stage.<br>(c) Consider use of a flow diagram	Results: Study population	RECORD 13.1: Describe in detail the selection of the persons included in the study (*i.e.,* study population selection) including filtering based on data quality, data availability and linkage. The selection of included persons can be described in the text and/or by means of the study flow diagram.	Results: Study population

	Item No.	STROBE items	Section/Location in manuscript where items are reported	RECORD items	Section/Location in manuscript where items are reported
Descriptive data	14	(a) Give characteristics of study participants (*e.g.*, demographic, clinical, social) and information on exposures and potential confounders<br>(b) Indicate the number of participants with missing data for each variable of interest<br>(c) *Cohort study* - summarise follow-up time (*e.g.*, average and total amount)	Results: Study population, table 1		
Outcome data	15	*Cohort study* - Report numbers of outcome events or summary measures over time<br>*Case-control study* - Report numbers in each exposure category, or summary measures of exposure<br>*Cross-sectional study* - Report numbers of outcome events or summary measures	Results, table 2		
Main results	16	(a) Give unadjusted estimates and, if applicable, confounder-adjusted estimates and their precision (e.g., 95% confidence interval). Make clear which confounders were adjusted for and why they were included<br>(b) Report category boundaries when continuous variables were categorized<br>(c) If relevant, consider translating estimates of relative risk into absolute risk for a meaningful time period	Results, table 1 and 2, supplementary material 4-5		
Other analyses	17	Report other analyses done—e.g., analyses of subgroups and interactions, and sensitivity analyses	Results, supplementary material 4-5		
**Discussion**					
Key results	18	Summarise key results with reference to study objectives	Discussion-Principal findings		

	Item No.	STROBE items	Section/Location in manuscript where items are reported	RECORD items	Section/Location in manuscript where items are reported
Limitations	19	Discuss limitations of the study, taking into account sources of potential bias or imprecision. Discuss both direction and magnitude of any potential bias	Discussion- Strengths and weaknesses	RECORD 19.1: Discuss the implications of using data that were not created or collected to answer the specific research question(s). Include discussion of misclassification bias, unmeasured confounding, missing data, and changing eligibility over time, as they pertain to the study being reported.	Page 16
Interpretation	20	Give a cautious overall interpretation of results considering objectives, limitations, multiplicity of analyses, results from similar studies, and other relevant evidence	Discussion- strengths and weaknesses		
Generalisability	21	Discuss the generalisability (external validity) of the study results	Discussion - findings in context		
**Other Information**					
Funding	22	Give the source of funding and the role of the funders for the present study and, if applicable, for the original study on which the present article is based	Funding		
Accessibility of protocol, raw data, and programming code		..		RECORD 22.1: Authors should provide information on how to access any supplemental information such as the study protocol, raw data, or programming code.	Data sharing statement, protocol, Supplementary material 2

Supplementary material 4: Hospital admissions for respiratory infections not necessarily recorded as COVID-19 for domiciliary care workers from March 2016 to October 2021.

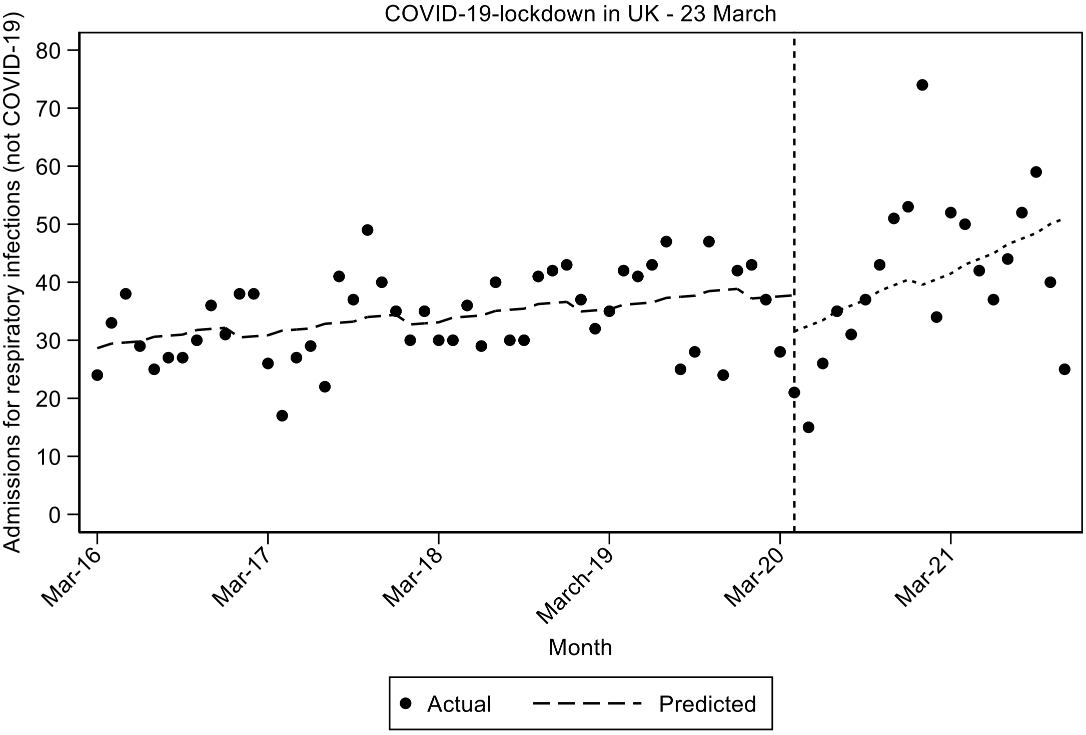

Supplementary material 5: Characteristics of domiciliary care workers receiving at least one fit note in the COVID-19 pandemic between 1st March 2020 and 31st November 2021.

Characteristics	N	%
**Source: SCW[a]**		
Sex		
Female	2,004	15.1
Male	293	11.9
Age (at registration)		
Under 35 years	572	12.9
35 to <45 years	380	13.5
45 to <55 years	625	15.8
55 years and over	720	15.9
Ethnicity		
White	2,220	14.8
Black	11	7.8
Mixed	*	*
Asian	14	13.0

**Characteristics**	**N**	**%**
Other	*	*
Mixed/Other	18	9.3
Not recorded	34	12.6
** numbers suppressed as <10. Mixed and Other categories are combined.*		
Lives with DCW		
No	2,168	14.7
Yes	129	13.2
Have disability		
No	2,089	14.8
Yes	23	12.2
Missing/Prefer not to answer	185	13.3
Qualifications as recorded at registration		
Competence confirmed	255	12.9
Induction Framework	460	15.4
Qualification Level 2	1,054	15.2
Qualification Level 3	470	14.1
Qualification Level 4+	48	10.9
Not recorded	10	23.3
Employment sector as recorded at registration		
Private	828	12.6
Third Sector	562	13.2
Local Authority - Social Services	678	20.5
Recruitment Agency/ Employment Agency	23	13.4
Health/Other		
Not recorded	206	14.8
Smoking status		
Non-smoker	917	14.5
Ex-smoker	601	12.0
Current smoker	779	17.8
Body Mass Index		
Underweight (<18.5)	37	14.5
Healthy weight (18.5-24.9)	466	14.7
Overweight (25-29.9)	576	15.8
Obese (≥30)	993	18.7
Not recorded	225	6.8
Any comorbidity[d]		
No	1,027	11.5
Yes	1,270	18.7
On COVID-19 shielding list		
No	2,175	14.4
Yes	122	19.2
Resident health board		
Aneurin Bevan	319	11.3
Betsi Cadwaladr	854	23.2
Cardiff and Vale	219	12.2
Cwm Taf Morgannwg	365	13.5

**Characteristics**	**N**	**%**
Hywel Dda	225	11.5
Powys	83	12.5
Swansea Bay	232	11.1
WIMD 2019 quintile		
1 (most deprived)	636	15.3
2	547	13.8
3	478	14.5
4	390	15.3
5 (most affluent)	246	14.2
Rurality classification		
Rural	718	14.6
Urban	1,579	14.6

a between 1st March 2020 to 31st November 2021; b data source = Social Care Wales (DSCW) data and include data that is self-reported by DCWs at 1st April. If missing, then other sources (ONS 2011 Census Wales (CENW), Wales Longitudinal General Practice (WLGP), Patient Episode Dataset for Wales (PEDW), Emergency Department Dataset (EDDS)) were used to impute (at index date=1st March 2020); c data source = WLGP; d data source=PEDW; e most recent occurring between 1st January 2000 and 1st March 2020; f based on residential address at 1st March 2020; g data source =Welsh Demographic Service Dataset (WDSD).

BMJ Publishing Group Limited (BMJ) disclaims all liability and responsibility arising from any reliance placed on this supplemental material which has been supplied by the author(s)

Supplementary material 6: Demographics of DCWs across the UK

Nation	Date published	Definition	Denom.	Age	Gender	Ethnicity
Wales[1]	April 2021	DCWs	22,131	44 years (mean)	84% F<br>16% M	97% White British
OSCAR sample	April 2020	DCWs	15,725	44 years (mean)	84% F<br>16% M	96% White British
England[2]	October 2021	DCWs	590,000	44 years (mean)	84% F<br>16% M	na
Scotland[3]	December 2020	Housing support/ care at home	74,870	46 years (median)	77% F<br>17 %M<br>6% unknown<br><br>84% F<br>16% M<br>(excluding unknowns)	98% White<br>2% Asian/Black
Northern Ireland[4]	October 2020	DCWs	16,303	20-29 years (mode)	91% F<br>8% M<br>1% Other	na

na = not available

[1] Domiciliary-care-worker-ENG.pdf (socialcare.wales) https://socialcare.wales/cms_assets/file-uploads/Domiciliary-care-worker-ENG.pdf

[2] The state of the adult social care sector and workforce in England (skillsforcare.org.uk) https://www.skillsforcare.org.uk/adult-social-care-workforce-data/Workforce-intelligence/publications/national-information/The-state-of-the-adult-social-care-sector-and-workforce-in-England.aspx

[3] Adults' services workforce tables - 2019 | Scottish Social Services Workforce Data (sssc.uk.com) https://data.sssc.uk.com/data-publications/248-adults-services-workforce-in-scotland-tables-2019

[4] Rapid learning review of domiciliary care in Northern Ireland (Department of Health) https://www.health-ni.gov.uk/rli-dom-care (page 13)

Cannings-John R, *et al. BMJ Open* 2023; 13:e070637. doi: 10.1136/bmjopen-2022-070637

BMJ Publishing Group Limited (BMJ) disclaims all liability and responsibility arising from any reliance placed on this supplemental material which has been supplied by the author(s)

The original protocol for the study, as a supplementary file

Cannings-John R, *et al. BMJ Open* 2023; 13:e070637. doi: 10.1136/bmjopen-2022-070637
