# Supplementary material for: Impact of the COVID-19 pandemic on domiciliary care workers in Wales, UK: a data linkage cohort study using the SAIL Databank
Source: BMJ Open. 2023 Jun 1;13(6):e070637. doi: 10.1136/bmjopen-2022-070637 (PMC10255029; doi:10.1136/bmjopen-2022-070637)
Supplement: Author's manuscript [file bmjopen-2022-070637.draft_revisions.pdf]

BMJ Open is committed to open peer review. As part of this commitment we make the peer review history of every article we publish publicly available.

When an article is published we post the peer reviewers' comments and the authors' responses online. We also post the versions of the paper that were used during peer review. These are the versions that the peer review comments apply to.

The versions of the paper that follow are the versions that were submitted during the peer review process. They are not the versions of record or the final published versions. They should not be cited or distributed as the published version of this manuscript.

BMJ Open is an open access journal and the full, final, typeset and author-corrected version of record of the manuscript is available on our site with no access controls, subscription charges or pay-per-view fees (http://bmjopen.bmj.com).

If you have any questions on BMJ Open's open peer review process please email info.bmjopen@bmj.com

BMJ Open

The impact of the COVID-19 pandemic on Domiciliary Care Workers in Wales (UK): a data linkage cohort study using the SAIL Databank.

Journal:	*BMJ Open*
Manuscript ID	bmjopen-2022-070637
Article Type:	Original research
Date Submitted by the Author:	30-Nov-2022
Complete List of Authors:	Cannings-John, Rebecca; Cardiff University<br>Schoenbuchner, Simon; Cardiff University, Centre for Trials Research<br>Jones, Hywel; Cardiff University, Division of Population Medicine<br>Lugg-Widger, Fiona; Cardiff University, Centre for Trials Research<br>Akbari, Ashley; Swansea University Medical School<br>Brookes-Howell, Lucy; Cardiff University Centre for Trials Research<br>Hood, Kerenza; Cardiff University, Centre for Trials Research<br>John, Ann; Swansea University, Health Data Research UK | Administrative Data Research Wales; Cardiff University, DECIPHer - Centre for Development, Evaluation, Complexity and Implementation in Public Health Improvement<br>Thomas, Daniel; Public Health Wales, Communicable Disease Surveillance Centre; Cardiff Metropolitan University<br>Prout, Hayley; Cardiff University School of Medicine, Centre for Trials Research<br>Robling, Michael; Cardiff University, Centre for Trials Research
Keywords:	COVID-19, MENTAL HEALTH, SOCIAL MEDICINE

SCHOLARONE™
Manuscripts

I, the Submitting Author has the right to grant and does grant on behalf of all authors of the Work (as defined in the below author licence), an exclusive licence and/or a non-exclusive licence for contributions from authors who are: i) UK Crown employees; ii) where BMJ has agreed a CC-BY licence shall apply, and/or iii) in accordance with the terms applicable for US Federal Government officers or employees acting as part of their official duties; on a worldwide, perpetual, irrevocable, royalty-free basis to BMJ Publishing Group Ltd ("BMJ") its licensees and where the relevant Journal is co-owned by BMJ to the co-owners of the Journal, to publish the Work in this journal and any other BMJ products and to exploit all rights, as set out in our licence.

The Submitting Author accepts and understands that any supply made under these terms is made by BMJ to the Submitting Author unless you are acting as an employee on behalf of your employer or a postgraduate student of an affiliated institution which is paying any applicable article publishing charge ("APC") for Open Access articles. Where the Submitting Author wishes to make the Work available on an Open Access basis (and intends to pay the relevant APC), the terms of reuse of such Open Access shall be governed by a Creative Commons licence – details of these licences and which Creative Commons licence will apply to this Work are set out in our licence referred to above.

Other than as permitted in any relevant BMJ Author's Self Archiving Policies, I confirm this Work has not been accepted for publication elsewhere, is not being considered for publication elsewhere and does not duplicate material already published. I confirm all authors consent to publication of this Work and authorise the granting of this licence.

For peer review only

Title: The impact of the COVID-19 pandemic on Domiciliary Care Workers in Wales (UK): a data linkage cohort study using the SAIL Databank.

Short running title: COVID-19 impact on domiciliary care workers

Authors:

Rebecca Cannings-John, PhD[1]

Simon Schoenbuchner, PhD[1]

Hywel Jones BSc DMS CStat[2]

Fiona Lugg-Widger, PhD[1]

Ashley Akbari, MSc[3]

Lucy Brookes-Howell, PhD[1]

Kerry Hood, PhD[1]

Ann John, MBBS MD [3,4]

Daniel Rh Thomas, PhD[5,6]

Hayley Prout, MPhil[1]

Michael Robling, PhD[1,4]

Affiliations: 1: Centre for Trials Research, School of Medicine, Cardiff University, Cardiff, United Kingdom; 2: Division of Population Medicine, Cardiff University; 3: Health Data Research UK (HDR UK) | Administrative Data Research Wales, Swansea University; 4: DECIPHer - Centre for Development, Evaluation, Complexity and Implementation in Public Health Improvement, sbarc|spark, Cardiff University; 5: Public Health Wales Communicable Disease Surveillance Centre; 6: Cardiff Metropolitan University.

Corresponding author:

Rebecca Cannings-John,
Centre for Trials Research,
Cardiff University,
Cardiff, UK
canningsrl@Cardiff.ac.uk
02920687248

For peer review only - http://bmjopen.bmj.com/site/about/guidelines.xhtml

ABSTRACT

Objectives: To quantify population health risks for domiciliary care workers in Wales working during the COVID-19 pandemic.

Design: A population-level retrospective routine data study linking occupational registration data to anonymised electronic health records maintained by the Secure Anonymised Information Linkage Databank in a privacy-protecting trusted research environment.

Setting: Registered domiciliary care workers population in Wales

Participants: We examined records for all linked domiciliary care workers in Wales from 1st March 2020 to 30th November 2021.

Primary and secondary outcome measures: Our primary outcome was confirmed COVID-19 infection; secondary outcomes included contacts for mental health, fit notes, respiratory infections not necessarily recorded as COVID-19, deaths involving COVID-19, all-cause mortality and suspected COVID-19.

Results: Confirmed COVID-19 rates increased over time to 24% by 30th November 2021. Rates varied by characteristics of care workers (e.g. lower in males and in older care workers), by where care workers lived (e.g. lower in rural areas, and more affluent areas), and were higher for care workers employed by local authority social services departments compared to staff employed in the private sector. 34% of care workers required support for a mental health condition, with general practice consultation and prescribing rates increasing in frequency when compared to the four years preceding the pandemic onset. COVID-19 related mortality are no greater than for the general population of 15- to 64-year-olds in Wales (0.034%).

Conclusions: While initial concerns about excess mortality are alleviated, the substantial pre-existing and increased mental health burden will require investment to provide long-term support to the sector's workforce.

Keywords: Domiciliary care worker, workforce, COVID-19, mental health, data linkage, social care, Wales

For peer review only - http://bmjopen.bmj.com/site/about/guidelines.xhtml

STRENGTHS AND LIMITATIONS OF THIS STUDY

- Relatively little is known about the objective health of the UK's domiciliary care workforce (DCWs), either pre- or during the COVID-19 pandemic; our study aims to address this deficit. As DCWs work with vulnerable clients, better protection for care workers is also likely to reduce risks to this sizeable community-based population.
- The linkage of registration data to electronic health records outcome data (including COVID-19 infection and mortality) provides a unique opportunity to explore a range of health outcomes for DCWs working in Wales during the pandemic.
- No DCWs were recorded as dissenting for their registration data to be made available for linkage.
- Ability to look at a range of outcomes and mental health outcomes over time in a time series analysis.
- Despite the comprehensive study population available in the OSCAR study, the recording of some data could introduce bias e.g. coding for COVID-19 biased towards individuals presenting in primary or secondary healthcare and the potential changing of coding due to service changes and consultation reductions in primary healthcare.

eview only

For peer review only - http://bmjopen.bmj.com/site/about/guidelines.xhtml

INTRODUCTION

During 2018-20, more than 950,000 people in the UK received domiciliary care from nearly 823,000 Domiciliary Care Workers (DCWs) from over 10,100 home care providers and the demand is predicted to increase significantly over the next 20 years [1]. DCWs provide support to adults in their own home with household tasks, personal care or any activities that allow the individual to maintain independence. Support can demand close contact, assisting with bodily functions, managing continence, assisting with oral and dental care, help with getting dressed, and providing other personal care.

From 1st April 2020, it became mandatory for DCWs in the active Welsh workforce to be registered with Social Care Wales. By June 2021, there were 22,131 registered DCWs in Wales, of whom 84% were female, with an average age of 44 years old, 51% worked in the private sector, and 36% had started in their current role within the previous three years [2]. Similarly, in England, most DCWs are female (84%), and work part-time (54%), about half work on zero hours contracts (48%) (the highest proportion of workers in adult social care), are aged on average 44 years old, and staff turnover is high (35%). During the pandemic, in England, roles in domiciliary care increased by around 7.4% compared to those in care homes, which observed a 0.2% decrease [3].

Since the emergence of COVID-19 in March 2020, a cross-sectional survey led by Ulster University found that during the early period of the COVID-19 pandemic (between May–July 2020 and November 2020–January 2021), both wellbeing and quality of working life deteriorated over all occupations including Social Care [4]. This decrease was again observed when care workers were surveyed between May and July 2021, with personal and work-related burnout reported to increase [5]. However, the survey did not aim to capture objectively recorded health outcomes specifically in DCWs, for example positive rates of COVID-19 infection and the risk factors which contribute to these.

The OSCAR study (Outcomes for Social Carers: an Analysis using Routine data) aimed to utilise the registration data collected by Social Care Wales, individually linked to secure anonymised electronic health record (EHR data sources) to understand the impact of COVID-19 on DCWs in Wales. Specifically we aimed to quantify rates of suspected and confirmed COVID-19 infection and key health outcomes as a consequence of COVID-19, explore outcome variation by demographics, work-related factors, lifestyle and comorbidities and over time, and examine how generalisable the quantitative findings were to other UK nations.

MATERIALS AND METHODS

Study design and data sources

For peer review only - http://bmjopen.bmj.com/site/about/guidelines.xhtml

This study is a population-level mixed methods retrospective routine data linkage study with analysis guided by exploratory qualitative interviews. Qualitative findings are reported separately [6]. Registration data for all DCWs in Wales registered by 1st April 2020 are held by Social Care Wales (DSCW) and available via the Secure Anonymised Information Linkage (SAIL) Databank (Swansea University) a privacy-protecting trusted research environment (TRE), which uses a standardised split-file secure, encrypted anonymisation process [7]. These data were combined with EHR data sources within the SAIL Databank (supplementary material 1 for data sources used). Prior to data being transferred to SAIL Databank from SCW, all DCWs had the opportunity to opt-out of their data going into the SAIL Databank for use in research.

For peer review only

Population

The study population was all registered DCWs resident in Wales on 1st March 2020 who did not subsequently opt-out to their data being linked for research, when DSCW data were added to the SAIL Databank or via their general practice (GP). Any DCWs not linked into the SAIL Databank with sufficient confidence were excluded. Additionally, individuals not registered as DCWs (including Domiciliary Care Managers, Adult Care Home Managers and Residential Child Care Workers) were excluded.

Primary and secondary outcomes

The primary outcome was confirmed COVID-19 infection, defined as the earliest of the following events: a positive polymerase chain reaction (PCR) test (Pathology Test Results dataset, PATD) [8], hospital admission (Patient Episode Database for Wales, PEDW) [9], death registration from COVID-19 (main or secondary diagnostic ICD-10 code=U07.1) (Annual District Death Extract, ADDE from the Office for National Statistics (ONS) mortality register) [10], or a COVID-19 diagnosis Read code (Welsh Longitudinal GP, WLGP) [11].

Secondary outcomes were:

- Suspected COVID-19 infection (Data sources: PATD, WLGP, PEDW, and ADDE);
- Contacts for mental health and diagnoses, psychotropic medication and admissions (including self-harm) (Data sources: WLGP, and PEDW);
- Fit notes as a general marker of medically confirmed (return from) illness (Data source: WLGP);
- Non-COVID-19 hospital admissions for respiratory conditions (Data source: PEDW);
- Deaths involving COVID-19 and all-cause mortality (Data source: ADDE).

For peer review only - http://bmjopen.bmj.com/site/about/guidelines.xhtml

In brief, we examined depression and anxiety symptoms and diagnoses [12,13], and severe mental health in GP data and hospital admissions data [14]. Self-harm, used GP data for definite and undetermined intent (WLGP) [15,16]. Fit notes also used GP data to cover medical/sickness/self-certificates issued to patients by GPs and included issues for returning to work. Non-COVID-19 hospital admissions for respiratory conditions were examined for potential miscoding of COVID-19 infection in the early pandemic and included any lower respiratory infection, pneumonia, and influenza-like-illness [17], exacerbations of chronic obstructive pulmonary disease (COPD) [18] and asthma [19].

Study period and follow-up

DCWs entered the cohort on 1st March 2020 and were followed up until 30th November 2021. We examined outcomes by waves of the pandemic; short term outcomes from 1st March 2020 to 31st August 2020 (Wave 1: reflecting a period before the relaxing of lockdown in Wales) and from 1st September 2020 to 28th February 2021 (Wave 2). Long-term outcomes were examined to 30th November 2021. DCWs were followed up until the earliest of occurrence of death, first migration out of Wales, the outcome of interest, or the end of the follow-up.

Domiciliary Care Worker Characteristics

We chose age, sex, ethnicity, health board, deprivation quintile and rurality as relevant characteristics for the DCWs using a number of data sources (supplementary material 1). We also identified DCWs living with disability, any comorbidities [14], shielding status, and lifestyle factors such as body mass index and smoking status. From the DSCW data, we were able to describe how individuals registered as a DCW (induction, registered with a qualification, competence confirmed by their manager) which reflects the different pathways open to care workers when mandatory registration was introduced [20]. Some pathways were transitional and are now no longer available (e.g. registration following induction). We also identified whether a DCW lived with another DCW and their employment sector at the time of registration (Private, Third sector, Local Authority/Social Services, Recruitment/Employment Agency/Other). Several items from the DSCW were not available due to confidentiality considerations, such as length of time in the job, type of employment (e.g.in social care, self-employed), and type of qualifications held (Qualifications and Credit Framework (QCF), National Vocational Qualification (NVQ) etc).

A full coding list for outcomes is in supplementary material 2 and also on Open Science Framework (OSF) (https://osf.io/9w6pe/) alongside codes for the comorbidities.

Governance

For peer review only - http://bmjopen.bmj.com/site/about/guidelines.xhtml

The study received approval from the School of Medicine Research Ethics Committee, Cardiff University [SMREC 20/114]. Approval was obtained from the SAIL Information Governance Review Panel (IGRP), Swansea University (Project ID: 1126) and data access was granted to named staff. Approval from the ONS Research Accreditation Panel (ONS RAP) was provided (16/12/2020) and approval from Social Care Wales was provided (26/01/2021). This process was generic and not specific to the OSCAR study.

Analysis

The linkage of DCWs to EHR were described and the study population characterised using frequencies and percentages. The presence of key health outcomes were described by the short and long-term periods; prevalence estimates presented alongside 95% confidence intervals (CIs). This study is primarily designed to describe variation in outcomes in the DCW population, by key subgroups, rather than for prediction or causal models. Using multilevel Cox regression to model time to first confirmed COVID-19 infection, we estimated hazard ratios (HRs) with 95% CIs, to quantify the risk of confirmed COVID-19 by demographics (age, sex, ethnicity, deprivation quintile), work-related factors (sector employed), lifestyle (smoking, BMI) and comorbidities. A similar modelling method was applied to fit notes. DCWs were censored at the time of the event, migration, death, or at the study end. The analysis approach for mental health was changed (and as such was exploratory) to examine which factors varied with pre-existing or newly arising mental ill health identified during the study time-period (as opposed to COVID-19 pandemic-related mental health) with a logistic regression estimating ORs alongside 95% CI. Equivalent up-to-date DCW workforce data from Scotland, Northern Ireland (NI) and England was obtained and compared to the Welsh DCW cohort. The prevalence estimates of outcomes were generalised to other nations.

To explore changes over time and the effect of the COVID-19 pandemic period, an interrupted time series approach was used to model hospital admissions for non-COVID-19 respiratory infections. Monthly data were divided into pre-pandemic (1st March 2016 to 28th February 2020) and the pandemic period (1st March 2020 to 31st October 2022). The changes in levels (baseline value of the outcome at time zero) and trends (the rate of change) of the pre-pandemic segment and pandemic segment were analysed using the *itsa* command in Stata. A Cumby-Huizinga test was used to determine the autocorrelation and was adjusted using Newey West AC estimators. Using the same approach and as a post-hoc analysis, the number of mental health GP contacts and prescriptions, and hospital admissions were also examined.

To comply with disclosure control process and approvals, and protect the privacy of anonymised individuals, numbers <5 are suppressed. Percentages associated with small frequencies are rounded to the nearest 1%. Study findings were reported in accordance with applicable reporting guidelines for

For peer review only - http://bmjopen.bmj.com/site/about/guidelines.xhtml

observational studies using administrative data (STROBE and RECORD) (supplementary table 3) [21,22]. Data cleaning, cohort assembly and statistical analyses were performed using Structured Query Language (SQL) (IBM Db2 11.1) [23] and R (v4.1.0-4.1.3) [24,25], and Stata (v17)[26] within the SAIL Databank privacy-protecting TRE.

Patient and public involvement

Two stakeholder groups provided input to the project, an ongoing Study Advisory Group and an Implementation Reference Group. Both groups included membership drawn from the domiciliary care sector. The Advisory Group met four times and provided scientific and policy oversight, monitored study progress and contributed to interpretation of emerging results. Membership included representation from a care workers charity, the care sector regulator and a service user. The Implementation Reference Group met twice and contributed to discussions about emerging findings, developing recommendations for policy and practice and advised on implementation routes and modes. Its membership included representation from care workers (including care manager), care users, trade unions, regulator, sector skills council and policy. Two policy briefings were co-produced with input from the IRG membership.

RESULTS

Study population

Data from 15,931 DCWs resident and working in Wales on 1st March 2020 were linked to EHR data (85% of them had GP records in SAIL) with 15,725 (98.7%) DCWs included in the final analysis. The majority of DCWs were female (84%), of white ethnic background (95%), 43% had at least one comorbidity of which 22% had been recorded with mental health related illness (Table 1).

Confirmed COVID-19 infection

Confirmed COVID-19 infection was 1.2% at 31st August 2020, rose to 14% by the end of February 2021, and reached 24% by the end of November 2021 (Table 2). There was no evidence to suggest a miscoding of COVID-19 infection in the early pandemic, with no difference in admissions for non-COVID-19 respiratory conditions over time (supplementary material 4). Confirmed COVID-19 infection was more prevalent in females, those who registered following the induction pathway compared to those with competency confirmed by a manager, those employed by the Local Authority Social Services at registration (Figure 1) compared to those employed in the private sector, those

For peer review only - http://bmjopen.bmj.com/site/about/guidelines.xhtml

living with another DCW, those with a BMI greater than 30 compared to those with a healthy BMI (18.5-24.9) and those living in an urban area (Table 1; supplementary material 5). Confirmed COVID-19 infection was less likely in older DCW. Variation was also observed across health boards. After adjusting for age, sex and deprivation, COVID-19 infection was still more prevalent in those with a co-morbidity (HR=1.08, 95% CI: 1.01 to 1.15) and additionally adjusting for health board, rurality, and qualification type, was more prevalent in those employed by the Local Authority Social Services at registration (1.35, 1.23 to 1.47).

Table 1. Characteristics of Domiciliary Care Workers, confirmed COVID-19 infection and contacts for mental health and diagnoses, psychotropic medication and admissions

	DCWs N=15,725		Crude HR (95% CI)	Crude OR (95% CI)
**DCW Characteristics**	**N**	**%**	**Confirmed COVID-19 infection[a]**	**Mental health[a]**
Sex[b]				
Female	13,253	84.3	ref	ref
Male	2,472	15.7	0.76 (0.69 to 0.84)	0.61 (0.55 to 0.67)
Age (at registration)[b]				
Under 35 years	4,425	28.1	ref	ref
35 to <45 years	2,822	17.9	1.07 (0.97 to 1.17)	0.99 (0.90 to 1.09)
45 to <55 years	3,955	25.2	0.85 (0.78 to 0.93)	1.02 (0.93 to 1.12)
55 years and over	4,523	28.8	0.68 (0.62 to 0.74)	0.82 (0.75 to 0.90)
Ethnicity[b]				
White	15,012	95.5	ref	ref
Black	142	0.9	1.06 (0.76 to 1.48)	0.35 (0.22 to 0.55)
Mixed	127	0.8	0.98 (0.68 to 1.42)	0.73 (0.49 to 1.07)
Asian	108	0.7	1.01 (0.68 to 1.50)	0.39 (0.22 to 0.62)
Other	66	0.4	0.86 (0.50 to 1.48)	0.39 (0.19 to 0.71)
Not recorded	*270*	*1.7*		
Lives with DCW[b]				
No	14,750	93.8	ref	ref
Yes	975	6.2	1.15 (1.01 to 1.30)	0.92 (0.80 to 1.06)
Have disability[b]				
No	14141	89.9	ref	ref
Yes	188	1.2	0.67 (0.47 to 0.96)	1.88 (1.41 to 2.50)
Not recorded /Prefer not to answer	*1,396*	*8.9*	-	-
Qualifications as recorded at registration[b]				
Competence confirmed	1982	12.6	ref	ref
Induction Framework	2984	19.0	1.15 (1.02 to 1.29)	1.20 (1.06 to 1.35)
Qualification Level 2	6935	44.1	1.12 (1.00 to 1.24)	1.05 (0.94 to 1.16)
Qualification Level 3	3340	21.2	1.03 (0.91 to 1.15)	0.98 (0.87 to 1.11)
Qualification Level 4+	441	2.8	0.91 (0.72 to 1.14)	0.96 (0.77 to 1.20)

For peer review only - http://bmjopen.bmj.com/site/about/guidelines.xhtml

DCW Characteristics	DCWs N=15,725 N	%	Crude HR (95% CI) Confirmed COVID-19 infection[a]	Crude OR (95% CI) Mental health[a]
Not recorded	*43*	*0.3*	-	
Employment sector as recorded at registration[b]				
Private	6,592	41.9	Ref	Ref
Third Sector	4,267	27.1	1.03 (0.95 to 1.12)	1.06 (0.98 to 1.16)
Local Authority-Social Services	3,306	21.0	1.23 (1.13 to 1.34)	1.11 (1.02 to 1.21)
Recruitment Agency/ Employment Agency	148	0.9	1.14 (0.84 to 1.54)	1.25 (0.91 to 1.71)
Health/Other	24	0.2		
Not recorded	*1388*	*8.8*		
Smoking status[c]				
Non-smoker	6,342	40.3	ref	ref
Ex-smoker	3,509	22.3	0.97 (0.89 to 1.05)	1.39 (1.28 to 1.52)
Current smoker	4,374	27.8	0.74 (0.68 to 0.80)	1.41 (1.30 to 1.53)
Not recorded	*1,500*	*9.5*	-	-
Body Mass Index[c]				
Underweight (<18.5)	256	1.6	0.84 (0.63 to 1.13)	1.28 (0.99 to 1.67)
Healthy weight (18.5-24.9)	3,173	20.2	ref	ref
Overweight (25-29.9)	3,652	23.2	1.09 (0.99 to 1.21)	1.18 (1.07 to 1.31)
Obese (≥30)	5,311	33.8	1.27 (1.16 to 1.39)	1.58 (1.44 to 1.73)
Not recorded	*3,333*	*21.2*	-	-
Any comorbidity[c,d,e]	6,804	43.3	1.07 (1.00 to 1.14)	2.90 (2.71 to 3.11)
Mental health related illnes[f]	3,431	21.8	-	
Asthma	2,699	17.2	-	
Coronary Heart Disease (CHD)	281	1.8	-	
Chronic Kidney Disease (CKD)	132	0.8	-	
Chronic Obstructive Pulmonary Disease (COPD)	299	1.9	-	
Epilepsy	238	1.5	-	
Osteoporotic fracture	462	2.9	-	
Stroke	135	0.9	-	
Diabetes	824	5.2	-	
On COVID-19 shielding list				
No	15,090	96.0	ref	ref
Yes	635	4.0	0.85 (0.72 to 1.02)	1.45 (1.23 to 1.70)
**Area level characteristics**[g,h]				
Resident health board				
Aneurin Bevan	2,819	17.9	ref	ref
Betsi Cadwaladr	3,683	23.4	0.63 (0.57 to 0.70)	0.84 (0.76 to 1.70)
Cardiff and Vale	1,796	11.4	0.96 (0.85 to 1.07)	1.06 (0.94 to 1.20)
Cwm Taf Morgannwg	2,702	17.2	1.05 (0.95 to 1.16)	1.25 (1.12 to 1.39)
Hywel Dda	1,965	12.5	0.69 (0.61 to 0.78)	0.75 (0.66 to 0.85)
Powys	662	4.2	0.53 (0.43 to 0.65)	0.35 (0.28 to 0.44)
Swansea Bay	2,098	13.3	0.96 (0.86 to 1.07)	1.12 (1.00 to 1.26)
WIMD 2019 quintile				

For peer review only

	DCWs N=15,725		Crude HR (95% CI)	Crude OR (95% CI)
DCW Characteristics	N	%	Confirmed COVID-19 infection[a]	Mental health[a]
1 (most deprived)	4,168	26.5	ref	ref
2	3,970	25.2	0.90 (0.83 to 0.98)	0.92 (0.84 to 1.01)
3	3,301	21.0	0.74 (0.67 to 0.81)	0.75 (0.68 to 0.82)
4	2,554	16.2	0.68 (0.61 to 0.76)	0.75 (0.67 to 0.83)
5 (most affluent)	1,732	11.0	0.74 (0.65 to 0.83)	0.86 (0.77 to 0.97)
Rurality classification				
Rural	4,932	31.4	ref	ref
Urban	10,793	68.6	1.38 (1.28 to 1.49)	1.36 (1.26 to 1.46)

a between 1st March 2020 to 31st November 2021; b data source = Social Care Wales (DSCW) data and include data that is self-reported by DCWs at 1st April. If missing, then other sources (ONS 2011 Census Wales (CENW), Wales Longitudinal General Practice (WLGP), Patient Episode Dataset for Wales (PEDW), Emergency Department Dataset (EDDS)) were used to impute (at index date=1st March 2020); c data source = WLGP; d data source=PEDW; e most recent occurring between 1st January 2000 and 1st March 2020; f Contacts and diagnoses for metal health and admissions; g based on residential address at 1st March 2020; h data source =Welsh Demographic Service Dataset (WDSD).

Table 2. Key outcomes by pandemic waves

	Short term (Wave 1) 1st March to 31st August 2020		Short term (Wave 1 and 2) 1st March 2020 to 28th February 2021		Long term 1st March 2020 to 30th November 2021	
Outcome	n	%	n	%	n	%
Confirmed COVID-19 infection	182	1.2	2,196	14.0	3,698	23.5
Suspected COVID-19 infection	326	2.1	2,335	14.8	3,822	24.3
Mental health[a]	3641	23.2	4,392	27.9	5,300	33.7
Self-harm	20	0.1	35	0.2	64	0.4
Fit notes	857	5.4	1,551	9.9	2,297	14.6
Non-COVID-19 respiratory infections[b]	126	0.8	300	1.9	467	3.0
All-cause mortality	*	*	*	*	23	0.1

* short term results suppressed as de-identifiable by subtraction from the long term results.

a Contacts and diagnosis for mental health, psychotropic medication and admissions; b Respiratory infections not necessarily recorded as COVID-19 infection.

Mental health

Contacts and diagnosis for mental health, psychotropic medication, and admissions were 23% in August 2020, rising to 28% and 34% by the end of February and November 2021 respectively (Table 2). The increase in mental health issues over time was also apparent when examining the monthly number of mental health related medication and contacts with GPs; prescribing was 13% in March 2016 rising to 20% in March 2020 (Figure 2a), increasing on average per month by 17.61 (95% CI:

15.51 to 19.70, p-value <0.001). No evidence of a change was observed immediately following the start of the pandemic (March to April 2020) but evidence of an increase after April 2020 compared to the pre–pandemic with an average monthly increase of 29.34 (20.71 to 37.97, <0.001). Similar patterns were observed for GP consultations. Mental health related hospital admissions were lower in March 2016 (around 15 admission per month) and significantly increased on average per month by 0.15 (0.06 to 0.25, 0.002) (Figure 2b). There was evidence to suggest a decrease in admissions immediately following the start of the pandemic but on increased on average by 0.99 admissions per month (0.44 to 1.53, <0.001), returning to pre-pandemic by the end of the study period.

Variations in mental health contacts were observed across many of the key demographics (e.g., lower in males and older care workers), and consistently higher in care workers with a disability reported at registration, with a comorbidity or shielding (Table 2, supplementary material 6). There was no variation in mental health contacts for the care workers by their qualification status; care workers in the local authority sector had slightly elevated risk of mental ill-health compared to those employed by the private sector at registration.

Fit notes

The issuing of fit notes as a general marker of medically confirmed (and return from) illness, increased over the time-period from 5% in August 2020, to 10% by at the end of February and 15% by the end of November 2021. Fit notes were more prevalent in females, those who registered following the induction pathway or held a Level 2 Qualification (compared to those with competency confirmed by a manager), those employed by the Local Authority Social Services at registration (compared to those employed in the private sector), DCWs with a comorbidity or were shielding, and those with a BMI greater than 30 (compared to those with a healthy BMI: 18.5-24.9) (supplementary material 7). Unlike confirmed COVID-19 infection, fit notes were more likely in those that were older (aged 45 and over). Both Betsi Cadwaladr and Powys health boards also had a higher rate of fit notes issued for DCWs, whereas Swansea Bay had less (when compared to Aneurin Bevan).

Suspected COVID-19 infection

Suspected COVID-19 infections increased over the pandemic from 2% in early pandemic to 15% by at the end of February, rising to 24% by the end of November 2021. Flagged suspected COVID-19 infection added little to the clinical picture given the relatively widespread availability of COVID-19 testing across the follow-up period and uncertainty regarding the fields that were used (i.e. just having a COVID-19 test was not a good indicator of having COVID-19 infection). No further modelling was therefore conducted.

For peer review only - http://bmjopen.bmj.com/site/about/guidelines.xhtml

Generalisability

Up-to-date DCW workforce from Scotland [27] and England [28] shows that the case-mix of these DCW populations are comparable to (and in some cases identical to) that presented for Wales with respect to age, sex, and ethnicity (supplementary material 8) and we would expect the prevalence of COVID-19 in these nations to therefore be similar. For domiciliary care workers in England based on a population of 590,000, the impact of meatal health illness is around 200,000. However, data from Northern Ireland (NI) showed that DCWs are younger on average (mode 20-29 years) (Department of Health), and the prevalence of COVID-19 infection may have been greater in this DCW workforce.

DISCUSSION

Principal findings

Confirmed COVID-19 rates increased from 14% during the first two pandemic waves (1st March 2020 to 28th February 2021) to 24% by the end of November 2021. Infection rates varied by personal characteristics of care workers (lower in males and in older care workers), by where care workers lived (lower in health boards such as Powys, in rural areas, in more affluent areas), and were higher for care workers employed by local authority social services departments compared to staff employed in the private sector. One third (34%) of all care workers required support for a mental health condition, with rates of attending a GP or receiving a relevant prescription higher compared to the four years preceding pandemic onset. Rates in mental health contacts varied by key demographics, and were consistently higher in care workers with a comorbidity, disability or who were shielding. Local authority care workers had a slightly elevated risk of mental health problems compared to those employed by the private sector. The mortality rate amongst care workers was no greater than that observed amongst the general population of 15- to 64-year-olds in Wales (which was 0.034%). Data from DCW workforces in England and Scotland showed a similar demographic with respect to age, sex and ethnicity. The impact of COVID-19 infection would therefore be similar to that observed in Wales. The NI workforce was younger and therefore we may expect a high prevalence rate.

Strengths and weaknesses

A principal strength of the study was the comprehensive and contemporaneous coverage of the care worker population in Wales through linkage to registration data. This contrasts with approaches reliant on census data which may be incomplete, at risk of being out of date or on sample surveys with varying levels of non-response [30,31]. With few records lost to linkage failure or opt-out, the study

For peer review only - http://bmjopen.bmj.com/site/about/guidelines.xhtml

cohort reflects well the population of DCWs reported by the regulator [2]. Access to EHRs in the SAIL Databank allowed for broader assessment of health than previously reported by ONS, which understandably focused on mortality in the early period of the pandemic [30]. Accessing SAIL data utilised coding developed for existing conditions and novel indications (e.g. COVID-19) enabling rapid application and greater confidence than coding developed from scratch [32].

The registration process was introduced by Social Care Wales to meet regulatory rules that came into force from 1st April 2020 [33]. Our qualitative interviews revealed greater heterogeneity in the registered workforce than anticipated [6]. Workers in supported shared living homes were registered as well as workers travelling to care for individuals living in their own home, detail unavailable from registration data alone. Our interviews highlighted that while there are some distinct role features (e.g. care workers in supported shared living homes may spend time working overnight) there is commonality both in terms of potential exposure risk and approaches to mitigation.

Administrative records do not usually capture subjective patient reported outcomes or wellbeing concerns. This may be a greater consideration if care workers felt reluctant or less able to present concerns to healthcare professionals during the pandemic, as has been observed in other settings [34]. Both quantitative and qualitative studies, involving a range of community-based health and social care staff provide ample evidence of impacts of the pandemic on the wellbeing of professionals [4–6,35,36]. Findings from our routine data study builds a more complete picture of the health and wellbeing consequences of the pandemic for care workers.

Outcome rates varied considerably based on characteristics and circumstances of care workers, such as age and geographical location. Such differences may not be directly attributable to the working conditions of care workers or even directly addressable. Nevertheless, it highlights that risks for care workers remain a vital consideration for employers. It remains to be determined whether any facet of the employment role such as staff training, occupational risk assessment or testing procedures may have contributed these differences or provide an opportunity to intervene.

Some methodological caveats include that we could not distinguish between DCWs who did not consult with their GP or where their GP had not contributed data to SAIL. This will result in downward biased GP outcome rates (e.g. if a mental health diagnosis truly occurred then a mental health diagnosis will be misclassified as having not occurred). Secondly, if missing GP data are not at random (e.g., if there is less GP coverage in some regions), it is more likely that we would have missed mental health diagnosis or fit notes from DCWs with certain characteristics, resulting in bias in our parameter estimates (ORs/HRs) for these outcomes. Thirdly, DCWs were followed up for different durations (due to migration in and out of Wales or from contributing/non-contributing GPs, or deaths). Whilst the time to event analyses (and corresponding HRs) account for this by censoring,

For peer review only - http://bmjopen.bmj.com/site/about/guidelines.xhtml

summary statistics and ORs do not. Lastly, cohort ageing and differences in cohort entry or exit dates would likely lead to upwards bias in the overall trends in the time series analyses.

Findings in context

ONS data from the initial three-month period of the pandemic suggested mortality rates for individuals employed as care workers and home carers exceeded that found in the general population [30]. However, data on occupational classification was missing for a large proportion of males and females dying with some COVID-19 involvement and, even where available, data on occupation may have been incorrect due to role and job changes since census data were provided. Our findings are more in line with the initial estimates provided by the Public Health England survey in June 2020 [31]. Our findings provide health outcome estimates over a much longer period, for a population rather than sample of care workers and for a broader range of outcomes.

Previous research on care worker health outcomes has often focused on musculoskeletal problems and dealing with challenging behaviour [37]. Nevertheless, pre-pandemic work has emphasised some broader harms experienced by a range of community-based home care workers (e.g. infection control) and the benefits that workers derive from their work [38–40]. Sterling and colleagues used US national CDC Behavioral Risk Factor Surveillance Survey data to explore outcomes of home health care workers employed by home care agencies, revealing [38]. Despite role differences between the US sample and domiciliary care workers in Wales, the pre-pandemic levels of mental ill-health are strikingly similar.

Evidence from our own work and that of others supports the value in co-produced solutions which draw on the direct experiences of care workers [41]. Systemic drivers (e.g. public funding for social care, staffing levels, levels of pay) and situational aspects of the role such as peripatetic working will not change quickly or even at all. With few evidence-based supportive approaches tailored to the circumstances of care workers [42] [43] optimising or innovating approaches to support the UK community of care workers may offer considerable long-term benefits to workforce and clients alike.

Conclusion

Higher rates of confirmed COVID-19 infection amongst domiciliary care workers in Wales did not translate into mortality rates greater than for the broader Welsh population. High baseline rates of mental ill-health further increased over time, a burden that fell unevenly across the workforce.

For peer review only - http://bmjopen.bmj.com/site/about/guidelines.xhtml

Funding

This research was funded by the Economic & Social Research Council (ESRC), as part of UK Research & Innovation's rapid response to COVID-19 (ES/V015206/1).

Competing Interest

The authors declare that they have no conflict of interest and that they satisfy the conditions for authorship.

Data sharing statement: Data are available upon reasonable request. The routine data used in this study are available in the SAIL Databank at Swansea University, Swansea, UK. All proposals to use SAIL data are subject to review by an IGRP. Before any data can be accessed, approval must be given by the IGRP. The IGRP gives careful consideration to each project to ensure proper and appropriate use of SAIL data. When access has been approved, it is gained through a privacy protecting safe haven and remote access system referred to as the SAIL Gateway. SAIL has established an application process to be followed by anyone who would like to access data via SAIL: https://www.saildatabank.com/application-process. This study has been approved by the IGRP as project 1126.

Author contributions: RCJ and MR were co-chief investigators of the study and led the drafting of the main manuscript. RM, RCJ, AJ, FLJ, AA, KH, and DT conceived and contributed to the design of the study. AA was responsible for managing access to data within the SAIL Databank and advising on constituent data sets. HJ was responsible for data management and preparing data for analysis. FLW was responsible for study management. SS and RCJ were responsible for developing the statistical analysis plan and conducting the main analysis. All authors contributed to the design and conduct of the study and interpretation of the analyses. All authors were responsible for reviewing and revising drafts of the manuscript and providing final approval.

Acknowledgements: The Centre for Trials Research receives funding from Health and Care Research Wales and Cancer Research UK. The data acquisition and COVID-19 research was supported by Health Data Research UK [HDR-9006], which receives its funding from HDR UK Ltd, and the ADR Wales programme of work) funded ADR UK (grant ES/S007393/1). The study team gratefully acknowledge the work of the Study Advisory Group for providing independent oversight and input; the Implementation Reference Group for reviewing early findings and co-producing recommendations for short and longer-term outcomes; and all domiciliary care workers who contributed their data for inclusion in the study analyses. This study makes use of anonymised data held in the Secure

For peer review only - http://bmjopen.bmj.com/site/about/guidelines.xhtml

Anonymised Information Linkage (SAIL) Databank. We would like to acknowledge all the data providers who make anonymised data available for research. We acknowledge the support of Social Care Wales in advising us at different stages of the study, supporting access to the registration data and aiding our reflection on study findings. This is an open access article distributed in accordance with the Creative Commons Attribution 4.0 Unported (CC BY 4.0) license, which permits others to copy, redistribute, remix, transform and build upon this work for any purpose, provided the original work is properly cited, a link to the licence is given, and indication of whether changes were made. See: https://creativecommons.org/licenses/by/4.0/.

Ethics Approval Statement

The study received approval from the School of Medicine Research Ethics Committee, Cardiff University [SMREC 20/114]. Approval was obtained from the SAIL Information Governance Review Panel (IGRP), Swansea University (Project ID: 1126) and data access was granted to named staff. Approval from the ONS Research Accreditation Panel (ONS RAP) was provided (16/12/2020) and approval from Social Care Wales was provided (26/01/2021). This process was generic and not specific to the OSCAR study.

References

1 Homecare. Home care facts and stats: number of providers, service users & workforce. 2021.https://www.homecare.co.uk/advice/home-care-facts-and-stats-number-of-providers-service-users-workforce (accessed 15 Jun 2022).

2 Social Care Wales. Domiciliary Care Workers on the Register. 2020. 2020. https://socialcare.wales/cms_assets/file-uploads/Domiciliary-Care-Workers-on-the-Register.pdf (accessed 16 Jun 2022).

3 Skills for care. Workforce Intelligence Summary Domiciliary care services in the adult social care sector 2020/21. 2021. https://www.skillsforcare.org.uk/adult-social-care-workforce-data-old/Workforce-intelligence/documents/State-of-the-adult-social-care-sector/Summary-of-domiciliary-care-services-2021.pdf (accessed 15 Jul 2022).

4 McFadden P, Neill RD, Moriarty J, *et al.* A Cross-Sectional Examination of the Mental Wellbeing, Coping and Quality of Working Life in Health and Social Care Workers in the UK at Two Time Points of the COVID-19 Pandemic. *Epidemiologia* 2021;**2**:227–42. doi:10.3390/epidemiologia2030017

5 Gillen P, Neill RD, Manthorpe J, *et al.* Decreasing Wellbeing and Increasing Use of Negative Coping Strategies: The Effect of the COVID-19 Pandemic on the UK Health and Social Care Workforce. *Epidemiologia* 2022;**3**:26–39. doi:10.3390/epidemiologia3010003

For peer review only - http://bmjopen.bmj.com/site/about/guidelines.xhtml

6 Prout H, Lugg-Widger F v., Brookes-Howell L, *et al.* "I don't mean to be rude, but could you put a mask on while I'm here?" A qualitative study of risks experienced by domiciliary care workers in Wales during the COVID-19 pandemic. *Health Soc Care Community* Published Online First: 24 November 2022. doi:10.1111/hsc.14109

7 Lyons RA, Jones KH, John G, *et al.* The SAIL databank: linking multiple health and social care datasets. *BMC Med Inform Decis Mak* 2009;**9**:3. doi:10.1186/1472-6947-9-3

8 SAIL Databank. COVID-19 Pathology Test Results. 2022.https://data.ukserp.ac.uk/Asset/View/53 (accessed 16 Jun 2022).

9 SAIL Databank. Patient Episode Database for Wales. 2022.https://data.ukserp.ac.uk/Asset/View/15 (accessed 16 Jun 2022).

10 SAIL Databank. Annual District Death Extract. 2022.https://data.ukserp.ac.uk/Asset/View/3 (accessed 16 Jun 2022).

11 SAIL Databank. Primary Care GP Dataset. 2022.https://data.ukserp.ac.uk/Asset/View/17 (accessed 16 Jun 2022).

12 John A, McGregor J, Fone D, *et al.* Case-finding for common mental disorders of anxiety and depression in primary care: an external validation of routinely collected data. *BMC Med Inform Decis Mak* 2016;**16**:35. doi:10.1186/s12911-016-0274-7

13 Rahman MA, Todd C, John A, *et al.* School achievement as a predictor of depression and self-harm in adolescence: linked education and health record study. *The British Journal of Psychiatry* 2018;**212**:215–21. doi:10.1192/bjp.2017.69

14 Clift AK, Coupland CAC, Keogh RH, *et al.* Living risk prediction algorithm (QCOVID) for risk of hospital admission and mortality from coronavirus 19 in adults: national derivation and validation cohort study. *BMJ* 2020;:m3731. doi:10.1136/bmj.m3731

15 Thomas KH, Davies N, Metcalfe C, *et al.* Validation of suicide and self-harm records in the Clinical Practice Research Datalink. *Br J Clin Pharmacol* 2013;**76**:145–57. doi:10.1111/bcp.12059

16 Marchant A, Turner S, Balbuena L, *et al.* Self-harm presentation across healthcare settings by sex in young people: an e-cohort study using routinely collected linked healthcare data in Wales, UK. *Arch Dis Child* 2020;**105**:347–54. doi:10.1136/archdischild-2019-317248

17 London School of Hygiene and Tropical Medicine Data Compass. Data Compass. https://datacompass.lshtm.ac.uk/id/eprint/2193/ (accessed 16 Jun 2022).

18 London School of Hygiene and Tropical Medicine. Data Compass. https://datacompass.lshtm.ac.uk/id/eprint/2037/1/copd_hes.txt (accessed 16 Jun 2022).

19 London School of Hygiene and Tropical Medicine. Data Compass. https://datacompass.lshtm.ac.uk/id/eprint/940 (accessed 16 Jun 2022).

20 Social Care Wales. Qualifications needed. 2022.https://socialcare.wales/registration/qualifications-needed#section-29926-anchor (accessed 8 Jul 2022).

For peer review only - http://bmjopen.bmj.com/site/about/guidelines.xhtml

21 Benchimol EI, Smeeth L, Guttmann A, *et al.* The REporting of studies Conducted using Observational Routinely-collected health Data (RECORD) Statement. *PLoS Med* 2015;**12**:e1001885. doi:10.1371/journal.pmed.1001885

22 Elm E von, Altman DG, Egger M, *et al.* Strengthening the reporting of observational studies in epidemiology (STROBE) statement: guidelines for reporting observational studies. *BMJ* 2007;**335**:806–8. doi:10.1136/bmj.39335.541782.AD

23 IBM Corp. Db2 for z/OS (Version 11.1). 2022.

24 R Core Team. R: A language and environment for statistical computing. R Foundation for Statistical Computing, Vienna, Austria,. Published Online First: 2022.www.R-project.org (accessed 8 Jul 2022).

25 Wickham H, Averick M, Bryan J, *et al.* Welcome to the Tidyverse. *J Open Source Softw* 2019;**4**:1686. doi:10.21105/joss.01686

26 StataCorp. Stata Statistical Software: Release 17. College Station, TX: StataCorp LLC. 2021.

27 Scottish Social Services Council. Adults' services workforce tables - 2019 | Scottish Social Services Workforce Data (). 2019. Adults' services workforce tables - 2019 | Scottish Social Services Workforce Data (sssc.uk.com) https://data.sssc.uk.com/data-publications/248-adults-services-workforce-in-scotland-tables-2019 (accessed 16 Jun 2022).

28 Skills for Care. The state of the adult social care sector and workforce in England. Published Online First: 2022.https://www.skillsforcare.org.uk/adult-social-care-workforce-data/Workforce-intelligence/publications/national-information/The-state-of-the-adult-social-care-sector-and-workforce-in-England.aspx (accessed 16 Jun 2022).

29 Department of Health. Rapid learning review of domiciliary care in Northern Ireland. https://www.health-ni.gov.uk/rli-dom-care (accessed 16 Jun 2022).

30 Office for National Statistics. Coronavirus (COVID-19) related deaths by occupation, England and Wales. https://www.ons.gov.uk/peoplepopulationandcommunity/healthandsocialcare/causesofdeath/datasets/coronaviruscovid19relateddeathsbyoccupationenglandandwales (accessed 22 Dec 2020).

31 Public Health England. Pilot point prevalence survey of COVID-19 among domiciliary care staff in England. 2020. https://www.gov.uk/government/publications/covid-19-prevalence-survey-domiciliary-care-staff-in-england (accessed 22 Dec 2020).

32 Lyons J, Akbari A, Torabi F, *et al.* Understanding and responding to COVID-19 in Wales: protocol for a privacy-protecting data platform for enhanced epidemiology and evaluation of interventions. *BMJ Open* 2020;**10**:e043010. doi:10.1136/bmjopen-2020-043010

33 Social Care Wales. Why we register. 2022.https://socialcare.wales/registration/why-we-register (accessed 16 Jun 2022).

34 Quinn-Scoggins HD, Cannings-John R, Moriarty Y, *et al.* Cancer symptom experience and help-seeking behaviour during the COVID-19 pandemic in the UK: a cross-sectional population survey. *BMJ Open* 2021;**11**:e053095. doi:10.1136/bmjopen-2021-053095

For peer review only - http://bmjopen.bmj.com/site/about/guidelines.xhtml

35 Aughterson H, McKinlay AR, Fancourt D, *et al.* Psychosocial impact on frontline health and social care professionals in the UK during the COVID-19 pandemic: a qualitative interview study. *BMJ Open* 2021;**11**:e047353. doi:10.1136/bmjopen-2020-047353

36 Markkanen P, Brouillette N, Quinn M, *et al.* "It changed everything": The Safe Home Care qualitative study of the COVID-19 pandemic's impact on home care aides, clients, and managers. *BMC Health Serv Res* 2021;**21**:1055. doi:10.1186/s12913-021-07076-x

37 Health and Safety Executive. Domiciliary care provided in people's own homes. https://www.hse.gov.uk/healthservices/domiciliary-care.htm (accessed 16 Jun 2022).

38 Sterling MR, Li J, Cho J, *et al.* Prevalence and Predictors of Home Health Care Workers' General, Physical, and Mental Health: Findings From the 2014–2018 Behavioral Risk Factor Surveillance System. *Am J Public Health* 2021;**111**:2239–50. doi:10.2105/AJPH.2021.306512

39 Denton MA, Zeytinoğlu IU, Davies S. Working in Clients' Homes: The Impact on the Mental Health and Well-Being of Visiting Home Care Workers. *Home Health Care Serv Q* 2002;**21**:1–27. doi:10.1300/J027v21n01_01

40 Quinn MM, Markkanen PK, Galligan CJ, *et al.* Occupational health of home care aides: results of the safe home care survey. *Occup Environ Med* 2016;**73**:237–45. doi:10.1136/oemed-2015-103031

41 Hayes L TAWH. Care and support workers' perceptions of health and safetyissues in socialcareduring the COVID-19 pandemic. Initial . 2020. https://media.www.kent.ac.uk/se/11148/CareworkersHealthandSafetyreport15042.pdf (accessed 16 Jun 2022).

42 Olson R, Thompson S v., Elliot DL, *et al.* Safety and Health Support for Home Care Workers: The COMPASS Randomized Controlled Trial. *Am J Public Health* 2016;**106**:1823–32. doi:10.2105/AJPH.2016.303327

43 Indregard A-MR, Knardahl S, Emberland JS, *et al.* Effectiveness of the Labour Inspection Authority's regulatory tools for work environment and employee health: study protocol for a cluster-randomised controlled trial among Norwegian home-care workers. *BMJ Open* 2019;**9**:e031226. doi:10.1136/bmjopen-2019-031226

For peer review only

For peer review only - http://bmjopen.bmj.com/site/about/guidelines.xhtml

Figure 1: Cumulative probabilities of confirmed COVID-19 infection over time by employment sector for domiciliary care workers

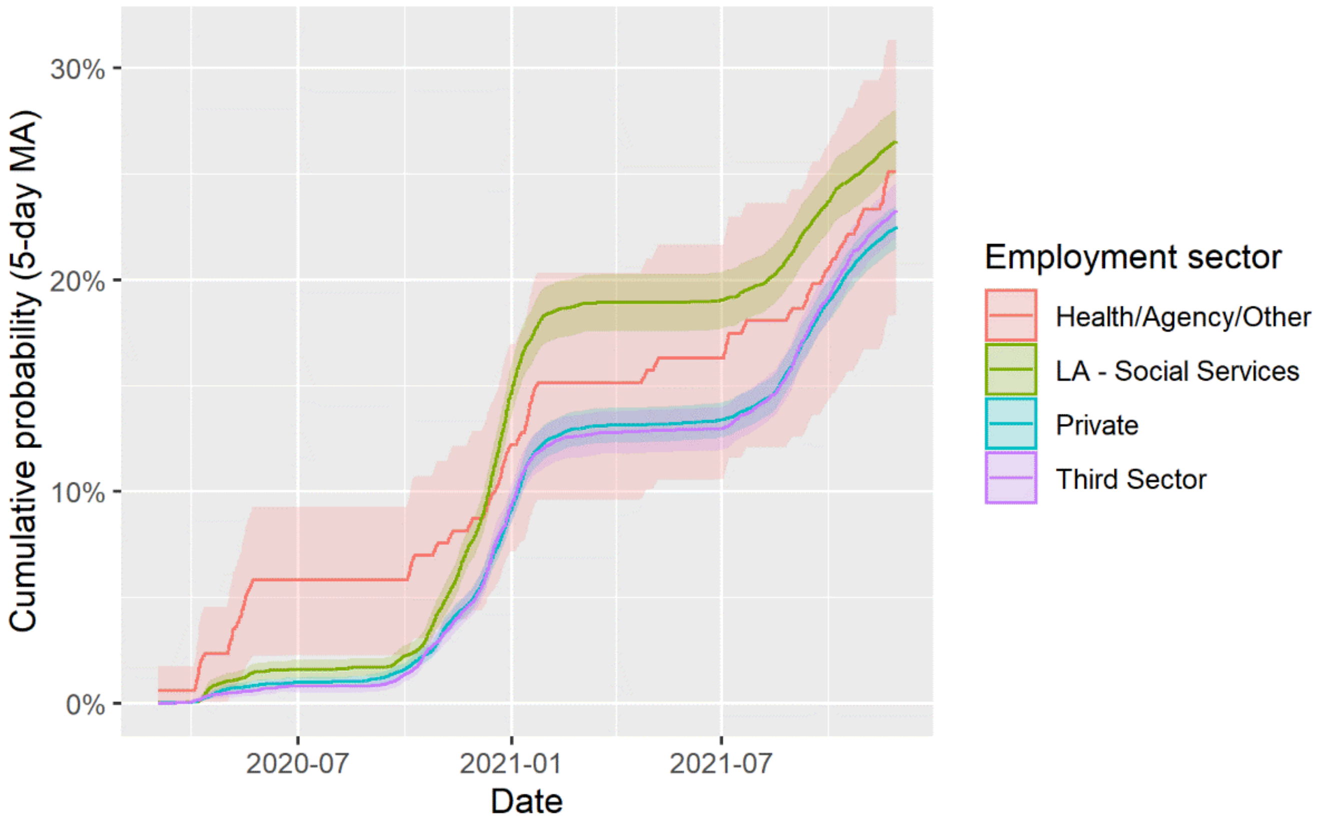

MA=moving average

view only

Figure 2a: Changes over time (March 2016-October 2021) in mental health related hospital admissions for domiciliary care workers

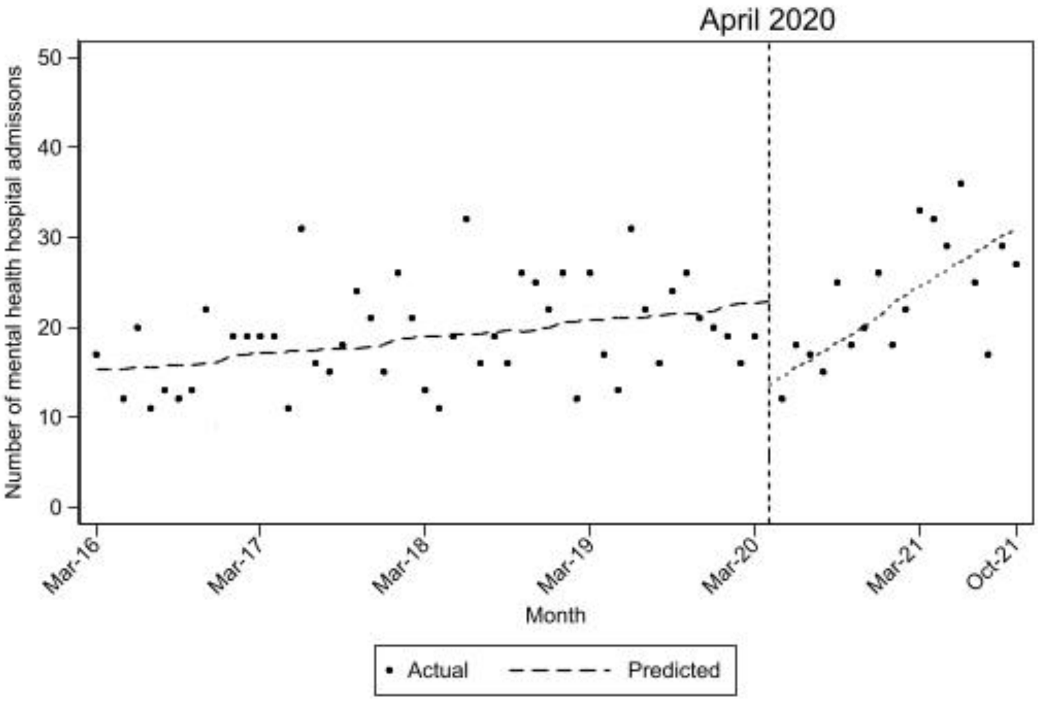

Figure 2b: Changes over time (March 2016-October 2021) in mental health related GP prescriptions for domiciliary care workers

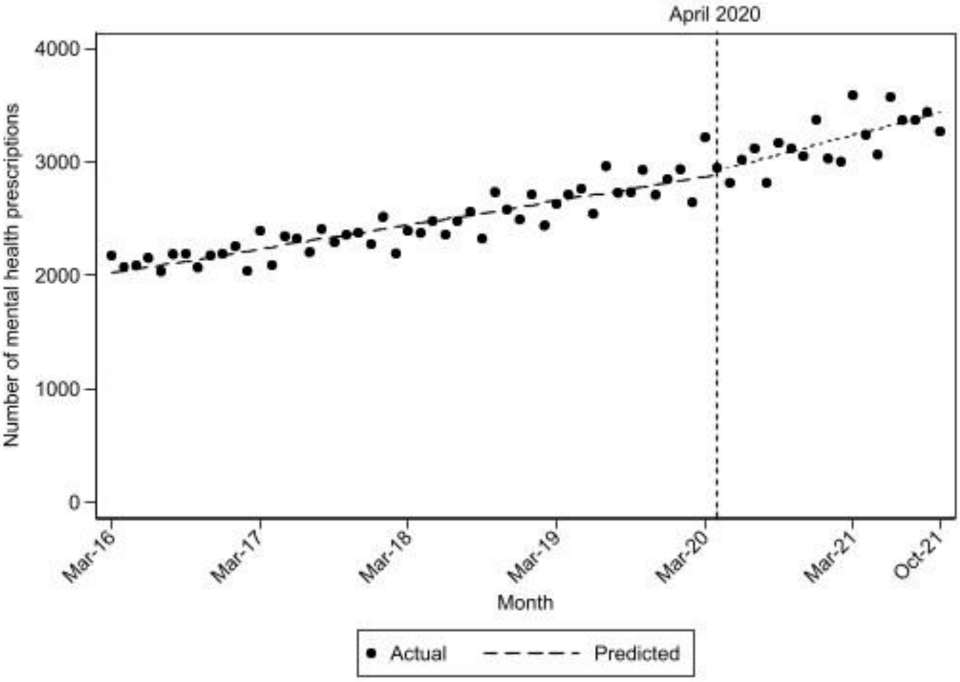

Supplementary material

For peer review only

For peer review only - http://bmjopen.bmj.com/site/about/guidelines.xhtml

Supplementary material 1: Data sources used in the OSCAR study

SAIL acronym	Name	Description	Meta-data link
ADDE	Annual District Death Extract	Monthly register of all deaths relating to Welsh residents, including those that died out of Wales from the Office for National Statistics (ONS).	https://web.www.healthdatagateway.org/dataset/15cf4241-abad-4dcc-95b0-8cd7c02be999
CENW	ONS 2011 Census Wales	A census in the UK is a count of all people and households. The census data held in SAIL was from the census held on 27 March 2011.	https://web.www.healthdatagateway.org/dataset/fc24482c-0f9b-445f-af7a-8598627c3b15
DSCW	Social Care Wales Dataset	Under the Care Standards Act 2000, SCW maintain a register of DCWs in Wales. The DSCW data was transferred securely from SCW to the SAIL Databank and made available as a core-restricted data source.	NA
EDDS	Emergency Department Dataset	Data captured for all NHS Wales Accident and Emergency department attendances. Data are collected and coded at each hospital	https://web.www.healthdatagateway.org/dataset/75c4dcb8-33bf-43f4-b2bb-db51b6621b2c
PATD	COVID-19 Test results	Daily update of all pillar 1 and pillar 2 PCR tests administered and associated results for Welsh residents and those receiving a test in Wales	https://web.www.healthdatagateway.org/dataset/f5f6d882-163d-4ef1-a53e-000fba409480
PEDW	Patient Episode Dataset for Wales	Weekly update of all inpatient and day case activity undertaken in NHS Wales plus data on Welsh residents treated in English Trusts	https://web.www.healthdatagateway.org/dataset/4c33a5d2-164c-41d7-9797-dc2b008cc852
WDSD	Welsh Demographic Service Dataset	Weekly update of all individuals registered with a Welsh general practice, inc. address and practice history	https://web.www.healthdatagateway.org/dataset/8a8a5e90-b0c6-4839-bcd2-c69e6e8dca6d
WLGP	Wales Longitudinal General Practice	Monthly update of all attendance and clinical information for SAIL submitting general practices (~80% national coverage)	https://web.www.healthdatagateway.org/dataset/33fc3ffd-aa4c-4a16-a32f-0c900aaea3d2

For peer review only - http://bmjopen.bmj.com/site/about/guidelines.xhtml

Supplementary material 2: Coding lists for OSCAR outcomes

Primary outcome

Confirmed-COVID: Confirmed COVID-19 will be defined as the earliest of the following events:

- Positive polymerase chain reaction (PCR) test result (Source: PATD);
- Hospital admission (main or secondary diagnostic ICD-10 code of U07.1 position in the admitted patient care component of PEDW);
- Death registration including a diagnosis ICD-10 code U07.1 (as underlying or contributing cause) from COVID-19 (Source: ADDE);
- COVID-19 diagnosis Read code (Source: WLGP).

TYPE	CODE	DESCRIPTION
ICD-10	U071	COVID-19 virus identified
READ	A795.	Coronavirus infection
READ	A7951	Disease caused by 2019-nCoV (novel coronavirus)
READ	A7952	COVID-19 confirmed by laboratory test
READ	A7953	COVID-19 confirmed using clinical diagnostic criteria

Secondary outcomes

Suspected COVID-19

We will ascertain DCWs with a suspected COVID-19 diagnosis taking the earliest of:

- Negative PCR or serology/antigen test from the COVID-19 test data (Source: PATD);
- Diagnosis ICD-10 code (U07.2) main or secondary diagnostic code position (Source: PEDW);
- Suspected COVID-19 Read codes (Source: WLGP).

TYPE	CODE	DESCRIPTION
ICD-10	U072	COVID-19 virus not identified
READ	1JX..	Suspected coronavirus infection
READ	1JX1.	Suspected coronavirus infection

For peer review only - http://bmjopen.bmj.com/site/about/guidelines.xhtml

READ	65PW.	Coronavirus contact
READ	65PW1	Exposure to 2019-nCoV (novel coronavirus) infection

Mental Health

(A) Hospital admissions (ICD-10)

CODE	DESCRIPTION
F20	Schizophrenia
F200	Paranoid schizophrenia
F201	Hebephrenic schizophrenia
F202	Catatonic schizophrenia
F203	Undifferentiated schizophrenia
F204	Post-schizophrenic depression
F205	Residual schizophrenia
F206	Simple schizophrenia
F208	Other schizophrenia
F209	Schizophrenia, unspecified
F21X	Schizotypal disorder
F22	Persistent delusional disorders
F220	Delusional disorder
F228	Other persistent delusional disorders
F229	Persistent delusional disorder, unspecified
F23	Acute and transient psychotic disorders
F230	Acute polymorphic psychotic disorder without symptoms of schizophrenia
F231	Acute polymorphic psychotic disorder with symptoms of schizophrenia
F232	Acute schizophrenia-like psychotic disorder
F233	Other acute predominantly delusional psychotic disorders
F238	Other acute and transient psychotic disorders
F239	Acute and transient psychotic disorder, unspecified
F24X	Induced delusional disorder

For peer review only

For peer review only - http://bmjopen.bmj.com/site/about/guidelines.xhtml

CODE	DESCRIPTION
F25	Schizoaffective disorders
F250	Schizoaffective disorder, manic type
F251	Schizoaffective disorder, depressive type
F252	Schizoaffective disorder, mixed type
F258	Other schizoaffective disorders
F259	Schizoaffective disorder, unspecified
F28X	Other nonorganic psychotic disorders
F29X	Unspecified nonorganic psychosis
F30	Manic episode
F300	Hypomania
F301	Mania without psychotic symptoms
F302	Mania with psychotic symptoms
F308	Other manic episodes
F309	Manic episode, unspecified
F31	Bipolar affective disorder
F310	Bipolar affective disorder, current episode hypomanic
F311	Bipolar affective disorder, current episode manic without psychotic symptoms
F312	Bipolar affective disorder, current episode manic with psychotic symptoms
F313	Bipolar affective disorder, current episode mild or moderate depression
F314	Bipolar affective disorder, current episode severe depression without psychotic symptoms
F315	Bipolar affective disorder, current episode severe depression with psychotic symptoms
F316	Bipolar affective disorder, current episode mixed
F317	Bipolar affective disorder, currently in remission
F318	Other bipolar affective disorders
F319	Bipolar affective disorder, unspecified
F32	Depressive episode
F320	Mild depressive episode

For peer review only - http://bmjopen.bmj.com/site/about/guidelines.xhtml

CODE	DESCRIPTION
F321	Moderate depressive episode
F322	Severe depressive episode without psychotic symptoms
F323	Severe depressive episode with psychotic symptoms
F328	Other depressive episodes
F329	Depressive episode, unspecified
F33	Recurrent depressive disorder
F330	Recurrent depressive disorder, current episode mild
F331	Recurrent depressive disorder, current episode moderate
F332	Recurrent depressive disorder, current episode severe without psychotic symptoms
F333	Recurrent depressive disorder, current episode severe with psychotic symptoms
F334	Recurrent depressive disorder, currently in remission
F338	Other recurrent depressive disorders
F339	Recurrent depressive disorder, unspecified

(B) GP contacts and prescriptions (Read)

CODE	DESCRIPTION
146D.	H/O: manic depressive disorder
1B12.	Nerves - nervousness
1B13.	Anxiousness
1B17.	Depressed
1B1U.	Symptoms of depression
1BH3.	Paranoid ideation
1BQ..	Loss of capacity for enjoyment
1BT..	Depressed mood
1BU..	Loss of hope for the future
2257	O/E - depressed
2258	O/E - anxious

For peer review only - http://bmjopen.bmj.com/site/about/guidelines.xhtml

CODE	DESCRIPTION
2259	O/E - nervous
66580	Antidepressant drug treatment changed
66590	Antidepressant drug treatment started
665A0	Antidepressant drug treatment stopped
d1...	HYPNOTICS
d11..	CHLORAL HYDRATE
d111.	*CHLORAL 500mg/5mL mixture
d112.	CHLORAL PAEDIATRIC 200mg/5mL elixir
d113.	*NOCTEC 500mg capsules
d114.	CHLORAL HYDRATE 500mg capsules
d115.	*WELLDORM 143mg/5mL elixir
d116.	*WELLDORM tablets
d117.	CHLORAL HYDRATE 414mg tablets
d118.	CHLORAL HYDRATE 143mg/5mL elixir
d119.	CHLORAL HYDRATE 500mg/5mL syrup
d11A.	SOMNWELL 707mg tablets
d12..	CLOMETHIAZOLE EDISYLATE [HYPNOTIC]
d121.	*HEMINEVRIN 192mg capsules
d122.	*HEMINEVRIN 250mg/5mL syrup
d123.	*HEMINEVRIN 8mg/mL infusion
d12v.	CLOMETHIAZOLE(base) 192mg capsules
d12w.	CLOMETHIAZOLE EDISILATE 250mg/5mL syrup
d12z.	CHLORMETHIAZOLE EDISYLATE 8mg/mL infusion
d13..	*DICHLORALPHENAZONE
d131.	*WELLDORM 650mg tablets
d132.	*WELLDORM 225mg/5mL elixir
d13y.	DICHLORALPHENAZONE 650mg tablets
d13z.	DICHLORALPHENAZONE 225mg/5mL elixir
d14..	*FLUNITRAZEPAM

For peer review only - http://bmjopen.bmj.com/site/about/guidelines.xhtml

CODE	DESCRIPTION
d141.	*ROHYPNOL 1mg tablets
d14z.	*FLUNITRAZEPAM 1mg tablets
d15..	FLURAZEPAM
d151.	DALMANE 15mg capsules
d152.	DALMANE 30mg capsules
d153.	*PAXANE 15mg capsules
d154.	*PAXANE 30mg capsules
d15y.	FLURAZEPAM 15mg capsules
d15z.	FLURAZEPAM 30mg capsules
d16..	LOPRAZOLAM
d161.	LOPRAZOLAM 1mg tablets
d162.	*DORMONOCT 1mg tablets
d17..	LORMETAZEPAM
d171.	LORMETAZEPAM 500micrograms tablets
d172.	LORMETAZEPAM 1mg tablets
d173.	NOCTAMID 500micrograms tablets
d174.	*NOCTAMID 1mg tablets
d18..	NITRAZEPAM
d181.	*NITRAZEPAM 5mg capsules
d182.	NITRAZEPAM 5mg tablets
d183.	*NITRAZEPAM 10mg tablets
d184.	NITRAZEPAM 2.5mg/5mL mixture
d185.	*MOGADON 5mg capsules
d186.	MOGADON 5mg tablets
d187.	*NITRADOS 5mg tablets
d188.	*NOCTESED 5mg tablets
d189.	REMNOS 5mg tablets
d18a.	*REMNOS 10mg tablets
d18b.	*SOMNITE 5mg tablets

For peer review only

For peer review only - http://bmjopen.bmj.com/site/about/guidelines.xhtml

CODE	DESCRIPTION
d18c.	*SOMNITE 2.5mg/5mL mixture
d18d.	*SUREM 5mg capsules
d18e.	*UNISOMNIA 5mg tablets
d18f.	NITRAZEPAM 5mg/5mL suspension
d19..	PROMETHAZINE HCL [HYPNOTIC] see section c8i..
d1a..	TEMAZEPAM [HYPNOTIC]
d1a1.	*TEMAZEPAM 10mg capsules
d1a2.	*TEMAZEPAM 15mg capsules
d1a3.	*TEMAZEPAM 20mg capsules
d1a4.	*TEMAZEPAM 30mg capsules
d1a5.	*TEMAZEPAM 10mg/5mL elixir
d1a6.	*NORMISON 10mg capsules
d1a7.	*NORMISON 20mg capsules
d1a8.	*TEMAZEPAM PLANPAK capsules
d1a9.	TEMAZEPAM 10mg tablets
d1aa.	TEMAZEPAM 20mg tablets
d1ab.	TEMAZEPAM GELTHIX 10mg capsules
d1ac.	TEMAZEPAM GELTHIX 20mg capsules
d1ad.	TEMAZEPAM GELTHIX 15mg capsules
d1ae.	TEMAZEPAM GELTHIX 30mg capsules
d1af.	TEMAZEPAM GEL FILLED 10mg capsules
d1ag.	TEMAZEPAM GEL FILLED 20mg capsules
d1ah.	TEMAZEPAM GEL FILLED 15mg capsules
d1ai.	TEMAZEPAM GEL FILLED 30mg capsules
d1aj.	TEMAZEPAM 10mg/sachet oral solution
d1ak.	TEMAZEPAM 20mg/sachet oral solution
d1al.	EUHYPNOS 10mg/sachet oral solution
d1am.	EUHYPNOS 20mg/sachet oral solution
d1an.	EUHYPNOS 10mg/5mL oral solution

For peer review only

For peer review only - http://bmjopen.bmj.com/site/about/guidelines.xhtml

CODE	DESCRIPTION
d1ao.	TEMAZEPAM 10mg/5mL sugar free oral solution
d1b..	*TRIAZOLAM
d1b1.	TRIAZOLAM 125microgram tablets
d1b2.	TRIAZOLAM 250microgram tablets
d1b3.	*HALCION 125microgram tablets
d1b4.	*HALCION 250microgram tablets
d1c..	TRICLOFOS SODIUM
d1c1.	*TRICLOFOS 500mg/5mL liquid
d1d..	ZOPICLONE
d1d1.	ZOPICLONE 7.5mg tablets
d1d2.	ZIMOVANE 7.5mg tablets
d1d3.	ZOPICLONE 3.75mg tablets
d1d4.	ZIMOVANE LS 3.75mg tablets
d1d5.	*ZILEZE 7.5 tablets
d1d6.	*ZILEZE 3.75 tablets
d1e..	*GLUTETHIMIDE [no drugs here]
d1f..	ZOLPIDEM
d1f1.	STILNOCT 5mg tablets
d1f2.	ZOLPIDEM TARTRATE 5mg tablets
d1f3.	ZOLPIDEM TARTRATE 10mg tablets
d1f4.	STILNOCT 10mg tablets
d1g..	ZALEPLON
d1g1.	SONATA 5mg capsules
d1g2.	SONATA 10mg capsules
d1gy.	ZALEPLON 5mg capsules
d1gz.	ZALEPLON 10mg capsules
d1h..	MELATONIN
d1h1.	CIRCADIN 2mg m/r tablets
d1hz.	MELATONIN 2mg m/r tablets

For peer review only - http://bmjopen.bmj.com/site/about/guidelines.xhtml

For peer review only

CODE	DESCRIPTION
d21..	DIAZEPAM [ANXIOLYTIC]
d22..	ALPRAZOLAM
d23..	BROMAZEPAM
d24..	CHLORDIAZEPOXIDE
d25..	CHLORMEZANONE
d26..	CLOBAZAM
d27..	CLORAZEPATE DIPOTASSIUM
d28..	HYDROXYZINE HCL [ANXIOLYTIC]
d29..	*KETAZOLAM
d2a..	LORAZEPAM [ANXIOLYTIC]
d2b..	*MEDAZEPAM
d2c..	MEPROBAMATE
d2d..	OXAZEPAM
d2f..	BUSPIRONE HYDROCHLORIDE
d2g..	FLUMAZENIL
d7...	TRICYCLIC ANTIDEPRESSANTS
d71..	AMITRIPTYLINE HYDROCHLORIDE [ANTIDEPRESSANT]
d711.	AMITRIPTYLINE 10mg tablets
d712.	AMITRIPTYLINE 25mg tablets
d713.	AMITRIPTYLINE 50mg tablets
d714.	*DOMICAL 10mg tablets
d715.	*DOMICAL 25mg tablets
d716.	*DOMICAL 50mg tablets
d717.	ELAVIL 10mg tablets
d718.	ELAVIL 25mg tablets
d719.	*LENTIZOL 25mg m/r capsules
d71a.	*LENTIZOL 50mg m/r capsules
d71b.	*TRYPTIZOL 75mg m/r capsules
d71c.	*TRYPTIZOL 10mg tablets

For peer review only - http://bmjopen.bmj.com/site/about/guidelines.xhtml

CODE	DESCRIPTION
d71d.	*TRYPTIZOL 25mg tablets
d71e.	*TRYPTIZOL 50mg tablets
d71f.	*TRYPTIZOL 10mg/5mL syrup
d71g.	TRYPTIZOL 100mg/10mL injection
d71h.	AMITRIPTYLINE 25mg/5mL sugar free solution
d71i.	AMITRIPTYLINE 50mg/5mL sugar free solution
d71u.	AMITRIPTYLINE 25mg m/r capsules
d71v.	AMITRIPTYLINE 50mg m/r capsules
d71w.	AMITRIPTYLINE 75mg m/r capsules
d71y.	*AMITRIPTYLINE 10mg/5mL syrup
d71z.	AMITRIPTYLINE HYDROCHLORIDE 100mg/10mL injection
d72..	*BUTRIPTYLINE
d721.	*EVADYNE 25mg tablets
d722.	*EVADYNE 50mg tablets
d72y.	*BUTRIPTYLINE 25mg tablets
d72z.	*BUTRIPTYLINE 50mg tablets
d73..	CLOMIPRAMINE HYDROCHLORIDE
d731.	*ANAFRANIL 10mg capsules
d732.	*ANAFRANIL 25mg capsules
d733.	*ANAFRANIL 50mg capsules
d734.	*ANAFRANIL 25mg/5mL syrup
d735.	*ANAFRANIL 25mg/2mL injection
d736.	*ANAFRANIL SR 75mg m/r tablets
d737.	*TRANQUAX 10mg capsules
d738.	*TRANQUAX 25mg capsules
d739.	TRANQUAX 50mg capsules
d73r.	*CLOMIPRAMINE HCL 50mg tablets
d73s.	*CLOMIPRAMINE HCL 10mg tablets
d73t.	*CLOMIPRAMINE HCL 25mg tablets

For peer review only - http://bmjopen.bmj.com/site/about/guidelines.xhtml

For peer review only

CODE	DESCRIPTION
d73u.	CLOMIPRAMINE HCL 10mg capsules
d73v.	CLOMIPRAMINE HCL 25mg capsules
d73w.	CLOMIPRAMINE HCL 50mg capsules
d73x.	CLOMIPRAMINE HYDROCHLORIDE 25mg/5mL syrup
d73y.	CLOMIPRAMINE HCL 25mg/2mL injection
d73z.	CLOMIPRAMINE HCL 75mg m/r tabs
d74..	DESIPRAMINE HYDROCHLORIDE
d741.	*PERTOFRAN 25mg tablets
d74z.	DESIPRAMINE HYDROCHLORIDE 25mg tablets
d75..	DOSULEPIN HYDROCHLORIDE
d751.	PROTHIADEN 25mg capsules
d752.	PROTHIADEN 75mg tablets
d753.	*PREPADINE 25mg capsules
d754.	*PREPADINE 75mg tablets
d755.	*DOTHAPAX 25mg capsules
d756.	*DOTHAPAX 75mg tablets
d759.	*THADEN 25mg capsules
d75A.	*THADEN 75mg tablets
d75y.	DOSULEPIN HYDROCHLORIDE 25mg capsules
d75z.	DOSULEPIN HYDROCHLORIDE 75mg tablets
d76..	DOXEPIN
d761.	*SINEQUAN 10mg capsules x56CP
d762.	*SINEQUAN 25mg capsules x28CP
d763.	*SINEQUAN 50mg capsules x28CP
d764.	*SINEQUAN 75mg capsules x28CP
d765.	*SINEPIN 25mg capsules
d766.	*SINEPIN 50mg capsules
d76w.	*DOXEPIN 10mg capsules
d76x.	DOXEPIN 25mg capsules

For peer review only - http://bmjopen.bmj.com/site/about/guidelines.xhtml

CODE	DESCRIPTION
d76y.	DOXEPIN 50mg capsules
d76z.	*DOXEPIN 75mg capsules
d77..	IMIPRAMINE HYDROCHLORIDE [ANTIDEPRESSANT]
d771.	IMIPRAMINE 10mg tabs
d772.	IMIPRAMINE 25mg tablets
d773.	*PRAMINIL 10mg tablets
d774.	*PRAMINIL 25mg tablets
d775.	*TOFRANIL 10mg tablets
d776.	*TOFRANIL 25mg tablets
d777.	*TOFRANIL 25mg/5mL syrup
d77w.	IMIPRAMINE HYDROCHLORIDE 25mg/5mL oral solution
d77x.	IMIPRAMINE HYDROCHLORIDE 10mg tablets
d77y.	IMIPRAMINE HYDROCHLORIDE 25mg tablets
d77z.	IMIPRAMINE HYDROCHLORIDE 25mg/5mL syrup
d78..	IPRINDOLE
d781.	*PRONDOL 15mg tablets
d782.	*PRONDOL 30mg tablets
d78y.	*IPRINDOLE 15mg tablets
d78z.	*IPRINDOLE 30mg tablets
d79..	LOFEPRAMINE
d791.	*GAMANIL 70mg tablets x56CP
d792.	LOMONT 70mg/5mL sugar free suspension
d793.	*FEPRAPAX 70mg tablets
d794.	*GAMANIL 70mg tablets
d79y.	LOFEPRAMINE 70mg/5mL sugar free suspension
d79z.	LOFEPRAMINE 70mg tablets
d7a..	MAPROTILINE HYDROCHLORIDE
d7a1.	*LUDIOMIL 10mg tablets
d7a2.	*LUDIOMIL 25mg tablets

For peer review only

For peer review only - http://bmjopen.bmj.com/site/about/guidelines.xhtml

CODE	DESCRIPTION
d7a3.	*LUDIOMIL 50mg tablets
d7a4.	*LUDIOMIL 75mg tablets x28CP
d7aw.	MAPROTILINE HYDROCHLORIDE 10mg tablets
d7ax.	MAPROTILINE HYDROCHLORIDE 25mg tablets
d7ay.	MAPROTILINE HYDROCHLORIDE 50mg tablets
d7az.	MAPROTILINE HYDROCHLORIDE 75mg tablets
d7b..	MIANSERIN HYDROCHLORIDE
d7b1.	MIANSERIN 10mg tablets
d7b2.	MIANSERIN 20mg tablets
d7b3.	MIANSERIN 30mg tablets
d7b4.	*BOLVIDON 10mg tablets
d7b5.	*BOLVIDON 20mg tablets
d7b6.	*BOLVIDON 30mg tablets
d7b7.	*NORVAL 10mg tablets
d7b8.	*NORVAL 20mg tablets
d7b9.	*NORVAL 30mg tablets
d7c..	NORTRIPTYLINE
d7c1.	*ALLEGRON 10mg tablets
d7c2.	*ALLEGRON 25mg tablets
d7c3.	*AVENTYL 10mg capsules
d7c4.	*AVENTYL 25mg capsules
d7c5.	*AVENTYL 10mg/5mL liquid
d7c6.	NORTRIPTYLINE 10mg tablets
d7c7.	*NORTRIPTYLINE 10mg/5mL liquid
d7c8.	NORTRIPTYLINE 25mg tablets
d7c9.	*NORTRIPTYLINE 10mg capsules
d7cy.	*NORTRIPTYLINE 25mg capsules
d7d..	PROTRIPTYLINE HYDROCHLORIDE
d7d1.	*CONCORDIN 5mg tablets

For peer review only

For peer review only - http://bmjopen.bmj.com/site/about/guidelines.xhtml

CODE	DESCRIPTION
d7d2.	*CONCORDIN 10mg tablets
d7d3.	PROTRIPTYLINE HYDROCHLORIDE 5mg tablets
d7d4.	PROTRIPTYLINE HYDROCHLORIDE 10mg tablets
d7e..	TRAZODONE HYDROCHLORIDE
d7e1.	MOLIPAXIN 50mg capsules x84CP
d7e2.	MOLIPAXIN 100mg capsules x56CP
d7e3.	*MOLIPAXIN 50mg/5mL liquid
d7e4.	MOLIPAXIN 150mg tablets x28CP
d7e5.	TRAZODONE HYDROCHLORIDE 150mg tablets
d7e6.	MOLIPAXIN CR 150mg m/r tablets x28
d7e7.	TRAZODONE HYDROCHLORIDE 150mg m/r tablets
d7ew.	TRAZODONE HYDROCHLORIDE 100mg capsules
d7ex.	TRAZODONE HYDROCHLORIDE 50mg capsules
d7ez.	TRAZODONE HYDROCHLORIDE 50mg/5mL liquid
d7f..	TRIMIPRAMINE
d7f1.	*SURMONTIL 50mg capsules x28CP
d7f2.	*SURMONTIL 10mg tablets
d7f3.	*SURMONTIL 25mg tablets
d7fx.	TRIMIPRAMINE 50mg capsules
d7fy.	TRIMIPRAMINE 10mg tablets
d7fz.	TRIMIPRAMINE 25mg tablets
d7g..	VILOXAZINE HYDROCHLORIDE
d7g1.	*VIVALAN 50mg tablets
d7gz.	*VILOXAZINE 50mg tablets
d7h..	AMOXAPINE
d7h1.	*AMOXAPINE 25mg tablets
d7h2.	*AMOXAPINE 50mg tablets
d7h3.	*AMOXAPINE 100mg tablets
d7h4.	*AMOXAPINE 150mg tablets

For peer review only - http://bmjopen.bmj.com/site/about/guidelines.xhtml

CODE	DESCRIPTION
d7h5.	*ASENDIS 25mg tablets
d7h6.	*ASENDIS 50mg tablets
d7h7.	*ASENDIS 100mg tablets
d7h8.	*ASENDIS 150mg tablets
d9...	COMPOUND ANTIDEPRESSANT DRUGS
d91..	COMPOUND ANTIDEPRESSANTS A-Z
d911.	*LIMBITROL 5 capsules
d912.	*LIMBITROL 10 capsules
d913.	*MOTIPRESS tablets x28CP
d914.	*MOTIVAL tablets
d915.	*PARSTELIN tablets
d916.	TRIPTAFEN tablets
d917.	*TRIPTAFEN-M tablets
da...	OTHER ANTIDEPRESSANT DRUGS
da1..	FLUPENTIXOL [ANTIDEPRESSANT]
da11.	FLUANXOL 500micrograms tablets
da12.	FLUANXOL 1mg tablets
da1y.	FLUPENTIXOL 500micrograms tablets
da1z.	FLUPENTIXOL 1mg tablets
da2..	TRYPTOPHAN
da21.	*OPTIMAX 500mg tablets
da22.	*OPTIMAX 1g/6g powder
da23.	*OPTIMAX WV 500mg tablets
da24.	*PACITRON 500mg tablets
da2y.	*TRYPTOPHAN 500mg tablets
da2z.	*TRYPTOPHAN 1g/6g powder
da3..	FLUVOXAMINE MALEATE
da31.	FAVERIN 50mg tablets
da32.	FLUVOXAMINE MALEATE 50mg tablets

For peer review only - http://bmjopen.bmj.com/site/about/guidelines.xhtml

CODE	DESCRIPTION
da33.	FAVERIN 100mg tablets
da34.	FLUVOXAMINE MALEATE 100mg tablets
da4..	FLUOXETINE HYDROCHLORIDE
da41.	FLUOXETINE 20mg capsules
da42.	*PROZAC 20mg capsules x30
da43.	FLUOXETINE 20mg/5mL oral liquid
da44.	PROZAC 20mg/5mL oral liquid
da45.	PROZAC 20mg capsules
da46.	FLUOXETINE 60mg capsules
da47.	*PROZAC 60mg capsules
da48.	*FELICIUM 20mg capsules
da49.	OXACTIN 20mg capsules
da4A.	RANFLUTIN 20mg capsules
da4B.	PROZIT 20mg/5mL oral solution
da4C.	PROZEP 20mg/5mL oral solution
da5..	SERTRALINE HYDROCHLORIDE
da51.	SERTRALINE 50mg tablets
da52.	SERTRALINE 100mg tablets
da53.	LUSTRAL 50mg tablets
da54.	LUSTRAL 100mg tablets
da6..	PAROXETINE HYDROCHLORIDE
da61.	PAROXETINE 20mg tablets
da62.	SEROXAT 20mg tablets x30
da63.	PAROXETINE 30mg tablets
da64.	SEROXAT 30mg tablets x30
da65.	PAROXETINE 10mg/5mL sugar free liquid
da66.	SEROXAT 10mg/5mL sugar free liquid
da67.	PAROXETINE 10mg tablets
da68.	SEROXAT 10mg tablets

For peer review only - http://bmjopen.bmj.com/site/about/guidelines.xhtml

CODE	DESCRIPTION
da7..	VENLAFAXINE
da71.	VENLAFAXINE 37.5mg tablets
da72.	VENLAFAXINE 75mg tablets
da73.	*EFEXOR 37.5mg tablets
da74.	*EFEXOR 75mg tablets
da75.	*VENLAFAXINE 50mg tablets
da76.	*EFEXOR 50mg tablets
da77.	VENLAFAXINE 75mg m/r capsules
da78.	EFEXOR XL 75mg m/r capsules
da79.	VENLAFAXINE 150mg m/r capsules
da7a.	VENAXX XL 150mg m/r capsules
da7A.	EFEXOR XL 150mg m/r capsules
da7b.	VAXALIN XL 75mg m/r capsules
da7B.	RODOMEL XL 75mg m/r capsules
da7c.	VAXALIN XL 150mg m/r capsules
da7C.	RODOMEL XL 150mg m/r capsules
da7d.	ALVENTA XL 75mg m/r capsules
da7D.	WINFEX XL 75mg m/r capsules
da7e.	ALVENTA XL 150mg m/r capsules
da7E.	WINFEX XL 150mg m/r capsules
da7f.	RANFAXINE XL 150mg m/r capsules
da7F.	TRIXAT XL 75mg m/r capsules
da7g.	RANFAXINE XL 75mg m/r capsules
da7G.	TRIXAT XL 150mg m/r capsules
da7h.	BONILUX XL 75mg m/r capsules
da7H.	VIEPAX XL 75mg m/r tablets
da7i.	BONILUX XL 150mg m/r capsules
da7I.	VENLAFAXINE 75mg m/r tablets
da7j.	TONPULAR XL 75mg m/r capsules

For peer review only

For peer review only - http://bmjopen.bmj.com/site/about/guidelines.xhtml

CODE	DESCRIPTION
da7J.	VIEPAX XL 150mg m/r tablets
da7k.	TONPULAR XL 150mg m/r capsules
da7K.	VENLAFAXINE 150mg m/r tablets
da7l.	FORAVEN XL 75mg m/r capsules
da7L.	*TARDCAPS XL 75mg m/r capsules
da7m.	FORAVEN XL 150mg m/r capsules
da7M.	*TARDCAPS XL 150mg m/r capsule
da7n.	DEPEFEX XL 75mg m/r capsules
da7N.	VIEPAX 37.5mg tablets
da7o.	DEPEFEX XL 150mg m/r capsules
da7O.	VIEPAX 75mg tablets
da7p.	VENLALIC XL 37.5mg m/r tablets
da7P.	VENSIR XL 75mg m/r capsules
da7q.	VENLAFAXINE 37.5mg m/r tablets
da7Q.	VENSIR XL 150mg m/r capsules
da7R.	TIFAXIN XL 75mg m/r capsules
da7S.	TIFAXIN XL 150mg m/r capsules
da7T.	VEXARIN XL 75mg m/r capsules
da7U.	VEXARIN XL 150mg m/r capsules
da7V.	VENLALIC XL 75mg m/r tablets
da7W.	VENLALIC XL 150mg m/r tablets
da7X.	VENLALIC XL 225mg m/r tablets
da7Y.	VENLAFAXINE 225mg m/r tablets
da7Z.	VENAXX XL 75mg m/r capsules
da8..	NEFAZODONE
da81.	NEFAZODONE HYDROCHLORIDE 100mg tablets
da82.	NEFAZODONE HYDROCHLORIDE 200mg tablets
da83.	*DUTONIN 100mg tablets
da84.	*DUTONIN 200mg tablets

For peer review only - http://bmjopen.bmj.com/site/about/guidelines.xhtml

CODE	DESCRIPTION
da85.	NEFAZODONE HCL 50mg+100mg+200mg initiation tablets pack
da86.	DUTONIN 50mg+100mg+200mg treatment initiation tablets pack
da9..	CITALOPRAM
da91.	CITALOPRAM 20mg tablets
da92.	CIPRAMIL 20mg tablets
da93.	CITALOPRAM 10mg tablets
da94.	*CIPRAMIL 10mg tablets
da95.	CITALOPRAM 40mg tablets
da96.	*CIPRAMIL 40mg tablets
da97.	CIPRAMIL 40mg/mL oral drops 15mL
da98.	*PAXORAN 10mg tablets
da99.	*PAXORAN 20mg tablets
da9A.	*PAXORAN 40mg tablets
da9z.	CITALOPRAM 40mg/mL oral drops
daA..	REBOXETINE
daA1.	REBOXETINE 4mg tablets
daA2.	EDRONAX 4mg tablets
daB..	MIRTAZAPINE
daB1.	MIRTAZAPINE 30mg tablets
daB2.	*ZISPIN 30mg tablets
daB3.	MIRTAZAPINE 30mg oro-dispersible tablets
daB4.	ZISPIN SOLTAB 30mg oro-dispersible tablets
daB5.	MIRTAZAPINE 15mg oro-dispersible tablets
daB6.	ZISPIN SOLTAB 15mg oro-dispersible tablets
daB7.	MIRTAZAPINE 45mg oro-dispersible tablets
daB8.	ZISPIN SOLTAB 45mg oro-dispersible tablets
daBy.	MIRTAZAPINE 45mg tablets
daBz.	MIRTAZAPINE 15mg tablets
daC..	ESCITALOPRAM

For peer review only - http://bmjopen.bmj.com/site/about/guidelines.xhtml

CODE	DESCRIPTION
daC1.	ESCITALOPRAM 10mg tablets
daC2.	CIPRALEX 10mg tablets
daC3.	ESCITALOPRAM 20mg tablets
daC4.	CIPRALEX 20mg tablets
daC5.	ESCITALOPRAM 5mg tablets
daC6.	CIPRALEX 5mg tablets
daC7.	ESCITALOPRAM 10mg/mL oral drops
daC8.	*CIPRALEX 10mg/mL oral drops
daC9.	CIPRALEX 20mg/mL oral drops
daCA.	ESCITALOPRAM 20mg/mL oral drops
daD..	AGOMELATINE
daD1.	VALDOXAN 25mg tablets
daD2.	AGOMELATINE 25mg tablets
E1...	Non-organic psychoses
E10..	Schizophrenic disorders
E100.	Simple schizophrenia
E1000	Unspecified schizophrenia
E1001	Subchronic schizophrenia
E1002	Chronic schizophrenic
E1003	Acute exacerbation of subchronic schizophrenia
E1004	Acute exacerbation of chronic schizophrenia
E1005	Schizophrenia in remission
E100z	Simple schizophrenia NOS
E101.	Hebephrenic schizophrenia
E1010	Unspecified hebephrenic schizophrenia
E1011	Subchronic hebephrenic schizophrenia
E1012	Chronic hebephrenic schizophrenia
E1013	Acute exacerbation of subchronic hebephrenic schizophrenia
E1014	Acute exacerbation of chronic hebephrenic schizophrenia

For peer review only - http://bmjopen.bmj.com/site/about/guidelines.xhtml

CODE	DESCRIPTION
E1015	Hebephrenic schizophrenia in remission
E101z	Hebephrenic schizophrenia NOS
E102.	Catatonic schizophrenia
E1020	Unspecified catatonic schizophrenia
E1021	Subchronic catatonic schizophrenia
E1022	Chronic catatonic schizophrenia
E1023	Acute exacerbation of subchronic catatonic schizophrenia
E1024	Acute exacerbation of chronic catatonic schizophrenia
E1025	Catatonic schizophrenia in remission
E102z	Catatonic schizophrenia NOS
E103.	Paranoid schizophrenia
E1030	Unspecified paranoid schizophrenia
E1031	Subchronic paranoid schizophrenia
E1032	Chronic paranoid schizophrenia
E1033	Acute exacerbation of subchronic paranoid schizophrenia
E1034	Acute exacerbation of chronic paranoid schizophrenia
E1035	Paranoid schizophrenia in remission
E103z	Paranoid schizophrenia NOS
E104.	Acute schizophrenic episode
E105.	Latent schizophrenia
E1050	Unspecified latent schizophrenia
E1051	Subchronic latent schizophrenia
E1052	Chronic latent schizophrenia
E1053	Acute exacerbation of subchronic latent schizophrenia
E1054	Acute exacerbation of chronic latent schizophrenia
E1055	Latent schizophrenia in remission
E105z	Latent schizophrenia NOS
E106.	Residual schizophrenia
E107.	Schizo-affective schizophrenia

For peer review only - http://bmjopen.bmj.com/site/about/guidelines.xhtml

CODE	DESCRIPTION
E1070	Unspecified schizo-affective schizophrenia
E1071	Subchronic schizo-affective schizophrenia
E1072	Chronic schizo-affective schizophrenia
E1073	Acute exacerbation subchronic schizo-affective schizophrenia
E1074	Acute exacerbation of chronic schizo-affective schizophrenia
E1075	Schizo-affective schizophrenia in remission
E107z	Schizo-affective schizophrenia NOS
E10y.	Other schizophrenia
E10y0	Atypical schizophrenia
E10y1	Coenesthopathic schizophrenia
E10yz	Other schizophrenia NOS
E10z.	Schizophrenia NOS
E11..	Affective psychoses
E110.	Manic disorder, single episode
E1100	Single manic episode, unspecified
E1101	Single manic episode, mild
E1102	Single manic episode, moderate
E1103	Single manic episode, severe without mention of psychosis
E1104	Single manic episode, severe, with psychosis
E1105	Single manic episode in partial or unspecified remission
E1106	Single manic episode in full remission
E110z	Manic disorder, single episode NOS
E111.	Recurrent manic episodes
E1110	Recurrent manic episodes, unspecified
E1111	Recurrent manic episodes, mild
E1112	Recurrent manic episodes, moderate
E1113	Recurrent manic episodes, severe without mention psychosis
E1114	Recurrent manic episodes, severe, with psychosis
E1115	Recurrent manic episodes, partial or unspecified remission

For peer review only - http://bmjopen.bmj.com/site/about/guidelines.xhtml

CODE	DESCRIPTION
E1116	Recurrent manic episodes, in full remission
E111z	Recurrent manic episode NOS
E112.	Single major depressive episode
E1120	Single major depressive episode, unspecified
E1121	Single major depressive episode, mild
E1122	Single major depressive episode, moderate
E1123	Single major depressive episode, severe, without psychosis
E1124	Single major depressive episode, severe, with psychosis
E1125	Single major depressive episode, partial or unspec remission
E1126	Single major depressive episode, in full remission
E112z	Single major depressive episode NOS
E113.	Recurrent major depressive episode
E1130	Recurrent major depressive episodes, unspecified
E1131	Recurrent major depressive episodes, mild
E1132	Recurrent major depressive episodes, moderate
E1133	Recurrent major depressive episodes, severe, no psychosis
E1134	Recurrent major depressive episodes, severe, with psychosis
E1135	Recurrent major depressive episodes,partial/unspec remission
E1136	Recurrent major depressive episodes, in full remission
E1137	Recurrent depression
E113z	Recurrent major depressive episode NOS
E114.	Bipolar affective disorder, currently manic
E1140	Bipolar affective disorder, currently manic, unspecified
E1141	Bipolar affective disorder, currently manic, mild
E1142	Bipolar affective disorder, currently manic, moderate
E1143	Bipolar affect disord, currently manic, severe, no psychosis
E1144	Bipolar affect disord, currently manic,severe with psychosis
E1145	Bipolar affect disord,currently manic, part/unspec remission
E1146	Bipolar affective disorder, currently manic, full remission

For peer review only - http://bmjopen.bmj.com/site/about/guidelines.xhtml

For peer review only

CODE	DESCRIPTION
E114z	Bipolar affective disorder, currently manic, NOS
E115.	Bipolar affective disorder, currently depressed
E1150	Bipolar affective disorder, currently depressed, unspecified
E1151	Bipolar affective disorder, currently depressed, mild
E1152	Bipolar affective disorder, currently depressed, moderate
E1153	Bipolar affect disord, now depressed, severe, no psychosis
E1154	Bipolar affect disord, now depressed, severe with psychosis
E1155	Bipolar affect disord, now depressed, part/unspec remission
E1156	Bipolar affective disorder, now depressed, in full remission
E115z	Bipolar affective disorder, currently depressed, NOS
E116.	Mixed bipolar affective disorder
E1160	Mixed bipolar affective disorder, unspecified
E1161	Mixed bipolar affective disorder, mild
E1162	Mixed bipolar affective disorder, moderate
E1163	Mixed bipolar affective disorder, severe, without psychosis
E1164	Mixed bipolar affective disorder, severe, with psychosis
E1165	Mixed bipolar affective disorder, partial/unspec remission
E1166	Mixed bipolar affective disorder, in full remission
E116z	Mixed bipolar affective disorder, NOS
E117.	Unspecified bipolar affective disorder
E1170	Unspecified bipolar affective disorder, unspecified
E1171	Unspecified bipolar affective disorder, mild
E1172	Unspecified bipolar affective disorder, moderate
E1173	Unspecified bipolar affective disorder, severe, no psychosis
E1174	Unspecified bipolar affective disorder,severe with psychosis
E1175	Unspecified bipolar affect disord, partial/unspec remission
E1176	Unspecified bipolar affective disorder, in full remission
E117z	Unspecified bipolar affective disorder, NOS
E118.	Seasonal affective disorder

For peer review only

CODE	DESCRIPTION
E11y.	Other and unspecified manic-depressive psychoses
E11y0	Unspecified manic-depressive psychoses
E11y1	Atypical manic disorder
E11y2	Atypical depressive disorder
E11y3	Other mixed manic-depressive psychoses
E11yz	Other and unspecified manic-depressive psychoses NOS
E11z.	Other and unspecified affective psychoses
E11z0	Unspecified affective psychoses NOS
E11zz	Other affective psychosis NOS
E12..	Paranoid states
E120.	Simple paranoid state
E121.	Chronic paranoid psychosis
E122.	Paraphrenia
E123.	Shared paranoid disorder
E12y.	Other paranoid states
E12y0	Paranoia querulans
E12yz	Other paranoid states NOS
E12z.	Paranoid psychosis NOS
E13..	Other nonorganic psychoses
E130.	Reactive depressive psychosis
E131.	Acute hysterical psychosis
E132.	Reactive confusion
E133.	Acute paranoid reaction
E134.	Psychogenic paranoid psychosis
E135.	Agitated depression
E13y.	Other reactive psychoses
E13y0	Psychogenic stupor
E13y1	Brief reactive psychosis
E13yz	Other reactive psychoses NOS

For peer review only - http://bmjopen.bmj.com/site/about/guidelines.xhtml

CODE	DESCRIPTION
E13z.	Nonorganic psychosis NOS
E1y..	Other specified non-organic psychoses
E1z..	Non-organic psychosis NOS
E200.	Anxiety states
E2000	Anxiety state unspecified
E2001	Panic disorder
E2002	Generalised anxiety disorder
E2003	Anxiety with depression
E2004	Chronic anxiety
E2005	Recurrent anxiety
E200z	Anxiety state NOS
E204.	Neurotic depression reactive type
E212.	Schizoid personality disorder
E2120	Unspecified schizoid personality disorder
E2121	Introverted personality
E2122	Schizotypal personality
E212z	Schizoid personality disorder NOS
E258.	Nondependent antidepressant type drug abuse
E2580	Nondependent antidepressant type drug abuse, unspecified
E2581	Nondependent antidepressant type drug abuse, continuous
E2582	Nondependent antidepressant type drug abuse, episodic
E2583	Nondependent antidepressant type drug abuse in remission
E258z	Nondependent antidepressant type drug abuse NOS
E291.	Prolonged depressive reaction
E2B..	Depressive disorder NEC
E2B0.	Postviral depression
E2B1.	Chronic depression
Eu...	[X]Mental and behavioural disorders
Eu2..	[X]Schizophrenia, schizotypal and delusional disorders

For peer review only

For peer review only - http://bmjopen.bmj.com/site/about/guidelines.xhtml

CODE	DESCRIPTION
Eu20.	[X]Schizophrenia
Eu200	[X]Paranoid schizophrenia
Eu201	[X]Hebephrenic schizophrenia
Eu202	[X]Catatonic schizophrenia
Eu203	[X]Undifferentiated schizophrenia
Eu204	[X]Post-schizophrenic depression
Eu205	[X]Residual schizophrenia
Eu206	[X]Simple schizophrenia
Eu20y	[X]Other schizophrenia
Eu20z	[X]Schizophrenia, unspecified
Eu21.	[X]Schizotypal disorder
Eu22.	[X]Persistent delusional disorders
Eu220	[X]Delusional disorder
Eu221	[X]Delusional misidentification syndrome
Eu222	[X]Cotard syndrome
Eu223	[X]Paranoid state in remission
Eu22y	[X]Other persistent delusional disorders
Eu22z	[X]Persistent delusional disorder, unspecified
Eu23.	[X]Acute and transient psychotic disorders
Eu230	[X]Acute polymorphic psychot disord without symp of schizoph
Eu231	[X]Acute polymorphic psychot disord with symp of schizophren
Eu232	[X]Acute schizophrenia-like psychotic disorder
Eu233	[X]Other acute predominantly delusional psychotic disorders
Eu23y	[X]Other acute and transient psychotic disorders
Eu23z	[X]Acute and transient psychotic disorder, unspecified
Eu24.	[X]Induced delusional disorder
Eu25.	[X]Schizoaffective disorders
Eu250	[X]Schizoaffective disorder, manic type
Eu251	[X]Schizoaffective disorder, depressive type

For peer review only - http://bmjopen.bmj.com/site/about/guidelines.xhtml

For peer review only

CODE	DESCRIPTION
Eu252	[X]Schizoaffective disorder, mixed type
Eu25y	[X]Other schizoaffective disorders
Eu25z	[X]Schizoaffective disorder, unspecified
Eu26.	[X]Nonorganic psychosis in remission
Eu2y.	[X]Other nonorganic psychotic disorders
Eu2z.	[X]Unspecified nonorganic psychosis
Eu3..	[X]Mood - affective disorders
Eu30.	[X]Manic episode
Eu300	[X]Hypomania
Eu301	[X]Mania without psychotic symptoms
Eu302	[X]Mania with psychotic symptoms
Eu30y	[X]Other manic episodes
Eu30z	[X]Manic episode, unspecified
Eu31.	[X]Bipolar affective disorder
Eu310	[X]Bipolar affective disorder, current episode hypomanic
Eu311	[X]Bipolar affect disorder cur epi manic wout psychotic symp
Eu312	[X]Bipolar affect disorder cur epi manic with psychotic symp
Eu313	[X]Bipolar affect disorder cur epi mild or moderate depressn
Eu314	[X]Bipol aff disord, curr epis sev depress, no psychot symp
Eu315	[X]Bipolar affect dis cur epi severe depres with psyc symp
Eu316	[X]Bipolar affective disorder, current episode mixed
Eu317	[X]Bipolar affective disorder, currently in remission
Eu318	[X]Bipolar affective disorder type I
Eu319	[X]Bipolar affective disorder type II
Eu31y	[X]Other bipolar affective disorders
Eu31z	[X]Bipolar affective disorder, unspecified
Eu32.	[X]Depressive episode
Eu320	[X]Mild depressive episode
Eu321	[X]Moderate depressive episode

For peer review only - http://bmjopen.bmj.com/site/about/guidelines.xhtml

For peer review only

CODE	DESCRIPTION
Eu322	[X]Severe depressive episode without psychotic symptoms
Eu323	[X]Severe depressive episode with psychotic symptoms
Eu324	[X]Mild depression
Eu328	[X]Major depression, severe with psychotic symptoms
Eu329	[X]Single major depr ep, severe with psych, psych in remiss
Eu32A	[X]Recurr major depr ep, severe with psych, psych in remiss
Eu32y	[X]Other depressive episodes
Eu32z	[X]Depressive episode, unspecified
Eu33.	[X]Recurrent depressive disorder
Eu330	[X]Recurrent depressive disorder, current episode mild
Eu331	[X]Recurrent depressive disorder, current episode moderate
Eu332	[X]Recurr depress disorder cur epi severe without psyc sympt
Eu333	[X]Recurrent depress disorder cur epi severe with psyc symp
Eu334	[X]Recurrent depressive disorder, currently in remission
Eu33y	[X]Other recurrent depressive disorders
Eu33z	[X]Recurrent depressive disorder, unspecified
Eu34.	[X]Persistent mood affective disorders
Eu341	[X]Dysthymia
Eu34y	[X]Other persistent mood affective disorders
Eu34z	[X]Persistent mood affective disorder, unspecified
Eu3y.	[X]Other mood affective disorders
Eu3y0	[X]Other single mood affective disorders
Eu3y1	[X]Other recurrent mood affective disorders
Eu3yy	[X]Other specified mood affective disorders
Eu3z.	[X]Unspecified mood affective disorder
Eu41.	[X]Other anxiety disorders
Eu410	[X]Panic disorder [episodic paroxysmal anxiety]
Eu411	[X]Generalized anxiety disorder
Eu412	[X]Mixed anxiety and depressive disorder

For peer review only

CODE	DESCRIPTION
Eu413	[X]Other mixed anxiety disorders
Eu41y	[X]Other specified anxiety disorders
Eu41z	[X]Anxiety disorder, unspecified
Eu601	[X]Schizoid personality disorder
R2y2.	[D]Nervousness
SL90.	Antidepressant poisoning
SL90z	Anti-depressant poisoning NOS
SyuFK	[X]Poisoning by other and unspecified antidepressants
T840.	Accidental poisoning by antidepressants
T840z	Accidental poisoning by antidepressants NOS
TJ90.	Adverse reaction to antidepressants
TJ90z	Adverse reaction to antidepressants NOS
U6090	[X]Tricyc/tetracyc antidepres caus advers eff therapeut use
U6091	[X]Monoamine oxid inhib antidep caus advers eff therap use
U6092	[X]Other unspec antidepres caus advers effect in therap use
ZV110	[V]Personal history of schizophrenia
ZV111	[V]Personal history of affective disorder

Fit notes (Read)

CODE	DESCRIPTION
13JJ1	Benefit agncy reprt unfit for work but fit note no lngr need
9D1..	MED3 - doctor's statement
9D11.	MED3 issued to patient
9D12.	MED3 duplicate issued
9D14.	MED3 issued - back to work
9D15.	eMED3 (2010) new statement issued, not fit for work
9D19.	MED3 (2010) issued by hand, not fit for work
9D1C.	MED3 (2010) issued to patient

For peer review only - http://bmjopen.bmj.com/site/about/guidelines.xhtml

9D1Z.	MED3 - NOS
9D5..	Private sickness certificate
9DC..	SC1 - self certificate admin.
9DC1.	SC1 self-certificate advised
9DC2.	SC1 self certificate given
9DCZ.	SC1 self-certification NOS
9DL..	SC2 - self-certificate administration
9DL0.	SC2 self-certificate advised
9DL1.	SC2 self certificate given
9K8..	Sickness certificate
ZV680	[V]Issue of medical certificate

Hospital admissions for Non-COVID-19 respiratory infections (ICD-10)

CODE	DESCRIPTION
A37	Whooping cough
A370	Whooping cough due to Bordetella pertussis
A371	Whooping cough due to Bordetella parapertussis
A378	Whooping cough due to other Bordetella species
A379	Whooping cough, unspecified
B012	Varicella pneumonia
B052	Measles complicated by pneumonia
B250	Cytomegaloviral pneumonitis
J10	Influenza due to identified seasonal influenza virus
J100	Influenza with pneumonia, seasonal influenza virus identified
J101	Influenza with other respiratory manifestations, seasonal influenza virus identified
J108	Influenza with other manifestations, seasonal influenza virus identified
J11	Influenza, virus not identified
J110	Influenza with pneumonia, virus not identified

For peer review only - http://bmjopen.bmj.com/site/about/guidelines.xhtml

CODE	DESCRIPTION
J111	Influenza with other respiratory manifestations, virus not identified
J118	Influenza with other manifestations, virus not identified
J12	Viral pneumonia, not elsewhere classified
J120	Adenoviral pneumonia
J121	Respiratory syncytial virus pneumonia
J122	Parainfluenza virus pneumonia
J123	Human metapneumovirus pneumonia
J128	Other viral pneumonia
J129	Viral pneumonia, unspecified
J15	Bacterial pneumonia, not elsewhere classified
J150	Pneumonia due to Klebsiella pneumoniae
J151	Pneumonia due to Pseudomonas
J152	Pneumonia due to staphylococcus
J153	Pneumonia due to streptococcus, group B
J154	Pneumonia due to other streptococci
J155	Pneumonia due to Escherichia coli
J156	Pneumonia due to other Gram-negative bacteria
J157	Pneumonia due to Mycoplasma pneumoniae
J158	Other bacterial pneumonia
J159	Bacterial pneumonia, unspecified
J16	Pneumonia due to other infectious organisms, not elsewhere classified
J168	Pneumonia due to other specified infectious organisms
J17	Pneumonia in diseases classified elsewhere
J170	Pneumonia in bacterial diseases classified elsewhere
J171	Pneumonia in viral diseases classified elsewhere
J172	Pneumonia in mycoses
J173	Pneumonia in parasitic diseases
J178	Pneumonia in other diseases classified elsewhere
J18	Pneumonia, organism unspecified

For peer review only - http://bmjopen.bmj.com/site/about/guidelines.xhtml

CODE	DESCRIPTION
J180	Bronchopneumonia, unspecified
J181	Lobar pneumonia, unspecified
J182	Hypostatic pneumonia, unspecified
J188	Other pneumonia, organism unspecified
J189	Pneumonia, unspecified
J20	Acute bronchitis
J200	Acute bronchitis due to Mycoplasma pneumoniae
J201	Acute bronchitis due to Haemophilus influenzae
J202	Acute bronchitis due to streptococcus
J203	Acute bronchitis due to coxsackievirus
J204	Acute bronchitis due to parainfluenza virus
J205	Acute bronchitis due to respiratory syncytial virus
J206	Acute bronchitis due to rhinovirus
J207	Acute bronchitis due to echovirus
J208	Acute bronchitis due to other specified organisms
J209	Acute bronchitis, unspecified
J21	Acute bronchiolitis
J210	Acute bronchiolitis due to respiratory syncytial virus
J211	Acute bronchiolitis due to human metapneumovirus
J218	Acute bronchiolitis due to other specified organisms
J219	Acute bronchiolitis, unspecified
J41	Simple and mucopurulent chronic bronchitis
J410	Simple chronic bronchitis
J411	Mucopurulent chronic bronchitis
J418	Mixed simple and mucopurulent chronic bronchitis
J43	Emphysema
J430	MacLeod syndrome
J431	Panlobular emphysema
J432	Centrilobular emphysema

For peer review only - http://bmjopen.bmj.com/site/about/guidelines.xhtml

CODE	DESCRIPTION
J438	Other emphysema
J439	Emphysema, unspecified
J440	Chronic obstructive pulmonary disease with acute lower respiratory infection
J441	Chronic obstructive pulmonary disease with acute exacerbation, unspecified
J448	Other specified chronic obstructive pulmonary disease
J449	Chronic obstructive pulmonary disease, unspecified
J45	Asthma
J450	Predominantly allergic asthma
J451	Nonallergic asthma
J458	Mixed asthma
J459	Asthma, unspecified
J851	Abscess of lung with pneumonia
J86	Pyothorax
J860	Pyothorax with fistula
J869	Pyothorax without fistula
J982	Interstitial emphysema
J983	Compensatory emphysema
U04	Severe acute respiratory syndrome [SARS]

For peer review only - http://bmjopen.bmj.com/site/about/guidelines.xhtml

For peer review only

Supplementary material 3: The RECORD statement – checklist of items, extended from the STROBE statement, that should be reported in observational studies using routinely collected health data.

	Item No.	STROBE items	Location in manuscript where items are reported	RECORD items	Location in manuscript where items are reported
**Title and abstract**					
	1	(a) Indicate the study's design with a commonly used term in the title or the abstract<br><br>(b) Provide in the abstract an informative and balanced summary of what was done and what was found	Page 1<br><br>Page 3	RECORD 1.1: The type of data used should be specified in the title or abstract. When possible, the name of the databases used should be included.<br><br>RECORD 1.2: If applicable, the geographic region and timeframe within which the study took place should be reported in the title or abstract.<br><br>RECORD 1.3: If linkage between databases was conducted for the study, this should be clearly stated in the title or abstract.	Page 1/ Page 3<br><br>Page 1/ Page 3<br><br>Page 3
**Introduction**					
Background rationale	2	Explain the scientific background and rationale for the investigation being reported	Page 5		
Objectives	3	State specific objectives, including any prespecified hypotheses	Page 5		
**Methods**					
Study Design	4	Present key elements of study design early in the paper	Page 6		
Setting	5	Describe the setting, locations, and relevant dates, including periods of recruitment, exposure, follow-up, and data collection	Page 6 and 7		
Participants	6	*(a) Cohort study* - Give the eligibility criteria, and the sources and methods of	Page 6	RECORD 6.1: The methods of study population selection (such as codes or	NA

For peer review only - http://bmjopen.bmj.com/site/about/guidelines.xhtml

		selection of participants. Describe methods of follow-up<br>*Case-control study* - Give the eligibility criteria, and the sources and methods of case ascertainment and control selection. Give the rationale for the choice of cases and controls<br>*Cross-sectional study* - Give the eligibility criteria, and the sources and methods of selection of participants<br><br>*(b) Cohort study* - For matched studies, give matching criteria and number of exposed and unexposed<br>*Case-control study* - For matched studies, give matching criteria and the number of controls per case		algorithms used to identify subjects) should be listed in detail. If this is not possible, an explanation should be provided.<br><br>RECORD 6.2: Any validation studies of the codes or algorithms used to select the population should be referenced. If validation was conducted for this study and not published elsewhere, detailed methods and results should be provided.<br><br>RECORD 6.3: If the study involved linkage of databases, consider use of a flow diagram or other graphical display to demonstrate the data linkage process, including the number of individuals with linked data at each stage.	 <br><br><br>NA<br><br><br><br><br><br><br>NA
Variables	7	Clearly define all outcomes, exposures, predictors, potential confounders, and effect modifiers. Give diagnostic criteria, if applicable.	Page 6/7	RECORD 7.1: A complete list of codes and algorithms used to classify exposures, outcomes, confounders, and effect modifiers should be provided. If these cannot be reported, an explanation should be provided.	Page 6/7
Data sources/ measurement	8	For each variable of interest, give sources of data and details of methods of assessment (measurement).<br>Describe comparability of assessment methods if there is more than one group	Page 7 and supplementary material 1		
Bias	9	Describe any efforts to address potential sources of bias	Page 16		
Study size	10	Explain how the study size was arrived at	Page 8		
Quantitative variables	11	Explain how quantitative variables were handled in the analyses. If applicable, describe which groupings were chosen, and why	Page 8/9		

For peer review only - http://bmjopen.bmj.com/site/about/guidelines.xhtml

For peer review only

Statistical methods	12	(a) Describe all statistical methods, including those used to control for confounding<br>(b) Describe any methods used to examine subgroups and interactions<br>(c) Explain how missing data were addressed<br>(d) *Cohort study* - If applicable, explain how loss to follow-up was addressed<br>*Case-control study* - If applicable, explain how matching of cases and controls was addressed<br>*Cross-sectional study* - If applicable, describe analytical methods taking account of sampling strategy<br>(e) Describe any sensitivity analyses	Page 8/9		
Data access and cleaning methods		..		RECORD 12.1: Authors should describe the extent to which the investigators had access to the database population used to create the study population.<br><br>RECORD 12.2: Authors should provide information on the data cleaning methods used in the study.	Page 9<br><br>Page 9
Linkage		..		RECORD 12.3: State whether the study included person-level, institutional-level, or other data linkage across two or more databases. The methods of linkage and methods of linkage quality evaluation should be provided.	Page 6
**Results**					
Participants	13	(a) Report the numbers of individuals at each stage of the study (*e.g.*, numbers potentially eligible, examined for eligibility, confirmed eligible, included in the study, completing follow-up, and analysed)		RECORD 13.1: Describe in detail the selection of the persons included in the study (*i.e.,* study population selection) including filtering based on data quality, data availability and linkage. The selection of included persons	Page 9

For peer review only - http://bmjopen.bmj.com/site/about/guidelines.xhtml

		(b) Give reasons for non-participation at each stage.<br>(c) Consider use of a flow diagram		can be described in the text and/or by means of the study flow diagram.	
Descriptive data	14	(a) Give characteristics of study participants (*e.g.*, demographic, clinical, social) and information on exposures and potential confounders<br>(b) Indicate the number of participants with missing data for each variable of interest<br>(c) *Cohort study* - summarise follow-up time (*e.g.*, average and total amount)	Page 10, table 1		
Outcome data	15	*Cohort study* - Report numbers of outcome events or summary measures over time<br>*Case-control study* - Report numbers in each exposure category, or summary measures of exposure<br>*Cross-sectional study* - Report numbers of outcome events or summary measures	Page 12, table 2		
Main results	16	(a) Give unadjusted estimates and, if applicable, confounder-adjusted estimates and their precision (e.g., 95% confidence interval). Make clear which confounders were adjusted for and why they were included<br>(b) Report category boundaries when continuous variables were categorized<br>(c) If relevant, consider translating estimates of relative risk into absolute risk for a meaningful time period	Table 1 and supplementary material 5,6,7		
Other analyses	17	Report other analyses done—e.g., analyses of subgroups and interactions, and sensitivity analyses	Pages 13/14 and supplementary material 6,7, 8		
**Discussion**					
Key results	18	Summarise key results with reference to study objectives	Page 14		

For peer review only - http://bmjopen.bmj.com/site/about/guidelines.xhtml

Limitations	19	Discuss limitations of the study, taking into account sources of potential bias or imprecision. Discuss both direction and magnitude of any potential bias	Page 15/16	RECORD 19.1: Discuss the implications of using data that were not created or collected to answer the specific research question(s). Include discussion of misclassification bias, unmeasured confounding, missing data, and changing eligibility over time, as they pertain to the study being reported.	Page 16
Interpretation	20	Give a cautious overall interpretation of results considering objectives, limitations, multiplicity of analyses, results from similar studies, and other relevant evidence	Page 17		
Generalisability	21	Discuss the generalisability (external validity) of the study results	Page 17/18		
**Other Information**					
Funding	22	Give the source of funding and the role of the funders for the present study and, if applicable, for the original study on which the present article is based	Page 2		
Accessibility of protocol, raw data, and programming code		..		RECORD 22.1: Authors should provide information on how to access any supplemental information such as the study protocol, raw data, or programming code.	Page 6

For peer review only

For peer review only - http://bmjopen.bmj.com/site/about/guidelines.xhtml

Supplementary material 4: Hospital admissions for respiratory infections not necessarily recorded as COVID-19 for domiciliary care workers from March 2016 to October 2021.

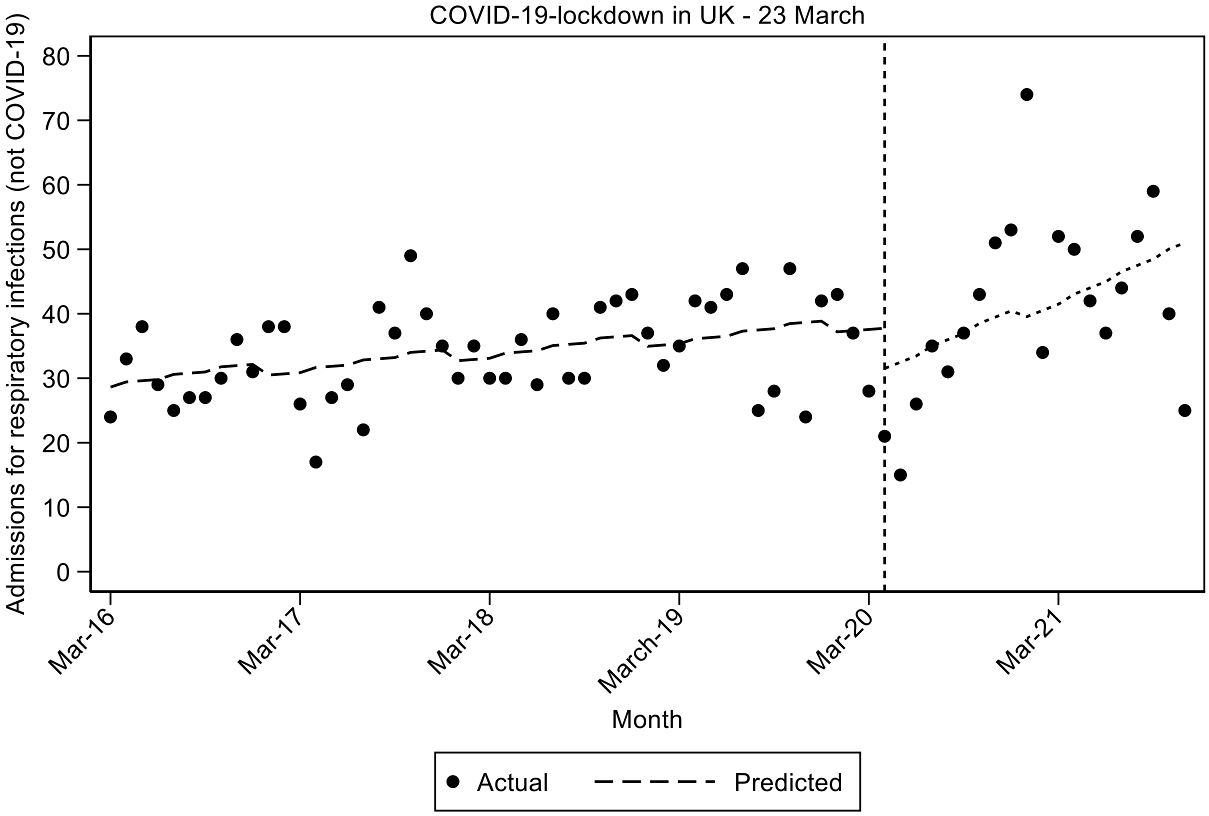

iew only

For peer review only - http://bmjopen.bmj.com/site/about/guidelines.xhtml

Supplementary material 5: Characteristics of domiciliary care workers with at least one confirmed COVID-19 infection in the COVID-19 pandemic between 1st March 2020 and 31st November 2021.

	Confirmed COVID-19 infection[a] N= 3,698		
**DCW Characteristics**	**N**	**%**	**Crude hazard ratio (95% confidence interval)**
Sex[b]			
Female	3,226	24.3	ref
Male	472	19.1	0.76 (0.69 to 0.84)
Age (at registration)[b]			
Under 35 years	1164	26.3	ref
35 to <45 years	795	28.2	1.07 (0.97 to 1.17)
45 to <55 years	897	22.7	0.85 (0.78 to 0.93)
55 years and over	842	18.6	0.68 (0.62 to 0.74)
Ethnicity[b]			
White	3,537	23.6	ref
Black	35	24.7	1.06 (0.76 to 1.48)
Mixed	*	*	0.98 (0.68 to 1.42)
Asian	25	23.2	1.01 (0.68 to 1.50)
Other	*	*	0.86 (0.50 to 1.48)
Mixed/Other	42	21.8	
Not recorded			
** numbers suppressed as <10. Mixed and Other categories are combined.*			
Lives with DCW[b]			
No	3,444	23.4	ref
Yes	254	26.1	1.15 (1.01 to 1.30)
Have disability[b]			
No	3,310	23.4	ref
Yes	30	16.0	0.67 (0.47 to 0.96)
Not recorded /Prefer not to answer	*358*	*25.6*	-
Qualifications as recorded at registration[b]			
Competence confirmed	438	22.1	ref
Induction Framework	737	24.7	1.15 (1.02 to 1.29)
Qualification Level 2	1,672	24.1	1.12 (1.00 to 1.24)
Qualification Level 3	752	22.5	1.03 (0.91 to 1.15)
Qualification Level 4+	88	20.0	0.91 (0.72 to 1.14)
Not recorded	*11*	*25.6*	-
Employment sector as recorded at registration[b]			
Private	1,480	22.5	ref
Third Sector	991	23.2	1.03 (0.95 to 1.12)
Local Authority-Social Services	876	26.5	1.23 (1.13 to 1.34)
Recruitment Agency/ Employment Agency/Health/Other	43	25.0	1.14 (0.84 to 1.54)
Not recorded	*308*	*22.2*	-
Smoking status[c]			
Non-smoker	1,638	25.8	ref

For peer review only - http://bmjopen.bmj.com/site/about/guidelines.xhtml

	Confirmed COVID-19 infection[a] N= 3,698		
**DCW Characteristics**	**N**	**%**	**Crude hazard ratio (95% confidence interval)**
Ex-smoker	1,185	23.7	0.97 (0.89 to 1.05)
Current smoker	875	20.0	0.74 (0.68 to 0.80)
Not recorded			-
Body Mass Index[c]			
Underweight (<18.5)	48	18.8	0.84 (0.63 to 1.13)
Healthy weight (18.5-24.9)	685	21.6	ref
Overweight (25-29.9)	847	23.2	1.09 (0.99 to 1.21)
Obese (≥30)	1398	26.3	1.27 (1.16 to 1.39)
Not recorded	*720*	*21.6*	-
Any comorbidity[c,d,e]			
No	2,046	22.9	ref
Yes	1,652	24.3	1.07 (1.00 to 1.14)
On COVID-19 shielding list			
No	3,569	23.7	ref
Yes	129	20.3	0.85 (0.72 to 1.02)
**Area level characteristics[f, g]**			
Resident health board			
Aneurin Bevan	758	26.9	ref
Betsi Cadwaladr	665	18.1	0.63 (0.57 to 0.70)
Cardiff and Vale	475	26.5	0.96 (0.85 to 1.07)
Cwm Taf Morgannwg	755	27.9	1.05 (0.95 to 1.16)
Hywel Dda	392	20.0	0.69 (0.61 to 0.78)
Powys	104	15.7	0.53 (0.43 to 0.65)
Swansea Bay	549	26.2	0.96 (0.86 to 1.07)
WIMD 2019 quintile			
1 (most deprived)	1,143	27.4	ref
2	991	25.0	0.90 (0.83 to 0.98)
3	697	21.1	0.74 (0.67 to 0.81)
4	503	19.7	0.68 (0.61 to 0.76)
5 (most affluent)	364	21.0	0.74 (0.65 to 0.83)
Rurality classification			
Rural	947	19.2	ref
Urban	2,751	25.5	1.38 (1.28 to 1.49)

a between 1st March 2020 to 31st November 2021; b data source = Social Care Wales (DSCW) data and include data that is self-reported by DCWs at 1st April. If missing, then other sources (ONS 2011 Census Wales (CENW), Wales Longitudinal General Practice (WLGP), Patient Episode Dataset for Wales (PEDW), Emergency Department Dataset (EDDS)) were used to impute (at index date=1st March 2020); c data source = WLGP; d data source=PEDW; e most recent occurring between 1st January 2000 and 1st March 2020; f based on residential address at 1st March 2020; g data source =Welsh Demographic Service Dataset (WDSD).

For peer review only

Supplementary material 6: Characteristics of domiciliary care workers with at least one contacts and diagnosis for mental health and psychotropic medication (in primary care), and hospital admissions in the COVID-19 pandemic between 1st March 2020 and 31st November 2021.

	Contacts and diagnosis for mental health, psychotropic medication and admissions[a]		
**DCW Characteristics**	**N**	**%**	**Crude HR (95% CI)**
Sex[b]			
Female	4,685	35.4	ref
Male	615	24.9	0.61 (0.55 to 0.67)
Age (at registration)[b]			
Under 35 years	1,542	34.9	ref
35 to <45 years	977	34.6	0.99 (0.90 to 1.09)
45 to <55 years	1,398	35.4	1.02 (0.93 to 1.12)
55 years and over	1,383	30.6	0.82 (0.75 to 0.90)
Ethnicity[b]			
White	5,128	34.2	ref
Black	22	15.5	0.35 (0.22 to 0.55)
Mixed	*	*	0.73 (0.49 to 1.07)
Asian	18	16.7	0.39 (0.22 to 0.62)
Other	*	*	0.39 (0.19 to 0.71)
Mixed/Other	46	23.8	
Not recorded	*86*	*31.9*	-
** numbers suppressed as <10. Mixed and Other categories are combined.*			
Lives with DCW[b]			
No	4,988	33.8	ref
Yes	312	32.0	0.92 (0.80 to 1.06)
Have disability[b]			
No	4,714	33.3	ref
Yes	91	48.4	1.88 (1.41 to 2.50)
Not recorded /Prefer not to answer	*495*	*35.5*	-
Qualifications as recorded at registration[b]			
Competence confirmed	646	32.6	ref
Induction Framework	1,094	36.7	1.20 (1.06 to 1.35)
Qualification Level 2	2,328	33.6	1.05 (0.94 to 1.16)
Qualification Level 3	1,077	32.3	0.98 (0.87 to 1.11)
Qualification Level 4+	140	31.8	0.96 (0.77 to 1.20)
Not recorded	*15*	*34.9*	-
Employment sector as recorded at registration[b]			
Private	2,154	32.7	ref
Third Sector	1,454	34.1	1.06 (0.98 to 1.16)
Local Authority-Social Services	1,159	35.1	1.11 (1.02 to 1.21)
Recruitment Agency/ Employment Agency Health/Other	65	37.8	1.25 (0.91 to 1.71)
Not recorded	*468*	*33.7*	-

For peer review only - http://bmjopen.bmj.com/site/about/guidelines.xhtml

	Contacts and diagnosis for mental health, psychotropic medication and admissions[a]		
**DCW Characteristics**	**N**	**%**	**Crude HR (95% CI)**
Smoking status[c]			
Non-smoker	2,076	32.7	ref
Ex-smoker	1,443	28.8	1.39 (1.28 to 1.52)
Current smoker	1,781	40.7	1.41 (1.30 to 1.53)
Not recorded			-
Body Mass Index[c]			
Underweight (<18.5)	99	38.7	1.28 (0.99 to 1.67)
Healthy weight (18.5-24.9)	1,045	32.9	ref
Overweight (25-29.9)	1,341	36.7	1.18 (1.07 to 1.31)
Obese (≥30)	2,320	43.7	1.58 (1.44 to 1.73)
Not recorded	*495*	*14.9*	-
Any comorbidity[c,d,e]			
No	2,095	23.5	ref
Yes	3,205	47.1	2.90 (2.71 to 3.11)
On COVID-19 shielding list			
No	5,033	33.4	ref
Yes	267	42.1	1.45 (1.23 to 1.70)
**Area level characteristics[f, g]**			
Resident health board			
Aneurin Bevan	980	34.8	ref
Betsi Cadwaladr	1,140	31.0	0.84 (0.76 to 1.70)
Cardiff and Vale	650	36.2	1.06 (0.94 to 1.20)
Cwm Taf Morgannwg	1,078	39.9	1.25 (1.12 to 1.39)
Hywel Dda	561	28.6	0.75 (0.66 to 0.85)
Powys	105	15.9	0.35 (0.28 to 0.44)
Swansea Bay	786	37.5	1.12 (1.00 to 1.26)
WIMD 2019 quintile			
1 (most deprived)	1,541	37.0	Ref
2	1,392	35.1	0.92 (0.84 to 1.01)
3	1,005	30.5	0.75 (0.68 to 0.82)
4	780	30.5	0.75 (0.67 to 0.83)
5 (most affluent)	582	33.6	0.86 (0.77 to 0.97)
Rurality classification			
Rural	1,435	29.1	ref
Urban	3,865	35.8	1.36 (1.26 to 1.46)

a between 1st March 2020 to 31st November 2021; b data source = Social Care Wales (DSCW) data and include data that is self-reported by DCWs at 1st April. If missing, then other sources (ONS 2011 Census Wales (CENW), Wales Longitudinal General Practice (WLGP), Patient Episode Dataset for Wales (PEDW), Emergency Department Dataset (EDDS)) were used to impute (at index date=1st March 2020); c data source = WLGP; d data source=PEDW; e most recent occurring between 1st January 2000 and 1st March 2020; f based on residential address at 1st March 2020; g data source =Welsh Demographic Service Dataset (WDSD).

For peer review only

Supplementary material 7: Characteristics of domiciliary care workers receiving at least one fit note in the COVID-19 pandemic between 1st March 2020 and 31st November 2021.

Characteristics	N	%	Crude HR (95% CI)
**Source: SCW[a]**			
Sex			
Female	2,004	15.1	ref
Male	293	11.9	0.77 (0.68 to 0.87)
Age (at registration)			
Under 35 years	572	12.9	ref
35 to <45 years	380	13.5	1.02 (0.90 to 1.17)
45 to <55 years	625	15.8	1.22 (1.09 to 1.37)
55 years and over	720	15.9	1.23 (1.10 to 1.37)
Ethnicity			
White	2,220	14.8	ref
Black	11	7.8	0.47 (0.29 to 0.85)
Mixed	*	*	0.54 (0.29 to 1.00)
Asian	14	13.0	0.89 (0.52 to 1.50)
Other	*	*	0.89 (0.44 to 1.78)
Mixed/Other	18	9.3	-
Not recorded	34	12.6	-
** numbers suppressed as <10. Mixed and Other categories are combined.*			
Lives with DCW			
No	2,168	14.7	Ref
Yes	129	13.2	0.92 (0.77 to 1.09)
Have disability			
No	2,089	14.8	Ref
Yes	23	12.2	0.85 (0.57 to 1.29)
Missing/Prefer not to answer	185	13.3	-
Qualifications as recorded at registration			
Competence confirmed	255	12.9	ref
Induction Framework	460	15.4	1.18 (1.01 to 1.37)
Qualification Level 2	1,054	15.2	1.17 (1.02 to 1.34)
Qualification Level 3	470	14.1	1.08 (0.92 to 1.25)
Qualification Level 4+	48	10.9	0.79 (0.58 to 1.07)
Not recorded	10	23.3	-
Employment sector as recorded at registration			
Private	828	12.6	ref
Third Sector	562	13.2	1.04 (0.93 to 1.16)
Local Authority - Social Services	678	20.5	1.71 (1.54 to 1.89)
Recruitment Agency/ Employment Agency	23	13.4	
Health/Other			1.05 (0.70 to 1.59)
Not recorded	206	14.8	
Smoking status			
Non-smoker	917	14.5	ref
Ex-smoker	601	12.0	1.14 (1.03 to 1.26)
Current smoker	779	17.8	1.24 (1.13 to 1.37)

For peer review only

For peer review only - http://bmjopen.bmj.com/site/about/guidelines.xhtml

Characteristics	N	%	Crude HR (95% CI)
Body Mass Index			
Underweight (<18.5)	37	14.5	0.98 (0.70 to 1.37)
Healthy weight (18.5-24.9)	466	14.7	Ref
Overweight (25-29.9)	576	15.8	1.05 (0.93 to 1.19)
Obese (≥30)	993	18.7	1.26 (1.13 to 1.40)
Not recorded	225	6.8	-
Any comorbidity[d]			
No	1,027	11.5	ref
Yes	1,270	18.7	1.49 (1.37 to 1.61)
On COVID-19 shielding list			
No	2,175	14.4	ref
Yes	122	19.2	1.42 (1.18 to 1.71)
Resident health board			
Aneurin Bevan	319	11.3	ref
Betsi Cadwaladr	854	23.2	2.27 (2.00 to 2.59)
Cardiff and Vale	219	12.2	0.99 (0.83 to 1.17)
Cwm Taf Morgannwg	365	13.5	1.00 (0.86 to 1.17)
Hywel Dda	225	11.5	1.01 (0.86 to 1.20)
Powys	83	12.5	2.16 (1.70 to 2.76)
Swansea Bay	232	11.1	0.83 (0.70 to 0.98)
WIMD 2019 quintile			
1 (most deprived)	636	15.3	ref
2	547	13.8	0.92 (0.82 to 1.04)
3	478	14.5	1.07 (0.95 to 1.21)
4	390	15.3	1.11 (0.98 to 1.26)
5 (most affluent)	246	14.2	0.99 (0.85 to 1.14)
Rurality classification			
Rural	718	14.6	ref
Urban	1,579	14.6	0.93 (0.85 to 1.01)

a between 1st March 2020 to 31st November 2021; b data source = Social Care Wales (DSCW) data and include data that is self-reported by DCWs at 1st April. If missing, then other sources (ONS 2011 Census Wales (CENW), Wales Longitudinal General Practice (WLGP), Patient Episode Dataset for Wales (PEDW), Emergency Department Dataset (EDDS)) were used to impute (at index date=1st March 2020); c data source = WLGP; d data source=PEDW; e most recent occurring between 1st January 2000 and 1st March 2020; f based on residential address at 1st March 2020; g data source =Welsh Demographic Service Dataset (WDSD).

For peer review only

Supplementary material 8: Demographics of DCWs across the UK

Nation	Date published	Definition	Denom.	Age	Gender	Ethnicity
Wales[1]	April 2021	DCWs	22,131	44 years (mean)	84% F<br>16% M	97% White British
OSCAR sample	April 2020	DCWs	15,725	44 years (mean)	84% F<br>16% M	96% White British
England[2]	October 2021	DCWs	590,000	44 years (mean)	84% F<br>16% M	na
Scotland[3]	December 2020	Housing support/ care at home	74,870	46 years (median)	77% F<br>17 %M<br>6% unknown<br><br>84% F<br>16% M<br>(excluding unknowns)	98% White<br>2% Asian/Black
Northern Ireland[4]	October 2020	DCWs	16,303	20-29 years (mode)	91% F<br>8% M<br>1% Other	na

na = not available

[1] Domiciliary-care-worker-ENG.pdf (socialcare.wales) https://socialcare.wales/cms_assets/file-uploads/Domiciliary-care-worker-ENG.pdf

[2] The state of the adult social care sector and workforce in England (skillsforcare.org.uk) https://www.skillsforcare.org.uk/adult-social-care-workforce-data/Workforce-intelligence/publications/national-information/The-state-of-the-adult-social-care-sector-and-workforce-in-England.aspx

[3] Adults' services workforce tables - 2019 | Scottish Social Services Workforce Data (sssc.uk.com) https://data.sssc.uk.com/data-publications/248-adults-services-workforce-in-scotland-tables-2019

[4] Rapid learning review of domiciliary care in Northern Ireland (Department of Health) https://www.health-ni.gov.uk/rli-dom-care (page 13)

For peer review only - http://bmjopen.bmj.com/site/about/guidelines.xhtml

BMJ Open

The impact of the COVID-19 pandemic on Domiciliary Care Workers in Wales (UK): a data linkage cohort study using the SAIL Databank.

Journal:	*BMJ Open*
Manuscript ID	bmjopen-2022-070637.R1
Article Type:	Original research
Date Submitted by the Author:	20-Apr-2023
Complete List of Authors:	Cannings-John, Rebecca; Cardiff University<br>Schoenbuchner, Simon; Cardiff University, Centre for Trials Research<br>Jones, Hywel; Cardiff University, Division of Population Medicine<br>Lugg-Widger, Fiona; Cardiff University, Centre for Trials Research<br>Akbari, Ashley; Swansea University Medical School, Faculty of Medicine, Health & Life Science<br>Brookes-Howell, Lucy; Cardiff University Centre for Trials Research<br>Hood, Kerenza; Cardiff University, Centre for Trials Research<br>John, Ann; Swansea University, Health Data Research UK | Administrative Data Research Wales; Cardiff University, DECIPHer - Centre for Development, Evaluation, Complexity and Implementation in Public Health Improvement<br>Thomas, Daniel; Public Health Wales, Communicable Disease Surveillance Centre; Cardiff Metropolitan University<br>Prout, Hayley; Cardiff University School of Medicine, Centre for Trials Research<br>Robling, Michael; Cardiff University, Centre for Trials Research
**Primary Subject Heading**:	Occupational and environmental medicine
Secondary Subject Heading:	Epidemiology, Infectious diseases, Mental health, Public health
Keywords:	COVID-19, MENTAL HEALTH, SOCIAL MEDICINE

SCHOLARONE™
Manuscripts

I, the Submitting Author has the right to grant and does grant on behalf of all authors of the Work (as defined in the below author licence), an exclusive licence and/or a non-exclusive licence for contributions from authors who are: i) UK Crown employees; ii) where BMJ has agreed a CC-BY licence shall apply, and/or iii) in accordance with the terms applicable for US Federal Government officers or employees acting as part of their official duties; on a worldwide, perpetual, irrevocable, royalty-free basis to BMJ Publishing Group Ltd ("BMJ") its licensees and where the relevant Journal is co-owned by BMJ to the co-owners of the Journal, to publish the Work in this journal and any other BMJ products and to exploit all rights, as set out in our licence.

The Submitting Author accepts and understands that any supply made under these terms is made by BMJ to the Submitting Author unless you are acting as an employee on behalf of your employer or a postgraduate student of an affiliated institution which is paying any applicable article publishing charge ("APC") for Open Access articles. Where the Submitting Author wishes to make the Work available on an Open Access basis (and intends to pay the relevant APC), the terms of reuse of such Open Access shall be governed by a Creative Commons licence – details of these licences and which Creative Commons licence will apply to this Work are set out in our licence referred to above.

Other than as permitted in any relevant BMJ Author's Self Archiving Policies, I confirm this Work has not been accepted for publication elsewhere, is not being considered for publication elsewhere and does not duplicate material already published. I confirm all authors consent to publication of this Work and authorise the granting of this licence.

For peer review only

Title: The impact of the COVID-19 pandemic on Domiciliary Care Workers in Wales (UK): a data linkage cohort study using the SAIL Databank.

Short running title: COVID-19 impact on domiciliary care workers

Authors:

Rebecca Cannings-John, PhD[1]

Simon Schoenbuchner, PhD[1]

Hywel Jones BSc DMS CStat[2]

Fiona Lugg-Widger, PhD[1]

Ashley Akbari, MSc[3]

Lucy Brookes-Howell, PhD[1]

Kerry Hood, PhD[1]

Ann John, MBBS MD [3,4]

Daniel Rh Thomas, PhD[5,6]

Hayley Prout, MPhil[1]

Michael Robling, PhD[1,4]

Affiliations: 1: Centre for Trials Research, School of Medicine, Cardiff University, Cardiff, United Kingdom; 2: Division of Population Medicine, Cardiff University; 3: Population Data Science, Swansea University Medical School, Faculty of Medicine, Health & Life Science, Swansea University; 4: DECIPHer - Centre for Development, Evaluation, Complexity and Implementation in Public Health Improvement, sbarc|spark, Cardiff University; 5: Public Health Wales Communicable Disease Surveillance Centre; 6: Cardiff Metropolitan University.

Corresponding author: Rebecca Cannings-John,
Centre for Trials Research,
Cardiff University,
Cardiff, UK
canningsrl@Cardiff.ac.uk
02920687248

For peer review only

ABSTRACT

Objectives: To quantify population health risks for domiciliary care workers in Wales working during the COVID-19 pandemic.

Design: A population-level retrospective routine data study linking occupational registration data to anonymised electronic health records maintained by the Secure Anonymised Information Linkage Databank in a privacy-protecting trusted research environment.

Setting: Registered domiciliary care workers population in Wales.

Participants: We examined records for all linked domiciliary care workers in Wales from 1st March 2020 to 30th November 2021.

Primary and secondary outcome measures: Our primary outcome was confirmed COVID-19 infection; secondary outcomes included contacts for mental health, fit notes, respiratory infections not necessarily recorded as COVID-19, deaths involving COVID-19, all-cause mortality and suspected COVID-19.

Results: Suspected and confirmed COVID-19 rates increased over time to 24% by 30th November 2021. Confirmed COVID-19 rates varied by characteristics of care workers (e.g. lower in males (19% vs. females 24%) and in older care workers (>55 years: 19% vs. <35 years: 26%), by where care workers lived (e.g. lower in rural areas 19% vs urban: 26%), and were higher for care workers employed by local authority social services departments compared to staff employed in the private sector (27% and 23% respectively). 34% of care workers required support for a mental health condition, with mental health related prescribing rates increasing in frequency when compared to the four years preceding the pandemic onset (a prescribing average monthly increase of 29.3 (95% CI: 20.7 to 38.0, p<0.001). Fit notes increased over the pandemic from 5% to 15% (August 2020 to November 2021 respectively). All-cause and COVID-19 related mortality are no greater than for the general population of 15- to 64-year-olds in Wales (0.1% and 0.034% respectively).

Conclusions: While initial concerns about excess mortality are alleviated, the substantial pre-existing and increased mental health burden will require investment to provide long-term support to the sector's workforce.

Keywords: Domiciliary care worker, workforce, COVID-19, mental health, data linkage, social care, Wales

For peer review only - http://bmjopen.bmj.com/site/about/guidelines.xhtml

STRENGTHS AND LIMITATIONS OF THIS STUDY

- Linking registration data to electronic health records in Wales provided a unique opportunity to explore health outcomes for DCWs working during the pandemic.
- No DCWs dissented for their registration data to be made available for linkage allowing a comprehensive population level analysis.
- Demographic data available by linked registration and healthcare datasets allowed assessment by key DCW sub-groups.
- The recording of some data could have introduced bias, e.g. coding for COVID-19 biased towards individuals presenting in primary or secondary healthcare.
- Our study provides health outcome estimates for DCWs over a longer period of the COVID-19 pandemic, for a population of care workers and a broad range of outcomes.

peer review only

For peer review only - http://bmjopen.bmj.com/site/about/guidelines.xhtml

INTRODUCTION

During 2018-20, more than 950,000 people in the UK received domiciliary care from nearly 823,000 Domiciliary Care Workers (DCWs) from over 10,100 home care providers and the demand is predicted to increase significantly over the next 20 years[1]. DCWs provide support to adults in their own home with household tasks, personal care or any activities that allow the individual to maintain independence. Support can demand close contact, assisting with bodily functions, managing continence, assisting with oral and dental care, and providing other personal care. In the UK, domiciliary care can be funded publicly by local councils, privately funded, or a combination of both. These close working conditions with clinically vulnerable people, and the varied functions that DCWs perform in a non-institutional setting in a workforce with a large number of employers, may increase exposure to COVID-19. A cross-sectional survey led by Ulster University found that during the early period of the COVID-19 pandemic (between May–July 2020 and November 2020–January 2021), both wellbeing and quality of working life deteriorated over all occupations including Social Care [2]. This decrease was again observed when care workers were surveyed between May and July 2021, with personal and work-related burnout reported to increase [2]. However, the survey did not aim to capture objectively recorded health outcomes specifically in DCWs, for example positive rates of COVID-19 infection and the risk factors which contribute to these.

From 1st April 2020, it became mandatory for DCWs in the active Welsh workforce to be registered with Social Care Wales [3]. The population of working DCWs in Wales during the COVID-19 pandemic is therefore known, and can be linked to anonymised data, including health and administrative data held in the SAIL Databank (Swansea University), providing a unique opportunity to explore a range of health outcomes (including COVID-19 infection and mortality).

The OSCAR study (Outcomes for Social Carers: an Analysis using Routine data) aimed to utilise the registration data collected by Social Care Wales, individually linked to secure anonymised electronic health records (EHRs) to quantify population health risks for DCWs in Wales working during the COVID-19 pandemic. Specifically, we aimed to quantify rates of suspected and confirmed COVID-19 infection and, to fully establish the range of adverse health outcomes affecting DCWs, key health outcomes such as mental health, fit notes, COVID-19 related and all-cause mortality, as a consequence of the pandemic. We compared mental health outcomes within the pandemic period to the pre-pandemic situation to assess the effect of the pandemic. Similarly we examined admissions over time for respiratory infection in the early pandemic to identify the potential miscoding of COVID-19 infection. Outcome variation was explored by demographics, work-related factors, lifestyle and comorbidities to develop timely public health policy messages that could be extended to DCWs in other nations of the UK.

For peer review only - http://bmjopen.bmj.com/site/about/guidelines.xhtml

MATERIALS AND METHODS

Study design and data sources

This study is a population-level mixed methods retrospective routine data linkage study with analysis guided by exploratory qualitative interviews. Qualitative findings are reported separately [4]. Registration data for all DCWs in Wales registered by 1st April 2020 are held by Social Care Wales (Domiciliary Social Care Worker (DSCW)) and available via the Secure Anonymised Information Linkage (SAIL) Databank (Swansea University) a privacy-protecting trusted research environment (TRE), which uses a standardised split-file secure, encrypted anonymisation process [5]. These data were combined with EHR data sources within the SAIL Databank (supplementary material 1 for data sources used).

Population

The study population was all registered DCWs resident in Wales on 1st March 2020 who did not subsequently opt-out to their data being transferred to the SAIL Databank and linked for research, either when DSCW data were added to the SAIL Databank or via their general practice (GP). Any DCWs not linked into the SAIL Databank with sufficient confidence were excluded. Additionally, individuals included in the dataset but registered as Domiciliary Care Managers, Adult Care Home Managers or Residential Child Care Workers were excluded.

Primary and secondary outcomes

The primary outcome was confirmed COVID-19 infection, defined as the earliest of the following events: a positive polymerase chain reaction (PCR) test (Pathology Test Results dataset, PATD) [6], hospital admission (Patient Episode Database for Wales, PEDW) [7], death registration from COVID-19 (main or secondary diagnostic ICD-10 code=U07.1) (Annual District Death Extract, ADDE from the Office for National Statistics (ONS) mortality register) [8], or a COVID-19 diagnosis (Welsh Longitudinal GP, WLGP) [9].

Secondary outcomes were:

- Suspected COVID-19 infection (Data sources: PATD, WLGP, PEDW, and ADDE);
- Contacts for mental health and diagnoses, psychotropic medication and admissions (including self-harm) (Data sources: WLGP, and PEDW);
- Fit notes as a general marker of medically confirmed illness (Data source: WLGP);
- Non-COVID-19 hospital admissions for respiratory conditions (Data source: PEDW);
- Deaths involving COVID-19 and all-cause mortality (Data source: ADDE).

For peer review only - http://bmjopen.bmj.com/site/about/guidelines.xhtml

In brief, we examined depression and anxiety symptoms and diagnoses [10,11], and severe mental health in GP data and hospital admissions data [12]. Self-harm, used GP data for definite and undetermined intent (WLGP) [13,14]. Fit notes also used GP data to cover medical/sickness/self-certificates issued to patients by GPs and included issues for returning to work. Hospital admissions for respiratory conditions were examined for potential miscoding of COVID-19 infection in the early pandemic (March to May 2020) and included any lower respiratory infection, pneumonia, and influenza-like-illness [15], exacerbations of chronic obstructive pulmonary disease (COPD) [16] and asthma [17].

Study period and follow-up

DCWs entered the cohort on 1st March 2020 and were followed up until 30th November 2021. We examined outcomes by waves of the pandemic; short term outcomes from 1st March 2020 to 31st August 2020 (Wave 1: reflecting a period before the relaxing of lockdown in Wales) and from 1st September 2020 to 28th February 2021 (Wave 2). Long-term outcomes were examined to 30th November 2021. DCWs were followed up until the earliest of occurrence of death, first migration out of Wales, the outcome of interest, or the end of the follow-up.

Domiciliary Care Worker Characteristics

We chose age, sex, ethnicity, health board, deprivation quintile and rurality as relevant characteristics for the DCWs using a number of data sources (supplementary material 1). We also identified DCWs living with disability, any comorbidities [12], shielding status, and lifestyle factors such as body mass index and smoking status. From the DSCW data, we were able to describe how individuals registered as a DCW (induction, registered with a qualification, competence confirmed by their manager) which reflects the different pathways open to care workers when mandatory registration was introduced [18]. Some pathways were transitional and are now no longer available (e.g. registration following induction). We also identified whether a DCW lived with another DCW and their employment sector at the time of registration (Private, Third sector, Local Authority/Social Services, Recruitment/Employment Agency/Other). Several items from the DSCW were not available due to confidentiality considerations, such as length of time in the job, type of employment (e.g.in social care, self-employed), and type of qualifications held (Qualifications and Credit Framework (QCF), National Vocational Qualification (NVQ) etc).

A full coding list for outcomes is in supplementary material 2 and also on Open Science Framework (OSF) (https://osf.io/9w6pe/) alongside codes for the comorbidities.

For peer review only - http://bmjopen.bmj.com/site/about/guidelines.xhtml

Governance

The study received approval from the School of Medicine Research Ethics Committee, Cardiff University [SMREC 20/114]. Approval was obtained from the SAIL Information Governance Review Panel (IGRP), Swansea University (Project ID: 1126) and data access was granted to named staff. Approval from the ONS Research Accreditation Panel (ONS RAP) was provided (16/12/2020) and approval from Social Care Wales was provided (26/01/2021). This process was generic and not specific to the OSCAR study.

Analysis

The linkage of DCWs to EHR were described and the study population characterised using frequencies and percentages. The presence of key health outcomes were described by the short and long-term periods; prevalence estimates presented alongside 95% confidence intervals (CIs). This study is primarily designed to describe variation in outcomes in the DCW population, by key subgroups, rather than for prediction or causal models. Using multilevel Cox regression to model time to first confirmed COVID-19 infection, we estimated hazard ratios (HRs) with 95% CIs, to quantify the risk of confirmed COVID-19 by comorbidity status adjusting for age, sex and deprivation, and also by their employing sector adjusting for health board, rurality, and qualification type. DCWs were censored at the time of the event, migration, death, or at the study end. Equivalent up-to-date DCW workforce data from Scotland, Northern Ireland (NI) and England was obtained and compared to the Welsh DCW cohort allowing us to generalise the estimate confirmed COVID-19 other nations.

To explore changes over time and the effect of the COVID-19 pandemic period, an interrupted time series approach was used to test the hypothesis that hospital admissions for non-COVID-19 respiratory infections would increase in comparison to the rates seen pre-March 2020 if there was misclassification of infections. Monthly data were divided into pre-pandemic (1st March 2016 to 28th February 2020) and the pandemic period (1st March 2020 to 31st October 2022). The changes in levels (baseline value of the outcome at time zero) and trends (the rate of change) of the pre-pandemic segment and pandemic segment were analysed using the *itsa* command in Stata. A Cumby-Huizinga test was used to determine the autocorrelation and was adjusted using Newey West AC estimators. Using the same approach and as a post-hoc analysis, the number of mental health GP contacts and prescriptions, and hospital admissions were also examined.

To comply with disclosure control process and approvals, and protect the privacy of anonymised individuals, numbers <5 are suppressed. Percentages associated with small frequencies are rounded to the nearest 1%. Study findings were reported in accordance with applicable reporting guidelines for observational studies using administrative data (STROBE and RECORD) (supplementary table 3)

For peer review only - http://bmjopen.bmj.com/site/about/guidelines.xhtml

[19,20]. Data cleaning, cohort assembly and statistical analyses were performed using Structured Query Language (SQL) (IBM Db2 11.1) [21] and R (v4.1.0-4.1.3) [22,23], and Stata (v17)[24] within the SAIL Databank privacy-protecting TRE.

Patient and public involvement

Two stakeholder groups provided input to the project, an ongoing Study Advisory Group and an Implementation Reference Group. Both groups included membership drawn from the domiciliary care sector and both contributed to interpretation of emerging results.

RESULTS

Study population

Data from 15,931 DCWs resident and working in Wales on 1st March 2020 were linked to EHR data (85% of them had GP records in SAIL). Records from 206 DCWs were excluded due to matching errors, leaving 15,725 (98.7%) DCWs included in the final analysis. The majority of DCWs were female (84%), of white ethnic background (95%), 43% had at least one comorbidity of which 22% had been recorded with mental health related illness (Table 1).

Confirmed COVID-19 infection

Confirmed COVID-19 infection was 1.2% at 31st August 2020, rose to 14% by the end of February 2021, and reached 24% by the end of November 2021 (Table 2). There was no evidence to suggest a miscoding of COVID-19 infection in the early pandemic, with no difference in admissions for non-COVID-19 respiratory conditions over time (supplementary material 4). Confirmed COVID-19 infection was more prevalent in females, those who registered following the induction pathway compared to those with competency confirmed by a manager, those employed by the Local Authority Social Services at registration compared to those employed in the private sector (Figure 1), those living with another DCW, those with a BMI greater than 30 compared to those with a healthy BMI (18.5 to 24.9) and those living in an urban area (Table 1). Confirmed COVID-19 infection was less prevalent in older DCW. Variation was also observed across health boards. After adjusting for age, sex and deprivation, COVID-19 infection was still more prevalent in those with a comorbidity (HR=1.08, 95% CI: 1.01 to 1.15) and additionally adjusting for health board, rurality, and qualification type, was more prevalent in those employed by the Local Authority Social Services at registration (1.35, 1.23 to 1.47).

For peer review only - http://bmjopen.bmj.com/site/about/guidelines.xhtml

Table 1. Characteristics of Domiciliary Care Workers, with at least one confirmed COVID-19 infection and contacts for mental health and diagnoses, psychotropic medication and admissions in the COVID-19 pandemic between 1st March 2020 and 31st November 2021

	DCWs N=15,725		Confirmed COVID-19 infection[a] N= 3,698		Contacts and diagnosis for mental health, psychotropic medication and admissions[a]	
DCW Characteristics	N	%	N	%	N	%
Sex[b]						
Female	13,253	84.3	3,226	24.3	4,685	35.4
Male	2,472	15.7	472	19.1	615	24.9
Age (at registration)[b]						
Under 35 years	4,425	28.1	1,164	26.3	1,542	34.9
35 to <45 years	2,822	17.9	795	28.2	977	34.6
45 to <55 years	3,955	25.2	897	22.7	1,398	35.4
55 years and over	4,523	28.8	842	18.6	1,383	30.6
Ethnicity[b]						
White	15,012	95.5	3,537	23.6	5,128	34.2
Black	142	0.9	35	24.7	22	15.5
Mixed	127	0.8	*	*	*	*
Asian	108	0.7	25	23.2	18	16.7
Other	66	0.4	*	*	*	*
Mixed/Other	193	1.2	42	21.8	46	23.8
Not recorded	270	1.7	59	21.9	86	31.9
* numbers suppressed as <10. Mixed and Other categories are combined.						
Lives with DCW[b]						
No	14,750	93.8	3,444	23.4	4,988	33.8
Yes	975	6.2	254	26.1	312	32.0
Have disability[b]						
No	14141	89.9	3,310	23.4	4,714	33.3
Yes	188	1.2	30	16.0	91	48.4
Not recorded /Prefer not to answer	1,396	8.9	358	25.6	495	35.5
Qualifications as recorded at registration[b]						
Competence confirmed	1982	12.6	438	22.1	646	32.6
Induction Framework	2984	19.0	737	24.7	1,094	36.7
Qualification Level 2	6935	44.1	1,672	24.1	2,328	33.6
Qualification Level 3	3340	21.2	752	22.5	1,077	32.3
Qualification Level 4+	441	2.8	88	20.0	140	31.8
Not recorded	43	0.3	11	25.6	15	32.7
Employment sector as recorded at registration[b]						
Private	6,592	41.9	1,480	22.5	2,154	32.7
Third Sector	4,267	27.1	991	23.2	1,454	34.1
Local Authority-Social Services	3,306	21.0	876	26.5	1,159	35.1
Recruitment/Employment Agency	148	0.9	43	25.0	65	37.8
Health/Other	24	0.2				

For peer review only - http://bmjopen.bmj.com/site/about/guidelines.xhtml

	DCWs N=15,725		Confirmed COVID-19 infection[a] N= 3,698		Contacts and diagnosis for mental health, psychotropic medication and admissions[a]	
DCW Characteristics	N	%	N	%	N	%
Not recorded	1388	8.8	308	22.2	468	33.7
Smoking status[c]						
Non-smoker	6,342	40.3	1,638	25.8	2,076	32.7
Current smoker	4,374	27.8	875	20.0	1,781	40.7
Ex-smoker	3,509	22.3	1,185	23.7	1,443	28.8
Not recorded	1,500	9.5				
Body Mass Index[c]						
Underweight (<18.5)	256	1.6	48	18.8	99	38.7
Healthy weight (18.5-24.9)	3,173	20.2	685	21.6	1,045	32.9
Overweight (25-29.9)	3,652	23.2	847	23.2	1,341	36.7
Obese (≥30)	5,311	33.8	1398	26.3	2,320	43.7
Not recorded	3,333	21.2	720	21.6	495	14.9
Any comorbidity[c,d,e]						
No	8,921	56.7	2,046	22.9	2,095	23.5
Yes	6,804	43.3	1,652	24.3	3,205	47.1
Mental health related illness[f]	3,431	21.8	-	-	-	-
Asthma	2,699	17.2	-	-	-	-
CHD	281	1.8	-	-	-	-
CKD	132	0.8	-	-	-	-
COPD	299	1.9	-	-	-	-
Epilepsy	238	1.5	-	-	-	-
Osteoporotic fracture	462	2.9	-	-	-	-
Stroke	135	0.9	-	-	-	-
Diabetes	824	5.2	-	-	-	-
On COVID-19 shielding list[g]						
No	15,090	96.0	3,569	23.7	5,033	33.4
Yes	635	4.0	129	20.3	267	42.1
Area level characteristics[h,i]						
Resident health board						
Aneurin Bevan	2,819	17.9	758	26.9	980	34.8
Betsi Cadwaladr	3,683	23.4	665	18.1	1,140	31.0
Cardiff and Vale	1,796	11.4	475	26.5	650	36.2
Cwm Taf Morgannwg	2,702	17.2	755	27.9	1,078	39.9
Hywel Dda	1,965	12.5	392	20.0	561	28.6
Powys	662	4.2	104	15.7	105	15.9
Swansea Bay	2,098	13.3	549	26.2	786	37.5
Welsh Index of Multiple Deprivation 2019 quintile[k]						
1 (most deprived)	4,168	26.5	1,143	27.4	1,541	37.0
2	3,970	25.2	991	25.0	1,392	35.1
3	3,301	21.0	697	21.1	1,005	30.5
4	2,554	16.2	503	19.7	780	30.5
5 (most affluent)	1,732	11.0	364	21.0	582	33.6

For peer review only

For peer review only - http://bmjopen.bmj.com/site/about/guidelines.xhtml

	DCWs N=15,725		Confirmed COVID-19 infection[a] N= 3,698		Contacts and diagnosis for mental health, psychotropic medication and admissions[a]	
DCW Characteristics	N	%	N	%	N	%
Rurality classification						
Rural	4,932	31.4	947	19.2	1,435	29.1
Urban	10,793	68.6	2,751	25.5	3,865	35.8

CHD= Coronary Heart Disease; CKD= Coronary Kidney Disease; COPD= Chronic Obstructive Pulmonary Disease.
a between 1st March 2020 to 31st November 2021; b data source = Domiciliary Social Care Workers (DSCW) data and include data that is self-reported by DCWs at 1st April. If missing, then other sources (ONS 2011 Census Wales (CENW), Wales Longitudinal General Practice (WLGP), Patient Episode Dataset for Wales (PEDW), Emergency Department Dataset (EDDS)) were used to impute (at index date=1st March 2020); c data source = WLGP; d data source=PEDW; e most recent occurring between 1st January 2000 and 1st March 2020; f Contacts and diagnoses for mental health and admissions; g people at a higher risk of getting seriously ill from COVID-19;h based on residential address at 1st March 2020; i data source =Welsh Demographic Service Dataset (WDSD); k The Welsh Index of Multiple Deprivation (WIMD) is the official measure of relative deprivation for small areas in Wales. Quintiles are derived by proportioning the derivation score into five equal groups.

Table 2. Key outcomes by pandemic waves

	Short term (Wave 1) 1st March to 31st August 2020		Short term (Wave 1 and 2) 1st March 2020 to 28th February 2021		Long term 1st March 2020 to 30th November 2021	
Outcome	n	%	n	%	n	%
Confirmed COVID-19 infection	182	1.2	2,196	14.0	3,698	23.5
Suspected COVID-19 infection	326	2.1	2,335	14.8	3,822	24.3
Mental health[a]	3641	23.2	4,392	27.9	5,300	33.7
Self-harm	20	0.1	35	0.2	64	0.4
Fit notes	857	5.4	1,551	9.9	2,297	14.6
Non-COVID-19 respiratory infections[b]	126	0.8	300	1.9	467	3.0
All-cause mortality	*	*	*	*	23	0.1

* short term results suppressed as de-identifiable by subtraction from the long term results.
a Contacts and diagnosis for mental health, psychotropic medication and admissions; b Respiratory infections not necessarily recorded as COVID-19 infection.

Suspected COVID-19 infection

Suspected COVID-19 infections increased over the pandemic from 2% in early pandemic to 15% by at the end of February 2021, rising to 24% by the end of November 2021 (Table 2). Examining the prevalence of suspected COVID-19 infection added little to the clinical picture given the relatively widespread availability of COVID-19 testing across the follow-up period and uncertainty regarding the fields that were used (i.e. just having a COVID-19 test was not a good indicator of having COVID-19 infection). No further modelling was therefore conducted.

For peer review only - http://bmjopen.bmj.com/site/about/guidelines.xhtml

Mental health

Contacts and diagnosis for mental health, psychotropic medication, and admissions were 23% in August 2020, rising to 28% and 34% by the end of February and November 2021 respectively (Table 2). The increase in mental health issues over time was also apparent when examining the monthly number of mental health related medication and contacts with GPs; prescribing mental health related medication was recorded in 13% of all DCWs in March 2016 rising to 20% in March 2020, prescriptions increasing on average per month by 17.6 (95% CI: 15.5 to 19.7, p-value <0.001) (Figure 2a). No evidence of a change was observed immediately following the start of the pandemic (March to April 2020) but there was evidence of an increase in prescriptions after April 2020 (compared to the pre–pandemic) with an average monthly increase of 29.3 (20.7 to 38.0, p-value <0.001). Similar patterns were observed for mental health related GP contacts. Mental health related hospital admissions were lower in March 2016 (around 15 admission per month) and significantly increased on average per month by 0.15 (0.06 to 0.25, 0.002) (Figure 2b). There was evidence to suggest a decrease in admissions immediately following the start of the pandemic but increased on average by 0.99 admissions per month (0.44 to 1.53, <0.001), returning to pre-pandemic by the end of the study period.

Variations in mental health contacts were observed across many of the key demographics (e.g., lower in males and older care workers), and consistently higher in care workers with a disability reported at registration, with a comorbidity or shielding (Table 1). There was no variation in mental health contacts for the care workers by their qualification status; care workers in the local authority sector had slightly elevated risk of mental ill-health compared to those employed by the private sector at registration.

Fit notes

The issuing of fit notes as a general marker of medically confirmed illness, increased over the time-period from 5% in August 2020, to 10% by at the end of February and 15% by the end of November 2021. Fit notes were more prevalent in females, those who registered following the induction pathway or held a Level 2 Qualification (compared to those with competency confirmed by a manager), those employed by the Local Authority Social Services at registration (compared to those employed in the private sector), DCWs with a comorbidity or were shielding, and those with a BMI greater than 30 (compared to those with a healthy BMI: 18.5 to 24.9) (supplementary material 5). Unlike confirmed COVID-19 infection, fit notes were more likely in those that were older (aged 45 and over). Both Betsi Cadwaladr and Powys health boards also had a higher rate of fit notes issued for DCWs, whereas Swansea Bay had less (when compared to Aneurin Bevan).

For peer review only - http://bmjopen.bmj.com/site/about/guidelines.xhtml

Deaths

Both all-cause and COVID-19-related mortality involved 0.1% and 0.034% respectively, of the care workers over the longer-term study period (1st March 2020 and 30th November 2021).

Comparison to other UK nations

Up-to-date DCW workforce from Scotland [25] and England [26] show comparable case-mix of DCW populations to (and in some cases identical to) that presented for Wales with respect to age, sex, and ethnicity (supplementary material 6) and we would expect the prevalence of confirmed COVID-19 in these nations to therefore be similar. For domiciliary care workers in England based on a population of 590,000, the impact of mental health illness is around 200,000. However, data from Northern Ireland (NI) showed that DCWs are younger on average (mode 20-29 years) [27], and the prevalence of COVID-19 infection may have been greater in this DCW workforce.

DISCUSSION

Principal findings

Confirmed COVID-19 rates increased from 14% during the first two pandemic waves (1st March 2020 to 28th February 2021) to 24% by the end of November 2021. Infection rates varied by personal characteristics of care workers (lower in males and in older care workers), by where care workers lived (lower in health boards such as Powys, in rural areas, in more affluent areas), and were higher for care workers employed by local authority social services departments compared to staff employed in the private sector. One third (34%) of all care workers required support for a mental health condition, with rates of attending a GP or receiving a relevant prescription higher compared to the four years preceding pandemic onset. Rates in mental health contacts varied by key demographics, and were consistently higher in care workers with a comorbidity, disability or who were shielding. Local authority care workers had a slightly elevated risk of mental health problems compared to those employed by the private sector. The issuing of fit notes as a general marker of medically confirmed illness, increased by 10% over the COVID-19 pandemic and were more prevalent in female DCWs, those with an underlying comorbidity or at a higher risk of getting seriously ill from COVID-19. Unlike confirmed COVID-19 infection, fit notes were more likely in those that were older (aged 45 and over). The mortality rate amongst care workers was no greater than that observed amongst the general population of 15- to 64-year-olds in Wales (which was 0.034%). Data from DCW workforces in England and Scotland showed a similar demographic with respect to age, sex and ethnicity. The

For peer review only - http://bmjopen.bmj.com/site/about/guidelines.xhtml

impact of COVID-19 infection would therefore be similar to that observed in Wales. The NI workforce was younger and therefore we may expect a high prevalence rate.

Strengths and weaknesses

A principal strength of the study was the comprehensive and contemporaneous coverage of the domiciliary care worker population in Wales through linkage to registration data. This contrasts with approaches reliant on census data which may be incomplete, at risk of being out of date or on sample surveys with varying levels of non-response [28,29]. With few records lost to linkage failure or opt-out, the study cohort reflects well the population of DCWs reported by the regulator [30]. Access to EHRs in the SAIL Databank allowed for broader assessment of health than previously reported by ONS, which understandably focused on mortality in the early period of the pandemic [28]. Accessing SAIL data utilised coding developed for existing conditions and novel indications (e.g. COVID-19) enabling rapid application and greater confidence than coding developed from scratch [31][31].

The registration process was introduced by Social Care Wales to meet regulatory rules that came into force from 1st April 2020 [3]. Registration as a domiciliary care worker reflected the role's focus on providing care whereas workers providing support only were not required to register. However, our qualitative interviews revealed greater heterogeneity in the registered workforce than anticipated [4]. Workers in supported shared living homes were registered, as well as workers travelling to care for individuals living in their own home, detail unavailable from registration data alone. In all cases, members of the study cohort are registered as DCWs and despite some potential differences in working role amongst the cohort, common factors are likely to involve close proximity with clients in their domestic setting. Differences may include provision of intimate personal care and assistance with bodily functions by DCWs only. Our inability to further differentiate between workers consistently working in either a care or support only role during the study period is a limitation, especially if this represents a difference in risk exposure.

Administrative records do not usually capture subjective patient reported outcomes or wellbeing concerns. This may be a greater consideration if care workers felt reluctant or less able to present concerns to healthcare professionals during the pandemic, as has been observed in other settings [32]. Both quantitative and qualitative studies, involving a range of community-based health and social care staff provide ample evidence of impacts of the pandemic on the wellbeing of professionals [2,4,33–35]. Findings from our routine data study builds a more complete picture of the health consequences of the pandemic for care workers.

Outcome rates varied considerably based on characteristics and circumstances of care workers, such as age and geographical location. Such differences may not be directly attributable to the working

For peer review only - http://bmjopen.bmj.com/site/about/guidelines.xhtml

conditions of care workers or even directly addressable. Nevertheless, it highlights that risks for care workers remain a vital consideration for employers.

Some methodological caveats include that we could not distinguish between DCWs who did not consult with their GP or where their GP had not contributed data to SAIL. This will result in downward biased GP outcome rates (e.g. if a mental health diagnosis truly occurred then a mental health diagnosis will be misclassified as having not occurred). Secondly, if missing GP data are not at random (e.g., if there is less GP coverage in some regions), it is more likely that we would have missed mental health diagnosis or fit notes from DCWs with certain characteristics, resulting in bias in our parameter estimates (ORs/HRs) for these outcomes. Thirdly, DCWs were followed up for different durations (due to migration in and out of Wales or from contributing/non-contributing GPs, or deaths). Whilst the time to event analyses (and corresponding HRs) account for this by censoring, summary statistics and ORs do not. Lastly, cohort ageing and differences in cohort entry or exit dates would likely lead to upwards bias in the overall trends in the time series analyses.

Findings in context

ONS data from the initial three-month period of the pandemic suggested that mortality rates for individuals employed as care workers and home carers were higher than those found in the general population [28]. However, data on occupational classification was missing for a large proportion dying with some COVID-19 involvement and, even where available, data on occupation may have been incorrect due to role and job changes since census data were provided. Our findings are more in line with the initial estimates provided by a Public Health England survey of DCWs in July 2020, where the rate of confirmed COVID-19 on PCR testing (regardless of symptoms) was low (0.1% (95% CI=0.02 to 0.04%) [29].

A semi-quantitative job exposure matrix (COVID-19-JEM) was developed to estimate the likelihood of workers becoming infected with SARS-CoV-2 in an occupational setting [36]. ‘Home workers’ and ‘home carers’ were assessed as scoring 14 on the matrix, indicating a relatively high risk of exposure due to characteristics inherent in their job. However, the threshold used (13+) would include half of the UK workforce and the matrix’s factors for transmission risk and mitigation could clearly mask a heterogeneity of risk as suggested in our own qualitative work [4]. An analysis of occupational differences in SARS-CoV-2 infection used the UK ONS COVID-19 infection survey (CIS) data for adults aged 20-64 years old [37,38]. The CIS period covered (April 2020 to November 2021) was almost equivalent to that in our own study. Using the four-digit Standard Occupational Classification (SOC) 2010, the study reported an elevated risk for social care staff (adjusting for multiple demographic factors) with an HR of 1.14 (95% CI: 1.04 to 1.24) when compared to non-essential workers. Of the 8,005 social care staff included in the CIS, 690 (8.6%) had at least one positive PCR

For peer review only - http://bmjopen.bmj.com/site/about/guidelines.xhtml

test during the study period. Interestingly, the elevated odds of an infection for social care staff reduced over the time assessed (as it did for healthcare workers), while it persisted for some other occupations such as education. Finally, in comparison, the report found equivocal evidence overall regarding infection rate for healthcare support workers (HR of 1.13; 95% CI: 0.96 to 1.32).

Comparing rates across studies may not always be helpful due to the differences in methods used and the time intervals covered. For example, while the ONS survey used RT-PCR testing, other studies have used other methods (e.g., self-administered point-of-care lateral flow immunoassay testing in REACT-2) [38,39]. Nevertheless, the higher rate of confirmed infection for DCWs in our study compared to social care staff in the ONS survey cannot be attributable to differences in time period covered as they were virtually equivalent. Whilst DCWs in Wales were likely to have tested several times in that time period, so would have participants in the ONS study (the CIS involved monthly testing after an initial baseline set of 5 weekly assessments). The lack of granularity associated with SOC codes and COVID-19-JEM to reflect contamination risk in specific settings is probably a limiting factor in such analyses. Our study sample remained specifically focused on workers registered as DCWs, which may reflect a higher level of occupational risk than for other workers classed as in caring personal services or in social care. In this context, within study population differences (e.g., by sex, gender) are less problematic to interpret especially given the comprehensive coverage of the DCW population and the large numbers of well characterised DCWs included.

Previous research on care worker health outcomes has often focused on musculoskeletal problems and dealing with challenging behaviour [40]. Nevertheless, pre-pandemic work has emphasised some broader harms experienced by a range of community-based home care workers (e.g. infection control) and the benefits that workers derive from their work [41–43]. Sterling and colleagues used US national CDC Behavioral Risk Factor Surveillance Survey data to explore outcomes of home health care workers employed by home care agencies. [41]. Despite role differences between the US sample and domiciliary care workers in Wales, the pre-pandemic levels of mental ill-health are interestingly similar. The reasons for such similarities are unclear and could simply reflect broader population similarities in the prevalence of mental ill-health rather than occupationally driven factors.

Conclusion

This study presents evidence of the direct and indirect impact of the COVID-19 pandemic upon DCWs in Wales. Higher rates of confirmed COVID-19 infection amongst DCWs did not translate into mortality rates greater than for the broader Welsh population. High baseline rates of mental ill-health further increased over time, a burden that fell unevenly across the workforce. It remains to be determined whether any facet of the employment role, such as staff training, occupational risk assessment or testing procedures, may have contributed to these differences or provided an opportunity to intervene. Evidence from our own work and that of others, supports the value of co-

For peer review only - http://bmjopen.bmj.com/site/about/guidelines.xhtml

produced solutions which draw on the direct experiences of care workers to support occupational related well-being [44]. Systemic drivers (e.g. public funding for social care, staffing levels, levels of pay) and situational aspects of the role such as peripatetic working will not change quickly or even at all. With few evidence-based supportive approaches tailored to the circumstances of care workers [45] [46] optimising or innovating approaches to support the UK community of care workers may offer considerable long-term benefits to workforce and clients alike.

For peer review only

Funding

This research was funded by the Economic & Social Research Council (ESRC), as part of UK Research & Innovation's rapid response to COVID-19 (ES/V015206/1).

Research Ethics Approval

The study received approval from the School of Medicine Research Ethics Committee, Cardiff University [SMREC 20/114]. Approval was obtained from the SAIL Information Governance Review Panel (IGRP), Swansea University (Project ID: 1126) and data access was granted to named staff. Approval from the ONS Research Accreditation Panel (ONS RAP) was provided (16/12/2020) and approval from Social Care Wales was provided (26/01/2021). This process was generic and not specific to the OSCAR study.

Competing Interest

The authors declare that they have no conflict of interest and that they satisfy the conditions for authorship.

Data sharing statement: The routine data used in this study are available in the SAIL Databank at Swansea University, Swansea, UK. All proposals to use SAIL data are subject to review by an IGRP. Before any data can be accessed, approval must be given by the IGRP. The IGRP gives careful consideration to each project to ensure proper and appropriate use of SAIL data. When access has been approved, it is gained through a privacy protecting safe haven and remote access system referred to as the SAIL Gateway. SAIL has established an application process to be followed by anyone who would like to access data via SAIL: https://www.saildatabank.com/application-process. This study has been approved by the IGRP as project 1126.

Author contributions: RCJ and MR were co-chief investigators of the study and led the drafting of the main manuscript. MR, RCJ, AJ, FLJ, AA, KH, LBH, and DT conceived and contributed to the design of the study. AA was responsible for managing access to data within the SAIL Databank and advising on constituent data sets. HJ was responsible for data management and preparing data for analysis. FLW was responsible for study management. SS and RCJ were responsible for developing the statistical analysis plan and conducting the main analysis. All authors (MR, RCJ, AJ, FLW, AA, KH, DT, SS, HJ, LBH, HP) contributed to the design and conduct of the study and interpretation of the analyses. All authors were responsible for reviewing and revising drafts of the manuscript and providing final approval.

For peer review only - http://bmjopen.bmj.com/site/about/guidelines.xhtml

Acknowledgements: The Centre for Trials Research receives funding from Health and Care Research Wales and Cancer Research UK. The data acquisition and COVID-19 research was supported by Health Data Research UK [HDR-9006], which receives its funding from HDR UK Ltd, and the ADR Wales programme of work) funded ADR UK (grant ES/S007393/1). The study team gratefully acknowledge the work of the Study Advisory Group for providing independent oversight and input; the Implementation Reference Group for reviewing early findings and co-producing recommendations for short and longer-term outcomes; and all domiciliary care workers who contributed their data for inclusion in the study analyses. This study makes use of anonymised data held in the Secure Anonymised Information Linkage (SAIL) Databank. We would like to acknowledge all the data providers who make anonymised data available for research. We acknowledge the support of Social Care Wales in advising us at different stages of the study, supporting access to the registration data and aiding our reflection on study findings. This is an open access article distributed in accordance with the Creative Commons Attribution 4.0 Unported (CC BY 4.0) license, which permits others to copy, redistribute, remix, transform and build upon this work for any purpose, provided the original work is properly cited, a link to the licence is given, and indication of whether changes were made. See: https://creativecommons.org/licenses/by/4.0/.

view only

References

1 Homecare. Home care facts and stats: number of providers, service users & workforce. 2021.https://www.homecare.co.uk/advice/home-care-facts-and-stats-number-of-providers-service-users-workforce (accessed 15 Jun 2022).

2 Gillen P, Neill RD, Manthorpe J, *et al.* Decreasing Wellbeing and Increasing Use of Negative Coping Strategies: The Effect of the COVID-19 Pandemic on the UK Health and Social Care Workforce. *Epidemiologia* 2022;**3**:26–39. doi:10.3390/epidemiologia3010003

3 Social Care Wales. Why we register. 2022.https://socialcare.wales/registration/why-we-register (accessed 16 Jun 2022).

4 Prout H, Lugg-Widger F V., Brookes-Howell L, *et al.* "I don't mean to be rude, but could you put a mask on while I'm here?" A qualitative study of risks experienced by domiciliary care workers in Wales during the COVID-19 pandemic. *Health Soc Care Community* Published Online First: 24 November 2022. doi:10.1111/hsc.14109

5 Lyons RA, Jones KH, John G, *et al.* The SAIL databank: linking multiple health and social care datasets. *BMC Med Inform Decis Mak* 2009;**9**:3. doi:10.1186/1472-6947-9-3

6 SAIL Databank. COVID-19 Pathology Test Results. 2022.https://data.ukserp.ac.uk/Asset/View/53 (accessed 16 Jun 2022).

7 SAIL Databank. Patient Episode Database for Wales. 2022.https://data.ukserp.ac.uk/Asset/View/15 (accessed 16 Jun 2022).

8 SAIL Databank. Annual District Death Extract. 2022.https://data.ukserp.ac.uk/Asset/View/3 (accessed 16 Jun 2022).

9 SAIL Databank. Primary Care GP Dataset. 2022.https://data.ukserp.ac.uk/Asset/View/17 (accessed 16 Jun 2022).

10 John A, McGregor J, Fone D, *et al.* Case-finding for common mental disorders of anxiety and depression in primary care: an external validation of routinely collected data. *BMC Med Inform Decis Mak* 2016;**16**:35. doi:10.1186/s12911-016-0274-7

11 Rahman MA, Todd C, John A, *et al.* School achievement as a predictor of depression and self-harm in adolescence: linked education and health record study. *The British Journal of Psychiatry* 2018;**212**:215–21. doi:10.1192/bjp.2017.69

12 Clift AK, Coupland CAC, Keogh RH, *et al.* Living risk prediction algorithm (QCOVID) for risk of hospital admission and mortality from coronavirus 19 in adults: national derivation and validation cohort study. *BMJ* 2020;:m3731. doi:10.1136/bmj.m3731

13 Thomas KH, Davies N, Metcalfe C, *et al.* Validation of suicide and self-harm records in the Clinical Practice Research Datalink. *Br J Clin Pharmacol* 2013;**76**:145–57. doi:10.1111/bcp.12059

14 Marchant A, Turner S, Balbuena L, *et al.* Self-harm presentation across healthcare settings by sex in young people: an e-cohort study using routinely collected linked healthcare data in Wales, UK. *Arch Dis Child* 2020;**105**:347–54. doi:10.1136/archdischild-2019-317248

For peer review only - http://bmjopen.bmj.com/site/about/guidelines.xhtml

15 London School of Hygiene and Tropical Medicine Data Compass. Data Compass. https://datacompass.lshtm.ac.uk/id/eprint/2193/ (accessed 16 Jun 2022).

16 London School of Hygiene and Tropical Medicine. Data Compass. https://datacompass.lshtm.ac.uk/id/eprint/2037/1/copd_hes.txt (accessed 16 Jun 2022).

17 London School of Hygiene and Tropical Medicine. Data Compass. https://datacompass.lshtm.ac.uk/id/eprint/940 (accessed 16 Jun 2022).

18 Social Care Wales. Qualifications needed. 2022.https://socialcare.wales/registration/qualifications-needed#section-29926-anchor (accessed 8 Jul 2022).

19 Benchimol EI, Smeeth L, Guttmann A, *et al.* The REporting of studies Conducted using Observational Routinely-collected health Data (RECORD) Statement. *PLoS Med* 2015;**12**:e1001885. doi:10.1371/journal.pmed.1001885

20 Elm E von, Altman DG, Egger M, *et al.* Strengthening the reporting of observational studies in epidemiology (STROBE) statement: guidelines for reporting observational studies. *BMJ* 2007;**335**:806–8. doi:10.1136/bmj.39335.541782.AD

21 IBM Corp. Db2 for z/OS (Version 11.1). 2022.

22 R Core Team. R: A language and environment for statistical computing. R Foundation for Statistical Computing, Vienna, Austria,. Published Online First: 2022.www.R-project.org (accessed 8 Jul 2022).

23 Wickham H, Averick M, Bryan J, *et al.* Welcome to the Tidyverse. *J Open Source Softw* 2019;**4**:1686. doi:10.21105/joss.01686

24 StataCorp. Stata Statistical Software: Release 17. College Station, TX: StataCorp LLC. 2021.

25 Scottish Social Services Council. Adults' services workforce tables - 2019 | Scottish Social Services Workforce Data (). 2019. Adults' services workforce tables - 2019 | Scottish Social Services Workforce Data (sssc.uk.com) https://data.sssc.uk.com/data-publications/248-adults-services-workforce-in-scotland-tables-2019 (accessed 16 Jun 2022).

26 Skills for Care. The state of the adult social care sector and workforce in England. Published Online First: 2022.https://www.skillsforcare.org.uk/adult-social-care-workforce-data/Workforce-intelligence/publications/national-information/The-state-of-the-adult-social-care-sector-and-workforce-in-England.aspx (accessed 16 Jun 2022).

27 Department of Health. Rapid learning review of domiciliary care in Northern Ireland. https://www.health-ni.gov.uk/rli-dom-care (accessed 16 Jun 2022).

28 Office for National Statistics. Coronavirus (COVID-19) related deaths by occupation, England and Wales. https://www.ons.gov.uk/peoplepopulationandcommunity/healthandsocialcare/causesofdeath/datasets/coronaviruscovid19relateddeathsbyoccupationenglandandwales (accessed 22 Dec 2020).

29 Public Health England. Pilot point prevalence survey of COVID-19 among domiciliary care staff in England. 2020. https://www.gov.uk/government/publications/covid-19-prevalence-survey-domiciliary-care-staff-in-england (accessed 22 Dec 2020).

For peer review only - http://bmjopen.bmj.com/site/about/guidelines.xhtml

30 Social Care Wales. Domiciliary Care Workers on the Register. 2020. 2020. https://socialcare.wales/cms_assets/file-uploads/Domiciliary-Care-Workers-on-the-Register.pdf (accessed 16 Jun 2022).

31 Lyons J, Akbari A, Torabi F, *et al.* Understanding and responding to COVID-19 in Wales: protocol for a privacy-protecting data platform for enhanced epidemiology and evaluation of interventions. *BMJ Open* 2020;**10**:e043010. doi:10.1136/bmjopen-2020-043010

32 Quinn-Scoggins HD, Cannings-John R, Moriarty Y, *et al.* Cancer symptom experience and help-seeking behaviour during the COVID-19 pandemic in the UK: a cross-sectional population survey. *BMJ Open* 2021;**11**:e053095. doi:10.1136/bmjopen-2021-053095

33 McFadden P, Neill RD, Moriarty J, *et al.* A Cross-Sectional Examination of the Mental Wellbeing, Coping and Quality of Working Life in Health and Social Care Workers in the UK at Two Time Points of the COVID-19 Pandemic. *Epidemiologia* 2021;**2**:227–42. doi:10.3390/epidemiologia2030017

34 Aughterson H, McKinlay AR, Fancourt D, *et al.* Psychosocial impact on frontline health and social care professionals in the UK during the COVID-19 pandemic: a qualitative interview study. *BMJ Open* 2021;**11**:e047353. doi:10.1136/bmjopen-2020-047353

35 Markkanen P, Brouillette N, Quinn M, *et al.* “It changed everything”: The Safe Home Care qualitative study of the COVID-19 pandemic’s impact on home care aides, clients, and managers. *BMC Health Serv Res* 2021;**21**:1055. doi:10.1186/s12913-021-07076-x

36 Oude Hengel KM, Burdorf A, Pronk A, *et al.* Exposure to a SARS-CoV-2 infection at work: development of an international job exposure matrix (COVID-19-JEM). *Scand J Work Environ Health* 2022;**48**:61–70. doi:10.5271/sjweh.3998

37 Rhodes S, Wilkinson J, Pearce N, *et al.* Occupational differences in SARS-CoV-2 infection: analysis of the UK ONS COVID-19 infection survey. *J Epidemiol Community Health (1978)* 2022;**76**:841–6. doi:10.1136/jech-2022-219101

38 Pouwels KB, House T, Pritchard E, *et al.* Community prevalence of SARS-CoV-2 in England from April to November, 2020: results from the ONS Coronavirus Infection Survey. *Lancet Public Health* 2021;**6**:e30–8. doi:10.1016/S2468-2667(20)30282-6

39 Whitaker M, Elliott J, Chadeau-Hyam M, *et al.* Persistent COVID-19 symptoms in a community study of 606,434 people in England. *Nat Commun* 2022;**13**:1957. doi:10.1038/s41467-022-29521-z

40 Health and Safety Executive. Domiciliary care provided in people’s own homes. https://www.hse.gov.uk/healthservices/domiciliary-care.htm (accessed 16 Jun 2022).

41 Sterling MR, Li J, Cho J, *et al.* Prevalence and Predictors of Home Health Care Workers’ General, Physical, and Mental Health: Findings From the 2014–2018 Behavioral Risk Factor Surveillance System. *Am J Public Health* 2021;**111**:2239–50. doi:10.2105/AJPH.2021.306512

42 Denton MA, Zeytinoğlu IU, Davies S. Working in Clients’ Homes: The Impact on the Mental Health and Well-Being of Visiting Home Care Workers. *Home Health Care Serv Q* 2002;**21**:1–27. doi:10.1300/J027v21n01_01

For peer review only - http://bmjopen.bmj.com/site/about/guidelines.xhtml

43 Quinn MM, Markkanen PK, Galligan CJ, *et al.* Occupational health of home care aides: results of the safe home care survey. *Occup Environ Med* 2016;**73**:237–45. doi:10.1136/oemed-2015-103031

44 Hayes L TAWH. Care and support workers' perceptions of health and safetyissues in socialcareduring the COVID-19 pandemic. Initial . 2020. https://media.www.kent.ac.uk/se/11148/CareworkersHealthandSafetyreport15042.pdf (accessed 16 Jun 2022).

45 Olson R, Thompson S v., Elliot DL, *et al.* Safety and Health Support for Home Care Workers: The COMPASS Randomized Controlled Trial. *Am J Public Health* 2016;**106**:1823–32. doi:10.2105/AJPH.2016.303327

46 Indregard A-MR, Knardahl S, Emberland JS, *et al.* Effectiveness of the Labour Inspection Authority's regulatory tools for work environment and employee health: study protocol for a cluster-randomised controlled trial among Norwegian home-care workers. *BMJ Open* 2019;**9**:e031226. doi:10.1136/bmjopen-2019-031226

For peer review only

Figure 1: Cumulative probabilities of confirmed COVID-19 infection over time by employment sector for domiciliary care workers

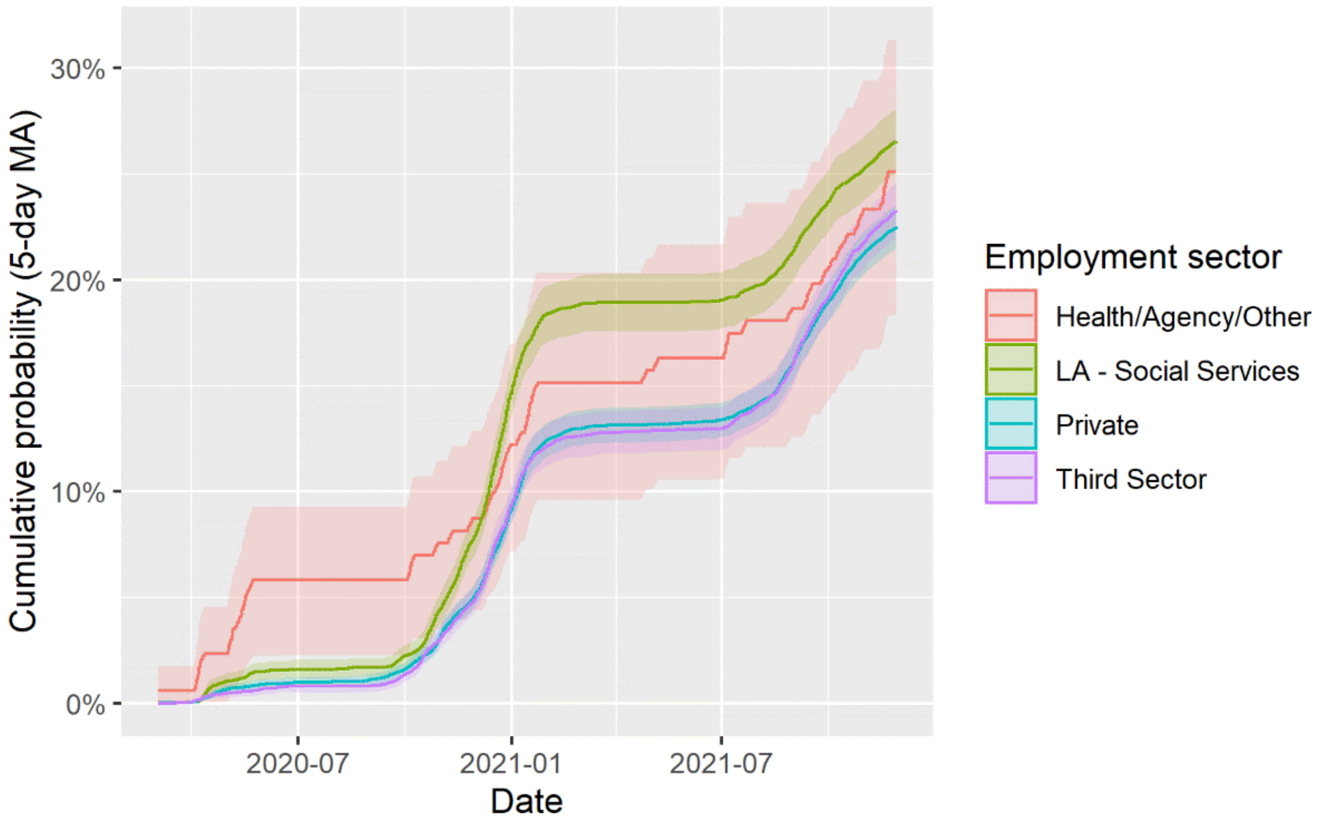

MA=moving average

view only

Figure 2a: Changes over time (March 2016-October 2021) in mental health related hospital admissions for domiciliary care workers

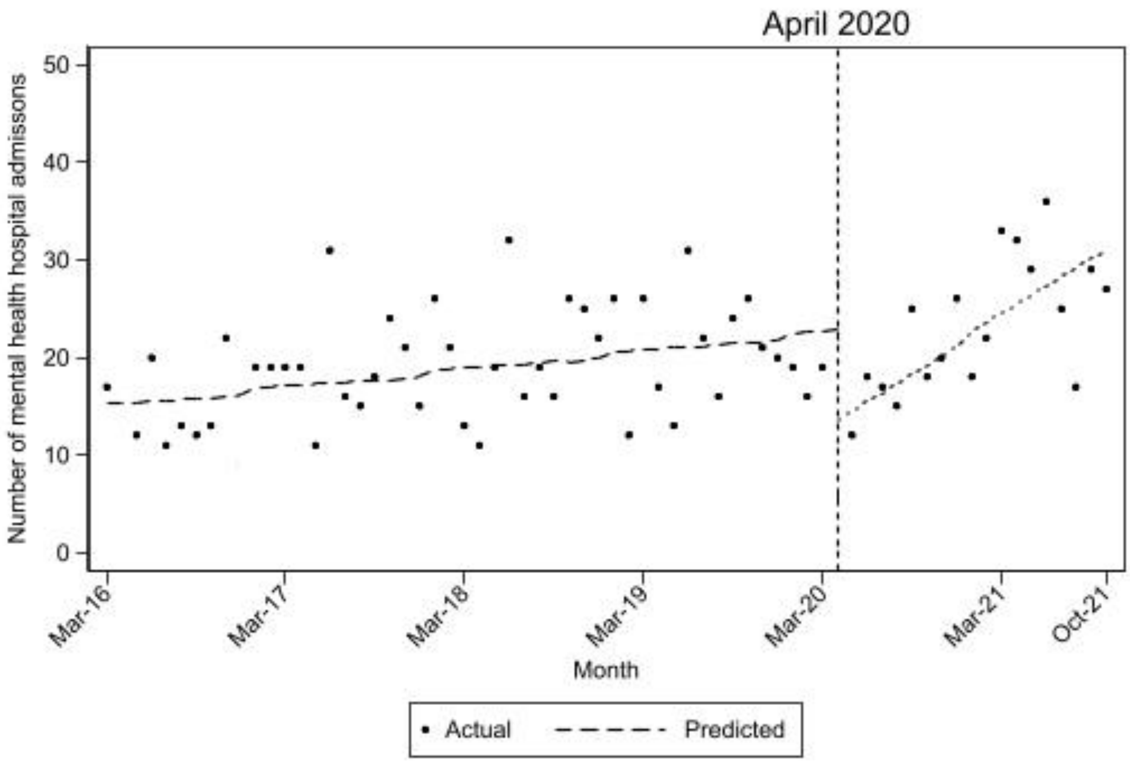

Figure 2b: Changes over time (March 2016-October 2021) in mental health related GP prescriptions for domiciliary care workers

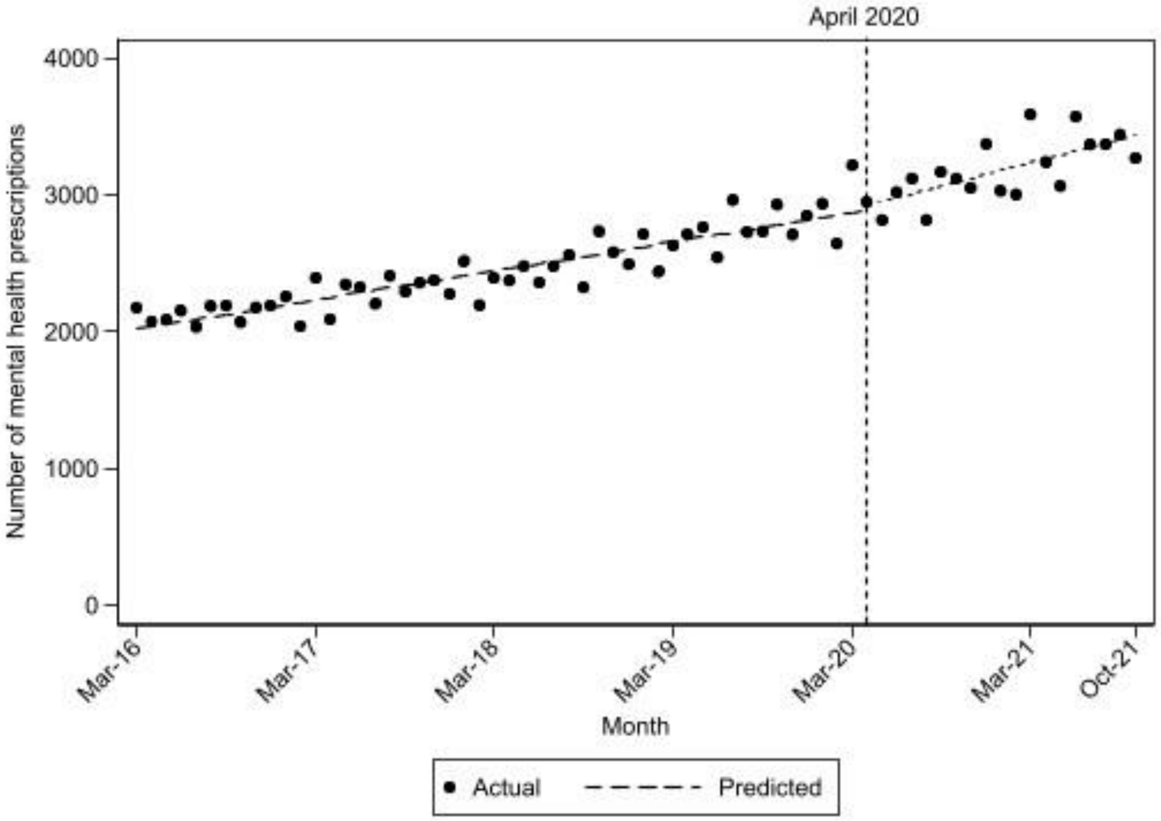

For peer review only

Supplementary material

For peer review only

For peer review only - http://bmjopen.bmj.com/site/about/guidelines.xhtml

Supplementary material 1: Data sources used in the OSCAR study

SAIL acronym	Name	Description	Meta-data link
ADDE	Annual District Death Extract	Monthly register of all deaths relating to Welsh residents, including those that died out of Wales from the Office for National Statistics (ONS).	https://web.www.healthdatagateway.org/dataset/15cf4241-abad-4dcc-95b0-8cd7c02be999
CENW	ONS 2011 Census Wales	A census in the UK is a count of all people and households. The census data held in SAIL was from the census held on 27 March 2011.	https://web.www.healthdatagateway.org/dataset/fc24482c-0f9b-445f-af7a-8598627c3b15
DSCW	Domiciliary Social Care Worker Dataset	Under the Care Standards Act 2000, SCW maintain a register of DCWs in Wales. The DSCW data was transferred securely from SCW to the SAIL Databank and made available as a core-restricted data source.	NA
EDDS	Emergency Department Dataset	Data captured for all NHS Wales Accident and Emergency department attendances. Data are collected and coded at each hospital	https://web.www.healthdatagateway.org/dataset/75c4dcb8-33bf-43f4-b2bb-db51b6621b2c
PATD	COVID-19 Test results	Daily update of all pillar 1 and pillar 2 PCR tests administered and associated results for Welsh residents and those receiving a test in Wales	https://web.www.healthdatagateway.org/dataset/f5f6d882-163d-4ef1-a53e-000fba409480
PEDW	Patient Episode Dataset for Wales	Weekly update of all inpatient and day case activity undertaken in NHS Wales plus data on Welsh residents treated in English Trusts	https://web.www.healthdatagateway.org/dataset/4c33a5d2-164c-41d7-9797-dc2b008cc852
WDSD	Welsh Demographic Service Dataset	Weekly update of all individuals registered with a Welsh general practice, inc. address and practice history	https://web.www.healthdatagateway.org/dataset/8a8a5e90-b0c6-4839-bcd2-c69e6e8dca6d
WLGP	Wales Longitudinal General Practice	Monthly update of all attendance and clinical information for SAIL submitting general practices (~80% national coverage)	https://web.www.healthdatagateway.org/dataset/33fc3ffd-aa4c-4a16-a32f-0c900aaea3d2

For peer review only - http://bmjopen.bmj.com/site/about/guidelines.xhtml

Supplementary material 2: Coding lists for OSCAR outcomes

Primary outcome

Confirmed-COVID: Confirmed COVID-19 will be defined as the earliest of the following events:

- Positive polymerase chain reaction (PCR) test result (Source: PATD);
- Hospital admission (main or secondary diagnostic ICD-10 code of U07.1 position in the admitted patient care component of PEDW);
- Death registration including a diagnosis ICD-10 code U07.1 (as underlying or contributing cause) from COVID-19 (Source: ADDE);
- COVID-19 diagnosis Read code (Source: WLGP).

TYPE	CODE	DESCRIPTION
ICD-10	U071	COVID-19 virus identified
READ	A795.	Coronavirus infection
READ	A7951	Disease caused by 2019-nCoV (novel coronavirus)
READ	A7952	COVID-19 confirmed by laboratory test
READ	A7953	COVID-19 confirmed using clinical diagnostic criteria

Secondary outcomes

Suspected COVID-19

We will ascertain DCWs with a suspected COVID-19 diagnosis taking the earliest of:
- Negative PCR or serology/antigen test from the COVID-19 test data (Source: PATD);
- Diagnosis ICD-10 code (U07.2) main or secondary diagnostic code position (Source: PEDW);
- Suspected COVID-19 Read codes (Source: WLGP).

TYPE	CODE	DESCRIPTION
ICD-10	U072	COVID-19 virus not identified
READ	1JX..	Suspected coronavirus infection
READ	1JX1.	Suspected coronavirus infection
READ	65PW.	Coronavirus contact
READ	65PW1	Exposure to 2019-nCoV (novel coronavirus) infection

Mental Health

(A) Hospital admissions (ICD-10)

CODE	DESCRIPTION
F20	Schizophrenia
F200	Paranoid schizophrenia
F201	Hebephrenic schizophrenia
F202	Catatonic schizophrenia
F203	Undifferentiated schizophrenia
F204	Post-schizophrenic depression

For peer review only - http://bmjopen.bmj.com/site/about/guidelines.xhtml

CODE	DESCRIPTION
F205	Residual schizophrenia
F206	Simple schizophrenia
F208	Other schizophrenia
F209	Schizophrenia, unspecified
F21X	Schizotypal disorder
F22	Persistent delusional disorders
F220	Delusional disorder
F228	Other persistent delusional disorders
F229	Persistent delusional disorder, unspecified
F23	Acute and transient psychotic disorders
F230	Acute polymorphic psychotic disorder without symptoms of schizophrenia
F231	Acute polymorphic psychotic disorder with symptoms of schizophrenia
F232	Acute schizophrenia-like psychotic disorder
F233	Other acute predominantly delusional psychotic disorders
F238	Other acute and transient psychotic disorders
F239	Acute and transient psychotic disorder, unspecified
F24X	Induced delusional disorder
F25	Schizoaffective disorders
F250	Schizoaffective disorder, manic type
F251	Schizoaffective disorder, depressive type
F252	Schizoaffective disorder, mixed type
F258	Other schizoaffective disorders
F259	Schizoaffective disorder, unspecified
F28X	Other nonorganic psychotic disorders
F29X	Unspecified nonorganic psychosis
F30	Manic episode
F300	Hypomania
F301	Mania without psychotic symptoms
F302	Mania with psychotic symptoms
F308	Other manic episodes
F309	Manic episode, unspecified
F31	Bipolar affective disorder
F310	Bipolar affective disorder, current episode hypomanic
F311	Bipolar affective disorder, current episode manic without psychotic symptoms
F312	Bipolar affective disorder, current episode manic with psychotic symptoms
F313	Bipolar affective disorder, current episode mild or moderate depression
F314	Bipolar affective disorder, current episode severe depression without psychotic symptoms
F315	Bipolar affective disorder, current episode severe depression with psychotic symptoms
F316	Bipolar affective disorder, current episode mixed
F317	Bipolar affective disorder, currently in remission
F318	Other bipolar affective disorders
F319	Bipolar affective disorder, unspecified
F32	Depressive episode
F320	Mild depressive episode

For peer review only - http://bmjopen.bmj.com/site/about/guidelines.xhtml

CODE	DESCRIPTION
F321	Moderate depressive episode
F322	Severe depressive episode without psychotic symptoms
F323	Severe depressive episode with psychotic symptoms
F328	Other depressive episodes
F329	Depressive episode, unspecified
F33	Recurrent depressive disorder
F330	Recurrent depressive disorder, current episode mild
F331	Recurrent depressive disorder, current episode moderate
F332	Recurrent depressive disorder, current episode severe without psychotic symptoms
F333	Recurrent depressive disorder, current episode severe with psychotic symptoms
F334	Recurrent depressive disorder, currently in remission
F338	Other recurrent depressive disorders
F339	Recurrent depressive disorder, unspecified

(B) GP contacts and prescriptions (Read)

CODE	DESCRIPTION
146D.	H/O: manic depressive disorder
1B12.	Nerves - nervousness
1B13.	Anxiousness
1B17.	Depressed
1B1U.	Symptoms of depression
1BH3.	Paranoid ideation
1BQ..	Loss of capacity for enjoyment
1BT..	Depressed mood
1BU..	Loss of hope for the future
2257	O/E - depressed
2258	O/E - anxious
2259	O/E - nervous
66580	Antidepressant drug treatment changed
66590	Antidepressant drug treatment started
665A0	Antidepressant drug treatment stopped
d1...	HYPNOTICS
d11..	CHLORAL HYDRATE
d111.	*CHLORAL 500mg/5mL mixture
d112.	CHLORAL PAEDIATRIC 200mg/5mL elixir
d113.	*NOCTEC 500mg capsules
d114.	CHLORAL HYDRATE 500mg capsules
d115.	*WELLDORM 143mg/5mL elixir
d116.	*WELLDORM tablets
d117.	CHLORAL HYDRATE 414mg tablets
d118.	CHLORAL HYDRATE 143mg/5mL elixir
d119.	CHLORAL HYDRATE 500mg/5mL syrup
d11A.	SOMNWELL 707mg tablets

For peer review only - http://bmjopen.bmj.com/site/about/guidelines.xhtml

CODE	DESCRIPTION
d12..	CLOMETHIAZOLE EDISYLATE [HYPNOTIC]
d121.	*HEMINEVRIN 192mg capsules
d122.	*HEMINEVRIN 250mg/5mL syrup
d123.	*HEMINEVRIN 8mg/mL infusion
d12v.	CLOMETHIAZOLE(base) 192mg capsules
d12w.	CLOMETHIAZOLE EDISILATE 250mg/5mL syrup
d12z.	CHLORMETHIAZOLE EDISYLATE 8mg/mL infusion
d13..	*DICHLORALPHENAZONE
d131.	*WELLDORM 650mg tablets
d132.	*WELLDORM 225mg/5mL elixir
d13y.	DICHLORALPHENAZONE 650mg tablets
d13z.	DICHLORALPHENAZONE 225mg/5mL elixir
d14..	*FLUNITRAZEPAM
d141.	*ROHYPNOL 1mg tablets
d14z.	*FLUNITRAZEPAM 1mg tablets
d15..	FLURAZEPAM
d151.	DALMANE 15mg capsules
d152.	DALMANE 30mg capsules
d153.	*PAXANE 15mg capsules
d154.	*PAXANE 30mg capsules
d15y.	FLURAZEPAM 15mg capsules
d15z.	FLURAZEPAM 30mg capsules
d16..	LOPRAZOLAM
d161.	LOPRAZOLAM 1mg tablets
d162.	*DORMONOCT 1mg tablets
d17..	LORMETAZEPAM
d171.	LORMETAZEPAM 500micrograms tablets
d172.	LORMETAZEPAM 1mg tablets
d173.	NOCTAMID 500micrograms tablets
d174.	*NOCTAMID 1mg tablets
d18..	NITRAZEPAM
d181.	*NITRAZEPAM 5mg capsules
d182.	NITRAZEPAM 5mg tablets
d183.	*NITRAZEPAM 10mg tablets
d184.	NITRAZEPAM 2.5mg/5mL mixture
d185.	*MOGADON 5mg capsules
d186.	MOGADON 5mg tablets
d187.	*NITRADOS 5mg tablets
d188.	*NOCTESED 5mg tablets
d189.	REMNOS 5mg tablets
d18a.	*REMNOS 10mg tablets
d18b.	*SOMNITE 5mg tablets
d18c.	*SOMNITE 2.5mg/5mL mixture
d18d.	*SUREM 5mg capsules
d18e.	*UNISOMNIA 5mg tablets

For peer review only - http://bmjopen.bmj.com/site/about/guidelines.xhtml

CODE	DESCRIPTION
d18f.	NITRAZEPAM 5mg/5mL suspension
d19..	PROMETHAZINE HCL [HYPNOTIC] see section c8i..
d1a..	TEMAZEPAM [HYPNOTIC]
d1a1.	*TEMAZEPAM 10mg capsules
d1a2.	*TEMAZEPAM 15mg capsules
d1a3.	*TEMAZEPAM 20mg capsules
d1a4.	*TEMAZEPAM 30mg capsules
d1a5.	*TEMAZEPAM 10mg/5mL elixir
d1a6.	*NORMISON 10mg capsules
d1a7.	*NORMISON 20mg capsules
d1a8.	*TEMAZEPAM PLANPAK capsules
d1a9.	TEMAZEPAM 10mg tablets
d1aa.	TEMAZEPAM 20mg tablets
d1ab.	TEMAZEPAM GELTHIX 10mg capsules
d1ac.	TEMAZEPAM GELTHIX 20mg capsules
d1ad.	TEMAZEPAM GELTHIX 15mg capsules
d1ae.	TEMAZEPAM GELTHIX 30mg capsules
d1af.	TEMAZEPAM GEL FILLED 10mg capsules
d1ag.	TEMAZEPAM GEL FILLED 20mg capsules
d1ah.	TEMAZEPAM GEL FILLED 15mg capsules
d1ai.	TEMAZEPAM GEL FILLED 30mg capsules
d1aj.	TEMAZEPAM 10mg/sachet oral solution
d1ak.	TEMAZEPAM 20mg/sachet oral solution
d1al.	EUHYPNOS 10mg/sachet oral solution
d1am.	EUHYPNOS 20mg/sachet oral solution
d1an.	EUHYPNOS 10mg/5mL oral solution
d1ao.	TEMAZEPAM 10mg/5mL sugar free oral solution
d1b..	*TRIAZOLAM
d1b1.	TRIAZOLAM 125microgram tablets
d1b2.	TRIAZOLAM 250microgram tablets
d1b3.	*HALCION 125microgram tablets
d1b4.	*HALCION 250microgram tablets
d1c..	TRICLOFOS SODIUM
d1c1.	*TRICLOFOS 500mg/5mL liquid
d1d..	ZOPICLONE
d1d1.	ZOPICLONE 7.5mg tablets
d1d2.	ZIMOVANE 7.5mg tablets
d1d3.	ZOPICLONE 3.75mg tablets
d1d4.	ZIMOVANE LS 3.75mg tablets
d1d5.	*ZILEZE 7.5 tablets
d1d6.	*ZILEZE 3.75 tablets
d1e..	*GLUTETHIMIDE [no drugs here]
d1f..	ZOLPIDEM
d1f1.	STILNOCT 5mg tablets
d1f2.	ZOLPIDEM TARTRATE 5mg tablets

For peer review only

CODE	DESCRIPTION
d1f3.	ZOLPIDEM TARTRATE 10mg tablets
d1f4.	STILNOCT 10mg tablets
d1g..	ZALEPLON
d1g1.	SONATA 5mg capsules
d1g2.	SONATA 10mg capsules
d1gy.	ZALEPLON 5mg capsules
d1gz.	ZALEPLON 10mg capsules
d1h..	MELATONIN
d1h1.	CIRCADIN 2mg m/r tablets
d1hz.	MELATONIN 2mg m/r tablets
d21..	DIAZEPAM [ANXIOLYTIC]
d22..	ALPRAZOLAM
d23..	BROMAZEPAM
d24..	CHLORDIAZEPOXIDE
d25..	CHLORMEZANONE
d26..	CLOBAZAM
d27..	CLORAZEPATE DIPOTASSIUM
d28..	HYDROXYZINE HCL [ANXIOLYTIC]
d29..	*KETAZOLAM
d2a..	LORAZEPAM [ANXIOLYTIC]
d2b..	*MEDAZEPAM
d2c..	MEPROBAMATE
d2d..	OXAZEPAM
d2f..	BUSPIRONE HYDROCHLORIDE
d2g..	FLUMAZENIL
d7...	TRICYCLIC ANTIDEPRESSANTS
d71..	AMITRIPTYLINE HYDROCHLORIDE [ANTIDEPRESSANT]
d711.	AMITRIPTYLINE 10mg tablets
d712.	AMITRIPTYLINE 25mg tablets
d713.	AMITRIPTYLINE 50mg tablets
d714.	*DOMICAL 10mg tablets
d715.	*DOMICAL 25mg tablets
d716.	*DOMICAL 50mg tablets
d717.	ELAVIL 10mg tablets
d718.	ELAVIL 25mg tablets
d719.	*LENTIZOL 25mg m/r capsules
d71a.	*LENTIZOL 50mg m/r capsules
d71b.	*TRYPTIZOL 75mg m/r capsules
d71c.	*TRYPTIZOL 10mg tablets
d71d.	*TRYPTIZOL 25mg tablets
d71e.	*TRYPTIZOL 50mg tablets
d71f.	*TRYPTIZOL 10mg/5mL syrup
d71g.	TRYPTIZOL 100mg/10mL injection
d71h.	AMITRIPTYLINE 25mg/5mL sugar free solution
d71i.	AMITRIPTYLINE 50mg/5mL sugar free solution

For peer review only - http://bmjopen.bmj.com/site/about/guidelines.xhtml

CODE	DESCRIPTION
d71u.	AMITRIPTYLINE 25mg m/r capsules
d71v.	AMITRIPTYLINE 50mg m/r capsules
d71w.	AMITRIPTYLINE 75mg m/r capsules
d71y.	*AMITRIPTYLINE 10mg/5mL syrup
d71z.	AMITRIPTYLINE HYDROCHLORIDE 100mg/10mL injection
d72..	*BUTRIPTYLINE
d721.	*EVADYNE 25mg tablets
d722.	*EVADYNE 50mg tablets
d72y.	*BUTRIPTYLINE 25mg tablets
d72z.	*BUTRIPTYLINE 50mg tablets
d73..	CLOMIPRAMINE HYDROCHLORIDE
d731.	*ANAFRANIL 10mg capsules
d732.	*ANAFRANIL 25mg capsules
d733.	*ANAFRANIL 50mg capsules
d734.	*ANAFRANIL 25mg/5mL syrup
d735.	*ANAFRANIL 25mg/2mL injection
d736.	*ANAFRANIL SR 75mg m/r tablets
d737.	*TRANQUAX 10mg capsules
d738.	*TRANQUAX 25mg capsules
d739.	TRANQUAX 50mg capsules
d73r.	*CLOMIPRAMINE HCL 50mg tablets
d73s.	*CLOMIPRAMINE HCL 10mg tablets
d73t.	*CLOMIPRAMINE HCL 25mg tablets
d73u.	CLOMIPRAMINE HCL 10mg capsules
d73v.	CLOMIPRAMINE HCL 25mg capsules
d73w.	CLOMIPRAMINE HCL 50mg capsules
d73x.	CLOMIPRAMINE HYDROCHLORIDE 25mg/5mL syrup
d73y.	CLOMIPRAMINE HCL 25mg/2mL injection
d73z.	CLOMIPRAMINE HCL 75mg m/r tabs
d74..	DESIPRAMINE HYDROCHLORIDE
d741.	*PERTOFRAN 25mg tablets
d74z.	DESIPRAMINE HYDROCHLORIDE 25mg tablets
d75..	DOSULEPIN HYDROCHLORIDE
d751.	PROTHIADEN 25mg capsules
d752.	PROTHIADEN 75mg tablets
d753.	*PREPADINE 25mg capsules
d754.	*PREPADINE 75mg tablets
d755.	*DOTHAPAX 25mg capsules
d756.	*DOTHAPAX 75mg tablets
d759.	*THADEN 25mg capsules
d75A.	*THADEN 75mg tablets
d75y.	DOSULEPIN HYDROCHLORIDE 25mg capsules
d75z.	DOSULEPIN HYDROCHLORIDE 75mg tablets
d76..	DOXEPIN
d761.	*SINEQUAN 10mg capsules x56CP

For peer review only - http://bmjopen.bmj.com/site/about/guidelines.xhtml

CODE	DESCRIPTION
d762.	*SINEQUAN 25mg capsules x28CP
d763.	*SINEQUAN 50mg capsules x28CP
d764.	*SINEQUAN 75mg capsules x28CP
d765.	*SINEPIN 25mg capsules
d766.	*SINEPIN 50mg capsules
d76w.	*DOXEPIN 10mg capsules
d76x.	DOXEPIN 25mg capsules
d76y.	DOXEPIN 50mg capsules
d76z.	*DOXEPIN 75mg capsules
d77..	IMIPRAMINE HYDROCHLORIDE [ANTIDEPRESSANT]
d771.	IMIPRAMINE 10mg tabs
d772.	IMIPRAMINE 25mg tablets
d773.	*PRAMINIL 10mg tablets
d774.	*PRAMINIL 25mg tablets
d775.	*TOFRANIL 10mg tablets
d776.	*TOFRANIL 25mg tablets
d777.	*TOFRANIL 25mg/5mL syrup
d77w.	IMIPRAMINE HYDROCHLORIDE 25mg/5mL oral solution
d77x.	IMIPRAMINE HYDROCHLORIDE 10mg tablets
d77y.	IMIPRAMINE HYDROCHLORIDE 25mg tablets
d77z.	IMIPRAMINE HYDROCHLORIDE 25mg/5mL syrup
d78..	IPRINDOLE
d781.	*PRONDOL 15mg tablets
d782.	*PRONDOL 30mg tablets
d78y.	*IPRINDOLE 15mg tablets
d78z.	*IPRINDOLE 30mg tablets
d79..	LOFEPRAMINE
d791.	*GAMANIL 70mg tablets x56CP
d792.	LOMONT 70mg/5mL sugar free suspension
d793.	*FEPRAPAX 70mg tablets
d794.	*GAMANIL 70mg tablets
d79y.	LOFEPRAMINE 70mg/5mL sugar free suspension
d79z.	LOFEPRAMINE 70mg tablets
d7a..	MAPROTILINE HYDROCHLORIDE
d7a1.	*LUDIOMIL 10mg tablets
d7a2.	*LUDIOMIL 25mg tablets
d7a3.	*LUDIOMIL 50mg tablets
d7a4.	*LUDIOMIL 75mg tablets x28CP
d7aw.	MAPROTILINE HYDROCHLORIDE 10mg tablets
d7ax.	MAPROTILINE HYDROCHLORIDE 25mg tablets
d7ay.	MAPROTILINE HYDROCHLORIDE 50mg tablets
d7az.	MAPROTILINE HYDROCHLORIDE 75mg tablets
d7b..	MIANSERIN HYDROCHLORIDE
d7b1.	MIANSERIN 10mg tablets
d7b2.	MIANSERIN 20mg tablets

For peer review only - http://bmjopen.bmj.com/site/about/guidelines.xhtml

CODE	DESCRIPTION
d7b3.	MIANSERIN 30mg tablets
d7b4.	*BOLVIDON 10mg tablets
d7b5.	*BOLVIDON 20mg tablets
d7b6.	*BOLVIDON 30mg tablets
d7b7.	*NORVAL 10mg tablets
d7b8.	*NORVAL 20mg tablets
d7b9.	*NORVAL 30mg tablets
d7c..	NORTRIPTYLINE
d7c1.	*ALLEGRON 10mg tablets
d7c2.	*ALLEGRON 25mg tablets
d7c3.	*AVENTYL 10mg capsules
d7c4.	*AVENTYL 25mg capsules
d7c5.	*AVENTYL 10mg/5mL liquid
d7c6.	NORTRIPTYLINE 10mg tablets
d7c7.	*NORTRIPTYLINE 10mg/5mL liquid
d7c8.	NORTRIPTYLINE 25mg tablets
d7c9.	*NORTRIPTYLINE 10mg capsules
d7cy.	*NORTRIPTYLINE 25mg capsules
d7d..	PROTRIPTYLINE HYDROCHLORIDE
d7d1.	*CONCORDIN 5mg tablets
d7d2.	*CONCORDIN 10mg tablets
d7d3.	PROTRIPTYLINE HYDROCHLORIDE 5mg tablets
d7d4.	PROTRIPTYLINE HYDROCHLORIDE 10mg tablets
d7e..	TRAZODONE HYDROCHLORIDE
d7e1.	MOLIPAXIN 50mg capsules x84CP
d7e2.	MOLIPAXIN 100mg capsules x56CP
d7e3.	*MOLIPAXIN 50mg/5mL liquid
d7e4.	MOLIPAXIN 150mg tablets x28CP
d7e5.	TRAZODONE HYDROCHLORIDE 150mg tablets
d7e6.	MOLIPAXIN CR 150mg m/r tablets x28
d7e7.	TRAZODONE HYDROCHLORIDE 150mg m/r tablets
d7ew.	TRAZODONE HYDROCHLORIDE 100mg capsules
d7ex.	TRAZODONE HYDROCHLORIDE 50mg capsules
d7ez.	TRAZODONE HYDROCHLORIDE 50mg/5mL liquid
d7f..	TRIMIPRAMINE
d7f1.	*SURMONTIL 50mg capsules x28CP
d7f2.	*SURMONTIL 10mg tablets
d7f3.	*SURMONTIL 25mg tablets
d7fx.	TRIMIPRAMINE 50mg capsules
d7fy.	TRIMIPRAMINE 10mg tablets
d7fz.	TRIMIPRAMINE 25mg tablets
d7g..	VILOXAZINE HYDROCHLORIDE
d7g1.	*VIVALAN 50mg tablets
d7gz.	*VILOXAZINE 50mg tablets
d7h..	AMOXAPINE

For peer review only - http://bmjopen.bmj.com/site/about/guidelines.xhtml

CODE	DESCRIPTION
d7h1.	*AMOXAPINE 25mg tablets
d7h2.	*AMOXAPINE 50mg tablets
d7h3.	*AMOXAPINE 100mg tablets
d7h4.	*AMOXAPINE 150mg tablets
d7h5.	*ASENDIS 25mg tablets
d7h6.	*ASENDIS 50mg tablets
d7h7.	*ASENDIS 100mg tablets
d7h8.	*ASENDIS 150mg tablets
d9...	COMPOUND ANTIDEPRESSANT DRUGS
d91..	COMPOUND ANTIDEPRESSANTS A-Z
d911.	*LIMBITROL 5 capsules
d912.	*LIMBITROL 10 capsules
d913.	*MOTIPRESS tablets x28CP
d914.	*MOTIVAL tablets
d915.	*PARSTELIN tablets
d916.	TRIPTAFEN tablets
d917.	*TRIPTAFEN-M tablets
da...	OTHER ANTIDEPRESSANT DRUGS
da1..	FLUPENTIXOL [ANTIDEPRESSANT]
da11.	FLUANXOL 500micrograms tablets
da12.	FLUANXOL 1mg tablets
da1y.	FLUPENTIXOL 500micrograms tablets
da1z.	FLUPENTIXOL 1mg tablets
da2..	TRYPTOPHAN
da21.	*OPTIMAX 500mg tablets
da22.	*OPTIMAX 1g/6g powder
da23.	*OPTIMAX WV 500mg tablets
da24.	*PACITRON 500mg tablets
da2y.	*TRYPTOPHAN 500mg tablets
da2z.	*TRYPTOPHAN 1g/6g powder
da3..	FLUVOXAMINE MALEATE
da31.	FAVERIN 50mg tablets
da32.	FLUVOXAMINE MALEATE 50mg tablets
da33.	FAVERIN 100mg tablets
da34.	FLUVOXAMINE MALEATE 100mg tablets
da4..	FLUOXETINE HYDROCHLORIDE
da41.	FLUOXETINE 20mg capsules
da42.	*PROZAC 20mg capsules x30
da43.	FLUOXETINE 20mg/5mL oral liquid
da44.	PROZAC 20mg/5mL oral liquid
da45.	PROZAC 20mg capsules
da46.	FLUOXETINE 60mg capsules
da47.	*PROZAC 60mg capsules
da48.	*FELICIUM 20mg capsules
da49.	OXACTIN 20mg capsules

For peer review only - http://bmjopen.bmj.com/site/about/guidelines.xhtml

CODE	DESCRIPTION
da4A.	RANFLUTIN 20mg capsules
da4B.	PROZIT 20mg/5mL oral solution
da4C.	PROZEP 20mg/5mL oral solution
da5..	SERTRALINE HYDROCHLORIDE
da51.	SERTRALINE 50mg tablets
da52.	SERTRALINE 100mg tablets
da53.	LUSTRAL 50mg tablets
da54.	LUSTRAL 100mg tablets
da6..	PAROXETINE HYDROCHLORIDE
da61.	PAROXETINE 20mg tablets
da62.	SEROXAT 20mg tablets x30
da63.	PAROXETINE 30mg tablets
da64.	SEROXAT 30mg tablets x30
da65.	PAROXETINE 10mg/5mL sugar free liquid
da66.	SEROXAT 10mg/5mL sugar free liquid
da67.	PAROXETINE 10mg tablets
da68.	SEROXAT 10mg tablets
da7..	VENLAFAXINE
da71.	VENLAFAXINE 37.5mg tablets
da72.	VENLAFAXINE 75mg tablets
da73.	*EFEXOR 37.5mg tablets
da74.	*EFEXOR 75mg tablets
da75.	*VENLAFAXINE 50mg tablets
da76.	*EFEXOR 50mg tablets
da77.	VENLAFAXINE 75mg m/r capsules
da78.	EFEXOR XL 75mg m/r capsules
da79.	VENLAFAXINE 150mg m/r capsules
da7a.	VENAXX XL 150mg m/r capsules
da7A.	EFEXOR XL 150mg m/r capsules
da7b.	VAXALIN XL 75mg m/r capsules
da7B.	RODOMEL XL 75mg m/r capsules
da7c.	VAXALIN XL 150mg m/r capsules
da7C.	RODOMEL XL 150mg m/r capsules
da7d.	ALVENTA XL 75mg m/r capsules
da7D.	WINFEX XL 75mg m/r capsules
da7e.	ALVENTA XL 150mg m/r capsules
da7E.	WINFEX XL 150mg m/r capsules
da7f.	RANFAXINE XL 150mg m/r capsules
da7F.	TRIXAT XL 75mg m/r capsules
da7g.	RANFAXINE XL 75mg m/r capsules
da7G.	TRIXAT XL 150mg m/r capsules
da7h.	BONILUX XL 75mg m/r capsules
da7H.	VIEPAX XL 75mg m/r tablets
da7i.	BONILUX XL 150mg m/r capsules
da7I.	VENLAFAXINE 75mg m/r tablets

For peer review only - http://bmjopen.bmj.com/site/about/guidelines.xhtml

CODE	DESCRIPTION
da7j.	TONPULAR XL 75mg m/r capsules
da7J.	VIEPAX XL 150mg m/r tablets
da7k.	TONPULAR XL 150mg m/r capsules
da7K.	VENLAFAXINE 150mg m/r tablets
da7l.	FORAVEN XL 75mg m/r capsules
da7L.	*TARDCAPS XL 75mg m/r capsules
da7m.	FORAVEN XL 150mg m/r capsules
da7M.	*TARDCAPS XL 150mg m/r capsule
da7n.	DEPEFEX XL 75mg m/r capsules
da7N.	VIEPAX 37.5mg tablets
da7o.	DEPEFEX XL 150mg m/r capsules
da7O.	VIEPAX 75mg tablets
da7p.	VENLALIC XL 37.5mg m/r tablets
da7P.	VENSIR XL 75mg m/r capsules
da7q.	VENLAFAXINE 37.5mg m/r tablets
da7Q.	VENSIR XL 150mg m/r capsules
da7R.	TIFAXIN XL 75mg m/r capsules
da7S.	TIFAXIN XL 150mg m/r capsules
da7T.	VEXARIN XL 75mg m/r capsules
da7U.	VEXARIN XL 150mg m/r capsules
da7V.	VENLALIC XL 75mg m/r tablets
da7W.	VENLALIC XL 150mg m/r tablets
da7X.	VENLALIC XL 225mg m/r tablets
da7Y.	VENLAFAXINE 225mg m/r tablets
da7Z.	VENAXX XL 75mg m/r capsules
da8..	NEFAZODONE
da81.	NEFAZODONE HYDROCHLORIDE 100mg tablets
da82.	NEFAZODONE HYDROCHLORIDE 200mg tablets
da83.	*DUTONIN 100mg tablets
da84.	*DUTONIN 200mg tablets
da85.	NEFAZODONE HCL 50mg+100mg+200mg initiation tablets pack
da86.	DUTONIN 50mg+100mg+200mg treatment initiation tablets pack
da9..	CITALOPRAM
da91.	CITALOPRAM 20mg tablets
da92.	CIPRAMIL 20mg tablets
da93.	CITALOPRAM 10mg tablets
da94.	*CIPRAMIL 10mg tablets
da95.	CITALOPRAM 40mg tablets
da96.	*CIPRAMIL 40mg tablets
da97.	CIPRAMIL 40mg/mL oral drops 15mL
da98.	*PAXORAN 10mg tablets
da99.	*PAXORAN 20mg tablets
da9A.	*PAXORAN 40mg tablets
da9z.	CITALOPRAM 40mg/mL oral drops
daA..	REBOXETINE

For peer review only

CODE	DESCRIPTION
daA1.	REBOXETINE 4mg tablets
daA2.	EDRONAX 4mg tablets
daB..	MIRTAZAPINE
daB1.	MIRTAZAPINE 30mg tablets
daB2.	*ZISPIN 30mg tablets
daB3.	MIRTAZAPINE 30mg oro-dispersible tablets
daB4.	ZISPIN SOLTAB 30mg oro-dispersible tablets
daB5.	MIRTAZAPINE 15mg oro-dispersible tablets
daB6.	ZISPIN SOLTAB 15mg oro-dispersible tablets
daB7.	MIRTAZAPINE 45mg oro-dispersible tablets
daB8.	ZISPIN SOLTAB 45mg oro-dispersible tablets
daBy.	MIRTAZAPINE 45mg tablets
daBz.	MIRTAZAPINE 15mg tablets
daC..	ESCITALOPRAM
daC1.	ESCITALOPRAM 10mg tablets
daC2.	CIPRALEX 10mg tablets
daC3.	ESCITALOPRAM 20mg tablets
daC4.	CIPRALEX 20mg tablets
daC5.	ESCITALOPRAM 5mg tablets
daC6.	CIPRALEX 5mg tablets
daC7.	ESCITALOPRAM 10mg/mL oral drops
daC8.	*CIPRALEX 10mg/mL oral drops
daC9.	CIPRALEX 20mg/mL oral drops
daCA.	ESCITALOPRAM 20mg/mL oral drops
daD..	AGOMELATINE
daD1.	VALDOXAN 25mg tablets
daD2.	AGOMELATINE 25mg tablets
E1...	Non-organic psychoses
E10..	Schizophrenic disorders
E100.	Simple schizophrenia
E1000	Unspecified schizophrenia
E1001	Subchronic schizophrenia
E1002	Chronic schizophrenic
E1003	Acute exacerbation of subchronic schizophrenia
E1004	Acute exacerbation of chronic schizophrenia
E1005	Schizophrenia in remission
E100z	Simple schizophrenia NOS
E101.	Hebephrenic schizophrenia
E1010	Unspecified hebephrenic schizophrenia
E1011	Subchronic hebephrenic schizophrenia
E1012	Chronic hebephrenic schizophrenia
E1013	Acute exacerbation of subchronic hebephrenic schizophrenia
E1014	Acute exacerbation of chronic hebephrenic schizophrenia
E1015	Hebephrenic schizophrenia in remission
E101z	Hebephrenic schizophrenia NOS

For peer review only

For peer review only - http://bmjopen.bmj.com/site/about/guidelines.xhtml

CODE	DESCRIPTION
E102.	Catatonic schizophrenia
E1020	Unspecified catatonic schizophrenia
E1021	Subchronic catatonic schizophrenia
E1022	Chronic catatonic schizophrenia
E1023	Acute exacerbation of subchronic catatonic schizophrenia
E1024	Acute exacerbation of chronic catatonic schizophrenia
E1025	Catatonic schizophrenia in remission
E102z	Catatonic schizophrenia NOS
E103.	Paranoid schizophrenia
E1030	Unspecified paranoid schizophrenia
E1031	Subchronic paranoid schizophrenia
E1032	Chronic paranoid schizophrenia
E1033	Acute exacerbation of subchronic paranoid schizophrenia
E1034	Acute exacerbation of chronic paranoid schizophrenia
E1035	Paranoid schizophrenia in remission
E103z	Paranoid schizophrenia NOS
E104.	Acute schizophrenic episode
E105.	Latent schizophrenia
E1050	Unspecified latent schizophrenia
E1051	Subchronic latent schizophrenia
E1052	Chronic latent schizophrenia
E1053	Acute exacerbation of subchronic latent schizophrenia
E1054	Acute exacerbation of chronic latent schizophrenia
E1055	Latent schizophrenia in remission
E105z	Latent schizophrenia NOS
E106.	Residual schizophrenia
E107.	Schizo-affective schizophrenia
E1070	Unspecified schizo-affective schizophrenia
E1071	Subchronic schizo-affective schizophrenia
E1072	Chronic schizo-affective schizophrenia
E1073	Acute exacerbation subchronic schizo-affective schizophrenia
E1074	Acute exacerbation of chronic schizo-affective schizophrenia
E1075	Schizo-affective schizophrenia in remission
E107z	Schizo-affective schizophrenia NOS
E10y.	Other schizophrenia
E10y0	Atypical schizophrenia
E10y1	Coenesthopathic schizophrenia
E10yz	Other schizophrenia NOS
E10z.	Schizophrenia NOS
E11..	Affective psychoses
E110.	Manic disorder, single episode
E1100	Single manic episode, unspecified
E1101	Single manic episode, mild
E1102	Single manic episode, moderate
E1103	Single manic episode, severe without mention of psychosis

For peer review only - http://bmjopen.bmj.com/site/about/guidelines.xhtml

For peer review only

CODE	DESCRIPTION
E1104	Single manic episode, severe, with psychosis
E1105	Single manic episode in partial or unspecified remission
E1106	Single manic episode in full remission
E110z	Manic disorder, single episode NOS
E111.	Recurrent manic episodes
E1110	Recurrent manic episodes, unspecified
E1111	Recurrent manic episodes, mild
E1112	Recurrent manic episodes, moderate
E1113	Recurrent manic episodes, severe without mention psychosis
E1114	Recurrent manic episodes, severe, with psychosis
E1115	Recurrent manic episodes, partial or unspecified remission
E1116	Recurrent manic episodes, in full remission
E111z	Recurrent manic episode NOS
E112.	Single major depressive episode
E1120	Single major depressive episode, unspecified
E1121	Single major depressive episode, mild
E1122	Single major depressive episode, moderate
E1123	Single major depressive episode, severe, without psychosis
E1124	Single major depressive episode, severe, with psychosis
E1125	Single major depressive episode, partial or unspec remission
E1126	Single major depressive episode, in full remission
E112z	Single major depressive episode NOS
E113.	Recurrent major depressive episode
E1130	Recurrent major depressive episodes, unspecified
E1131	Recurrent major depressive episodes, mild
E1132	Recurrent major depressive episodes, moderate
E1133	Recurrent major depressive episodes, severe, no psychosis
E1134	Recurrent major depressive episodes, severe, with psychosis
E1135	Recurrent major depressive episodes,partial/unspec remission
E1136	Recurrent major depressive episodes, in full remission
E1137	Recurrent depression
E113z	Recurrent major depressive episode NOS
E114.	Bipolar affective disorder, currently manic
E1140	Bipolar affective disorder, currently manic, unspecified
E1141	Bipolar affective disorder, currently manic, mild
E1142	Bipolar affective disorder, currently manic, moderate
E1143	Bipolar affect disord, currently manic, severe, no psychosis
E1144	Bipolar affect disord, currently manic,severe with psychosis
E1145	Bipolar affect disord,currently manic, part/unspec remission
E1146	Bipolar affective disorder, currently manic, full remission
E114z	Bipolar affective disorder, currently manic, NOS
E115.	Bipolar affective disorder, currently depressed
E1150	Bipolar affective disorder, currently depressed, unspecified
E1151	Bipolar affective disorder, currently depressed, mild
E1152	Bipolar affective disorder, currently depressed, moderate

For peer review only - http://bmjopen.bmj.com/site/about/guidelines.xhtml

CODE	DESCRIPTION
E1153	Bipolar affect disord, now depressed, severe, no psychosis
E1154	Bipolar affect disord, now depressed, severe with psychosis
E1155	Bipolar affect disord, now depressed, part/unspec remission
E1156	Bipolar affective disorder, now depressed, in full remission
E115z	Bipolar affective disorder, currently depressed, NOS
E116.	Mixed bipolar affective disorder
E1160	Mixed bipolar affective disorder, unspecified
E1161	Mixed bipolar affective disorder, mild
E1162	Mixed bipolar affective disorder, moderate
E1163	Mixed bipolar affective disorder, severe, without psychosis
E1164	Mixed bipolar affective disorder, severe, with psychosis
E1165	Mixed bipolar affective disorder, partial/unspec remission
E1166	Mixed bipolar affective disorder, in full remission
E116z	Mixed bipolar affective disorder, NOS
E117.	Unspecified bipolar affective disorder
E1170	Unspecified bipolar affective disorder, unspecified
E1171	Unspecified bipolar affective disorder, mild
E1172	Unspecified bipolar affective disorder, moderate
E1173	Unspecified bipolar affective disorder, severe, no psychosis
E1174	Unspecified bipolar affective disorder,severe with psychosis
E1175	Unspecified bipolar affect disord, partial/unspec remission
E1176	Unspecified bipolar affective disorder, in full remission
E117z	Unspecified bipolar affective disorder, NOS
E118.	Seasonal affective disorder
E11y.	Other and unspecified manic-depressive psychoses
E11y0	Unspecified manic-depressive psychoses
E11y1	Atypical manic disorder
E11y2	Atypical depressive disorder
E11y3	Other mixed manic-depressive psychoses
E11yz	Other and unspecified manic-depressive psychoses NOS
E11z.	Other and unspecified affective psychoses
E11z0	Unspecified affective psychoses NOS
E11zz	Other affective psychosis NOS
E12..	Paranoid states
E120.	Simple paranoid state
E121.	Chronic paranoid psychosis
E122.	Paraphrenia
E123.	Shared paranoid disorder
E12y.	Other paranoid states
E12y0	Paranoia querulans
E12yz	Other paranoid states NOS
E12z.	Paranoid psychosis NOS
E13..	Other nonorganic psychoses
E130.	Reactive depressive psychosis
E131.	Acute hysterical psychosis

For peer review only

For peer review only - http://bmjopen.bmj.com/site/about/guidelines.xhtml

CODE	DESCRIPTION
E132.	Reactive confusion
E133.	Acute paranoid reaction
E134.	Psychogenic paranoid psychosis
E135.	Agitated depression
E13y.	Other reactive psychoses
E13y0	Psychogenic stupor
E13y1	Brief reactive psychosis
E13yz	Other reactive psychoses NOS
E13z.	Nonorganic psychosis NOS
E1y..	Other specified non-organic psychoses
E1z..	Non-organic psychosis NOS
E200.	Anxiety states
E2000	Anxiety state unspecified
E2001	Panic disorder
E2002	Generalised anxiety disorder
E2003	Anxiety with depression
E2004	Chronic anxiety
E2005	Recurrent anxiety
E200z	Anxiety state NOS
E204.	Neurotic depression reactive type
E212.	Schizoid personality disorder
E2120	Unspecified schizoid personality disorder
E2121	Introverted personality
E2122	Schizotypal personality
E212z	Schizoid personality disorder NOS
E258.	Nondependent antidepressant type drug abuse
E2580	Nondependent antidepressant type drug abuse, unspecified
E2581	Nondependent antidepressant type drug abuse, continuous
E2582	Nondependent antidepressant type drug abuse, episodic
E2583	Nondependent antidepressant type drug abuse in remission
E258z	Nondependent antidepressant type drug abuse NOS
E291.	Prolonged depressive reaction
E2B..	Depressive disorder NEC
E2B0.	Postviral depression
E2B1.	Chronic depression
Eu...	[X]Mental and behavioural disorders
Eu2..	[X]Schizophrenia, schizotypal and delusional disorders
Eu20.	[X]Schizophrenia
Eu200	[X]Paranoid schizophrenia
Eu201	[X]Hebephrenic schizophrenia
Eu202	[X]Catatonic schizophrenia
Eu203	[X]Undifferentiated schizophrenia
Eu204	[X]Post-schizophrenic depression
Eu205	[X]Residual schizophrenia
Eu206	[X]Simple schizophrenia

For peer review only

CODE	DESCRIPTION
Eu20y	[X]Other schizophrenia
Eu20z	[X]Schizophrenia, unspecified
Eu21.	[X]Schizotypal disorder
Eu22.	[X]Persistent delusional disorders
Eu220	[X]Delusional disorder
Eu221	[X]Delusional misidentification syndrome
Eu222	[X]Cotard syndrome
Eu223	[X]Paranoid state in remission
Eu22y	[X]Other persistent delusional disorders
Eu22z	[X]Persistent delusional disorder, unspecified
Eu23.	[X]Acute and transient psychotic disorders
Eu230	[X]Acute polymorphic psychot disord without symp of schizoph
Eu231	[X]Acute polymorphic psychot disord with symp of schizophren
Eu232	[X]Acute schizophrenia-like psychotic disorder
Eu233	[X]Other acute predominantly delusional psychotic disorders
Eu23y	[X]Other acute and transient psychotic disorders
Eu23z	[X]Acute and transient psychotic disorder, unspecified
Eu24.	[X]Induced delusional disorder
Eu25.	[X]Schizoaffective disorders
Eu250	[X]Schizoaffective disorder, manic type
Eu251	[X]Schizoaffective disorder, depressive type
Eu252	[X]Schizoaffective disorder, mixed type
Eu25y	[X]Other schizoaffective disorders
Eu25z	[X]Schizoaffective disorder, unspecified
Eu26.	[X]Nonorganic psychosis in remission
Eu2y.	[X]Other nonorganic psychotic disorders
Eu2z.	[X]Unspecified nonorganic psychosis
Eu3..	[X]Mood - affective disorders
Eu30.	[X]Manic episode
Eu300	[X]Hypomania
Eu301	[X]Mania without psychotic symptoms
Eu302	[X]Mania with psychotic symptoms
Eu30y	[X]Other manic episodes
Eu30z	[X]Manic episode, unspecified
Eu31.	[X]Bipolar affective disorder
Eu310	[X]Bipolar affective disorder, current episode hypomanic
Eu311	[X]Bipolar affect disorder cur epi manic wout psychotic symp
Eu312	[X]Bipolar affect disorder cur epi manic with psychotic symp
Eu313	[X]Bipolar affect disorder cur epi mild or moderate depressn
Eu314	[X]Bipol aff disord, curr epis sev depress, no psychot symp
Eu315	[X]Bipolar affect dis cur epi severe depres with psyc symp
Eu316	[X]Bipolar affective disorder, current episode mixed
Eu317	[X]Bipolar affective disorder, currently in remission
Eu318	[X]Bipolar affective disorder type I
Eu319	[X]Bipolar affective disorder type II

For peer review only - http://bmjopen.bmj.com/site/about/guidelines.xhtml

CODE	DESCRIPTION
Eu31y	[X]Other bipolar affective disorders
Eu31z	[X]Bipolar affective disorder, unspecified
Eu32.	[X]Depressive episode
Eu320	[X]Mild depressive episode
Eu321	[X]Moderate depressive episode
Eu322	[X]Severe depressive episode without psychotic symptoms
Eu323	[X]Severe depressive episode with psychotic symptoms
Eu324	[X]Mild depression
Eu328	[X]Major depression, severe with psychotic symptoms
Eu329	[X]Single major depr ep, severe with psych, psych in remiss
Eu32A	[X]Recurr major depr ep, severe with psych, psych in remiss
Eu32y	[X]Other depressive episodes
Eu32z	[X]Depressive episode, unspecified
Eu33.	[X]Recurrent depressive disorder
Eu330	[X]Recurrent depressive disorder, current episode mild
Eu331	[X]Recurrent depressive disorder, current episode moderate
Eu332	[X]Recurr depress disorder cur epi severe without psyc sympt
Eu333	[X]Recurrent depress disorder cur epi severe with psyc symp
Eu334	[X]Recurrent depressive disorder, currently in remission
Eu33y	[X]Other recurrent depressive disorders
Eu33z	[X]Recurrent depressive disorder, unspecified
Eu34.	[X]Persistent mood affective disorders
Eu341	[X]Dysthymia
Eu34y	[X]Other persistent mood affective disorders
Eu34z	[X]Persistent mood affective disorder, unspecified
Eu3y.	[X]Other mood affective disorders
Eu3y0	[X]Other single mood affective disorders
Eu3y1	[X]Other recurrent mood affective disorders
Eu3yy	[X]Other specified mood affective disorders
Eu3z.	[X]Unspecified mood affective disorder
Eu41.	[X]Other anxiety disorders
Eu410	[X]Panic disorder [episodic paroxysmal anxiety]
Eu411	[X]Generalized anxiety disorder
Eu412	[X]Mixed anxiety and depressive disorder
Eu413	[X]Other mixed anxiety disorders
Eu41y	[X]Other specified anxiety disorders
Eu41z	[X]Anxiety disorder, unspecified
Eu601	[X]Schizoid personality disorder
R2y2.	[D]Nervousness
SL90.	Antidepressant poisoning
SL90z	Anti-depressant poisoning NOS
SyuFK	[X]Poisoning by other and unspecified antidepressants
T840.	Accidental poisoning by antidepressants
T840z	Accidental poisoning by antidepressants NOS
TJ90.	Adverse reaction to antidepressants

For peer review only - http://bmjopen.bmj.com/site/about/guidelines.xhtml

CODE	DESCRIPTION
TJ90z	Adverse reaction to antidepressants NOS
U6090	[X]Tricyc/tetracyc antidepres caus advers eff therapeut use
U6091	[X]Monoamine oxid inhib antidep caus advers eff therap use
U6092	[X]Other unspec antidepres caus advers effect in therap use
ZV110	[V]Personal history of schizophrenia
ZV111	[V]Personal history of affective disorder

Fit notes (Read)

CODE	DESCRIPTION
13JJ1	Benefit agncy reprt unfit for work but fit note no lngr need
9D1..	MED3 - doctor's statement
9D11.	MED3 issued to patient
9D12.	MED3 duplicate issued
9D14.	MED3 issued - back to work
9D15.	eMED3 (2010) new statement issued, not fit for work
9D19.	MED3 (2010) issued by hand, not fit for work
9D1C.	MED3 (2010) issued to patient
9D1Z.	MED3 - NOS
9D5..	Private sickness certificate
9DC..	SC1 - self certificate admin.
9DC1.	SC1 self-certificate advised
9DC2.	SC1 self certificate given
9DCZ.	SC1 self-certification NOS
9DL..	SC2 - self-certificate administration
9DL0.	SC2 self-certificate advised
9DL1.	SC2 self certificate given
9K8..	Sickness certificate
ZV680	[V]Issue of medical certificate

Hospital admissions for Non-COVID-19 respiratory infections (ICD-10)

CODE	DESCRIPTION
A37	Whooping cough
A370	Whooping cough due to Bordetella pertussis
A371	Whooping cough due to Bordetella parapertussis
A378	Whooping cough due to other Bordetella species
A379	Whooping cough, unspecified
B012	Varicella pneumonia
B052	Measles complicated by pneumonia
B250	Cytomegaloviral pneumonitis
J10	Influenza due to identified seasonal influenza virus
J100	Influenza with pneumonia, seasonal influenza virus identified
J101	Influenza with other respiratory manifestations, seasonal influenza virus identified

For peer review only - http://bmjopen.bmj.com/site/about/guidelines.xhtml

CODE	DESCRIPTION
J108	Influenza with other manifestations, seasonal influenza virus identified
J11	Influenza, virus not identified
J110	Influenza with pneumonia, virus not identified
J111	Influenza with other respiratory manifestations, virus not identified
J118	Influenza with other manifestations, virus not identified
J12	Viral pneumonia, not elsewhere classified
J120	Adenoviral pneumonia
J121	Respiratory syncytial virus pneumonia
J122	Parainfluenza virus pneumonia
J123	Human metapneumovirus pneumonia
J128	Other viral pneumonia
J129	Viral pneumonia, unspecified
J15	Bacterial pneumonia, not elsewhere classified
J150	Pneumonia due to Klebsiella pneumoniae
J151	Pneumonia due to Pseudomonas
J152	Pneumonia due to staphylococcus
J153	Pneumonia due to streptococcus, group B
J154	Pneumonia due to other streptococci
J155	Pneumonia due to Escherichia coli
J156	Pneumonia due to other Gram-negative bacteria
J157	Pneumonia due to Mycoplasma pneumoniae
J158	Other bacterial pneumonia
J159	Bacterial pneumonia, unspecified
J16	Pneumonia due to other infectious organisms, not elsewhere classified
J168	Pneumonia due to other specified infectious organisms
J17	Pneumonia in diseases classified elsewhere
J170	Pneumonia in bacterial diseases classified elsewhere
J171	Pneumonia in viral diseases classified elsewhere
J172	Pneumonia in mycoses
J173	Pneumonia in parasitic diseases
J178	Pneumonia in other diseases classified elsewhere
J18	Pneumonia, organism unspecified
J180	Bronchopneumonia, unspecified
J181	Lobar pneumonia, unspecified
J182	Hypostatic pneumonia, unspecified
J188	Other pneumonia, organism unspecified
J189	Pneumonia, unspecified
J20	Acute bronchitis
J200	Acute bronchitis due to Mycoplasma pneumoniae
J201	Acute bronchitis due to Haemophilus influenzae
J202	Acute bronchitis due to streptococcus
J203	Acute bronchitis due to coxsackievirus
J204	Acute bronchitis due to parainfluenza virus
J205	Acute bronchitis due to respiratory syncytial virus
J206	Acute bronchitis due to rhinovirus

For peer review only

CODE	DESCRIPTION
J207	Acute bronchitis due to echovirus
J208	Acute bronchitis due to other specified organisms
J209	Acute bronchitis, unspecified
J21	Acute bronchiolitis
J210	Acute bronchiolitis due to respiratory syncytial virus
J211	Acute bronchiolitis due to human metapneumovirus
J218	Acute bronchiolitis due to other specified organisms
J219	Acute bronchiolitis, unspecified
J41	Simple and mucopurulent chronic bronchitis
J410	Simple chronic bronchitis
J411	Mucopurulent chronic bronchitis
J418	Mixed simple and mucopurulent chronic bronchitis
J43	Emphysema
J430	MacLeod syndrome
J431	Panlobular emphysema
J432	Centrilobular emphysema
J438	Other emphysema
J439	Emphysema, unspecified
J440	Chronic obstructive pulmonary disease with acute lower respiratory infection
J441	Chronic obstructive pulmonary disease with acute exacerbation, unspecified
J448	Other specified chronic obstructive pulmonary disease
J449	Chronic obstructive pulmonary disease, unspecified
J45	Asthma
J450	Predominantly allergic asthma
J451	Nonallergic asthma
J458	Mixed asthma
J459	Asthma, unspecified
J851	Abscess of lung with pneumonia
J86	Pyothorax
J860	Pyothorax with fistula
J869	Pyothorax without fistula
J982	Interstitial emphysema
J983	Compensatory emphysema
U04	Severe acute respiratory syndrome [SARS]

For peer review only

Supplementary material 3: The RECORD statement – checklist of items, extended from the STROBE statement, that should be reported in observational studies using routinely collected health data.

	Item No.	STROBE items	Section/Location in manuscript where items are reported	RECORD items	Section/Location in manuscript where items are reported
**Title and abstract**					
	1	(a) Indicate the study's design with a commonly used term in the title or the abstract<br><br>(b) Provide in the abstract an informative and balanced summary of what was done and what was found	Title<br><br>Title	RECORD 1.1: The type of data used should be specified in the title or abstract. When possible, the name of the databases used should be included.<br><br>RECORD 1.2: If applicable, the geographic region and timeframe within which the study took place should be reported in the title or abstract.<br><br>RECORD 1.3: If linkage between databases was conducted for the study, this should be clearly stated in the title or abstract.	Title<br><br>Title<br><br>NA
**Introduction**					
Background rationale	2	Explain the scientific background and rationale for the investigation being reported	Introduction		
Objectives	3	State specific objectives, including any prespecified hypotheses	Introduction		
**Methods**					
Study Design	4	Present key elements of study design early in the paper	Study design and data sources		
Setting	5	Describe the setting, locations, and relevant dates, including periods of recruitment, exposure, follow-up, and data collection	Materials and methods		
Participants	6	*(a) Cohort study* - Give the eligibility criteria, and the sources and methods of selection of participants. Describe methods of follow-up<br>*Case-control study* - Give the eligibility criteria, and the sources and methods of case	Population	RECORD 6.1: The methods of study population selection (such as codes or algorithms used to identify subjects) should be listed in detail. If this is not possible, an explanation should be provided.	NA

For peer review only

For peer review only - http://bmjopen.bmj.com/site/about/guidelines.xhtml

	Item No.	STROBE items	Section/Location in manuscript where items are reported	RECORD items	Section/Location in manuscript where items are reported
		ascertainment and control selection. Give the rationale for the choice of cases and controls<br>*Cross-sectional study* - Give the eligibility criteria, and the sources and methods of selection of participants<br><br>*(b) Cohort study* - For matched studies, give matching criteria and number of exposed and unexposed<br>*Case-control study* - For matched studies, give matching criteria and the number of controls per case		RECORD 6.2: Any validation studies of the codes or algorithms used to select the population should be referenced. If validation was conducted for this study and not published elsewhere, detailed methods and results should be provided.<br><br>RECORD 6.3: If the study involved linkage of databases, consider use of a flow diagram or other graphical display to demonstrate the data linkage process, including the number of individuals with linked data at each stage.	NA<br><br>NA
Variables	7	Clearly define all outcomes, exposures, predictors, potential confounders, and effect modifiers. Give diagnostic criteria, if applicable.	Primary and secondary outcomes	RECORD 7.1: A complete list of codes and algorithms used to classify exposures, outcomes, confounders, and effect modifiers should be provided. If these cannot be reported, an explanation should be provided.	Supplementary material
Data sources/ measurement	8	For each variable of interest, give sources of data and details of methods of assessment (measurement).<br>Describe comparability of assessment methods if there is more than one group	Primary and secondary outcomes and supplementary material 1		
Bias	9	Describe any efforts to address potential sources of bias	Discussion – strengths and weaknesses		
Study size	10	Explain how the study size was arrived at	Protocol paper		
Quantitative variables	11	Explain how quantitative variables were handled in the analyses. If applicable, describe which groupings were chosen, and why	Analysis		
Statistical methods	12	(a) Describe all statistical methods, including those used to control for confounding	Analysis		

For peer review only - http://bmjopen.bmj.com/site/about/guidelines.xhtml

	Item No.	STROBE items	Section/Location in manuscript where items are reported	RECORD items	Section/Location in manuscript where items are reported
		(b) Describe any methods used to examine subgroups and interactions<br>(c) Explain how missing data were addressed<br>(d) *Cohort study* - If applicable, explain how loss to follow-up was addressed<br>*Case-control study* - If applicable, explain how matching of cases and controls was addressed<br>*Cross-sectional study* - If applicable, describe analytical methods taking account of sampling strategy<br>(e) Describe any sensitivity analyses			
Data access and cleaning methods		..		RECORD 12.1: Authors should describe the extent to which the investigators had access to the database population used to create the study population.<br><br>RECORD 12.2: Authors should provide information on the data cleaning methods used in the study.	Analysis<br><br>Analysis
Linkage		..		RECORD 12.3: State whether the study included person-level, institutional-level, or other data linkage across two or more databases. The methods of linkage and methods of linkage quality evaluation should be provided.	Study design and data sources
**Results**					
Participants	13	(a) Report the numbers of individuals at each stage of the study (*e.g.*, numbers potentially eligible, examined for eligibility, confirmed eligible, included in the study, completing follow-up, and analysed)<br>(b) Give reasons for non-participation at each stage.<br>(c) Consider use of a flow diagram	Results: Study population	RECORD 13.1: Describe in detail the selection of the persons included in the study (*i.e.,* study population selection) including filtering based on data quality, data availability and linkage. The selection of included persons can be described in the text and/or by means of the study flow diagram.	Results: Study population

For peer review only - http://bmjopen.bmj.com/site/about/guidelines.xhtml

	Item No.	STROBE items	Section/Location in manuscript where items are reported	RECORD items	Section/Location in manuscript where items are reported
Descriptive data	14	(a) Give characteristics of study participants (*e.g.*, demographic, clinical, social) and information on exposures and potential confounders<br>(b) Indicate the number of participants with missing data for each variable of interest<br>(c) *Cohort study* - summarise follow-up time (*e.g.*, average and total amount)	Results: Study population, table 1		
Outcome data	15	*Cohort study* - Report numbers of outcome events or summary measures over time<br>*Case-control study* - Report numbers in each exposure category, or summary measures of exposure<br>*Cross-sectional study* - Report numbers of outcome events or summary measures	Results, table 2		
Main results	16	(a) Give unadjusted estimates and, if applicable, confounder-adjusted estimates and their precision (e.g., 95% confidence interval). Make clear which confounders were adjusted for and why they were included<br>(b) Report category boundaries when continuous variables were categorized<br>(c) If relevant, consider translating estimates of relative risk into absolute risk for a meaningful time period	Results, table 1 and 2, supplementary material 4-5		
Other analyses	17	Report other analyses done—e.g., analyses of subgroups and interactions, and sensitivity analyses	Results, supplementary material 4-5		
**Discussion**					
Key results	18	Summarise key results with reference to study objectives	Discussion-Principal findings		

For peer review only - http://bmjopen.bmj.com/site/about/guidelines.xhtml

	Item No.	STROBE items	Section/Location in manuscript where items are reported	RECORD items	Section/Location in manuscript where items are reported
Limitations	19	Discuss limitations of the study, taking into account sources of potential bias or imprecision. Discuss both direction and magnitude of any potential bias	Discussion-Strengths and weaknesses	RECORD 19.1: Discuss the implications of using data that were not created or collected to answer the specific research question(s). Include discussion of misclassification bias, unmeasured confounding, missing data, and changing eligibility over time, as they pertain to the study being reported.	Page 16
Interpretation	20	Give a cautious overall interpretation of results considering objectives, limitations, multiplicity of analyses, results from similar studies, and other relevant evidence	Discussion-strengths and weaknesses		
Generalisability	21	Discuss the generalisability (external validity) of the study results	Discussion - findings in context		
**Other Information**					
Funding	22	Give the source of funding and the role of the funders for the present study and, if applicable, for the original study on which the present article is based	Funding		
Accessibility of protocol, raw data, and programming code		..		RECORD 22.1: Authors should provide information on how to access any supplemental information such as the study protocol, raw data, or programming code.	Data sharing statement, protocol, Supplementary material 2

For peer review only - http://bmjopen.bmj.com/site/about/guidelines.xhtml

Supplementary material 4: Hospital admissions for respiratory infections not necessarily recorded as COVID-19 for domiciliary care workers from March 2016 to October 2021.

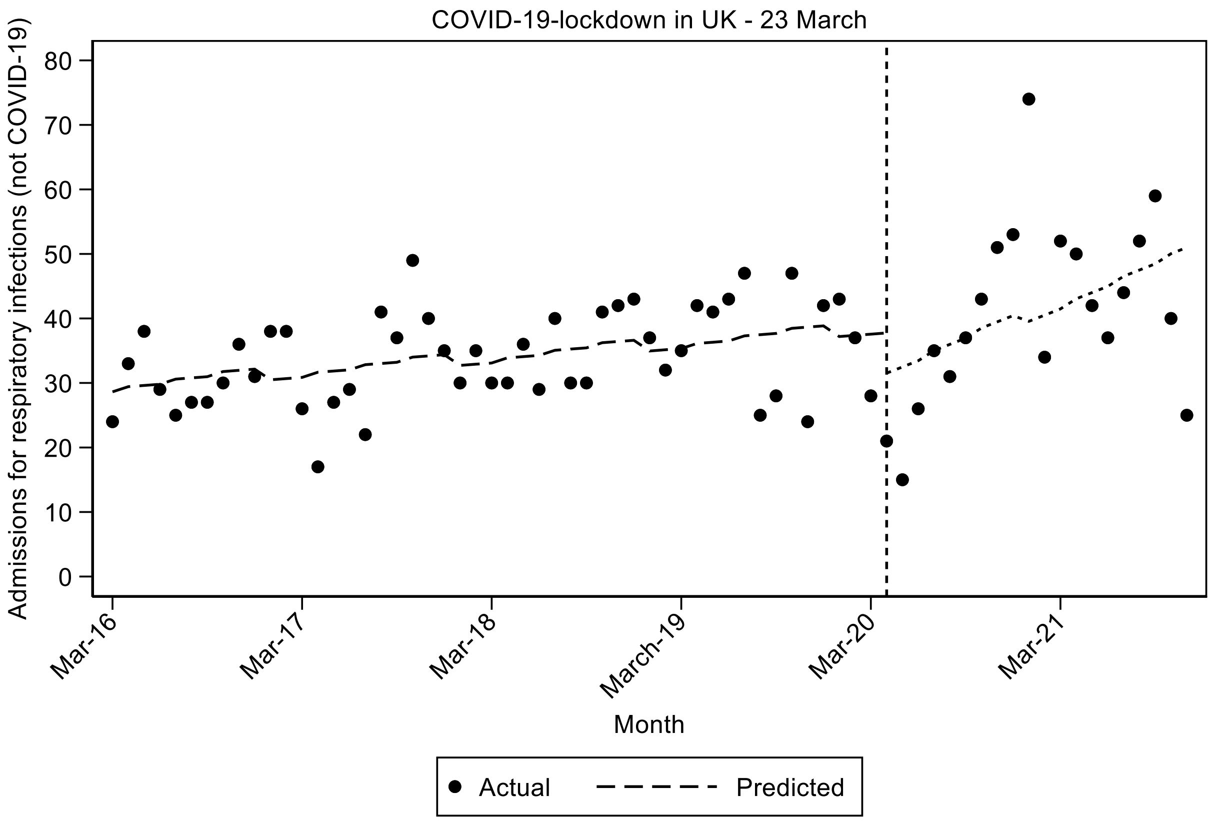

review only

Supplementary material 5: Characteristics of domiciliary care workers receiving at least one fit note in the COVID-19 pandemic between 1st March 2020 and 31st November 2021.

Characteristics	N	%
**Source: SCW[a]**		
Sex		
Female	2,004	15.1
Male	293	11.9
Age (at registration)		
Under 35 years	572	12.9
35 to <45 years	380	13.5
45 to <55 years	625	15.8
55 years and over	720	15.9
Ethnicity		
White	2,220	14.8
Black	11	7.8
Mixed	*	*
Asian	14	13.0

For peer review only - http://bmjopen.bmj.com/site/about/guidelines.xhtml

Characteristics	N	%
Other	*	*
Mixed/Other	18	9.3
Not recorded	34	12.6
** numbers suppressed as <10. Mixed and Other categories are combined.*		
Lives with DCW		
No	2,168	14.7
Yes	129	13.2
Have disability		
No	2,089	14.8
Yes	23	12.2
Missing/Prefer not to answer	185	13.3
Qualifications as recorded at registration		
Competence confirmed	255	12.9
Induction Framework	460	15.4
Qualification Level 2	1,054	15.2
Qualification Level 3	470	14.1
Qualification Level 4+	48	10.9
Not recorded	10	23.3
Employment sector as recorded at registration		
Private	828	12.6
Third Sector	562	13.2
Local Authority - Social Services	678	20.5
Recruitment Agency/ Employment Agency	23	13.4
Health/Other		
Not recorded	206	14.8
Smoking status		
Non-smoker	917	14.5
Ex-smoker	601	12.0
Current smoker	779	17.8
Body Mass Index		
Underweight (<18.5)	37	14.5
Healthy weight (18.5-24.9)	466	14.7
Overweight (25-29.9)	576	15.8
Obese (≥30)	993	18.7
Not recorded	225	6.8
Any comorbidity[d]		
No	1,027	11.5
Yes	1,270	18.7
On COVID-19 shielding list		
No	2,175	14.4
Yes	122	19.2
Resident health board		
Aneurin Bevan	319	11.3
Betsi Cadwaladr	854	23.2
Cardiff and Vale	219	12.2
Cwm Taf Morgannwg	365	13.5

For peer review only

For peer review only - http://bmjopen.bmj.com/site/about/guidelines.xhtml

Characteristics	N	%
Hywel Dda	225	11.5
Powys	83	12.5
Swansea Bay	232	11.1
WIMD 2019 quintile		
1 (most deprived)	636	15.3
2	547	13.8
3	478	14.5
4	390	15.3
5 (most affluent)	246	14.2
Rurality classification		
Rural	718	14.6
Urban	1,579	14.6

a between 1st March 2020 to 31st November 2021; b data source = Social Care Wales (DSCW) data and include data that is self-reported by DCWs at 1st April. If missing, then other sources (ONS 2011 Census Wales (CENW), Wales Longitudinal General Practice (WLGP), Patient Episode Dataset for Wales (PEDW), Emergency Department Dataset (EDDS)) were used to impute (at index date=1st March 2020); c data source = WLGP; d data source=PEDW; e most recent occurring between 1st January 2000 and 1st March 2020; f based on residential address at 1st March 2020; g data source =Welsh Demographic Service Dataset (WDSD).

For peer review only

For peer review only - http://bmjopen.bmj.com/site/about/guidelines.xhtml

For peer review only

For peer review only - http://bmjopen.bmj.com/site/about/guidelines.xhtml

For peer review only

For peer review only - http://bmjopen.bmj.com/site/about/guidelines.xhtml

Supplementary material 6: Demographics of DCWs across the UK

Nation	Date published	Definition	Denom.	Age	Gender	Ethnicity
Wales[1]	April 2021	DCWs	22,131	44 years (mean)	84% F<br>16% M	97% White British
OSCAR sample	April 2020	DCWs	15,725	44 years (mean)	84% F<br>16% M	96% White British
England[2]	October 2021	DCWs	590,000	44 years (mean)	84% F<br>16% M	na
Scotland[3]	December 2020	Housing support/ care at home	74,870	46 years (median)	77% F<br>17 %M<br>6% unknown<br><br>84% F<br>16% M<br>(excluding unknowns)	98% White<br>2% Asian/Black
Northern Ireland[4]	October 2020	DCWs	16,303	20-29 years (mode)	91% F<br>8% M<br>1% Other	na

na = not available

[1] Domiciliary-care-worker-ENG.pdf (socialcare.wales) https://socialcare.wales/cms_assets/file-uploads/Domiciliary-care-worker-ENG.pdf

[2] The state of the adult social care sector and workforce in England (skillsforcare.org.uk) https://www.skillsforcare.org.uk/adult-social-care-workforce-data/Workforce-intelligence/publications/national-information/The-state-of-the-adult-social-care-sector-and-workforce-in-England.aspx

[3] Adults' services workforce tables - 2019 | Scottish Social Services Workforce Data (sssc.uk.com) https://data.sssc.uk.com/data-publications/248-adults-services-workforce-in-scotland-tables-2019

[4] Rapid learning review of domiciliary care in Northern Ireland (Department of Health) https://www.health-ni.gov.uk/rli-dom-care (page 13)

For peer review only - http://bmjopen.bmj.com/site/about/guidelines.xhtml

The original protocol for the study, as a supplementary file

For peer review only

For peer review only - http://bmjopen.bmj.com/site/about/guidelines.xhtml

BMJ Open

Impact of the COVID-19 pandemic on domiciliary care workers in Wales, UK: a data linkage cohort study using the SAIL Databank

Journal:	*BMJ Open*
Manuscript ID	bmjopen-2022-070637.R2
Article Type:	Original research
Date Submitted by the Author:	11-May-2023
Complete List of Authors:	Cannings-John, Rebecca; Cardiff University<br>Schoenbuchner, Simon; Cardiff University, Centre for Trials Research<br>Jones, Hywel; Cardiff University, Division of Population Medicine<br>Lugg-Widger, Fiona; Cardiff University, Centre for Trials Research<br>Akbari, Ashley; Swansea University Medical School, Faculty of Medicine, Health & Life Science<br>Brookes-Howell, Lucy; Cardiff University Centre for Trials Research<br>Hood, Kerenza; Cardiff University, Centre for Trials Research<br>John, Ann; Swansea University, Health Data Research UK | Administrative Data Research Wales; Cardiff University, DECIPHer - Centre for Development, Evaluation, Complexity and Implementation in Public Health Improvement<br>Thomas, Daniel; Public Health Wales, Communicable Disease Surveillance Centre; Cardiff Metropolitan University<br>Prout, Hayley; Cardiff University School of Medicine, Centre for Trials Research<br>Robling, Michael; Cardiff University, Centre for Trials Research
**Primary Subject Heading**:	Occupational and environmental medicine
Secondary Subject Heading:	Epidemiology, Infectious diseases, Mental health, Public health
Keywords:	COVID-19, MENTAL HEALTH, SOCIAL MEDICINE

SCHOLARONE™
Manuscripts

I, the Submitting Author has the right to grant and does grant on behalf of all authors of the Work (as defined in the below author licence), an exclusive licence and/or a non-exclusive licence for contributions from authors who are: i) UK Crown employees; ii) where BMJ has agreed a CC-BY licence shall apply, and/or iii) in accordance with the terms applicable for US Federal Government officers or employees acting as part of their official duties; on a worldwide, perpetual, irrevocable, royalty-free basis to BMJ Publishing Group Ltd ("BMJ") its licensees and where the relevant Journal is co-owned by BMJ to the co-owners of the Journal, to publish the Work in this journal and any other BMJ products and to exploit all rights, as set out in our licence.

The Submitting Author accepts and understands that any supply made under these terms is made by BMJ to the Submitting Author unless you are acting as an employee on behalf of your employer or a postgraduate student of an affiliated institution which is paying any applicable article publishing charge ("APC") for Open Access articles. Where the Submitting Author wishes to make the Work available on an Open Access basis (and intends to pay the relevant APC), the terms of reuse of such Open Access shall be governed by a Creative Commons licence – details of these licences and which Creative Commons licence will apply to this Work are set out in our licence referred to above.

Other than as permitted in any relevant BMJ Author's Self Archiving Policies, I confirm this Work has not been accepted for publication elsewhere, is not being considered for publication elsewhere and does not duplicate material already published. I confirm all authors consent to publication of this Work and authorise the granting of this licence.

For peer review only

Impact of the COVID-19 pandemic on domiciliary care workers in Wales, UK: a data linkage cohort study using the SAIL Databank

Authors:

Rebecca Cannings-John, PhD[1]

Simon Schoenbuchner, PhD[1]

Hywel Jones BSc DMS CStat[2]

Fiona Lugg-Widger, PhD[1]

Ashley Akbari, MSc[3]

Lucy Brookes-Howell, PhD[1]

Kerry Hood, PhD[1]

Ann John, MBBS MD [3,4]

Daniel Rh Thomas, PhD[5,6]

Hayley Prout, MPhil[1]

Michael Robling, PhD[1,4]

Affiliations: 1: Centre for Trials Research, School of Medicine, Cardiff University, Cardiff, United Kingdom; 2: Division of Population Medicine, Cardiff University; 3: Population Data Science, Swansea University Medical School, Faculty of Medicine, Health & Life Science, Swansea University; 4: DECIPHer - Centre for Development, Evaluation, Complexity and Implementation in Public Health Improvement, sbarc|spark, Cardiff University; 5: Public Health Wales Communicable Disease Surveillance Centre; 6: Cardiff Metropolitan University.

Correspondence to:

Rebecca Cannings-John,

Centre for Trials Research,

Cardiff University,

Cardiff, UK

canningsrl@cardiff.ac.uk

For peer review only - http://bmjopen.bmj.com/site/about/guidelines.xhtml

ABSTRACT

Objectives: To quantify population health risks for domiciliary care workers (DCWs) in Wales, UK, working during the COVID-19 pandemic.

Design: A population-level retrospective study linking occupational registration data to anonymised electronic health records maintained by the Secure Anonymised Information Linkage Databank in a privacy-protecting trusted research environment.

Setting: Registered DCWs population in Wales.

Participants: Records for all linked DCWs from 1st March 2020 to 30th November 2021.

Primary and secondary outcome measures: Our primary outcome was confirmed COVID-19 infection; secondary outcomes included contacts for suspected COVID-19, mental health including self-harm, fit notes, respiratory infections not necessarily recorded as COVID-19, deaths involving COVID-19, and all-causes mortality.

Results: Confirmed and suspected COVID-19 infection rates increased over the study period to 24% by 30th November 2021. Confirmed COVID-19 varied by sex (males: 19% vs. females: 24%) and age (>55 years: 19% vs. <35 years: 26%) and were higher for care workers employed by local authority social services departments compared to the private sector (27% and 23%, respectively). 34% of DCWs required support for a mental health condition, with mental health related prescribing increasing in frequency when compared to the pre-pandemic period. Events for self-harm increased from 0.2% to 0.4% over the study period as did the issuing of fit notes. There was no evidence to suggest a miscoding of COVID-19 infection with non-COVID-19 respiratory conditions. COVID-19-related and all-cause mortality were no greater than for the general population of 15- to 64-year-olds in Wales (0.1% and 0.034%, respectively). A comparable DCW workforce in Scotland and England would result in a comparable rate of COVID-19 infection; whilst the younger workforce in Northern Ireland may result in a greater infection rate.

Conclusions: While initial concerns about excess mortality are alleviated, the substantial pre-existing and increased mental health burden for DCWs will require investment to provide long-term support to the sector's workforce.

Keywords: Domiciliary care worker, workforce, COVID-19, mental health, data linkage, social care, Wales

STRENGTHS AND LIMITATIONS OF THIS STUDY

- Linking registration data to electronic health records in Wales provided a unique opportunity to explore health outcomes for domiciliary care workers (DCWs) working during the pandemic.

For peer review only - http://bmjopen.bmj.com/site/about/guidelines.xhtml

- No DCWs dissented for their registration data to be made available for linkage, allowing a comprehensive population level analysis.
- Demographic data available by linked registration and healthcare datasets allowed assessment by key DCW sub-groups.
- Our study provides a broad range of health outcome estimates for DCWs over an extended period of the COVID-19 pandemic.
- The recording of some data could have introduced bias, e.g. coding for COVID-19 biased towards individuals presenting in primary or secondary healthcare.

For peer review only

INTRODUCTION

During 2018-20, more than 950,000 people in the UK received domiciliary care from nearly 823,000 domiciliary care workers (DCWs) from over 10,100 home care providers and the demand is predicted to increase significantly over the next 20 years[1]. DCWs provide support to adults in their own home with household tasks, personal care or any activities that allow the individual to maintain independence. Support can demand close contact, assisting with bodily functions, managing continence, assisting with oral and dental care, and providing other personal care. In the UK, domiciliary care can be funded publicly by local councils, privately funded, or a combination of both.

These close working conditions with clinically vulnerable people, and the varied functions that DCWs perform in a non-institutional setting in a workforce with a large number of employers, may increase exposure to COVID-19. A cross-sectional survey led by Ulster University found that during the early period of the COVID-19 pandemic (between May–July 2020 and November 2020–January 2021), both wellbeing and quality of working life deteriorated over all occupations including Social Care [2]. This decrease was again observed when care workers were surveyed between May and July 2021, with personal and work-related burnout reported to increase [2]. However, the survey did not aim to capture objectively recorded health outcomes specifically in DCWs, for example positive rates of COVID-19 infection and the risk factors which contribute to these. In 2020, the Office for National Statistics (ONS) reported elevated mortality rates due to COVID-19 for 'care workers and home carers' compared to the general population in England and Wales [3].] In June 2020, Public Health England reported COVID-19 infection rates for DCWs in line with general population rates [4]. Methodological differences between the two studies may explain contrasting estimates and neither study offered population coverage for a well-defined cohort of DCWs. Therefore, despite a policy interest in understanding level of risk for a population of workers potentially at increased likelihood of exposure to COVID-19 (and consequently the risk they could pose to clients), there was uncertainty about rates of COVID-19 infection or any other health outcome.

From 1st April 2020, it became mandatory for DCWs in the active Welsh workforce to be registered with Social Care Wales [5]. The population of working DCWs in Wales during the COVID-19 pandemic is therefore known, and can be linked to anonymised data, including health and administrative data held in the SAIL Databank (Swansea University), providing a unique opportunity to explore a range of health outcomes (including COVID-19 infection and mortality).

The OSCAR study (Outcomes for Social Carers: an Analysis using Routine data) aimed to utilise the registration data collected by Social Care Wales, individually linked to secure anonymised electronic health records (EHRs) to quantify population health risks for DCWs in Wales working during the COVID-19 pandemic. Specifically, we aimed to quantify rates of confirmed and suspected COVID-19 infection and, to fully establish the range of adverse health outcomes potentially affecting DCWs

For peer review only - http://bmjopen.bmj.com/site/about/guidelines.xhtml

such as mental health including self-harm, issuing of fit notes, non-COVID-19 hospital admissions for respiratory conditions, and COVID-19 related and all-cause mortality, as a consequence of the pandemic. We compared mental health outcomes (GP contacts and prescriptions, and hospital admissions) within the pandemic period to the pre-pandemic situation to assess the effect of the pandemic. Similarly, we examined admissions over time for respiratory infection in the early pandemic to identify the potential miscoding of COVID-19 infection. Outcome variation was explored by demographics, work-related factors, lifestyle and comorbidities. To develop timely public health policy messages that could be extended to DCWs in other nations of the UK, using up-to-date DCW workforce data from Scotland, Northern Ireland and England we compared these populations to the Welsh DCW workforce and assessed the generalisability of prevalence findings to each nation.

METHODS

Study design and data sources

This study is a population-level mixed methods retrospective routine data linkage study with analysis guided by exploratory qualitative interviews. Qualitative findings are reported separately [6]. Registration data for all DCWs in Wales registered by 1st April 2020 are held by Social Care Wales (Domiciliary Social Care Worker (DSCW)) and available via the Secure Anonymised Information Linkage (SAIL) Databank (Swansea University) a privacy-protecting trusted research environment (TRE), which uses a standardised split-file secure, encrypted anonymisation process [7]. These data were combined with I data sources within the SAIL Databank (supplementary material 1 for data sources used).

Population

The study population was all registered DCWs resident in Wales on 1st March 2020 who did not subsequently opt-out to their data being transferred to the SAIL Databank and linked for research, either when DSCW data were added to the SAIL Databank or via their general practice (GP). Any DCWs not linked into the SAIL Databank with sufficient confidence were excluded. Additionally, individuals included in the dataset but registered as Domiciliary Care Managers, Adult Care Home Managers or Residential Child Care Workers were excluded.

Primary and secondary outcomes

The primary outcome was confirmed COVID-19 infection, defined as the earliest of the following events: a positive polymerase chain reaction (PCR) test (Pathology Test Results dataset, PATD) [8], hospital admission (Patient Episode Database for Wales, PEDW) [9], death registration from COVID-19 (main or secondary diagnostic ICD-10 code=U07.1) (Annual District Death Extract, ADDE from the Office for National Statistics (ONS) mortality register) [10], or a COVID-19 diagnosis (Welsh Longitudinal GP, WLGP) [11].

For peer review only - http://bmjopen.bmj.com/site/about/guidelines.xhtml

Secondary outcomes were:

- Suspected COVID-19 infection (Data sources: PATD, WLGP, PEDW, and ADDE);
- Contacts for mental health and diagnoses, psychotropic medication and admissions (including self-harm) (Data sources: WLGP, and PEDW);
- Fit notes as a general marker of medically confirmed illness (Data source: WLGP);
- Non-COVID-19 hospital admissions for respiratory conditions (Data source: PEDW);
- Deaths involving COVID-19 and all-cause mortality (Data source: ADDE).

In brief, we examined depression and anxiety symptoms and diagnoses [12,13], and severe mental health in GP data and hospital admissions data [14]. Self-harm, used GP data for definite and undetermined intent (WLGP) [15,16]. Fit notes also used GP data to cover medical/sickness/self-certificates issued to patients by GPs and included issues for returning to work. Hospital admissions for respiratory conditions were examined for potential miscoding of COVID-19 infection in the early pandemic (March to May 2020) and included any lower respiratory infection, pneumonia, and influenza-like-illness [17], exacerbations of chronic obstructive pulmonary disease (COPD) [18] and asthma [19].

Study period and follow-up

DCWs entered the cohort on 1st March 2020 and were followed up until 30th November 2021. We examined outcomes by waves of the pandemic; short term outcomes from 1st March 2020 to 31st August 2020 (Wave 1: reflecting a period before the relaxing of lockdown in Wales) and from 1st September 2020 to 28th February 2021 (Wave 2). Long-term outcomes were examined to 30th November 2021. DCWs were followed up until the earliest of occurrence of death, first migration out of Wales, the outcome of interest, or the end of the follow-up.

DCW characteristics

We chose age, sex, ethnicity, health board, deprivation quintile and rurality as relevant characteristics for the DCWs using a number of data sources (supplementary material 1). We also identified DCWs living with disability, any comorbidities [14], shielding status, and lifestyle factors such as body mass index and smoking status. From the DSCW data, we were able to describe how individuals registered as a DCW (induction, registered with a qualification, competence confirmed by their manager) which reflects the different pathways open to care workers when mandatory registration was introduced [20]. Some pathways were transitional and are now no longer available (e.g. registration following induction). We also identified whether a DCW lived with another DCW and their employment sector at the time of registration (Private, Third sector, Local Authority/Social Services, Recruitment/Employment Agency/Other). Several items from the DSCW were not available due to confidentiality considerations, such as length of time in the job, type of employment (e.g.in social

For peer review only - http://bmjopen.bmj.com/site/about/guidelines.xhtml

care, self-employed), and type of qualifications held (Qualifications and Credit Framework (QCF), National Vocational Qualification (NVQ) etc).

A full coding list for outcomes is in supplementary material 2 and also on Open Science Framework (OSF) (https://osf.io/9w6pe/) alongside codes for the comorbidities.

Governance

The study received approval from the School of Medicine Research Ethics Committee, Cardiff University [SMREC 20/114]. Approval was obtained from the SAIL Information Governance Review Panel (IGRP), Swansea University (Project ID: 1126) and data access was granted to named staff. Approval from the ONS Research Accreditation Panel (ONS RAP) was provided (16/12/2020) and approval from Social Care Wales was provided (26/01/2021). This process was generic and not specific to the OSCAR study.

Analysis

The linkage of DCWs IEHR were described and the study population characterised using frequencies and percentages. The presence of key health outcomes were described by the short and long-term periods; prevalence estimates presented alongside 95% confidence intervals (CIs). This study is primarily designed to describe variation in outcomes in the DCW population, by key subgroups, rather than for prediction or causal models. Using multilevel Cox regression to model time to first confirmed COVID-19 infection, we estimated hazard ratios (HRs) with 95% CIs, to quantify the risk of confirmed COVID-19 by comorbidity status adjusting for age, sex and deprivation, and also by their employing sector adjusting for health board, rurality, and qualification type. DCWs were censored at the time of the event, migration, death, or at the study end.

To explore changes over time and the effect of the COVID-19 pandemic period, an interrupted time series approach was used to test the hypothesis that hospital admissions for non-COVID-19 respiratory infections would increase in comparison to the rates seen pre-March 2020 if there was misclassification of infections. Monthly data were divided into pre-pandemic (1st March 2016 to 28th February 2020) and the pandemic period (1st March 2020 to 31st October 2022). The changes in levels (baseline value of the outcome at time zero) and trends (the rate of change) of the pre-pandemic segment and pandemic segment were analysed using the *itsa* command in Stata. A Cumby-Huizinga test was used to determine the autocorrelation and was adjusted using Newey West AC estimators. Using the same approach and as a post-hoc analysis, the number of mental health GP contacts and prescriptions, and hospital admissions were also examined.

Equivalent up-to-date DCW workforce data from Scotland, Northern Ireland and England was obtained and compared to the Welsh DCW cohort allowing us to generalise the estimate confirmed COVID-19 other nations.

For peer review only - http://bmjopen.bmj.com/site/about/guidelines.xhtml

To comply with disclosure control process and approvals, and protect the privacy of anonymised individuals, numbers <5 are suppressed. Percentages associated with small frequencies are rounded to the nearest 1%. Study findings were reported in accordance with applicable reporting guidelines for observational studies using administrative data (STROBE and RECORD) (supplementary material 3) [21,22]. Data cleaning, cohort assembly and statistical analyses were performed using Structured Query Language (SQL) (IBM Db2 11.1) [23] and R (v4.1.0-4.1.3) [24,25], and Stata (v17)[26] within the SAIL Databank privacy-protecting TRE.

Patient and public involvement

Two stakeholder groups provided input to the project, an ongoing Study Advisory Group and an Implementation Reference Group. Both groups included membership drawn from the domiciliary care sector and both contributed to interpretation of emerging results.

RESULTS

Study population

Data from 15,931 DCWs resident and working in Wales on 1st March 2020 were linkIto EHR data (85% of them had GP records in SAIL). Records from 206 DCWs were excluded due to matching errors, leaving 15,725 (98.7%) DCWs included in the final analysis. The majority of DCWs were female (84%), of white ethnic background (95%), 43% had at least one comorbidity of which 22% had been recorded with mental health related illness (Table 1).

Confirmed COVID-19 infection

Confirmed COVID-19 infection was 1.2% at 31st August 2020, rose to 14% by the end of February 2021, and reached 24% by the end of November 2021 (Table 2). There was no evidence to suggest a miscoding of COVID-19 infection in the early pandemic, with no difference in admissions for non-COVID-19 respiratory conditions over time (supplementary material 4). Confirmed COVID-19 infection was more prevalent in females, those who registered following the induction pathway compared to those with competency confirmed by a manager, those employed by the Local Authority Social Services at registration compared to those employed in the private sector (Figure 1), those living with another DCW, those with a BMI greater than 30 compared to those with a healthy BMI (18.5 to 24.9) and those living in an urban area (Table 1). Confirmed COVID-19 infection was less prevalent in older DCW. Variation was also observed across health boards. After adjusting for age, sex and deprivation, COVID-19 infection was still more prevalent in those with a comorbidity (HR=1.08, 95% CI: 1.01 to 1.15) and additionally adjusting for health board, rurality, and

For peer review only - http://bmjopen.bmj.com/site/about/guidelines.xhtml

qualification type, was more prevalent in those employed by the Local Authority Social Services at registration (1.35, 1.23 to 1.47).

Table 1. Characteristics of domiciliary care workers with at least one confirmed COVID-19 infection and contacts for mental health and diagnoses, psychotropic medication and admissions in the COVID-19 pandemic between 1st March 2020 and 31st November 2021

	DCWs N=15,725		Confirmed COVID-19[a] N= 3,698		Mental health[a]	
	n	%	n	%	n	%
**DCW Characteristics**						
Sex[b]						
Female	13,253	84.3	3,226	24.3	4,685	35.4
Male	2,472	15.7	472	19.1	615	24.9
Age (at registration)[b]						
Under 35 years	4,425	28.1	1,164	26.3	1,542	34.9
35 to <45 years	2,822	17.9	795	28.2	977	34.6
45 to <55 years	3,955	25.2	897	22.7	1,398	35.4
55 years and over	4,523	28.8	842	18.6	1,383	30.6
Ethnicity[b]						
White	15,012	95.5	3,537	23.6	5,128	34.2
Black	142	0.9	35	24.7	22	15.5
Mixed	127	0.8	*	*	*	*
Asian	108	0.7	25	23.2	18	16.7
Other	66	0.4	*	*	*	*
Mixed/Other	193	1.2	42	21.8	46	23.8
Not recorded	270	1.7	59	21.9	86	31.9
Lives with DCW?[b] - Yes	975	6.2	254	26.1	312	32.0
No	14,750	93.8	3,444	23.4	4,988	33.8
Has disability?[b] - Yes	188	1.2	30	16.0	91	48.4
No	14141	89.9	3,310	23.4	4,714	33.3
Not recorded /Prefer not to answer	1,396	8.9	358	25.6	495	35.5
Qualifications as recorded at registration[b]						
Competence confirmed	1982	12.6	438	22.1	646	32.6
Induction Framework	2984	19.0	737	24.7	1,094	36.7
Qualification Level 2	6935	44.1	1,672	24.1	2,328	33.6
Qualification Level 3	3340	21.2	752	22.5	1,077	32.3
Qualification Level 4+	441	2.8	88	20.0	140	31.8
Not recorded	43	0.3	11	25.6	15	32.7
Employment sector as recorded at registration[b]						
Private	6,592	41.9	1,480	22.5	2,154	32.7
Third Sector	4,267	27.1	991	23.2	1,454	34.1
Local Authority-Social Services	3,306	21.0	876	26.5	1,159	35.1
Recruitment/Employment Agency	148	0.9	43	25.0	65	37.8
Health/Other	24	0.2				
Not recorded	1388	8.8	308	22.2	468	33.7
Smoking status[c]						

For peer review only

	DCWs N=15,725		Confirmed COVID-19[a] N= 3,698		Mental health[a]	
	n	%	n	%	n	%
Non-smoker	6,342	40.3	1,638	25.8	2,076	32.7
Current smoker	4,374	27.8	875	20.0	1,781	40.7
Ex-smoker	3,509	22.3	1,185	23.7	1,443	28.8
Not recorded	1,500	9.5				
Body Mass Index[c]						
Underweight (<18.5)	256	1.6	48	18.8	99	38.7
Healthy weight (18.5-24.9)	3,173	20.2	685	21.6	1,045	32.9
Overweight (25-29.9)	3,652	23.2	847	23.2	1,341	36.7
Obese (≥30)	5,311	33.8	1398	26.3	2,320	43.7
Not recorded	3,333	21.2	720	21.6	495	14.9
Any comorbidity? [c,d,e] - Yes	6,804	43.3	1,652	24.3	3,205	47.1
No	8,921	56.7	2,046	22.9	2,095	23.5
Mental health related illness[f]	3,431	21.8	-	-	-	-
Asthma	2,699	17.2	-	-	-	-
CHD	281	1.8	-	-	-	-
CKD	132	0.8	-	-	-	-
COPD	299	1.9	-	-	-	-
Epilepsy	238	1.5	-	-	-	-
Osteoporotic fracture	462	2.9	-	-	-	-
Stroke	135	0.9	-	-	-	-
Diabetes	824	5.2	-	-	-	-
On COVID-19 shielding list? [g] - Yes	635	4.0	129	20.3	267	42.1
No	15,090	96.0	3,569	23.7	5,033	33.4
**Area level characteristics[h,i]**						
Resident health board						
Aneurin Bevan	2,819	17.9	758	26.9	980	34.8
Betsi Cadwaladr	3,683	23.4	665	18.1	1,140	31.0
Cardiff and Vale	1,796	11.4	475	26.5	650	36.2
Cwm Taf Morgannwg	2,702	17.2	755	27.9	1,078	39.9
Hywel Dda	1,965	12.5	392	20.0	561	28.6
Powys	662	4.2	104	15.7	105	15.9
Swansea Bay	2,098	13.3	549	26.2	786	37.5
Deprivation quintile[k]						
1 (most deprived)	4,168	26.5	1,143	27.4	1,541	37.0
2	3,970	25.2	991	25.0	1,392	35.1
3	3,301	21.0	697	21.1	1,005	30.5
4	2,554	16.2	503	19.7	780	30.5
5 (most affluent)	1,732	11.0	364	21.0	582	33.6
Rurality classification						
Rural	4,932	31.4	947	19.2	1,435	29.1
Urban	10,793	68.6	2,751	25.5	3,865	35.8

CHD= Coronary Heart Disease; CKD= Coronary Kidney Disease; COPD= Chronic Obstructive Pulmonary Disease. *Numbers suppressed as <10. Mixed and Other categories are combined. (a) Between 1st March 2020 to 31st November 2021; (b) Data source = Domiciliary Social Care Workers (DSCW) data and include data that is self-reported by DCWs at 1st April. If missing, then other sources (ONS 2011 Census Wales (CENW), Wales Longitudinal General Practice (WLGP), Patient Episode Dataset for Wales (PEDW), Emergency Department

For peer review only - http://bmjopen.bmj.com/site/about/guidelines.xhtml

Dataset (EDDS)) were used to impute (at index date=1st March 2020); (c) Data source = WLGP; (d) Data source=PEDW; (e) Most recent occurring between 1st January 2000 and 1st March 2020; (f) Contacts and diagnoses for mental health and admissions; (g) People at a higher risk of getting seriously ill from COVID-19; (h) Based on residential address at 1st March 2020; (i) Data source =Welsh Demographic Service Dataset (WDSD); (k) The Welsh Index of Multiple Deprivation (WIMD) is the official measure of relative deprivation for small areas in Wales. Quintiles are derived by proportioning the derivation score into five equal groups.

Table 2. Key outcomes by pandemic wave

	Short term (Wave 1) 1st March to 31st August 2020		Short term (Wave 1 and 2) 1st March 2020 to 28th February 2021		Long term 1st March 2020 to 30th November 2021	
Outcome	N	%	n	%	n	%
Confirmed COVID-19 infection	182	1.2	2,196	14.0	3,698	23.5
Suspected COVID-19 infection	326	2.1	2,335	14.8	3,822	24.3
Mental health[a]	3641	23.2	4,392	27.9	5,300	33.7
Self-harm	20	0.1	35	0.2	64	0.4
Fit notes	857	5.4	1,551	9.9	2,297	14.6
Non-COVID-19 respiratory infections[b]	126	0.8	300	1.9	467	3.0
All-cause mortality	*	*	*	*	23	0.1

*Short term results suppressed as de-identifiable by subtraction from the long term results.

(a) Contacts and diagnosis for mental health, psychotropic medication and admissions; (b) Respiratory infections not necessarily recorded as COVID-19 infection.

Suspected COVID-19 infection

Suspected COVID-19 infections increased over the pandemic from 2% in early pandemic to 15% by at the end of February 2021, rising to 24% by the end of November 2021 (Table 2). Examining the prevalence of suspected COVID-19 infection added little to the clinical picture given the relatively widespread availability of COVID-19 testing across the follow-up period and uncertainty regarding the fields that were used (i.e. just having a COVID-19 test was not a good indicator of having COVID-19 infection). No further modelling was therefore conducted.

Mental health including self-harm

Contacts and diagnosis for mental health, psychotropic medication, and admissions were 23% in August 2020, rising to 28% and 34% by the end of February and November 2021 respectively (Table 2). Increases were also observed for self-harm (from 0.2% to 0.4%) over the same time period (Table 2). The increase in mental health issues over time was also apparent when examining the monthly number of mental health related medication and contacts with GPs; prescribing mental health related medication was recorded in 13% of all DCWs in March 2016 rising to 20% in March 2020, prescriptions increasing on average per month by 17.6 (95% CI: 15.5 to 19.7, p-value <0.001) (Figure 2a). No evidence of a change was observed immediately following the start of the pandemic (March

For peer review only - http://bmjopen.bmj.com/site/about/guidelines.xhtml

to April 2020) but there was evidence of an increase in prescriptions after April 2020 (compared to the pre–pandemic) with an average monthly increase of 29.3 (20.7 to 38.0, p-value <0.001). Similar patterns were observed for mental health related GP contacts. Mental health related hospital admissions were lower in March 2016 (around 15 admission per month) and significantly increased on average per month by 0.15 (0.06 to 0.25, 0.002) (Figure 2b). There was evidence to suggest a decrease in admissions immediately following the start of the pandemic but increased on average by 0.99 admissions per month (0.44 to 1.53, <0.001), returning to pre-pandemic by the end of the study period.

Variations in mental health contacts were observed across many of the key demographics (e.g., lower in males and older care workers), and consistently higher in care workers with a disability reported at registration, with a comorbidity or shielding (Table 1). There was no variation in mental health contacts for the care workers by their qualification status; care workers in the local authority sector had slightly elevated risk of mental ill-health compared to those employed by the private sector at registration.

Fit notes

The issuing of fit notes as a general marker of medically confirmed illness, increased over the time-period from 5% in August 2020, to 10% by at the end of February and 15% by the end of November 2021 (Table 2). Fit notes were more prevalent in females, those who registered following the induction pathway or held a Level 2 Qualification (compared to those with competency confirmed by a manager), those employed by the Local Authority Social Services at registration (compared to those employed in the private sector), DCWs with a comorbidity or were shielding, and those with a BMI greater than 30 (compared to those with a healthy BMI: 18.5 to 24.9) (supplementary material 5). Unlike confirmed COVID-19 infection, fit notes were more likely in those that were older (aged 45 and over). Both Betsi Cadwaladr and Powys health boards also had a higher rate of fit notes issued for DCWs, whereas Swansea Bay had less (when compared to Aneurin Bevan).

Deaths

Both all-cause and COVID-19-related mortality involved 0.1% and 0.034% respectively, of the care workers over the longer-term study period (1st March 2020 and 30th November 2021) (Table 2).

Comparison to other UK nations

Up-to-date DCW workforce from Scotland [27] and England [28] show comparable case-mix of DCW populations to (and in some cases identical to) that presented for Wales with respect to age, sex, and ethnicity (supplementary material 6) and we would expect the prevalence of confirmed COVID-19 in these nations to therefore be similar. For DCWs in England based on a population of 590,000, the impact of mental health illness is around 200,000. However, data from Northern Ireland (NI)

For peer review only - http://bmjopen.bmj.com/site/about/guidelines.xhtml

showed that DCWs are younger on average (mode 20-29 years) [29], and the prevalence of COVID-19 infection may have been greater in this DCW workforce.

DISCUSSION

Principal findings

Confirmed COVID-19 infection rates increased from 14% during the first two pandemic waves (1st March 2020 to 28th February 2021) to 24% by the end of November 2021. Infection rates varied by personal characteristics of care workers (lower in males and in older care workers), by where care workers lived (lower in health boards such as Powys, in rural areas, in more affluent areas), and were higher for care workers employed by local authority social services departments compared to staff employed in the private sector. Suspected COVID-19 infections similarly increased to 24% over the study period. One third (34%) of all care workers required support for a mental health condition, with rates of attending a GP or receiving a relevant prescription higher compared to the four years preceding pandemic onset. Rates in mental health contacts varied by key demographics and were consistently higher in care workers with a comorbidity, disability or who were shielding. Local authority care workers had a slightly elevated risk of mental health problems compared to those employed by the private sector. Events for self-harm also increased, from 0.2% to 0.4%. The issuing of fit notes as a general marker of medically confirmed illness, increased by 10% over the COVID-19 pandemic and were more prevalent in female DCWs, those with an underlying comorbidity or at a higher risk of getting seriously ill from COVID-19. Unlike confirmed COVID-19 infection, fit notes were more likely in those that were older (aged 45 and over). There was no evidence to suggest a miscoding of COVID-19 infection in the early pandemic. The mortality rate amongst care workers was no greater than that observed amongst the general population of 15- to 64-year-olds in Wales (which was 0.034%). Data from DCW workforces in England and Scotland showed a similar demographic with respect to age, sex and ethnicity. The impact of COVID-19 infection would therefore be similar to that observed in Wales. The NI workforce was younger and therefore we may expect a high prevalence rate.

Strengths and weaknesses

A principal strength of the study was the comprehensive and contemporaneous coverage of the DCW population in Wales through linkage to registration data. This contrasts with approaches reliant on census data which may be incomplete, at risk of being out of date or on sample surveys with varying levels of non-response [3,4]. With few records lost to linkage failure or opt-out, the study cohort reflects well the population of DCWs reported by the regulator [30]. Access to EHRs in the SAIL Databank allowed for broader assessment of health than previously reported by ONS, which

For peer review only - http://bmjopen.bmj.com/site/about/guidelines.xhtml

understandably focused on mortality in the early period of the pandemic [3]. Accessing SAIL data utilised coding developed for existing conditions and novel indications (e.g. COVID-19) enabling rapid application and greater confidence than coding developed from scratch [31][31].

The registration process was introduced by Social Care Wales to meet regulatory rules that came into force from 1st April 2020 [5]. Registration as a DCW reflected the role's focus on providing care whereas workers providing support only were not required to register. However, our qualitative interviews revealed greater heterogeneity in the registered workforce than anticipated [6]. Workers in supported shared living homes were registered, as well as workers travelling to care for individuals living in their own home, detail unavailable from registration data alone. In all cases, members of the study cohort are registered as DCWs and despite some potential differences in working role amongst the cohort, common factors are likely to involve close proximity with clients in their domestic setting. Differences may include provision of intimate personal care and assistance with bodily functions by DCWs only. Our inability to further differentiate between workers consistently working in either a care or support only role during the study period is a limitation, especially if this represents a difference in risk exposure. Integrating qualitative interviews (which have been separately reported) with the quantitative routine data analyses has provided insight into the working experience and context of DCWs and how that may translate to variability in infection risk and mitigation [6]. It has illuminated the heterogeneity of roles being undertaken by those registered under the umbrella role of DCW and therefore we would consider a strength.

Administrative records do not usually capture subjective patient reported outcomes or wellbeing concerns. This may be a greater consideration if care workers felt reluctant or less able to present concerns to healthcare professionals during the pandemic, as has been observed in other settings [32]. Both quantitative and qualitative studies, involving a range of community-based health and social care staff provide ample evidence of impacts of the pandemic on the wellbeing of professionals [2,6,33–35]. Findings from our routine data study builds a more complete picture of the health consequences of the pandemic for care workers.

Outcome rates varied considerably based on characteristics and circumstances of care workers, such as age and geographical location. Such differences may not be directly attributable to the working conditions of care workers or even directly addressable. Nevertheless, it highlights that risks for care workers remain a vital consideration for employers.

Some methodological caveats include that we could not distinguish between DCWs who did not consult with their GP or where their GP had not contributed data to SAIL. This will result in downward biased GP outcome rates (e.g. if a mental health diagnosis truly occurred then a mental health diagnosis will be misclassified as having not occurred). Secondly, if missing GP data are not at random (e.g., if there is less GP coverage in some regions), it is more likely that we would have

For peer review only - http://bmjopen.bmj.com/site/about/guidelines.xhtml

missed mental health diagnosis or fit notes from DCWs with certain characteristics, resulting in bias in our parameter estimates (ORs/HRs) for these outcomes. Thirdly, DCWs were followed up for different durations (due to migration in and out of Wales or from contributing/non-contributing GPs, or deaths). Whilst the time to event analyses (and corresponding HRs) account for this by censoring, summary statistics and ORs do not. Lastly, cohort ageing and differences in cohort entry or exit dates would likely lead to upwards bias in the overall trends in the time series analyses.

Findings in context

In contrast to our findings, ONS data from the initial three-month period of the pandemic suggested that mortality rates for individuals employed as care workers and home carers were higher than those found in the general population [3]. However, data on occupational classification was missing for a large proportion dying with some COVID-19 involvement and, even where available, data on occupation may have been incorrect due to role and job changes since census data were provided (i.e. every ten years). Our findings are more in line with the initial estimates provided by a Public Health England (PHE) survey of DCWs in July 2020, where the rate of confirmed COVID-19 on PCR testing (regardless of symptoms) was low (0.1%, 95% CI=0.01 to 0.36%) [4]. The PHE study differed from out study in several ways. In the PHE study, DCWs were identified through purposive sampling of care providing organisations by region (i.e. to achieve similar numbers of respondents by region), initially approached based on convenience. Within participating organisations, a convenience sample of staff were approached to take part in the survey which involved staff returning a nasal swab and short questionnaire during a two-week study window (2nd to 16th June, 2020). DCW staff included in the PHE study were 84.3% female, with a median age of 41 years and 75.8% of those providing details about ethnicity were white. Two of the 2,015 DCWs returning a swab in the two-week study period were positive for SARS-CoV-2 on PCR testing, while 41 DCWs reported symptoms of COVID-19 in the 14 days prior to the swab being taken.

Differing study methodologies may reduce the value of direct comparisons, while also offering different levels of insight. Clarity of the study population is one such parameter. At the commencement of the pandemic consideration of work-place characteristics, opportunities to mitigate risk and rapidly emerging evidence of infection and associated mortality drove interest in social care and in our study, the work of DCWs. Subsequently, a semi-quantitative job exposure matrix (COVID-19-JEM) was developed to estimate the likelihood of workers becoming infected with SARS-CoV-2 in an occupational setting [36]. ‘Home workers’ and ‘home carers’ were assessed as scoring 14 on the matrix, indicating a relatively high risk of exposure due to characteristics inherent in their job. However, the threshold used (13+) would include half of the UK workforce and the matrix’s factors for transmission risk and mitigation could clearly mask a heterogeneity of risk as suggested in our own qualitative work [6]. An analysis of occupational differences in SARS-CoV-2 infection used the

For peer review only - http://bmjopen.bmj.com/site/about/guidelines.xhtml

UK ONS COVID-19 infection survey (CIS) data for adults aged 20-64 years old [37,38]. The CIS period covered (April 2020 to November 2021) was almost equivalent to that in our own study. Using the four-digit Standard Occupational Classification (SOC) 2010, the study reported an elevated risk for social care staff (adjusting for multiple demographic factors) with an HR of 1.14 (95% CI: 1.04 to 1.24) when compared to non-essential workers. Of the 8,005 social care staff included in the CIS, 690 (8.6%) had at least one positive PCR test during the study period. Interestingly, the elevated odds of an infection for social care staff reduced over the time assessed (as it did for healthcare workers), while it persisted for some other occupations such as education. Finally, in comparison, the report found equivocal evidence overall regarding infection rate for healthcare support workers (HR of 1.13; 95% CI: 0.96 to 1.32).

Comparing rates across studies may not always be helpful due to the differences in methods used and the time intervals covered. For example, while the ONS survey used RT-PCR testing, other studies have used other methods (e.g., self-administered point-of-care lateral flow immunoassay testing in REACT-2) [38,39]. Nevertheless, the higher rate of confirmed infection for DCWs in our study compared to social care staff in the ONS survey cannot be attributable to differences in time period covered as they were virtually equivalent. Whilst DCWs in Wales were likely to have tested several times in that time period, so would have participants in the ONS study (the CIS involved monthly testing after an initial baseline set of 5 weekly assessments). The lack of granularity associated with SOC codes and COVID-19-JEM to reflect contamination risk in specific settings is probably a limiting factor in such analyses. Our study sample remained specifically focused on workers registered as DCWs, which may reflect a higher level of occupational risk than for other workers classed as in caring personal services or in social care. In this context, within study population differences (e.g., by sex, gender) are less problematic to interpret especially given the comprehensive coverage of the DCW population and the large numbers of well characterised DCWs included.

Previous research on care worker health outcomes has often focused on musculoskeletal problems and dealing with challenging behaviour [40]. Nevertheless, pre-pandemic work has emphasised some broader harms experienced by a range of community-based home care workers (e.g. infection control) and the benefits that workers derive from their work [41–43]. Sterling and colleagues used US national CDC Behavioral Risk Factor Surveillance Survey data to explore outcomes of home health care workers employed by home care agencies. [41]. Despite role differences between the US sample and DCWs in Wales, the pre-pandemic levels of mental ill-health are interestingly similar. The reasons for such similarities are unclear and could simply reflect broader population similarities in the prevalence of mental ill-health rather than occupationally driven factors.

CONCLUSION

For peer review only - http://bmjopen.bmj.com/site/about/guidelines.xhtml

This study presents evidence of the direct and indirect impact of the COVID-19 pandemic upon DCWs in Wales. Higher rates of confirmed COVID-19 infection amongst DCWs did not translate into mortality rates greater than for the broader Welsh population. High baseline rates of mental ill-health further increased over time, a burden that fell unevenly across the workforce. It remains to be determined whether any facet of the employment role, such as staff training, occupational risk assessment or testing procedures, may have contributed to these differences or provided an opportunity to intervene. Evidence from our own work and that of others, supports the value of co-produced solutions which draw on the direct experiences of care workers to support occupational related well-being [44]. Systemic drivers (e.g. public funding for social care, staffing levels, levels of pay) and situational aspects of the role such as peripatetic working will not change quickly or even at all. With few evidence-based supportive approaches tailored to the circumstances of care workers [45] [46] optimising or innovating approaches to support the UK community of care workers may offer considerable long-term benefits to workforce and clients alike.

For peer review only

Funding

This research was funded by the Economic & Social Research Council (ESRC), as part of UK Research & Innovation's rapid response to COVID-19 (ES/V015206/1).

Research ethics approval

The study received approval from the School of Medicine Research Ethics Committee, Cardiff University [SMREC 20/114]. Approval was obtained from the SAIL Information Governance Review Panel (IGRP), Swansea University (Project ID: 1126) and data access was granted to named staff. Approval from the ONS Research Accreditation Panel (ONS RAP) was provided (16/12/2020) and approval from Social Care Wales was provided (26/01/2021). This process was generic and not specific to the OSCAR study.

Competing interests

The authors declare that they have no conflict of interest and that they satisfy the conditions for authorship.

Data availability statement

The routine data used in this study are available in the SAIL Databank at Swansea University, Swansea, UK. All proposals to use SAIL data are subject to review by an IGRP. Before any data can be accessed, approval must be given by the IGRP. The IGRP gives careful consideration to each project to ensure proper and appropriate use of SAIL data. When access has been approved, it is gained through a privacy protecting safe haven and remote access system referred to as the SAIL Gateway. SAIL has established an application process to be followed by anyone who would like to access data via SAIL: https://www.saildatabank.com/application-process. This study has been approved by the IGRP as project 1126.

Contributors

RCJ and MR were co-chief investigators of the study and led the drafting of the main manuscript. MR, RCJ, AJ, FLW, AA, KH, LBH, and DT conceived and contributed to the design of the study. AA was responsible for managing access to data within the SAIL Databank and advising on constituent data sets. HJ was responsible for data management and preparing data for analysis. FLW was responsible for study management. SS and RCJ were responsible for developing the statistical analysis plan and conducting the main analysis. All authors (MR, RCJ, AJ, FLW, AA, KH, DT, SS, HJ, LBH, HP)

For peer review only - http://bmjopen.bmj.com/site/about/guidelines.xhtml

contributed to the design and conduct of the study and interpretation of the analyses. All authors were responsible for reviewing and revising drafts of the manuscript and providing final approval.

Acknowledgements

The Centre for Trials Research receives funding from Health and Care Research Wales and Cancer Research UK. The data acquisition and COVID-19 research was supported by Health Data Research UK [HDR-9006], which receives its funding from HDR UK Ltd, and the ADR Wales programme of work) funded ADR UK (grant ES/S007393/1). The study team gratefully acknowledge the work of the Study Advisory Group for providing independent oversight and input; the Implementation Reference Group for reviewing early findings and co-producing recommendations for short and longer-term outcomes; and all domiciliary care workers who contributed their data for inclusion in the study analyses. This study makes use of anonymised data held in the Secure Anonymised Information Linkage (SAIL) Databank. We would like to acknowledge all the data providers who make anonymised data available for research. We acknowledge the support of Social Care Wales in advising us at different stages of the study, supporting access to the registration data and aiding our reflection on study findings. This is an open access article distributed in accordance with the Creative Commons Attribution 4.0 Unported (CC BY 4.0) license, which permits others to copy, redistribute, remix, transform and build upon this work for any purpose, provided the original work is properly cited, a link to the licence is given, and indication of whether changes were made.
See: https://creativecommons.org/licenses/by/4.0/.

only

For peer review only - http://bmjopen.bmj.com/site/about/guidelines.xhtml

References

1 Homecare. Home care facts and stats: number of providers, service users & workforce. 2021.https://www.homecare.co.uk/advice/home-care-facts-and-stats-number-of-providers-service-users-workforce (accessed 15 Jun 2022).

2 Gillen P, Neill RD, Manthorpe J, *et al.* Decreasing Wellbeing and Increasing Use of Negative Coping Strategies: The Effect of the COVID-19 Pandemic on the UK Health and Social Care Workforce. *Epidemiologia* 2022;**3**:26–39. doi:10.3390/epidemiologia3010003

3 Office for National Statistics. Coronavirus (COVID-19) related deaths by occupation, England and Wales. https://www.ons.gov.uk/peoplepopulationandcommunity/healthandsocialcare/causesofdeath/datasets/coronaviruscovid19relateddeathsbyoccupationenglandandwales (accessed 22 Dec 2020).

4 Public Health England. Pilot point prevalence survey of COVID-19 among domiciliary care staff in England. 2020. https://www.gov.uk/government/publications/covid-19-prevalence-survey-domiciliary-care-staff-in-england (accessed 22 Dec 2020).

5 Social Care Wales. Why we register. 2022.https://socialcare.wales/registration/why-we-register (accessed 16 Jun 2022).

6 Prout H, Lugg-Widger F V., Brookes-Howell L, *et al.* "I don't mean to be rude, but could you put a mask on while I'm here?" A qualitative study of risks experienced by domiciliary care workers in Wales during the COVID-19 pandemic. *Health Soc Care Community* Published Online First: 24 November 2022. doi:10.1111/hsc.14109

7 Lyons RA, Jones KH, John G, *et al.* The SAIL databank: linking multiple health and social care datasets. *BMC Med Inform Decis Mak* 2009;**9**:3. doi:10.1186/1472-6947-9-3

8 SAIL Databank. COVID-19 Pathology Test Results. 2022.https://data.ukserp.ac.uk/Asset/View/53 (accessed 16 Jun 2022).

9 SAIL Databank. Patient Episode Database for Wales. 2022.https://data.ukserp.ac.uk/Asset/View/15 (accessed 16 Jun 2022).

10 SAIL Databank. Annual District Death Extract. 2022.https://data.ukserp.ac.uk/Asset/View/3 (accessed 16 Jun 2022).

11 SAIL Databank. Primary Care GP Dataset. 2022.https://data.ukserp.ac.uk/Asset/View/17 (accessed 16 Jun 2022).

12 John A, McGregor J, Fone D, *et al.* Case-finding for common mental disorders of anxiety and depression in primary care: an external validation of routinely collected data. *BMC Med Inform Decis Mak* 2016;**16**:35. doi:10.1186/s12911-016-0274-7

13 Rahman MA, Todd C, John A, *et al.* School achievement as a predictor of depression and self-harm in adolescence: linked education and health record study. *The British Journal of Psychiatry* 2018;**212**:215–21. doi:10.1192/bjp.2017.69

14 Clift AK, Coupland CAC, Keogh RH, *et al.* Living risk prediction algorithm (QCOVID) for risk of hospital admission and mortality from coronavirus 19 in adults: national derivation and validation cohort study. *BMJ* 2020;:m3731. doi:10.1136/bmj.m3731

15 Thomas KH, Davies N, Metcalfe C, *et al.* Validation of suicide and self-harm records in the Clinical Practice Research Datalink. *Br J Clin Pharmacol* 2013;**76**:145–57. doi:10.1111/bcp.12059

For peer review only - http://bmjopen.bmj.com/site/about/guidelines.xhtml

16 Marchant A, Turner S, Balbuena L, *et al.* Self-harm presentation across healthcare settings by sex in young people: an e-cohort study using routinely collected linked healthcare data in Wales, UK. *Arch Dis Child* 2020;**105**:347–54. doi:10.1136/archdischild-2019-317248

17 London School of Hygiene and Tropical Medicine Data Compass. Data Compass. https://datacompass.lshtm.ac.uk/id/eprint/2193/ (accessed 16 Jun 2022).

18 London School of Hygiene and Tropical Medicine. Data Compass. https://datacompass.lshtm.ac.uk/id/eprint/2037/1/copd_hes.txt (accessed 16 Jun 2022).

19 London School of Hygiene and Tropical Medicine. Data Compass. https://datacompass.lshtm.ac.uk/id/eprint/940 (accessed 16 Jun 2022).

20 Social Care Wales. Qualifications needed. 2022.https://socialcare.wales/registration/qualifications-needed#section-29926-anchor (accessed 8 Jul 2022).

21 Benchimol EI, Smeeth L, Guttmann A, *et al.* The REporting of studies Conducted using Observational Routinely-collected health Data (RECORD) Statement. *PLoS Med* 2015;**12**:e1001885. doi:10.1371/journal.pmed.1001885

22 Elm E von, Altman DG, Egger M, *et al.* Strengthening the reporting of observational studies in epidemiology (STROBE) statement: guidelines for reporting observational studies. *BMJ* 2007;**335**:806–8. doi:10.1136/bmj.39335.541782.AD

23 IBM Corp. Db2 for z/OS (Version 11.1). 2022.

24 R Core Team. R: A language and environment for statistical computing. R Foundation for Statistical Computing, Vienna, Austria,. Published Online First: 2022.www.R-project.org (accessed 8 Jul 2022).

25 Wickham H, Averick M, Bryan J, *et al.* Welcome to the Tidyverse. *J Open Source Softw* 2019;**4**:1686. doi:10.21105/joss.01686

26 StataCorp. Stata Statistical Software: Release 17. College Station, TX: StataCorp LLC. 2021.

27 Scottish Social Services Council. Adults' services workforce tables - 2019 | Scottish Social Services Workforce Data (). 2019. Adults' services workforce tables - 2019 | Scottish Social Services Workforce Data (sssc.uk.com) https://data.sssc.uk.com/data-publications/248-adults-services-workforce-in-scotland-tables-2019 (accessed 16 Jun 2022).

28 Skills for Care. The state of the adult social care sector and workforce in England. Published Online First: 2022.https://www.skillsforcare.org.uk/adult-social-care-workforce-data/Workforce-intelligence/publications/national-information/The-state-of-the-adult-social-care-sector-and-workforce-in-England.aspx (accessed 16 Jun 2022).

29 Department of Health. Rapid learning review of domiciliary care in Northern Ireland. https://www.health-ni.gov.uk/rli-dom-care (accessed 16 Jun 2022).

30 Social Care Wales. Domiciliary Care Workers on the Register. 2020. 2020. https://socialcare.wales/cms_assets/file-uploads/Domiciliary-Care-Workers-on-the-Register.pdf (accessed 16 Jun 2022).

31 Lyons J, Akbari A, Torabi F, *et al.* Understanding and responding to COVID-19 in Wales: protocol for a privacy-protecting data platform for enhanced epidemiology and evaluation of interventions. *BMJ Open* 2020;**10**:e043010. doi:10.1136/bmjopen-2020-043010

For peer review only

For peer review only - http://bmjopen.bmj.com/site/about/guidelines.xhtml

32 Quinn-Scoggins HD, Cannings-John R, Moriarty Y, *et al.* Cancer symptom experience and help-seeking behaviour during the COVID-19 pandemic in the UK: a cross-sectional population survey. *BMJ Open* 2021;**11**:e053095. doi:10.1136/bmjopen-2021-053095

33 McFadden P, Neill RD, Moriarty J, *et al.* A Cross-Sectional Examination of the Mental Wellbeing, Coping and Quality of Working Life in Health and Social Care Workers in the UK at Two Time Points of the COVID-19 Pandemic. *Epidemiologia* 2021;**2**:227–42. doi:10.3390/epidemiologia2030017

34 Aughterson H, McKinlay AR, Fancourt D, *et al.* Psychosocial impact on frontline health and social care professionals in the UK during the COVID-19 pandemic: a qualitative interview study. *BMJ Open* 2021;**11**:e047353. doi:10.1136/bmjopen-2020-047353

35 Markkanen P, Brouillette N, Quinn M, *et al.* "It changed everything": The Safe Home Care qualitative study of the COVID-19 pandemic's impact on home care aides, clients, and managers. *BMC Health Serv Res* 2021;**21**:1055. doi:10.1186/s12913-021-07076-x

36 Oude Hengel KM, Burdorf A, Pronk A, *et al.* Exposure to a SARS-CoV-2 infection at work: development of an international job exposure matrix (COVID-19-JEM). *Scand J Work Environ Health* 2022;**48**:61–70. doi:10.5271/sjweh.3998

37 Rhodes S, Wilkinson J, Pearce N, *et al.* Occupational differences in SARS-CoV-2 infection: analysis of the UK ONS COVID-19 infection survey. *J Epidemiol Community Health (1978)* 2022;**76**:841–6. doi:10.1136/jech-2022-219101

38 Pouwels KB, House T, Pritchard E, *et al.* Community prevalence of SARS-CoV-2 in England from April to November, 2020: results from the ONS Coronavirus Infection Survey. *Lancet Public Health* 2021;**6**:e30–8. doi:10.1016/S2468-2667(20)30282-6

39 Whitaker M, Elliott J, Chadeau-Hyam M, *et al.* Persistent COVID-19 symptoms in a community study of 606,434 people in England. *Nat Commun* 2022;**13**:1957. doi:10.1038/s41467-022-29521-z

40 Health and Safety Executive. Domiciliary care provided in people's own homes. https://www.hse.gov.uk/healthservices/domiciliary-care.htm (accessed 16 Jun 2022).

41 Sterling MR, Li J, Cho J, *et al.* Prevalence and Predictors of Home Health Care Workers' General, Physical, and Mental Health: Findings From the 2014–2018 Behavioral Risk Factor Surveillance System. *Am J Public Health* 2021;**111**:2239–50. doi:10.2105/AJPH.2021.306512

42 Denton MA, Zeytinoğlu IU, Davies S. Working in Clients' Homes: The Impact on the Mental Health and Well-Being of Visiting Home Care Workers. *Home Health Care Serv Q* 2002;**21**:1–27. doi:10.1300/J027v21n01_01

43 Quinn MM, Markkanen PK, Galligan CJ, *et al.* Occupational health of home care aides: results of the safe home care survey. *Occup Environ Med* 2016;**73**:237–45. doi:10.1136/oemed-2015-103031

44 Hayes L TAWH. Care and support workers' perceptions of health and safetyissues in socialcareduring the COVID-19 pandemic. Initial . 2020. https://media.www.kent.ac.uk/se/11148/CareworkersHealthandSafetyreport15042.pdf (accessed 16 Jun 2022).

45 Olson R, Thompson S v., Elliot DL, *et al.* Safety and Health Support for Home Care Workers: The COMPASS Randomized Controlled Trial. *Am J Public Health* 2016;**106**:1823–32. doi:10.2105/AJPH.2016.303327

For peer review only - http://bmjopen.bmj.com/site/about/guidelines.xhtml

46 Indregard A-MR, Knardahl S, Emberland JS, *et al.* Effectiveness of the Labour Inspection Authority's regulatory tools for work environment and employee health: study protocol for a cluster-randomised controlled trial among Norwegian home-care workers. *BMJ Open* 2019;**9**:e031226. doi:10.1136/bmjopen-2019-031226

Figure 1. Cumulative probabilities of confirmed COVID-19 infection over time by employment sector for domiciliary care workers

Figure 2. Changes over time (March 2016-October 2021) in mental health related hospital admissions (a) and GP prescriptions (b) for domiciliary care workers

For peer review only

Figure 1: Cumulative probabilities of confirmed COVID-19 infection over time by employment sector for domiciliary care workers

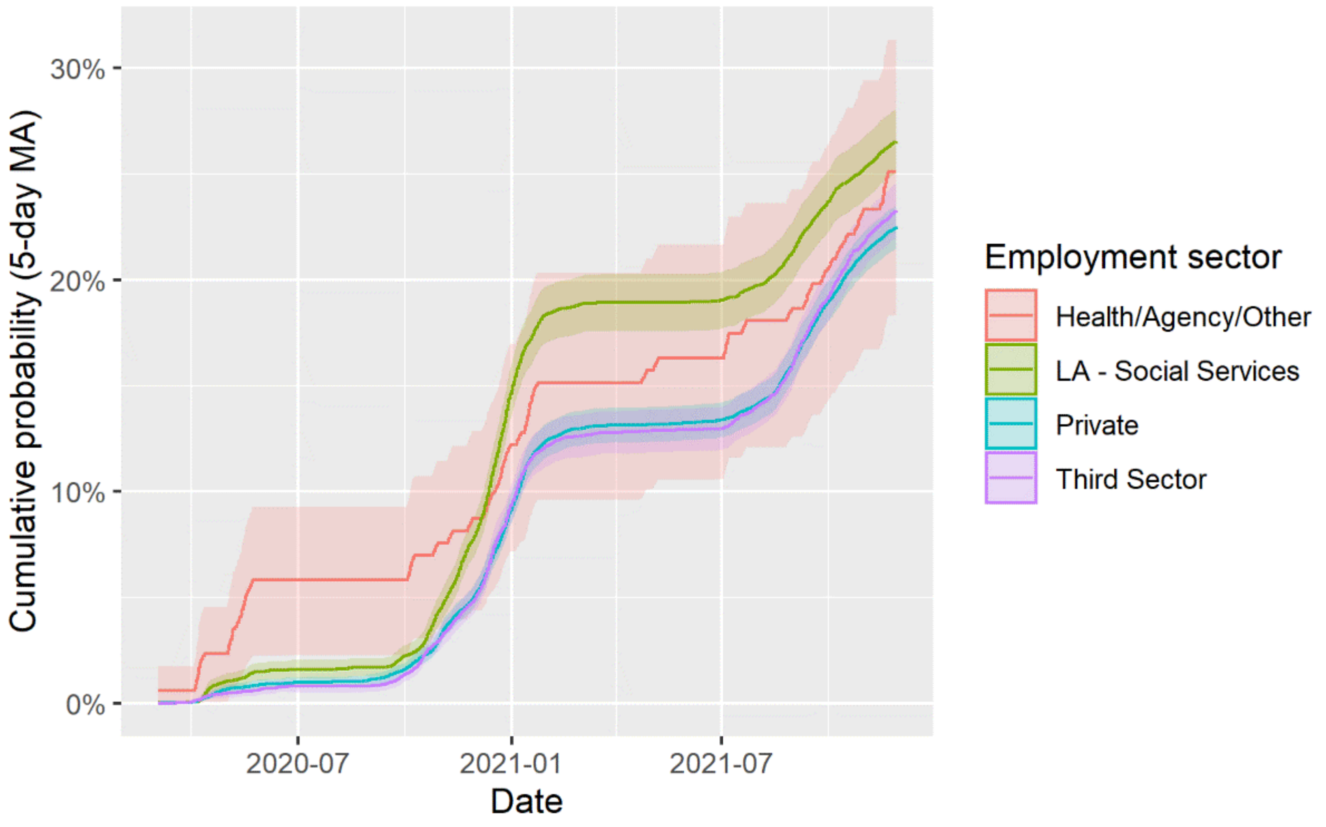

MA=moving average

view only

Figure 2a: Changes over time (March 2016-October 2021) in mental health related hospital admissions for domiciliary care workers

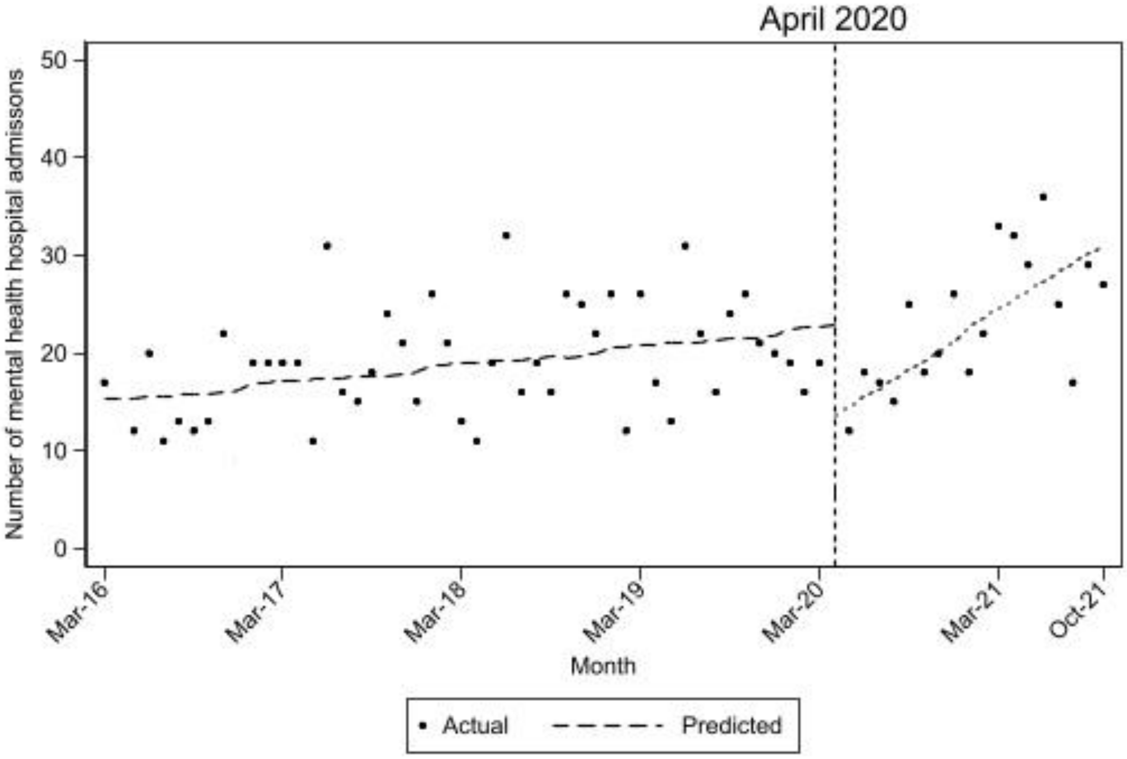

Figure 2b: Changes over time (March 2016-October 2021) in mental health related GP prescriptions for domiciliary care workers

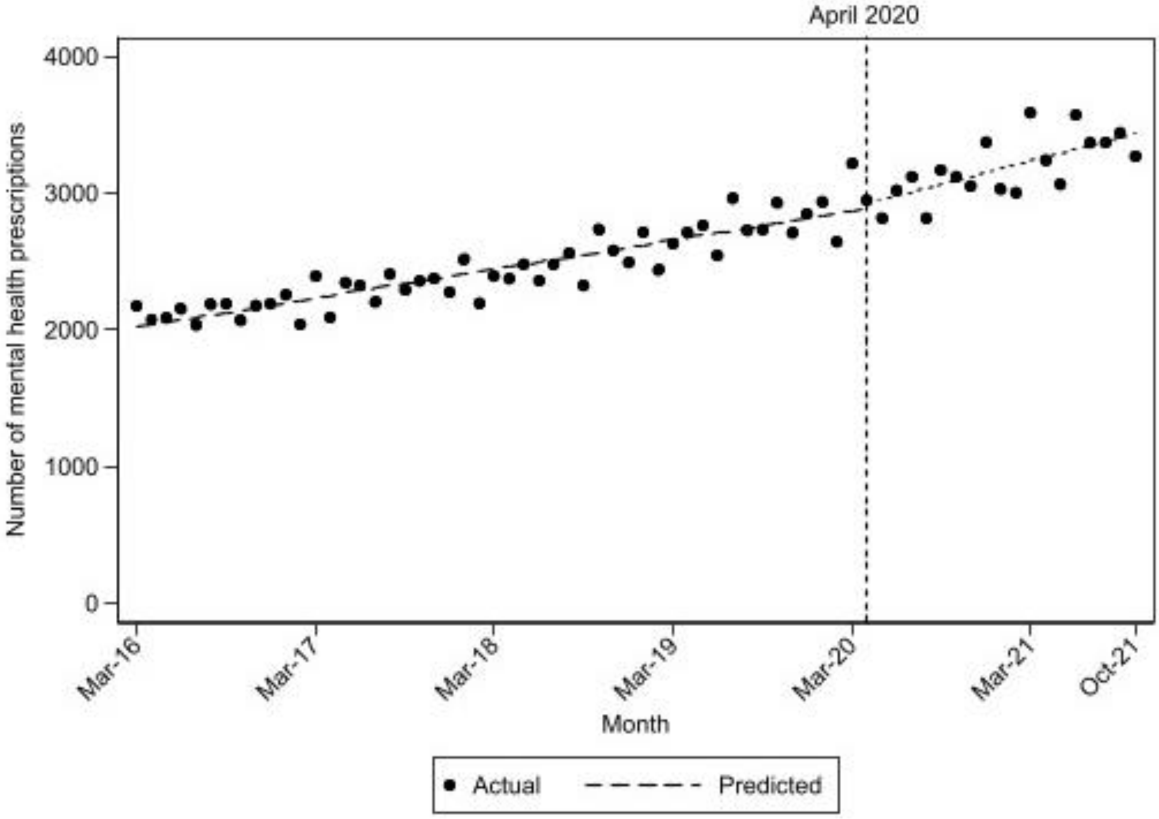

Supplementary material

For peer review only

Supplementary material 1: Data sources used in the OSCAR study

SAIL acronym	Name	Description	Meta-data link
ADDE	Annual District Death Extract	Monthly register of all deaths relating to Welsh residents, including those that died out of Wales from the Office for National Statistics (ONS).	https://web.www.healthdatagateway.org/dataset/15cf4241-abad-4dcc-95b0-8cd7c02be999
CENW	ONS 2011 Census Wales	A census in the UK is a count of all people and households. The census data held in SAIL was from the census held on 27 March 2011.	https://web.www.healthdatagateway.org/dataset/fc24482c-0f9b-445f-af7a-8598627c3b15
DSCW	Domiciliary Social Care Worker Dataset	Under the Care Standards Act 2000, SCW maintain a register of DCWs in Wales. The DSCW data was transferred securely from SCW to the SAIL Databank and made available as a core-restricted data source.	NA
EDDS	Emergency Department Dataset	Data captured for all NHS Wales Accident and Emergency department attendances. Data are collected and coded at each hospital	https://web.www.healthdatagateway.org/dataset/75c4dcb8-33bf-43f4-b2bb-db51b6621b2c
PATD	COVID-19 Test results	Daily update of all pillar 1 and pillar 2 PCR tests administered and associated results for Welsh residents and those receiving a test in Wales	https://web.www.healthdatagateway.org/dataset/f5f6d882-163d-4ef1-a53e-000fba409480
PEDW	Patient Episode Dataset for Wales	Weekly update of all inpatient and day case activity undertaken in NHS Wales plus data on Welsh residents treated in English Trusts	https://web.www.healthdatagateway.org/dataset/4c33a5d2-164c-41d7-9797-dc2b008cc852
WDSD	Welsh Demographic Service Dataset	Weekly update of all individuals registered with a Welsh general practice, inc. address and practice history	https://web.www.healthdatagateway.org/dataset/8a8a5e90-b0c6-4839-bcd2-c69e6e8dca6d
WLGP	Wales Longitudinal General Practice	Monthly update of all attendance and clinical information for SAIL submitting general practices (~80% national coverage)	https://web.www.healthdatagateway.org/dataset/33fc3ffd-aa4c-4a16-a32f-0c900aaea3d2

Supplementary material 2: Coding lists for OSCAR outcomes

Primary outcome

Confirmed-COVID: Confirmed COVID-19 will be defined as the earliest of the following events:

- Positive polymerase chain reaction (PCR) test result (Source: PATD);
- Hospital admission (main or secondary diagnostic ICD-10 code of U07.1 position in the admitted patient care component of PEDW);
- Death registration including a diagnosis ICD-10 code U07.1 (as underlying or contributing cause) from COVID-19 (Source: ADDE);
- COVID-19 diagnosis Read code (Source: WLGP).

TYPE	CODE	DESCRIPTION
ICD-10	U071	COVID-19 virus identified
READ	A795.	Coronavirus infection
READ	A7951	Disease caused by 2019-nCoV (novel coronavirus)
READ	A7952	COVID-19 confirmed by laboratory test
READ	A7953	COVID-19 confirmed using clinical diagnostic criteria

Secondary outcomes

Suspected COVID-19

We will ascertain DCWs with a suspected COVID-19 diagnosis taking the earliest of:

- Negative PCR or serology/antigen test from the COVID-19 test data (Source: PATD);
- Diagnosis ICD-10 code (U07.2) main or secondary diagnostic code position (Source: PEDW);
- Suspected COVID-19 Read codes (Source: WLGP).

TYPE	CODE	DESCRIPTION
ICD-10	U072	COVID-19 virus not identified
READ	1JX..	Suspected coronavirus infection
READ	1JX1.	Suspected coronavirus infection
READ	65PW.	Coronavirus contact
READ	65PW1	Exposure to 2019-nCoV (novel coronavirus) infection

Mental Health

(A) Hospital admissions (ICD-10)

CODE	DESCRIPTION
F20	Schizophrenia
F200	Paranoid schizophrenia
F201	Hebephrenic schizophrenia
F202	Catatonic schizophrenia
F203	Undifferentiated schizophrenia
F204	Post-schizophrenic depression

For peer review only - http://bmjopen.bmj.com/site/about/guidelines.xhtml

CODE	DESCRIPTION
F205	Residual schizophrenia
F206	Simple schizophrenia
F208	Other schizophrenia
F209	Schizophrenia, unspecified
F21X	Schizotypal disorder
F22	Persistent delusional disorders
F220	Delusional disorder
F228	Other persistent delusional disorders
F229	Persistent delusional disorder, unspecified
F23	Acute and transient psychotic disorders
F230	Acute polymorphic psychotic disorder without symptoms of schizophrenia
F231	Acute polymorphic psychotic disorder with symptoms of schizophrenia
F232	Acute schizophrenia-like psychotic disorder
F233	Other acute predominantly delusional psychotic disorders
F238	Other acute and transient psychotic disorders
F239	Acute and transient psychotic disorder, unspecified
F24X	Induced delusional disorder
F25	Schizoaffective disorders
F250	Schizoaffective disorder, manic type
F251	Schizoaffective disorder, depressive type
F252	Schizoaffective disorder, mixed type
F258	Other schizoaffective disorders
F259	Schizoaffective disorder, unspecified
F28X	Other nonorganic psychotic disorders
F29X	Unspecified nonorganic psychosis
F30	Manic episode
F300	Hypomania
F301	Mania without psychotic symptoms
F302	Mania with psychotic symptoms
F308	Other manic episodes
F309	Manic episode, unspecified
F31	Bipolar affective disorder
F310	Bipolar affective disorder, current episode hypomanic
F311	Bipolar affective disorder, current episode manic without psychotic symptoms
F312	Bipolar affective disorder, current episode manic with psychotic symptoms
F313	Bipolar affective disorder, current episode mild or moderate depression
F314	Bipolar affective disorder, current episode severe depression without psychotic symptoms
F315	Bipolar affective disorder, current episode severe depression with psychotic symptoms
F316	Bipolar affective disorder, current episode mixed
F317	Bipolar affective disorder, currently in remission
F318	Other bipolar affective disorders
F319	Bipolar affective disorder, unspecified
F32	Depressive episode
F320	Mild depressive episode

For peer review only - http://bmjopen.bmj.com/site/about/guidelines.xhtml

CODE	DESCRIPTION
F321	Moderate depressive episode
F322	Severe depressive episode without psychotic symptoms
F323	Severe depressive episode with psychotic symptoms
F328	Other depressive episodes
F329	Depressive episode, unspecified
F33	Recurrent depressive disorder
F330	Recurrent depressive disorder, current episode mild
F331	Recurrent depressive disorder, current episode moderate
F332	Recurrent depressive disorder, current episode severe without psychotic symptoms
F333	Recurrent depressive disorder, current episode severe with psychotic symptoms
F334	Recurrent depressive disorder, currently in remission
F338	Other recurrent depressive disorders
F339	Recurrent depressive disorder, unspecified

(B) GP contacts and prescriptions (Read)

CODE	DESCRIPTION
146D.	H/O: manic depressive disorder
1B12.	Nerves - nervousness
1B13.	Anxiousness
1B17.	Depressed
1B1U.	Symptoms of depression
1BH3.	Paranoid ideation
1BQ..	Loss of capacity for enjoyment
1BT..	Depressed mood
1BU..	Loss of hope for the future
2257	O/E - depressed
2258	O/E - anxious
2259	O/E - nervous
66580	Antidepressant drug treatment changed
66590	Antidepressant drug treatment started
665A0	Antidepressant drug treatment stopped
d1...	HYPNOTICS
d11..	CHLORAL HYDRATE
d111.	*CHLORAL 500mg/5mL mixture
d112.	CHLORAL PAEDIATRIC 200mg/5mL elixir
d113.	*NOCTEC 500mg capsules
d114.	CHLORAL HYDRATE 500mg capsules
d115.	*WELLDORM 143mg/5mL elixir
d116.	*WELLDORM tablets
d117.	CHLORAL HYDRATE 414mg tablets
d118.	CHLORAL HYDRATE 143mg/5mL elixir
d119.	CHLORAL HYDRATE 500mg/5mL syrup
d11A.	SOMNWELL 707mg tablets

For peer review only - http://bmjopen.bmj.com/site/about/guidelines.xhtml

CODE	DESCRIPTION
d12..	CLOMETHIAZOLE EDISYLATE [HYPNOTIC]
d121.	*HEMINEVRIN 192mg capsules
d122.	*HEMINEVRIN 250mg/5mL syrup
d123.	*HEMINEVRIN 8mg/mL infusion
d12v.	CLOMETHIAZOLE(base) 192mg capsules
d12w.	CLOMETHIAZOLE EDISILATE 250mg/5mL syrup
d12z.	CHLORMETHIAZOLE EDISYLATE 8mg/mL infusion
d13..	*DICHLORALPHENAZONE
d131.	*WELLDORM 650mg tablets
d132.	*WELLDORM 225mg/5mL elixir
d13y.	DICHLORALPHENAZONE 650mg tablets
d13z.	DICHLORALPHENAZONE 225mg/5mL elixir
d14..	*FLUNITRAZEPAM
d141.	*ROHYPNOL 1mg tablets
d14z.	*FLUNITRAZEPAM 1mg tablets
d15..	FLURAZEPAM
d151.	DALMANE 15mg capsules
d152.	DALMANE 30mg capsules
d153.	*PAXANE 15mg capsules
d154.	*PAXANE 30mg capsules
d15y.	FLURAZEPAM 15mg capsules
d15z.	FLURAZEPAM 30mg capsules
d16..	LOPRAZOLAM
d161.	LOPRAZOLAM 1mg tablets
d162.	*DORMONOCT 1mg tablets
d17..	LORMETAZEPAM
d171.	LORMETAZEPAM 500micrograms tablets
d172.	LORMETAZEPAM 1mg tablets
d173.	NOCTAMID 500micrograms tablets
d174.	*NOCTAMID 1mg tablets
d18..	NITRAZEPAM
d181.	*NITRAZEPAM 5mg capsules
d182.	NITRAZEPAM 5mg tablets
d183.	*NITRAZEPAM 10mg tablets
d184.	NITRAZEPAM 2.5mg/5mL mixture
d185.	*MOGADON 5mg capsules
d186.	MOGADON 5mg tablets
d187.	*NITRADOS 5mg tablets
d188.	*NOCTESED 5mg tablets
d189.	REMNOS 5mg tablets
d18a.	*REMNOS 10mg tablets
d18b.	*SOMNITE 5mg tablets
d18c.	*SOMNITE 2.5mg/5mL mixture
d18d.	*SUREM 5mg capsules
d18e.	*UNISOMNIA 5mg tablets

For peer review only - http://bmjopen.bmj.com/site/about/guidelines.xhtml

CODE	DESCRIPTION
d18f.	NITRAZEPAM 5mg/5mL suspension
d19..	PROMETHAZINE HCL [HYPNOTIC] see section c8i..
d1a..	TEMAZEPAM [HYPNOTIC]
d1a1.	*TEMAZEPAM 10mg capsules
d1a2.	*TEMAZEPAM 15mg capsules
d1a3.	*TEMAZEPAM 20mg capsules
d1a4.	*TEMAZEPAM 30mg capsules
d1a5.	*TEMAZEPAM 10mg/5mL elixir
d1a6.	*NORMISON 10mg capsules
d1a7.	*NORMISON 20mg capsules
d1a8.	*TEMAZEPAM PLANPAK capsules
d1a9.	TEMAZEPAM 10mg tablets
d1aa.	TEMAZEPAM 20mg tablets
d1ab.	TEMAZEPAM GELTHIX 10mg capsules
d1ac.	TEMAZEPAM GELTHIX 20mg capsules
d1ad.	TEMAZEPAM GELTHIX 15mg capsules
d1ae.	TEMAZEPAM GELTHIX 30mg capsules
d1af.	TEMAZEPAM GEL FILLED 10mg capsules
d1ag.	TEMAZEPAM GEL FILLED 20mg capsules
d1ah.	TEMAZEPAM GEL FILLED 15mg capsules
d1ai.	TEMAZEPAM GEL FILLED 30mg capsules
d1aj.	TEMAZEPAM 10mg/sachet oral solution
d1ak.	TEMAZEPAM 20mg/sachet oral solution
d1al.	EUHYPNOS 10mg/sachet oral solution
d1am.	EUHYPNOS 20mg/sachet oral solution
d1an.	EUHYPNOS 10mg/5mL oral solution
d1ao.	TEMAZEPAM 10mg/5mL sugar free oral solution
d1b..	*TRIAZOLAM
d1b1.	TRIAZOLAM 125microgram tablets
d1b2.	TRIAZOLAM 250microgram tablets
d1b3.	*HALCION 125microgram tablets
d1b4.	*HALCION 250microgram tablets
d1c..	TRICLOFOS SODIUM
d1c1.	*TRICLOFOS 500mg/5mL liquid
d1d..	ZOPICLONE
d1d1.	ZOPICLONE 7.5mg tablets
d1d2.	ZIMOVANE 7.5mg tablets
d1d3.	ZOPICLONE 3.75mg tablets
d1d4.	ZIMOVANE LS 3.75mg tablets
d1d5.	*ZILEZE 7.5 tablets
d1d6.	*ZILEZE 3.75 tablets
d1e..	*GLUTETHIMIDE [no drugs here]
d1f..	ZOLPIDEM
d1f1.	STILNOCT 5mg tablets
d1f2.	ZOLPIDEM TARTRATE 5mg tablets

For peer review only

For peer review only - http://bmjopen.bmj.com/site/about/guidelines.xhtml

CODE	DESCRIPTION
d1f3.	ZOLPIDEM TARTRATE 10mg tablets
d1f4.	STILNOCT 10mg tablets
d1g..	ZALEPLON
d1g1.	SONATA 5mg capsules
d1g2.	SONATA 10mg capsules
d1gy.	ZALEPLON 5mg capsules
d1gz.	ZALEPLON 10mg capsules
d1h..	MELATONIN
d1h1.	CIRCADIN 2mg m/r tablets
d1hz.	MELATONIN 2mg m/r tablets
d21..	DIAZEPAM [ANXIOLYTIC]
d22..	ALPRAZOLAM
d23..	BROMAZEPAM
d24..	CHLORDIAZEPOXIDE
d25..	CHLORMEZANONE
d26..	CLOBAZAM
d27..	CLORAZEPATE DIPOTASSIUM
d28..	HYDROXYZINE HCL [ANXIOLYTIC]
d29..	*KETAZOLAM
d2a..	LORAZEPAM [ANXIOLYTIC]
d2b..	*MEDAZEPAM
d2c..	MEPROBAMATE
d2d..	OXAZEPAM
d2f..	BUSPIRONE HYDROCHLORIDE
d2g..	FLUMAZENIL
d7...	TRICYCLIC ANTIDEPRESSANTS
d71..	AMITRIPTYLINE HYDROCHLORIDE [ANTIDEPRESSANT]
d711.	AMITRIPTYLINE 10mg tablets
d712.	AMITRIPTYLINE 25mg tablets
d713.	AMITRIPTYLINE 50mg tablets
d714.	*DOMICAL 10mg tablets
d715.	*DOMICAL 25mg tablets
d716.	*DOMICAL 50mg tablets
d717.	ELAVIL 10mg tablets
d718.	ELAVIL 25mg tablets
d719.	*LENTIZOL 25mg m/r capsules
d71a.	*LENTIZOL 50mg m/r capsules
d71b.	*TRYPTIZOL 75mg m/r capsules
d71c.	*TRYPTIZOL 10mg tablets
d71d.	*TRYPTIZOL 25mg tablets
d71e.	*TRYPTIZOL 50mg tablets
d71f.	*TRYPTIZOL 10mg/5mL syrup
d71g.	TRYPTIZOL 100mg/10mL injection
d71h.	AMITRIPTYLINE 25mg/5mL sugar free solution
d71i.	AMITRIPTYLINE 50mg/5mL sugar free solution

For peer review only - http://bmjopen.bmj.com/site/about/guidelines.xhtml

CODE	DESCRIPTION
d71u.	AMITRIPTYLINE 25mg m/r capsules
d71v.	AMITRIPTYLINE 50mg m/r capsules
d71w.	AMITRIPTYLINE 75mg m/r capsules
d71y.	*AMITRIPTYLINE 10mg/5mL syrup
d71z.	AMITRIPTYLINE HYDROCHLORIDE 100mg/10mL injection
d72..	*BUTRIPTYLINE
d721.	*EVADYNE 25mg tablets
d722.	*EVADYNE 50mg tablets
d72y.	*BUTRIPTYLINE 25mg tablets
d72z.	*BUTRIPTYLINE 50mg tablets
d73..	CLOMIPRAMINE HYDROCHLORIDE
d731.	*ANAFRANIL 10mg capsules
d732.	*ANAFRANIL 25mg capsules
d733.	*ANAFRANIL 50mg capsules
d734.	*ANAFRANIL 25mg/5mL syrup
d735.	*ANAFRANIL 25mg/2mL injection
d736.	*ANAFRANIL SR 75mg m/r tablets
d737.	*TRANQUAX 10mg capsules
d738.	*TRANQUAX 25mg capsules
d739.	TRANQUAX 50mg capsules
d73r.	*CLOMIPRAMINE HCL 50mg tablets
d73s.	*CLOMIPRAMINE HCL 10mg tablets
d73t.	*CLOMIPRAMINE HCL 25mg tablets
d73u.	CLOMIPRAMINE HCL 10mg capsules
d73v.	CLOMIPRAMINE HCL 25mg capsules
d73w.	CLOMIPRAMINE HCL 50mg capsules
d73x.	CLOMIPRAMINE HYDROCHLORIDE 25mg/5mL syrup
d73y.	CLOMIPRAMINE HCL 25mg/2mL injection
d73z.	CLOMIPRAMINE HCL 75mg m/r tabs
d74..	DESIPRAMINE HYDROCHLORIDE
d741.	*PERTOFRAN 25mg tablets
d74z.	DESIPRAMINE HYDROCHLORIDE 25mg tablets
d75..	DOSULEPIN HYDROCHLORIDE
d751.	PROTHIADEN 25mg capsules
d752.	PROTHIADEN 75mg tablets
d753.	*PREPADINE 25mg capsules
d754.	*PREPADINE 75mg tablets
d755.	*DOTHAPAX 25mg capsules
d756.	*DOTHAPAX 75mg tablets
d759.	*THADEN 25mg capsules
d75A.	*THADEN 75mg tablets
d75y.	DOSULEPIN HYDROCHLORIDE 25mg capsules
d75z.	DOSULEPIN HYDROCHLORIDE 75mg tablets
d76..	DOXEPIN
d761.	*SINEQUAN 10mg capsules x56CP

For peer review only - http://bmjopen.bmj.com/site/about/guidelines.xhtml

CODE	DESCRIPTION
d762.	*SINEQUAN 25mg capsules x28CP
d763.	*SINEQUAN 50mg capsules x28CP
d764.	*SINEQUAN 75mg capsules x28CP
d765.	*SINEPIN 25mg capsules
d766.	*SINEPIN 50mg capsules
d76w.	*DOXEPIN 10mg capsules
d76x.	DOXEPIN 25mg capsules
d76y.	DOXEPIN 50mg capsules
d76z.	*DOXEPIN 75mg capsules
d77..	IMIPRAMINE HYDROCHLORIDE [ANTIDEPRESSANT]
d771.	IMIPRAMINE 10mg tabs
d772.	IMIPRAMINE 25mg tablets
d773.	*PRAMINIL 10mg tablets
d774.	*PRAMINIL 25mg tablets
d775.	*TOFRANIL 10mg tablets
d776.	*TOFRANIL 25mg tablets
d777.	*TOFRANIL 25mg/5mL syrup
d77w.	IMIPRAMINE HYDROCHLORIDE 25mg/5mL oral solution
d77x.	IMIPRAMINE HYDROCHLORIDE 10mg tablets
d77y.	IMIPRAMINE HYDROCHLORIDE 25mg tablets
d77z.	IMIPRAMINE HYDROCHLORIDE 25mg/5mL syrup
d78..	IPRINDOLE
d781.	*PRONDOL 15mg tablets
d782.	*PRONDOL 30mg tablets
d78y.	*IPRINDOLE 15mg tablets
d78z.	*IPRINDOLE 30mg tablets
d79..	LOFEPRAMINE
d791.	*GAMANIL 70mg tablets x56CP
d792.	LOMONT 70mg/5mL sugar free suspension
d793.	*FEPRAPAX 70mg tablets
d794.	*GAMANIL 70mg tablets
d79y.	LOFEPRAMINE 70mg/5mL sugar free suspension
d79z.	LOFEPRAMINE 70mg tablets
d7a..	MAPROTILINE HYDROCHLORIDE
d7a1.	*LUDIOMIL 10mg tablets
d7a2.	*LUDIOMIL 25mg tablets
d7a3.	*LUDIOMIL 50mg tablets
d7a4.	*LUDIOMIL 75mg tablets x28CP
d7aw.	MAPROTILINE HYDROCHLORIDE 10mg tablets
d7ax.	MAPROTILINE HYDROCHLORIDE 25mg tablets
d7ay.	MAPROTILINE HYDROCHLORIDE 50mg tablets
d7az.	MAPROTILINE HYDROCHLORIDE 75mg tablets
d7b..	MIANSERIN HYDROCHLORIDE
d7b1.	MIANSERIN 10mg tablets
d7b2.	MIANSERIN 20mg tablets

For peer review only - http://bmjopen.bmj.com/site/about/guidelines.xhtml

CODE	DESCRIPTION
d7b3.	MIANSERIN 30mg tablets
d7b4.	*BOLVIDON 10mg tablets
d7b5.	*BOLVIDON 20mg tablets
d7b6.	*BOLVIDON 30mg tablets
d7b7.	*NORVAL 10mg tablets
d7b8.	*NORVAL 20mg tablets
d7b9.	*NORVAL 30mg tablets
d7c..	NORTRIPTYLINE
d7c1.	*ALLEGRON 10mg tablets
d7c2.	*ALLEGRON 25mg tablets
d7c3.	*AVENTYL 10mg capsules
d7c4.	*AVENTYL 25mg capsules
d7c5.	*AVENTYL 10mg/5mL liquid
d7c6.	NORTRIPTYLINE 10mg tablets
d7c7.	*NORTRIPTYLINE 10mg/5mL liquid
d7c8.	NORTRIPTYLINE 25mg tablets
d7c9.	*NORTRIPTYLINE 10mg capsules
d7cy.	*NORTRIPTYLINE 25mg capsules
d7d..	PROTRIPTYLINE HYDROCHLORIDE
d7d1.	*CONCORDIN 5mg tablets
d7d2.	*CONCORDIN 10mg tablets
d7d3.	PROTRIPTYLINE HYDROCHLORIDE 5mg tablets
d7d4.	PROTRIPTYLINE HYDROCHLORIDE 10mg tablets
d7e..	TRAZODONE HYDROCHLORIDE
d7e1.	MOLIPAXIN 50mg capsules x84CP
d7e2.	MOLIPAXIN 100mg capsules x56CP
d7e3.	*MOLIPAXIN 50mg/5mL liquid
d7e4.	MOLIPAXIN 150mg tablets x28CP
d7e5.	TRAZODONE HYDROCHLORIDE 150mg tablets
d7e6.	MOLIPAXIN CR 150mg m/r tablets x28
d7e7.	TRAZODONE HYDROCHLORIDE 150mg m/r tablets
d7ew.	TRAZODONE HYDROCHLORIDE 100mg capsules
d7ex.	TRAZODONE HYDROCHLORIDE 50mg capsules
d7ez.	TRAZODONE HYDROCHLORIDE 50mg/5mL liquid
d7f..	TRIMIPRAMINE
d7f1.	*SURMONTIL 50mg capsules x28CP
d7f2.	*SURMONTIL 10mg tablets
d7f3.	*SURMONTIL 25mg tablets
d7fx.	TRIMIPRAMINE 50mg capsules
d7fy.	TRIMIPRAMINE 10mg tablets
d7fz.	TRIMIPRAMINE 25mg tablets
d7g..	VILOXAZINE HYDROCHLORIDE
d7g1.	*VIVALAN 50mg tablets
d7gz.	*VILOXAZINE 50mg tablets
d7h..	AMOXAPINE

For peer review only - http://bmjopen.bmj.com/site/about/guidelines.xhtml

CODE	DESCRIPTION
d7h1.	*AMOXAPINE 25mg tablets
d7h2.	*AMOXAPINE 50mg tablets
d7h3.	*AMOXAPINE 100mg tablets
d7h4.	*AMOXAPINE 150mg tablets
d7h5.	*ASENDIS 25mg tablets
d7h6.	*ASENDIS 50mg tablets
d7h7.	*ASENDIS 100mg tablets
d7h8.	*ASENDIS 150mg tablets
d9...	COMPOUND ANTIDEPRESSANT DRUGS
d91..	COMPOUND ANTIDEPRESSANTS A-Z
d911.	*LIMBITROL 5 capsules
d912.	*LIMBITROL 10 capsules
d913.	*MOTIPRESS tablets x28CP
d914.	*MOTIVAL tablets
d915.	*PARSTELIN tablets
d916.	TRIPTAFEN tablets
d917.	*TRIPTAFEN-M tablets
da...	OTHER ANTIDEPRESSANT DRUGS
da1..	FLUPENTIXOL [ANTIDEPRESSANT]
da11.	FLUANXOL 500micrograms tablets
da12.	FLUANXOL 1mg tablets
da1y.	FLUPENTIXOL 500micrograms tablets
da1z.	FLUPENTIXOL 1mg tablets
da2..	TRYPTOPHAN
da21.	*OPTIMAX 500mg tablets
da22.	*OPTIMAX 1g/6g powder
da23.	*OPTIMAX WV 500mg tablets
da24.	*PACITRON 500mg tablets
da2y.	*TRYPTOPHAN 500mg tablets
da2z.	*TRYPTOPHAN 1g/6g powder
da3..	FLUVOXAMINE MALEATE
da31.	FAVERIN 50mg tablets
da32.	FLUVOXAMINE MALEATE 50mg tablets
da33.	FAVERIN 100mg tablets
da34.	FLUVOXAMINE MALEATE 100mg tablets
da4..	FLUOXETINE HYDROCHLORIDE
da41.	FLUOXETINE 20mg capsules
da42.	*PROZAC 20mg capsules x30
da43.	FLUOXETINE 20mg/5mL oral liquid
da44.	PROZAC 20mg/5mL oral liquid
da45.	PROZAC 20mg capsules
da46.	FLUOXETINE 60mg capsules
da47.	*PROZAC 60mg capsules
da48.	*FELICIUM 20mg capsules
da49.	OXACTIN 20mg capsules

For peer review only

CODE	DESCRIPTION
da4A.	RANFLUTIN 20mg capsules
da4B.	PROZIT 20mg/5mL oral solution
da4C.	PROZEP 20mg/5mL oral solution
da5..	SERTRALINE HYDROCHLORIDE
da51.	SERTRALINE 50mg tablets
da52.	SERTRALINE 100mg tablets
da53.	LUSTRAL 50mg tablets
da54.	LUSTRAL 100mg tablets
da6..	PAROXETINE HYDROCHLORIDE
da61.	PAROXETINE 20mg tablets
da62.	SEROXAT 20mg tablets x30
da63.	PAROXETINE 30mg tablets
da64.	SEROXAT 30mg tablets x30
da65.	PAROXETINE 10mg/5mL sugar free liquid
da66.	SEROXAT 10mg/5mL sugar free liquid
da67.	PAROXETINE 10mg tablets
da68.	SEROXAT 10mg tablets
da7..	VENLAFAXINE
da71.	VENLAFAXINE 37.5mg tablets
da72.	VENLAFAXINE 75mg tablets
da73.	*EFEXOR 37.5mg tablets
da74.	*EFEXOR 75mg tablets
da75.	*VENLAFAXINE 50mg tablets
da76.	*EFEXOR 50mg tablets
da77.	VENLAFAXINE 75mg m/r capsules
da78.	EFEXOR XL 75mg m/r capsules
da79.	VENLAFAXINE 150mg m/r capsules
da7a.	VENAXX XL 150mg m/r capsules
da7A.	EFEXOR XL 150mg m/r capsules
da7b.	VAXALIN XL 75mg m/r capsules
da7B.	RODOMEL XL 75mg m/r capsules
da7c.	VAXALIN XL 150mg m/r capsules
da7C.	RODOMEL XL 150mg m/r capsules
da7d.	ALVENTA XL 75mg m/r capsules
da7D.	WINFEX XL 75mg m/r capsules
da7e.	ALVENTA XL 150mg m/r capsules
da7E.	WINFEX XL 150mg m/r capsules
da7f.	RANFAXINE XL 150mg m/r capsules
da7F.	TRIXAT XL 75mg m/r capsules
da7g.	RANFAXINE XL 75mg m/r capsules
da7G.	TRIXAT XL 150mg m/r capsules
da7h.	BONILUX XL 75mg m/r capsules
da7H.	VIEPAX XL 75mg m/r tablets
da7i.	BONILUX XL 150mg m/r capsules
da7I.	VENLAFAXINE 75mg m/r tablets

For peer review only

CODE	DESCRIPTION
da7j.	TONPULAR XL 75mg m/r capsules
da7J.	VIEPAX XL 150mg m/r tablets
da7k.	TONPULAR XL 150mg m/r capsules
da7K.	VENLAFAXINE 150mg m/r tablets
da7l.	FORAVEN XL 75mg m/r capsules
da7L.	*TARDCAPS XL 75mg m/r capsules
da7m.	FORAVEN XL 150mg m/r capsules
da7M.	*TARDCAPS XL 150mg m/r capsule
da7n.	DEPEFEX XL 75mg m/r capsules
da7N.	VIEPAX 37.5mg tablets
da7o.	DEPEFEX XL 150mg m/r capsules
da7O.	VIEPAX 75mg tablets
da7p.	VENLALIC XL 37.5mg m/r tablets
da7P.	VENSIR XL 75mg m/r capsules
da7q.	VENLAFAXINE 37.5mg m/r tablets
da7Q.	VENSIR XL 150mg m/r capsules
da7R.	TIFAXIN XL 75mg m/r capsules
da7S.	TIFAXIN XL 150mg m/r capsules
da7T.	VEXARIN XL 75mg m/r capsules
da7U.	VEXARIN XL 150mg m/r capsules
da7V.	VENLALIC XL 75mg m/r tablets
da7W.	VENLALIC XL 150mg m/r tablets
da7X.	VENLALIC XL 225mg m/r tablets
da7Y.	VENLAFAXINE 225mg m/r tablets
da7Z.	VENAXX XL 75mg m/r capsules
da8..	NEFAZODONE
da81.	NEFAZODONE HYDROCHLORIDE 100mg tablets
da82.	NEFAZODONE HYDROCHLORIDE 200mg tablets
da83.	*DUTONIN 100mg tablets
da84.	*DUTONIN 200mg tablets
da85.	NEFAZODONE HCL 50mg+100mg+200mg initiation tablets pack
da86.	DUTONIN 50mg+100mg+200mg treatment initiation tablets pack
da9..	CITALOPRAM
da91.	CITALOPRAM 20mg tablets
da92.	CIPRAMIL 20mg tablets
da93.	CITALOPRAM 10mg tablets
da94.	*CIPRAMIL 10mg tablets
da95.	CITALOPRAM 40mg tablets
da96.	*CIPRAMIL 40mg tablets
da97.	CIPRAMIL 40mg/mL oral drops 15mL
da98.	*PAXORAN 10mg tablets
da99.	*PAXORAN 20mg tablets
da9A.	*PAXORAN 40mg tablets
da9z.	CITALOPRAM 40mg/mL oral drops
daA..	REBOXETINE

For peer review only - http://bmjopen.bmj.com/site/about/guidelines.xhtml

For peer review only

CODE	DESCRIPTION
daA1.	REBOXETINE 4mg tablets
daA2.	EDRONAX 4mg tablets
daB..	MIRTAZAPINE
daB1.	MIRTAZAPINE 30mg tablets
daB2.	*ZISPIN 30mg tablets
daB3.	MIRTAZAPINE 30mg oro-dispersible tablets
daB4.	ZISPIN SOLTAB 30mg oro-dispersible tablets
daB5.	MIRTAZAPINE 15mg oro-dispersible tablets
daB6.	ZISPIN SOLTAB 15mg oro-dispersible tablets
daB7.	MIRTAZAPINE 45mg oro-dispersible tablets
daB8.	ZISPIN SOLTAB 45mg oro-dispersible tablets
daBy.	MIRTAZAPINE 45mg tablets
daBz.	MIRTAZAPINE 15mg tablets
daC..	ESCITALOPRAM
daC1.	ESCITALOPRAM 10mg tablets
daC2.	CIPRALEX 10mg tablets
daC3.	ESCITALOPRAM 20mg tablets
daC4.	CIPRALEX 20mg tablets
daC5.	ESCITALOPRAM 5mg tablets
daC6.	CIPRALEX 5mg tablets
daC7.	ESCITALOPRAM 10mg/mL oral drops
daC8.	*CIPRALEX 10mg/mL oral drops
daC9.	CIPRALEX 20mg/mL oral drops
daCA.	ESCITALOPRAM 20mg/mL oral drops
daD..	AGOMELATINE
daD1.	VALDOXAN 25mg tablets
daD2.	AGOMELATINE 25mg tablets
E1...	Non-organic psychoses
E10..	Schizophrenic disorders
E100.	Simple schizophrenia
E1000	Unspecified schizophrenia
E1001	Subchronic schizophrenia
E1002	Chronic schizophrenic
E1003	Acute exacerbation of subchronic schizophrenia
E1004	Acute exacerbation of chronic schizophrenia
E1005	Schizophrenia in remission
E100z	Simple schizophrenia NOS
E101.	Hebephrenic schizophrenia
E1010	Unspecified hebephrenic schizophrenia
E1011	Subchronic hebephrenic schizophrenia
E1012	Chronic hebephrenic schizophrenia
E1013	Acute exacerbation of subchronic hebephrenic schizophrenia
E1014	Acute exacerbation of chronic hebephrenic schizophrenia
E1015	Hebephrenic schizophrenia in remission
E101z	Hebephrenic schizophrenia NOS

For peer review only - http://bmjopen.bmj.com/site/about/guidelines.xhtml

CODE	DESCRIPTION
E102.	Catatonic schizophrenia
E1020	Unspecified catatonic schizophrenia
E1021	Subchronic catatonic schizophrenia
E1022	Chronic catatonic schizophrenia
E1023	Acute exacerbation of subchronic catatonic schizophrenia
E1024	Acute exacerbation of chronic catatonic schizophrenia
E1025	Catatonic schizophrenia in remission
E102z	Catatonic schizophrenia NOS
E103.	Paranoid schizophrenia
E1030	Unspecified paranoid schizophrenia
E1031	Subchronic paranoid schizophrenia
E1032	Chronic paranoid schizophrenia
E1033	Acute exacerbation of subchronic paranoid schizophrenia
E1034	Acute exacerbation of chronic paranoid schizophrenia
E1035	Paranoid schizophrenia in remission
E103z	Paranoid schizophrenia NOS
E104.	Acute schizophrenic episode
E105.	Latent schizophrenia
E1050	Unspecified latent schizophrenia
E1051	Subchronic latent schizophrenia
E1052	Chronic latent schizophrenia
E1053	Acute exacerbation of subchronic latent schizophrenia
E1054	Acute exacerbation of chronic latent schizophrenia
E1055	Latent schizophrenia in remission
E105z	Latent schizophrenia NOS
E106.	Residual schizophrenia
E107.	Schizo-affective schizophrenia
E1070	Unspecified schizo-affective schizophrenia
E1071	Subchronic schizo-affective schizophrenia
E1072	Chronic schizo-affective schizophrenia
E1073	Acute exacerbation subchronic schizo-affective schizophrenia
E1074	Acute exacerbation of chronic schizo-affective schizophrenia
E1075	Schizo-affective schizophrenia in remission
E107z	Schizo-affective schizophrenia NOS
E10y.	Other schizophrenia
E10y0	Atypical schizophrenia
E10y1	Coenesthopathic schizophrenia
E10yz	Other schizophrenia NOS
E10z.	Schizophrenia NOS
E11..	Affective psychoses
E110.	Manic disorder, single episode
E1100	Single manic episode, unspecified
E1101	Single manic episode, mild
E1102	Single manic episode, moderate
E1103	Single manic episode, severe without mention of psychosis

For peer review only - http://bmjopen.bmj.com/site/about/guidelines.xhtml

CODE	DESCRIPTION
E1104	Single manic episode, severe, with psychosis
E1105	Single manic episode in partial or unspecified remission
E1106	Single manic episode in full remission
E110z	Manic disorder, single episode NOS
E111.	Recurrent manic episodes
E1110	Recurrent manic episodes, unspecified
E1111	Recurrent manic episodes, mild
E1112	Recurrent manic episodes, moderate
E1113	Recurrent manic episodes, severe without mention psychosis
E1114	Recurrent manic episodes, severe, with psychosis
E1115	Recurrent manic episodes, partial or unspecified remission
E1116	Recurrent manic episodes, in full remission
E111z	Recurrent manic episode NOS
E112.	Single major depressive episode
E1120	Single major depressive episode, unspecified
E1121	Single major depressive episode, mild
E1122	Single major depressive episode, moderate
E1123	Single major depressive episode, severe, without psychosis
E1124	Single major depressive episode, severe, with psychosis
E1125	Single major depressive episode, partial or unspec remission
E1126	Single major depressive episode, in full remission
E112z	Single major depressive episode NOS
E113.	Recurrent major depressive episode
E1130	Recurrent major depressive episodes, unspecified
E1131	Recurrent major depressive episodes, mild
E1132	Recurrent major depressive episodes, moderate
E1133	Recurrent major depressive episodes, severe, no psychosis
E1134	Recurrent major depressive episodes, severe, with psychosis
E1135	Recurrent major depressive episodes,partial/unspec remission
E1136	Recurrent major depressive episodes, in full remission
E1137	Recurrent depression
E113z	Recurrent major depressive episode NOS
E114.	Bipolar affective disorder, currently manic
E1140	Bipolar affective disorder, currently manic, unspecified
E1141	Bipolar affective disorder, currently manic, mild
E1142	Bipolar affective disorder, currently manic, moderate
E1143	Bipolar affect disord, currently manic, severe, no psychosis
E1144	Bipolar affect disord, currently manic,severe with psychosis
E1145	Bipolar affect disord,currently manic, part/unspec remission
E1146	Bipolar affective disorder, currently manic, full remission
E114z	Bipolar affective disorder, currently manic, NOS
E115.	Bipolar affective disorder, currently depressed
E1150	Bipolar affective disorder, currently depressed, unspecified
E1151	Bipolar affective disorder, currently depressed, mild
E1152	Bipolar affective disorder, currently depressed, moderate

For peer review only - http://bmjopen.bmj.com/site/about/guidelines.xhtml

CODE	DESCRIPTION
E1153	Bipolar affect disord, now depressed, severe, no psychosis
E1154	Bipolar affect disord, now depressed, severe with psychosis
E1155	Bipolar affect disord, now depressed, part/unspec remission
E1156	Bipolar affective disorder, now depressed, in full remission
E115z	Bipolar affective disorder, currently depressed, NOS
E116.	Mixed bipolar affective disorder
E1160	Mixed bipolar affective disorder, unspecified
E1161	Mixed bipolar affective disorder, mild
E1162	Mixed bipolar affective disorder, moderate
E1163	Mixed bipolar affective disorder, severe, without psychosis
E1164	Mixed bipolar affective disorder, severe, with psychosis
E1165	Mixed bipolar affective disorder, partial/unspec remission
E1166	Mixed bipolar affective disorder, in full remission
E116z	Mixed bipolar affective disorder, NOS
E117.	Unspecified bipolar affective disorder
E1170	Unspecified bipolar affective disorder, unspecified
E1171	Unspecified bipolar affective disorder, mild
E1172	Unspecified bipolar affective disorder, moderate
E1173	Unspecified bipolar affective disorder, severe, no psychosis
E1174	Unspecified bipolar affective disorder,severe with psychosis
E1175	Unspecified bipolar affect disord, partial/unspec remission
E1176	Unspecified bipolar affective disorder, in full remission
E117z	Unspecified bipolar affective disorder, NOS
E118.	Seasonal affective disorder
E11y.	Other and unspecified manic-depressive psychoses
E11y0	Unspecified manic-depressive psychoses
E11y1	Atypical manic disorder
E11y2	Atypical depressive disorder
E11y3	Other mixed manic-depressive psychoses
E11yz	Other and unspecified manic-depressive psychoses NOS
E11z.	Other and unspecified affective psychoses
E11z0	Unspecified affective psychoses NOS
E11zz	Other affective psychosis NOS
E12..	Paranoid states
E120.	Simple paranoid state
E121.	Chronic paranoid psychosis
E122.	Paraphrenia
E123.	Shared paranoid disorder
E12y.	Other paranoid states
E12y0	Paranoia querulans
E12yz	Other paranoid states NOS
E12z.	Paranoid psychosis NOS
E13..	Other nonorganic psychoses
E130.	Reactive depressive psychosis
E131.	Acute hysterical psychosis

For peer review only - http://bmjopen.bmj.com/site/about/guidelines.xhtml

CODE	DESCRIPTION
E132.	Reactive confusion
E133.	Acute paranoid reaction
E134.	Psychogenic paranoid psychosis
E135.	Agitated depression
E13y.	Other reactive psychoses
E13y0	Psychogenic stupor
E13y1	Brief reactive psychosis
E13yz	Other reactive psychoses NOS
E13z.	Nonorganic psychosis NOS
E1y..	Other specified non-organic psychoses
E1z..	Non-organic psychosis NOS
E200.	Anxiety states
E2000	Anxiety state unspecified
E2001	Panic disorder
E2002	Generalised anxiety disorder
E2003	Anxiety with depression
E2004	Chronic anxiety
E2005	Recurrent anxiety
E200z	Anxiety state NOS
E204.	Neurotic depression reactive type
E212.	Schizoid personality disorder
E2120	Unspecified schizoid personality disorder
E2121	Introverted personality
E2122	Schizotypal personality
E212z	Schizoid personality disorder NOS
E258.	Nondependent antidepressant type drug abuse
E2580	Nondependent antidepressant type drug abuse, unspecified
E2581	Nondependent antidepressant type drug abuse, continuous
E2582	Nondependent antidepressant type drug abuse, episodic
E2583	Nondependent antidepressant type drug abuse in remission
E258z	Nondependent antidepressant type drug abuse NOS
E291.	Prolonged depressive reaction
E2B..	Depressive disorder NEC
E2B0.	Postviral depression
E2B1.	Chronic depression
Eu...	[X]Mental and behavioural disorders
Eu2..	[X]Schizophrenia, schizotypal and delusional disorders
Eu20.	[X]Schizophrenia
Eu200	[X]Paranoid schizophrenia
Eu201	[X]Hebephrenic schizophrenia
Eu202	[X]Catatonic schizophrenia
Eu203	[X]Undifferentiated schizophrenia
Eu204	[X]Post-schizophrenic depression
Eu205	[X]Residual schizophrenia
Eu206	[X]Simple schizophrenia

For peer review only

For peer review only - http://bmjopen.bmj.com/site/about/guidelines.xhtml

CODE	DESCRIPTION
Eu20y	[X]Other schizophrenia
Eu20z	[X]Schizophrenia, unspecified
Eu21.	[X]Schizotypal disorder
Eu22.	[X]Persistent delusional disorders
Eu220	[X]Delusional disorder
Eu221	[X]Delusional misidentification syndrome
Eu222	[X]Cotard syndrome
Eu223	[X]Paranoid state in remission
Eu22y	[X]Other persistent delusional disorders
Eu22z	[X]Persistent delusional disorder, unspecified
Eu23.	[X]Acute and transient psychotic disorders
Eu230	[X]Acute polymorphic psychot disord without symp of schizoph
Eu231	[X]Acute polymorphic psychot disord with symp of schizophren
Eu232	[X]Acute schizophrenia-like psychotic disorder
Eu233	[X]Other acute predominantly delusional psychotic disorders
Eu23y	[X]Other acute and transient psychotic disorders
Eu23z	[X]Acute and transient psychotic disorder, unspecified
Eu24.	[X]Induced delusional disorder
Eu25.	[X]Schizoaffective disorders
Eu250	[X]Schizoaffective disorder, manic type
Eu251	[X]Schizoaffective disorder, depressive type
Eu252	[X]Schizoaffective disorder, mixed type
Eu25y	[X]Other schizoaffective disorders
Eu25z	[X]Schizoaffective disorder, unspecified
Eu26.	[X]Nonorganic psychosis in remission
Eu2y.	[X]Other nonorganic psychotic disorders
Eu2z.	[X]Unspecified nonorganic psychosis
Eu3..	[X]Mood - affective disorders
Eu30.	[X]Manic episode
Eu300	[X]Hypomania
Eu301	[X]Mania without psychotic symptoms
Eu302	[X]Mania with psychotic symptoms
Eu30y	[X]Other manic episodes
Eu30z	[X]Manic episode, unspecified
Eu31.	[X]Bipolar affective disorder
Eu310	[X]Bipolar affective disorder, current episode hypomanic
Eu311	[X]Bipolar affect disorder cur epi manic wout psychotic symp
Eu312	[X]Bipolar affect disorder cur epi manic with psychotic symp
Eu313	[X]Bipolar affect disorder cur epi mild or moderate depressn
Eu314	[X]Bipol aff disord, curr epis sev depress, no psychot symp
Eu315	[X]Bipolar affect dis cur epi severe depres with psyc symp
Eu316	[X]Bipolar affective disorder, current episode mixed
Eu317	[X]Bipolar affective disorder, currently in remission
Eu318	[X]Bipolar affective disorder type I
Eu319	[X]Bipolar affective disorder type II

For peer review only - http://bmjopen.bmj.com/site/about/guidelines.xhtml

CODE	DESCRIPTION
Eu31y	[X]Other bipolar affective disorders
Eu31z	[X]Bipolar affective disorder, unspecified
Eu32.	[X]Depressive episode
Eu320	[X]Mild depressive episode
Eu321	[X]Moderate depressive episode
Eu322	[X]Severe depressive episode without psychotic symptoms
Eu323	[X]Severe depressive episode with psychotic symptoms
Eu324	[X]Mild depression
Eu328	[X]Major depression, severe with psychotic symptoms
Eu329	[X]Single major depr ep, severe with psych, psych in remiss
Eu32A	[X]Recurr major depr ep, severe with psych, psych in remiss
Eu32y	[X]Other depressive episodes
Eu32z	[X]Depressive episode, unspecified
Eu33.	[X]Recurrent depressive disorder
Eu330	[X]Recurrent depressive disorder, current episode mild
Eu331	[X]Recurrent depressive disorder, current episode moderate
Eu332	[X]Recurr depress disorder cur epi severe without psyc sympt
Eu333	[X]Recurrent depress disorder cur epi severe with psyc symp
Eu334	[X]Recurrent depressive disorder, currently in remission
Eu33y	[X]Other recurrent depressive disorders
Eu33z	[X]Recurrent depressive disorder, unspecified
Eu34.	[X]Persistent mood affective disorders
Eu341	[X]Dysthymia
Eu34y	[X]Other persistent mood affective disorders
Eu34z	[X]Persistent mood affective disorder, unspecified
Eu3y.	[X]Other mood affective disorders
Eu3y0	[X]Other single mood affective disorders
Eu3y1	[X]Other recurrent mood affective disorders
Eu3yy	[X]Other specified mood affective disorders
Eu3z.	[X]Unspecified mood affective disorder
Eu41.	[X]Other anxiety disorders
Eu410	[X]Panic disorder [episodic paroxysmal anxiety]
Eu411	[X]Generalized anxiety disorder
Eu412	[X]Mixed anxiety and depressive disorder
Eu413	[X]Other mixed anxiety disorders
Eu41y	[X]Other specified anxiety disorders
Eu41z	[X]Anxiety disorder, unspecified
Eu601	[X]Schizoid personality disorder
R2y2.	[D]Nervousness
SL90.	Antidepressant poisoning
SL90z	Anti-depressant poisoning NOS
SyuFK	[X]Poisoning by other and unspecified antidepressants
T840.	Accidental poisoning by antidepressants
T840z	Accidental poisoning by antidepressants NOS
TJ90.	Adverse reaction to antidepressants

For peer review only - http://bmjopen.bmj.com/site/about/guidelines.xhtml

CODE	DESCRIPTION
TJ90z	Adverse reaction to antidepressants NOS
U6090	[X]Tricyc/tetracyc antidepres caus advers eff therapeut use
U6091	[X]Monoamine oxid inhib antidep caus advers eff therap use
U6092	[X]Other unspec antidepres caus advers effect in therap use
ZV110	[V]Personal history of schizophrenia
ZV111	[V]Personal history of affective disorder

Fit notes (Read)

CODE	DESCRIPTION
13JJ1	Benefit agncy reprt unfit for work but fit note no lngr need
9D1..	MED3 - doctor's statement
9D11.	MED3 issued to patient
9D12.	MED3 duplicate issued
9D14.	MED3 issued - back to work
9D15.	eMED3 (2010) new statement issued, not fit for work
9D19.	MED3 (2010) issued by hand, not fit for work
9D1C.	MED3 (2010) issued to patient
9D1Z.	MED3 - NOS
9D5..	Private sickness certificate
9DC..	SC1 - self certificate admin.
9DC1.	SC1 self-certificate advised
9DC2.	SC1 self certificate given
9DCZ.	SC1 self-certification NOS
9DL..	SC2 - self-certificate administration
9DL0.	SC2 self-certificate advised
9DL1.	SC2 self certificate given
9K8..	Sickness certificate
ZV680	[V]Issue of medical certificate

Hospital admissions for Non-COVID-19 respiratory infections (ICD-10)

CODE	DESCRIPTION
A37	Whooping cough
A370	Whooping cough due to Bordetella pertussis
A371	Whooping cough due to Bordetella parapertussis
A378	Whooping cough due to other Bordetella species
A379	Whooping cough, unspecified
B012	Varicella pneumonia
B052	Measles complicated by pneumonia
B250	Cytomegaloviral pneumonitis
J10	Influenza due to identified seasonal influenza virus
J100	Influenza with pneumonia, seasonal influenza virus identified
J101	Influenza with other respiratory manifestations, seasonal influenza virus identified

For peer review only - http://bmjopen.bmj.com/site/about/guidelines.xhtml

CODE	DESCRIPTION
J108	Influenza with other manifestations, seasonal influenza virus identified
J11	Influenza, virus not identified
J110	Influenza with pneumonia, virus not identified
J111	Influenza with other respiratory manifestations, virus not identified
J118	Influenza with other manifestations, virus not identified
J12	Viral pneumonia, not elsewhere classified
J120	Adenoviral pneumonia
J121	Respiratory syncytial virus pneumonia
J122	Parainfluenza virus pneumonia
J123	Human metapneumovirus pneumonia
J128	Other viral pneumonia
J129	Viral pneumonia, unspecified
J15	Bacterial pneumonia, not elsewhere classified
J150	Pneumonia due to Klebsiella pneumoniae
J151	Pneumonia due to Pseudomonas
J152	Pneumonia due to staphylococcus
J153	Pneumonia due to streptococcus, group B
J154	Pneumonia due to other streptococci
J155	Pneumonia due to Escherichia coli
J156	Pneumonia due to other Gram-negative bacteria
J157	Pneumonia due to Mycoplasma pneumoniae
J158	Other bacterial pneumonia
J159	Bacterial pneumonia, unspecified
J16	Pneumonia due to other infectious organisms, not elsewhere classified
J168	Pneumonia due to other specified infectious organisms
J17	Pneumonia in diseases classified elsewhere
J170	Pneumonia in bacterial diseases classified elsewhere
J171	Pneumonia in viral diseases classified elsewhere
J172	Pneumonia in mycoses
J173	Pneumonia in parasitic diseases
J178	Pneumonia in other diseases classified elsewhere
J18	Pneumonia, organism unspecified
J180	Bronchopneumonia, unspecified
J181	Lobar pneumonia, unspecified
J182	Hypostatic pneumonia, unspecified
J188	Other pneumonia, organism unspecified
J189	Pneumonia, unspecified
J20	Acute bronchitis
J200	Acute bronchitis due to Mycoplasma pneumoniae
J201	Acute bronchitis due to Haemophilus influenzae
J202	Acute bronchitis due to streptococcus
J203	Acute bronchitis due to coxsackievirus
J204	Acute bronchitis due to parainfluenza virus
J205	Acute bronchitis due to respiratory syncytial virus
J206	Acute bronchitis due to rhinovirus

For peer review only - http://bmjopen.bmj.com/site/about/guidelines.xhtml

CODE	DESCRIPTION
J207	Acute bronchitis due to echovirus
J208	Acute bronchitis due to other specified organisms
J209	Acute bronchitis, unspecified
J21	Acute bronchiolitis
J210	Acute bronchiolitis due to respiratory syncytial virus
J211	Acute bronchiolitis due to human metapneumovirus
J218	Acute bronchiolitis due to other specified organisms
J219	Acute bronchiolitis, unspecified
J41	Simple and mucopurulent chronic bronchitis
J410	Simple chronic bronchitis
J411	Mucopurulent chronic bronchitis
J418	Mixed simple and mucopurulent chronic bronchitis
J43	Emphysema
J430	MacLeod syndrome
J431	Panlobular emphysema
J432	Centrilobular emphysema
J438	Other emphysema
J439	Emphysema, unspecified
J440	Chronic obstructive pulmonary disease with acute lower respiratory infection
J441	Chronic obstructive pulmonary disease with acute exacerbation, unspecified
J448	Other specified chronic obstructive pulmonary disease
J449	Chronic obstructive pulmonary disease, unspecified
J45	Asthma
J450	Predominantly allergic asthma
J451	Nonallergic asthma
J458	Mixed asthma
J459	Asthma, unspecified
J851	Abscess of lung with pneumonia
J86	Pyothorax
J860	Pyothorax with fistula
J869	Pyothorax without fistula
J982	Interstitial emphysema
J983	Compensatory emphysema
U04	Severe acute respiratory syndrome [SARS]

For peer review only

Supplementary material 3: The RECORD statement – checklist of items, extended from the STROBE statement, that should be reported in observational studies using routinely collected health data.

	Item No.	STROBE items	Section/Location in manuscript where items are reported	RECORD items	Section/Location in manuscript where items are reported
**Title and abstract**					
	1	(a) Indicate the study's design with a commonly used term in the title or the abstract<br><br>(b) Provide in the abstract an informative and balanced summary of what was done and what was found	Title<br><br>Title	RECORD 1.1: The type of data used should be specified in the title or abstract. When possible, the name of the databases used should be included.<br><br>RECORD 1.2: If applicable, the geographic region and timeframe within which the study took place should be reported in the title or abstract.<br><br>RECORD 1.3: If linkage between databases was conducted for the study, this should be clearly stated in the title or abstract.	Title<br><br>Title<br><br>NA
**Introduction**					
Background rationale	2	Explain the scientific background and rationale for the investigation being reported	Introduction		
Objectives	3	State specific objectives, including any prespecified hypotheses	Introduction		
**Methods**					
Study Design	4	Present key elements of study design early in the paper	Study design and data sources		
Setting	5	Describe the setting, locations, and relevant dates, including periods of recruitment, exposure, follow-up, and data collection	Materials and methods		
Participants	6	*(a) Cohort study* - Give the eligibility criteria, and the sources and methods of selection of participants. Describe methods of follow-up<br>*Case-control study* - Give the eligibility criteria, and the sources and methods of case	Population	RECORD 6.1: The methods of study population selection (such as codes or algorithms used to identify subjects) should be listed in detail. If this is not possible, an explanation should be provided.	NA

For peer review only - http://bmjopen.bmj.com/site/about/guidelines.xhtml

	Item No.	STROBE items	Section/Location in manuscript where items are reported	RECORD items	Section/Location in manuscript where items are reported
		ascertainment and control selection. Give the rationale for the choice of cases and controls<br>*Cross-sectional study* - Give the eligibility criteria, and the sources and methods of selection of participants<br><br>*(b) Cohort study* - For matched studies, give matching criteria and number of exposed and unexposed<br>*Case-control study* - For matched studies, give matching criteria and the number of controls per case		RECORD 6.2: Any validation studies of the codes or algorithms used to select the population should be referenced. If validation was conducted for this study and not published elsewhere, detailed methods and results should be provided.<br><br>RECORD 6.3: If the study involved linkage of databases, consider use of a flow diagram or other graphical display to demonstrate the data linkage process, including the number of individuals with linked data at each stage.	NA<br><br>NA
Variables	7	Clearly define all outcomes, exposures, predictors, potential confounders, and effect modifiers. Give diagnostic criteria, if applicable.	Primary and secondary outcomes	RECORD 7.1: A complete list of codes and algorithms used to classify exposures, outcomes, confounders, and effect modifiers should be provided. If these cannot be reported, an explanation should be provided.	Supplementary material
Data sources/ measurement	8	For each variable of interest, give sources of data and details of methods of assessment (measurement).<br>Describe comparability of assessment methods if there is more than one group	Primary and secondary outcomes and supplementary material 1		
Bias	9	Describe any efforts to address potential sources of bias	Discussion – strengths and weaknesses		
Study size	10	Explain how the study size was arrived at	Protocol paper		
Quantitative variables	11	Explain how quantitative variables were handled in the analyses. If applicable, describe which groupings were chosen, and why	Analysis		
Statistical methods	12	(a) Describe all statistical methods, including those used to control for confounding	Analysis		

For peer review only - http://bmjopen.bmj.com/site/about/guidelines.xhtml

	Item No.	STROBE items	Section/Location in manuscript where items are reported	RECORD items	Section/Location in manuscript where items are reported
		(b) Describe any methods used to examine subgroups and interactions<br>(c) Explain how missing data were addressed<br>(d) *Cohort study* - If applicable, explain how loss to follow-up was addressed<br>*Case-control study* - If applicable, explain how matching of cases and controls was addressed<br>*Cross-sectional study* - If applicable, describe analytical methods taking account of sampling strategy<br>(e) Describe any sensitivity analyses			
Data access and cleaning methods		..		RECORD 12.1: Authors should describe the extent to which the investigators had access to the database population used to create the study population.<br><br>RECORD 12.2: Authors should provide information on the data cleaning methods used in the study.	Analysis<br><br>Analysis
Linkage		..		RECORD 12.3: State whether the study included person-level, institutional-level, or other data linkage across two or more databases. The methods of linkage and methods of linkage quality evaluation should be provided.	Study design and data sources
**Results**					
Participants	13	(a) Report the numbers of individuals at each stage of the study (*e.g.*, numbers potentially eligible, examined for eligibility, confirmed eligible, included in the study, completing follow-up, and analysed)<br>(b) Give reasons for non-participation at each stage.<br>(c) Consider use of a flow diagram	Results: Study population	RECORD 13.1: Describe in detail the selection of the persons included in the study (*i.e.,* study population selection) including filtering based on data quality, data availability and linkage. The selection of included persons can be described in the text and/or by means of the study flow diagram.	Results: Study population

For peer review only - http://bmjopen.bmj.com/site/about/guidelines.xhtml

	Item No.	STROBE items	Section/Location in manuscript where items are reported	RECORD items	Section/Location in manuscript where items are reported
Descriptive data	14	(a) Give characteristics of study participants (*e.g.*, demographic, clinical, social) and information on exposures and potential confounders<br>(b) Indicate the number of participants with missing data for each variable of interest<br>(c) *Cohort study* - summarise follow-up time (*e.g.*, average and total amount)	Results: Study population, table 1		
Outcome data	15	*Cohort study* - Report numbers of outcome events or summary measures over time<br>*Case-control study* - Report numbers in each exposure category, or summary measures of exposure<br>*Cross-sectional study* - Report numbers of outcome events or summary measures	Results, table 2		
Main results	16	(a) Give unadjusted estimates and, if applicable, confounder-adjusted estimates and their precision (e.g., 95% confidence interval). Make clear which confounders were adjusted for and why they were included<br>(b) Report category boundaries when continuous variables were categorized<br>(c) If relevant, consider translating estimates of relative risk into absolute risk for a meaningful time period	Results, table 1 and 2, supplementary material 4-5		
Other analyses	17	Report other analyses done—e.g., analyses of subgroups and interactions, and sensitivity analyses	Results, supplementary material 4-5		
**Discussion**					
Key results	18	Summarise key results with reference to study objectives	Discussion-Principal findings		

For peer review only - http://bmjopen.bmj.com/site/about/guidelines.xhtml

	Item No.	STROBE items	Section/Location in manuscript where items are reported	RECORD items	Section/Location in manuscript where items are reported
Limitations	19	Discuss limitations of the study, taking into account sources of potential bias or imprecision. Discuss both direction and magnitude of any potential bias	Discussion- Strengths and weaknesses	RECORD 19.1: Discuss the implications of using data that were not created or collected to answer the specific research question(s). Include discussion of misclassification bias, unmeasured confounding, missing data, and changing eligibility over time, as they pertain to the study being reported.	Page 16
Interpretation	20	Give a cautious overall interpretation of results considering objectives, limitations, multiplicity of analyses, results from similar studies, and other relevant evidence	Discussion- strengths and weaknesses		
Generalisability	21	Discuss the generalisability (external validity) of the study results	Discussion - findings in context		
**Other Information**					
Funding	22	Give the source of funding and the role of the funders for the present study and, if applicable, for the original study on which the present article is based	Funding		
Accessibility of protocol, raw data, and programming code		..		RECORD 22.1: Authors should provide information on how to access any supplemental information such as the study protocol, raw data, or programming code.	Data sharing statement, protocol, Supplementary material 2

For peer review only - http://bmjopen.bmj.com/site/about/guidelines.xhtml

Supplementary material 4: Hospital admissions for respiratory infections not necessarily recorded as COVID-19 for domiciliary care workers from March 2016 to October 2021.

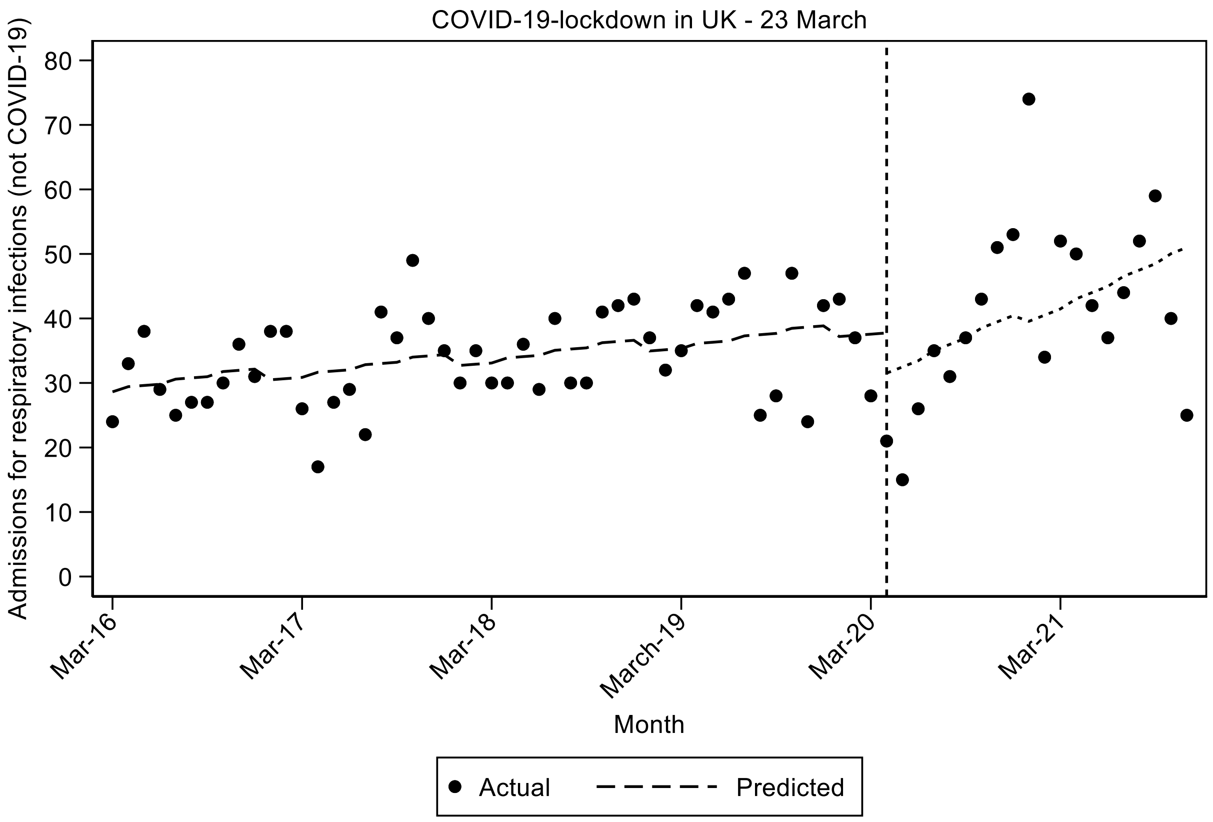

Supplementary material 5: Characteristics of domiciliary care workers receiving at least one fit note in the COVID-19 pandemic between 1st March 2020 and 31st November 2021.

**Characteristics**	**N**	**%**
**Source: SCW[a]**		
Sex		
Female	2,004	15.1
Male	293	11.9
Age (at registration)		
Under 35 years	572	12.9
35 to <45 years	380	13.5
45 to <55 years	625	15.8
55 years and over	720	15.9
Ethnicity		
White	2,220	14.8
Black	11	7.8
Mixed	*	*
Asian	14	13.0

For peer review only - http://bmjopen.bmj.com/site/about/guidelines.xhtml

**Characteristics**	**N**	**%**
Other	*	*
Mixed/Other	18	9.3
Not recorded	34	12.6
** numbers suppressed as <10. Mixed and Other categories are combined.*		
Lives with DCW		
No	2,168	14.7
Yes	129	13.2
Have disability		
No	2,089	14.8
Yes	23	12.2
Missing/Prefer not to answer	185	13.3
Qualifications as recorded at registration		
Competence confirmed	255	12.9
Induction Framework	460	15.4
Qualification Level 2	1,054	15.2
Qualification Level 3	470	14.1
Qualification Level 4+	48	10.9
Not recorded	10	23.3
Employment sector as recorded at registration		
Private	828	12.6
Third Sector	562	13.2
Local Authority - Social Services	678	20.5
Recruitment Agency/ Employment Agency	23	13.4
Health/Other		
Not recorded	206	14.8
Smoking status		
Non-smoker	917	14.5
Ex-smoker	601	12.0
Current smoker	779	17.8
Body Mass Index		
Underweight (<18.5)	37	14.5
Healthy weight (18.5-24.9)	466	14.7
Overweight (25-29.9)	576	15.8
Obese (≥30)	993	18.7
Not recorded	225	6.8
Any comorbidity[d]		
No	1,027	11.5
Yes	1,270	18.7
On COVID-19 shielding list		
No	2,175	14.4
Yes	122	19.2
Resident health board		
Aneurin Bevan	319	11.3
Betsi Cadwaladr	854	23.2
Cardiff and Vale	219	12.2
Cwm Taf Morgannwg	365	13.5

For peer review only

Characteristics	N	%
Hywel Dda	225	11.5
Powys	83	12.5
Swansea Bay	232	11.1
WIMD 2019 quintile		
1 (most deprived)	636	15.3
2	547	13.8
3	478	14.5
4	390	15.3
5 (most affluent)	246	14.2
Rurality classification		
Rural	718	14.6
Urban	1,579	14.6

a between 1st March 2020 to 31st November 2021; b data source = Social Care Wales (DSCW) data and include data that is self-reported by DCWs at 1st April. If missing, then other sources (ONS 2011 Census Wales (CENW), Wales Longitudinal General Practice (WLGP), Patient Episode Dataset for Wales (PEDW), Emergency Department Dataset (EDDS)) were used to impute (at index date=1st March 2020); c data source = WLGP; d data source=PEDW; e most recent occurring between 1st January 2000 and 1st March 2020; f based on residential address at 1st March 2020; g data source =Welsh Demographic Service Dataset (WDSD).

For peer review only

For peer review only

For peer review only - http://bmjopen.bmj.com/site/about/guidelines.xhtml

For peer review only

For peer review only - http://bmjopen.bmj.com/site/about/guidelines.xhtml

Supplementary material 6: Demographics of DCWs across the UK

Nation	Date published	Definition	Denom.	Age	Gender	Ethnicity
Wales[1]	April 2021	DCWs	22,131	44 years (mean)	84% F<br>16% M	97% White British
OSCAR sample	April 2020	DCWs	15,725	44 years (mean)	84% F<br>16% M	96% White British
England[2]	October 2021	DCWs	590,000	44 years (mean)	84% F<br>16% M	na
Scotland[3]	December 2020	Housing support/ care at home	74,870	46 years (median)	77% F<br>17 %M<br>6% unknown<br><br>84% F<br>16% M<br>(excluding unknowns)	98% White<br>2% Asian/Black
Northern Ireland[4]	October 2020	DCWs	16,303	20-29 years (mode)	91% F<br>8% M<br>1% Other	na

na = not available

[1] Domiciliary-care-worker-ENG.pdf (socialcare.wales) https://socialcare.wales/cms_assets/file-uploads/Domiciliary-care-worker-ENG.pdf

[2] The state of the adult social care sector and workforce in England (skillsforcare.org.uk) https://www.skillsforcare.org.uk/adult-social-care-workforce-data/Workforce-intelligence/publications/national-information/The-state-of-the-adult-social-care-sector-and-workforce-in-England.aspx

[3] Adults' services workforce tables - 2019 | Scottish Social Services Workforce Data (sssc.uk.com) https://data.sssc.uk.com/data-publications/248-adults-services-workforce-in-scotland-tables-2019

[4] Rapid learning review of domiciliary care in Northern Ireland (Department of Health) https://www.health-ni.gov.uk/rli-dom-care (page 13)

For peer review only

The original protocol for the study, as a supplementary file

For peer review only

For peer review only - http://bmjopen.bmj.com/site/about/guidelines.xhtml
